# The Complexity of Comparative Adsorption of C_6_ Hydrocarbons (Benzene, Cyclohexane, *n*-Hexane) at Metal–Organic Frameworks

**DOI:** 10.3390/nano12203614

**Published:** 2022-10-15

**Authors:** Christian Jansen, Nabil Assahub, Alex Spieß, Jun Liang, Alexa Schmitz, Shanghua Xing, Serkan Gökpinar, Christoph Janiak

**Affiliations:** 1 Institut für Anorganische Chemie und Strukturchemie, Heinrich-Heine-Universität, D-40225 Düsseldorf, Germany; 2 Microtrac Retsch GmbH, Retsch-Allee 1-5, D-42781 Haan, Germany

**Keywords:** metal–organic frameworks (MOFs), zeolitic imidazolate frameworks (ZIFs), vapor adsorption, C_6_ volatile organic compounds (VOCs), benzene adsorption, cyclohexane adsorption, *n*-hexane adsorption, IAST selectivity

## Abstract

The relatively stable MOFs Alfum, MIL-160, DUT-4, DUT-5, MIL-53-TDC, MIL-53, UiO-66, UiO-66-NH_2_, UiO-66(F)_4_, UiO-67, DUT-67, NH_2_-MIL-125, MIL-125, MIL-101(Cr), ZIF-8, ZIF-11 and ZIF-7 were studied for their C_6_ sorption properties. An understanding of the uptake of the larger C_6_ molecules cannot simply be achieved with surface area and pore volume (from N_2_ sorption) but involves the complex micropore structure of the MOF. The maximum adsorption capacity at p p_0_^−1^ = 0.9 was shown by DUT-4 for benzene, MIL-101(Cr) for cyclohexane and DUT-5 for *n*-hexane. In the low-pressure range from p p_0_^−1^ = 0.1 down to 0.05 the highest benzene uptake is given by DUT-5, DUT-67/UiO-67 and MIL-101(Cr), for cyclohexane and *n*-hexane by DUT-5, UiO-67 and MIL-101(Cr). The highest uptake capacity at p p_0_^−1^ = 0.02 was seen with MIL-53 for benzene, MIL-125 for cyclohexane and DUT-5 for *n*-hexane. DUT-5 and MIL-101(Cr) are the MOFs with the widest pore window openings/cross sections but the low-pressure uptake seems to be controlled by a complex combination of ligand and pore-size effect. IAST selectivities between the three binary mixtures show a finely tuned and difficult to predict interplay of pore window size with (critical) adsorptive size and possibly a role of electrostatics through functional groups such as NH_2_.

## 1. Introduction

Toxic volatile organic compounds (VOCs) in the atmosphere are of general concern and their removal, prevention of emission, and the separation of organic molecules in industrial processes [1] is technologically important [2,3,4,5,6,7,8,9,10,11,12]. A specific group of VOCs are hydrocarbons [11], which can be classified by the number of their carbon atoms. VOCs can be selectively adsorbed by specific materials. State of the art of VOC-removal by adsorption is the use of activated carbon [13], or zeolites [1]. However, the selectivity of activated carbon or zeolites for different VOCs is low, hence, components from a mixture of similar VOCs are difficult to separate by these standard adsorption materials. On the other hand, the design possibilities of metal-organic framework (MOF) adsorbents should allow for the separation of chemically similar VOC mixtures. MOFs are typically three-dimensional coordination networks with potential voids from the combination of metal clusters (secondary building units, SBUs) and organic ligands (called linkers) [14]. MOFs have already been investigated for the adsorption of VOCs such as *n*-hexane, xylene, toluene, acetaldehyde in vapor [7,15], liquid phase [16,17], or under humid conditions [18]. MOFs offer advantages [19] for VOC removal over materials [20] like zeolites [21], activated carbon [22], or silica gel [23]. A large variation of linkers and metal-atoms in MOFs [24] allows to adapt the pore size to the VOC to be adsorbed and separated [25]. The MOF linker can contain functional groups [26] for specific interactions to the VOC. The adsorptive selectivity of benzene over cyclohexane was improved with a nitro-decorated MOF which stabilized the interactions between the framework and benzene through a smaller window diameter with increased *π*···*π*-stacking and C-H···O (nitro) hydrogen bonding [27]. The presence of open-metal sites [28] could give an added high selectivity towards adsorptives. In the example of MOF-74 the adsorbate benzene can be arranged more structured at the open-metal sites than in bulk liquid benzene [29]. Such effects can be used to achieve a high affinity for benzene towards its separation from other VOCs. The flexibility of the framework in MOFs such as in MIL-53 with its breathing-effect [30], or in ZIF-7 with its gate-opening-effect is another opportunity to achieve a selective adsorption of a specific VOC [31,32,33,34]. By introducing defects in MOFs such as UiO-66, which is one of the most common defective MOFs [35], the maximum toluene adsorption capacity could be increased [36].

While the adsorption capacity and selectivity of MOFs for different VOCs has been widely tested, the stability of the MOF towards a VOC has been less examined. An important topic in VOC adsorption and separation deals with C_6_ hydrocarbons, e.g., benzene, cyclohexane and *n*-hexane. These three VOCs are among the ones found in indoor environments [37]. Benzene adsorption on MOFs is partly well examined [38,39]. In industry, the separation of benzene and cyclohexane by distillation is one of the most difficult cases due to their similar boiling point and vapor pressure [40,41]. The adsorption of *n*-hexane is particularly important with regard to its metabolization to nerve-damaging toxic 2,5-hexanedione [42]. The adsorption and separation of benzene, cyclohexane and *n*-hexane with MOFs has been examined with single vapor adsorption isotherms [43,44,45,46], in liquid phases [47] and with breakthrough experiments [48]. The recyclability and long-term stability of MOFs towards C_6_ vapor sorption has been seldom addressed and the focus is only on individual MOFs and no comparative investigations of a comprehensive series of MOFs [29,49].

Furthermore, only for some MOFs the ideal adsorbed solution theory (IAST) is used to simulate the separation properties of the MOF from single gas adsorption isotherms for a selective sorption of benzene over cyclohexane or different hexane isomers [50,51,52]. This method is a useful first approximation to study the separation properties and to obtain an overview of many different MOFs.

In this work, for the first time a broad series of MOFs with different metals were comparatively investigated for their adsorption of the C_6_-VOCs benzene, cyclohexane and *n*-hexane by volumetric sorption analysis, not so much for their maximum uptake capacity but especially for their uptake at low pressure down to p p_0_^−1^ = 0.02, to find MOFs for the removal of C_6_ traces. Furthermore, the stability after VOC adsorption and their IAST selectivities were taken into account. The stability of the MOFs was tested in liquid and vapor phase over a few days to simulate a potential long-term application.

## 2. Materials and Methods

All commercial chemicals were used as received (see Section S1 in the Supplementary Materials). The MOFs were synthesized according to literature reported procedures or optimized syntheses which are given in the Supplementary Materials.

Powder X-ray diffractometry (PXRD) was performed at ambient temperature on a D2 phaser (Bruker AXS, Karlsruhe, Germany) using Cu-K_α_ radiation (λ = 1.54182 Å) between 5° < 2*θ* < 50° with a scan rate of 0.0125° s^−1^ (300 W, 30 kV, 10 mA) and on a Miniflex 600 (Rigaku, Tokyo, Japan) using Cu-K_α_ radiation (λ = 1.54182 Å) between 2° < 2*θ* < 50° with a scan rate of 0.083° s^−1^ (600 W, 40 kV, 15 mA) and a D/teX ultra detector. Analyses of the diffractograms were carried out with Match 3.1.0 software. All PXRD patterns are collected in Sections S3 and S12.

Thermogravimetric analysis (TGA) was measured on a Netzsch TG209 F3 Tarsus (Netzsch, Selb, Germany) device under nitrogen atmosphere, ramping 10 K min^−1^ to 600 °C. TGA curves are given in Section S7.

Scanning electron microscopy (SEM) images were acquired on a JEOL JSM-6510 Advanced electron microscope (Jeol, Akishima, Japan) with a LaB_6_ cathode at 5–20 keV. The microscope was equipped with a Xflash 410 (Bruker, Billerica, MA, USA) silicon drift detector for energy-dispersive X-ray spectroscopy. SEM images are collected in Section S8.

Nitrogen sorption isotherms for the determination of Brunauer-Emmett-Teller [53] BET surface areas were obtained at 77 K within a pressure range of p = 10^−3^-1 bar on a Quantachrome NOVA (Quantachrome, Odelzhausen, Germany) instrument using ca. 20–50 mg of sample (nitrogen with purity of 99.999%, 5.0). Each sample was degassed under vacuum (<10^−2^ mbar) at 393 K for ca. 3 h prior to measurement using a FloVac (Quantachrome, Odelzhausen, Germany) degasser. All BET surface areas were calculated from five adsorption points applying Rouquerol plots (r > 0.998). All N_2_-sorption isotherms are shown in Section S4. Total pore volumes were calculated from the N_2_-sorption isotherm at p p_0_^−1^ = 0.9 for pore sizes ≤ 20 nm. NLDFT calculations were carried out with the native NovaWin 11.03 software using the ‘N_2_ at 77 K on carbon, slit pore, NLDFT equilibrium’ model. Thickness model for calculation of micropore volumes and micropore areas was set to ‘De Boer’. Micropore volumes were calculated from the N_2_ adsorption isotherm at p p_0_^−1^ = 0.1 for pores with d ≤ 2 nm (20 Å). Micropore volumes (V_micro_) were calculated by the t-plot method (‘De Boer’ model).

Vapor sorption experiments were carried out on a Quantachrome VStar4 (Quantachrome, Odelzhausen, Germany) instrument. Each sample was degassed under vacuum (<10^−3^ mbar) at 393 K for ca. 3 h prior to measurement, using a FloVac (Quantachrome, Odelzhausen, Germany) degasser. The detailed experimental equilibrium settings and all vapor sorption isotherms are given in Section S6. The measurement conditions had been set to achieve thermodynamic equilibrium with longer equilibrium times at low pressures and faster equilibration times in the saturated plateau region at higher relative pressures (Section S6). On average the time for the adsorption isotherm branch was 48 h, for the desorption branch 24 h. The vapor isotherms were recorded to only p p_0_^−1^ = 0.9 to avoid the vapor condensation when approaching p p_0_^−1^ = 1. The absolute pressures at 293 K were 75.26 Torr for benzene, 77.51 Torr for cyclohexane and 121.53 Torr for *n*-hexane.

The cyclic benzene adsorption experiment was performed at 298 K on a BELSORP-max II (HP model) (Microtrac MRB, Haan, Germany). The sample was pretreated under vacuum at 373 K for 3 h for a full adsorption cycle and evacuated at 298 K for 0.5 h for a short adsorption cycle. Adsorption points are measured at a relative pressure of about p p_0_^−1^ 0.3 with high dosing rates to reduce the measurement time (fluctuations in relative pressure possible).

Carbon dioxide sorption was measured with a Quantachrome Autosorb iQ MP (Quantachrome, Odelzhausen, Germany) at 273 K (ice/deionized water bath) (Section S13). The Autosorb iQ MP is equipped with oil-free vacuum pumps (ultimate vacuum <10^−8^ mbar) and valves, which guaranteed contamination free measurements. The sample was connected to the preparation port of the sorption analyzer and degassed under vacuum. After weighing, the sample tube was then transferred to the analysis port of the sorption analyzer. The samples were degassed before each measurement for a minimum of 3 h at 393 K under vacuum. The gas for the sorption measurements was of ultrapure grades (99.999%, 5.0) and the STP volumes are given at 273.15 K, 1 atm (1.013 bar). All carbon dioxide sorption isotherms are depicted in Section S5.

## 3. Results and Discussion

**Figure 1:**
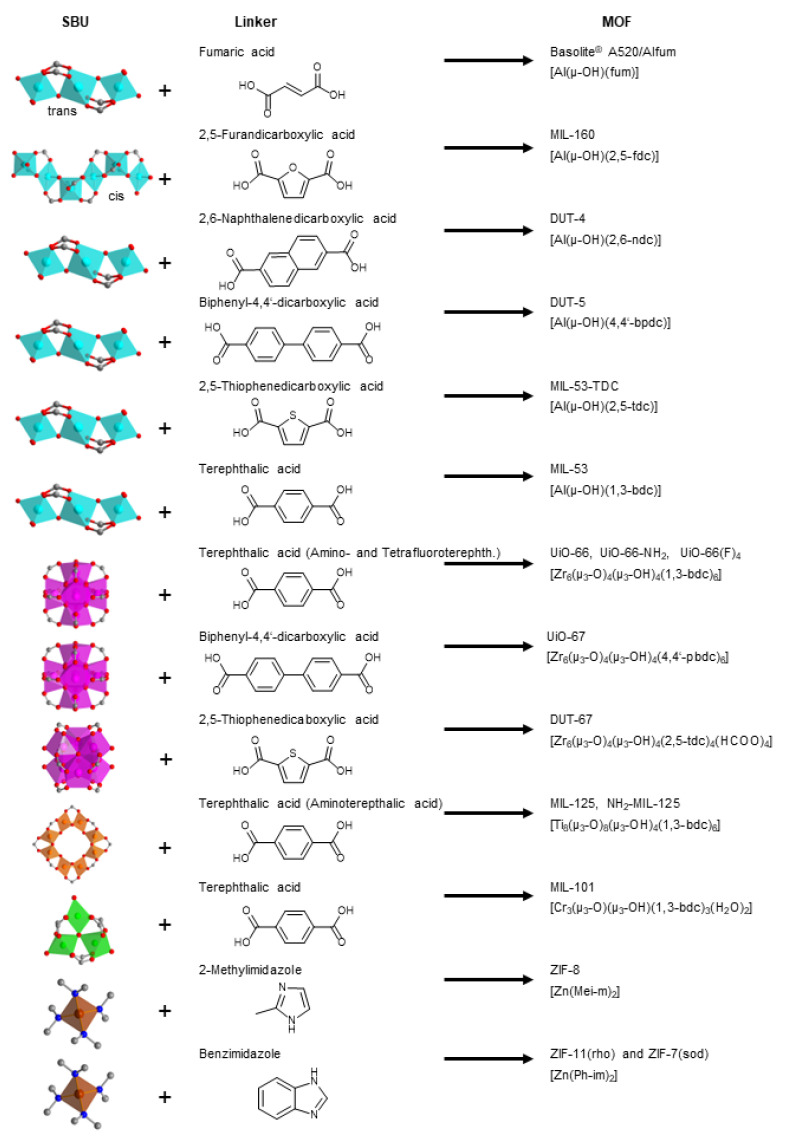
MOFs investigated here for C_6_-VOC adsorption. Secondary building units (SBUs) depict the metal atoms and their coordination polyhedra (Al cyan, Zr magenta, Ti orange, Cr green and Zn brown), which are connected via the linker (acid or imidazole form in the middle column) to form the respective MOFs (right column) (carbon gray, oxygen red, nitrogen blue). For further details to the MOFs see Section S2.

The seven aluminum-MOFs Basolite A520 [54] or Aflum [55], MIL-160 [56], DUT-4 [57], DUT-5 [58], MIL-53-TDC [59], MIL-53 [60], five zirconium-MOFs UiO-66, UiO-66-NH_2_ [61], UiO-66(F)_4_ [62], UiO-67 [63], DUT-67 [64], two titanium-MOFs MIL-125 [65] and MIL-125-NH_2_ [66], the chromium-MOF MIL-101(Cr) [67] and the three (Zn-)ZIFs ZIF-8 [68], ZIF-11 [69] and ZIF-7 [70,71], which are used for comparative C_6_ adsorption are depicted in Figure 1 with their secondary building unit, the linker, the acronym and their formula (MIL = Materials of Institute Lavoisier, DUT = Dresden University of Technology, TDC = thiophenedicarboxylate, UiO = Universitet i Oslo, ZIF = zeolitic imidazolate framework).

The MOFs can be considered as typical representatives which are frequently encountered in many studies. Powder X-ray diffractograms of the synthesized samples positively match with the simulations from the deposited structure files and thereby authenticate the crystalline phase (Figures S14–S24). The BET surface area and pore volumes from nitrogen sorption isotherms are in the range found in the literature (Table S2).

The three VOCs benzene, cyclohexane and *n*-hexane have six carbon atoms and no dipole moment, but different structures and electronic properties. In the gaseous phase the adsorptive benzene has a kinetic diameter of 5.85 Å, cyclohexane of 6 Å, and linear *n*-hexane of 4.3 Å [37]. The more specific van-der-Waals dimensions along the x, y and z axis of the molecule are for benzene x = 6.628 (MIN2), y = 7.337, z = 3.277 Å, cyclohexane x = 7.168, y = 6.580 (MIN2), z = 4.982 Å, *n*-hexane x = 10.344, y = 4.536 (MIN2), z = 4.014 Å with the value which is denoted as MIN2 being the critical dimension for a diffusion through cylindrical pore cross sections [72].

### 3.1. C_6_ Sorption Isotherms

**Figure 2:**
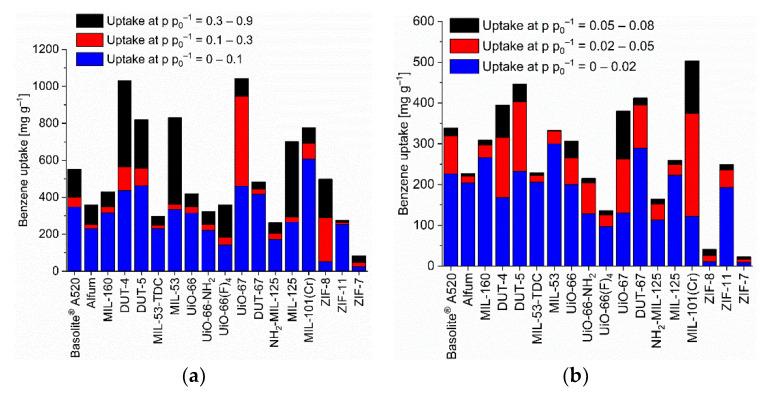
Benzene uptake capacity (at 293 K) of the different MOFs at different relative pressures. (**a**) At relative pressures of 0.1, 0.3 and 0.9; (**b**) at relative pressures of 0.02, 0.05 and 0.08 (specific values in Table S5). For the surface-specific uptake at p p_0_^−1^ = 0.1 see Figure S88a.

**Figure 3:**
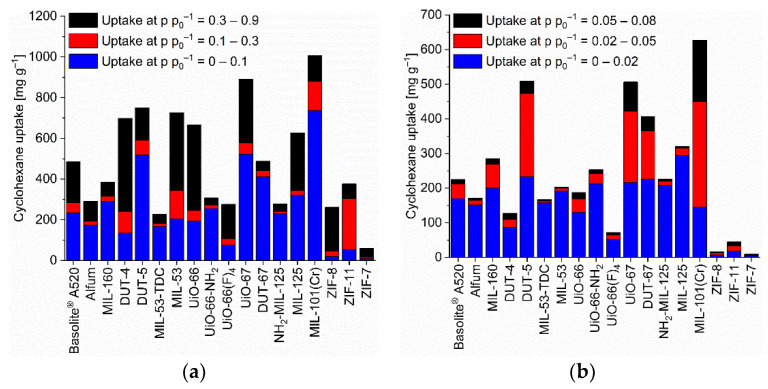
Cyclohexane uptake capacity (at 293 K) of the different MOFs at different relative pressures. (**a**) At relative pressures of 0.1, 0.3 and 0.9; (**b**) at relative pressures of 0.02, 0.05 and 0.08 (specific values in Table S6). For the surface-specific uptake at p p_0_^−1^ = 0.1 see Figure S88b.

**Figure 4:**
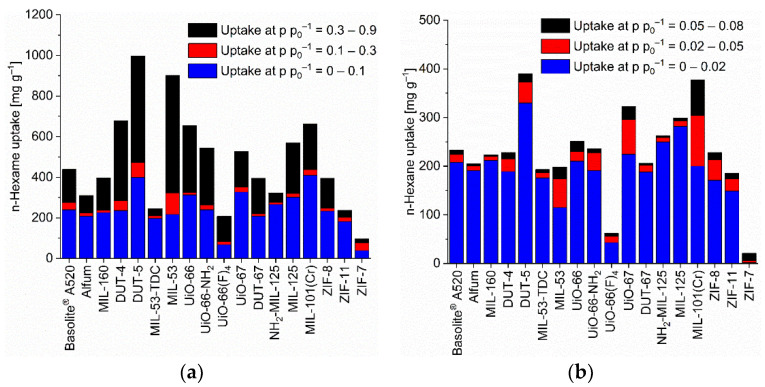
*n*-Hexane uptake capacity (at 293 K) of the different MOFs at different relative pressures. (**a**) At relative pressures of 0.1, 0.3 and 0.9; (**b**) at relative pressures of 0.02, 0.05 and 0.08 (specific values in Table S7). For the surface-specific uptake at p p_0_^−1^ = 0.1 see Figure S88c.

Most MOFs have adsorption isotherms towards the C_6_-VOCs which are a composite of Type-I and -II or have a Type-IV [53] or F-I adsorption isotherm [73]. The Type-I-II composite follows a Type-Ia, -Ib in the lower pressure section (p p_0_^−1^ < ~0.5) and a Type-II branch at higher relative pressure (Table S4, Figures S36–S46, for further details on the isotherm categorization). Most MOF-adsorbate pairs reach at least half of their maximum uptake at p p_0_^−1^ = 0.9 already at p p_0_^−1^ = 0.1, following Type-I, -F-I or -IV isotherms (Tables S4–S7, Figure 2, Figure 3 and Figure 4). There are only few MOF-adsorbate pairs with rather little adsorption at p p_0_^−1^ = 0.1 followed with a much larger uptake at higher pressure as evidenced by Type -II, -F-III or -V isotherms or in Type-F-I or -IV isotherms with much less uptake in the low versus the higher-pressure region. These few MOFs are ZIF-8 and ZIF-7 for benzene, ZIF-8, ZIF-11 and ZIF-7 for cyclohexane. Noteworthy, also UiO-66 and UiO-66(F)_4_ feature Type-I-II isotherms with only 1/3 of the cyclohexane uptake at p p_0_^−1^ 0.1 versus 0.9 and for UiO-66(F)_4_ also towards *n*-hexane (Tables S5–S7). This feature of a low uptake at p p_0_^−1^ = 0.1 is mostly accompanied by a wide hysteresis. For the ZIFs the low uptake at p p_0_^−1^ = 0.1 is due to a gate-opening effect in these materials with their narrow pore windows (cf. Section S12. For the UiO-66 compounds with their cluster or linker defects the guest may induce a distortion of the framework which then allows the accommodation of further adsorbates. Several isotherms exhibit a second gradual uptake step above ~0.4 p p_0_^−1^ followed by a saturation plateau such that the isotherm resembles an F-I or a Type-IV isotherm (Table S4, Figures S36–S46).

In the desorption branch the MOFs with the Type-II, Type-IV of F-I isotherm feature a wide or very wide hysteresis. Out of the 18 MOFs investigated here, 15 gave desorption isotherms with a wide or very wide hysteresis to the adsorption branch, at least for one of the vapors. Eight MOFs had a wide to very wide hysteresis to all three vapors. The visual appearance of the sorption isotherms differs also with the vapor for some MOFs. Upon further analysis (vide infra) there does not appear to be a unifying correlation of the isotherm shape with the MOF structure or vapor. In the literature benzene adsorptions in MOFs with a discernible hysteresis are interpreted with guest-host C-H···*π* or *π*···*π* interactions (Figure S128c) [74]. When no hysteresis is found this is seen to indicate that the molecules can move unrestricted in and out of the pores, as can be expected if the critical dimensions of the adsorptive molecule (for benzene x = 6.6 Å, for cyclohexane y = 6.6 Å, for *n*-hexane y = 4.5 Å) are smaller than the pore window cross sectional diameter [72].

It was beyond the scope of this work to theoretically study the adsorbate-adsorbent interactions in our MOFs. For example, the adsorption of benzene in MOF-74 at different pressure points was studied by Liu et al. [29]. The adsorption starts at 0.01 Pa with single molecules which lie flat on the adsorbent surface in separate pores. At 0.1 Pa, additional adsorbed benzene molecules begin to form a monolayer while other pores are still empty. At 0.2 Pa, benzene molecules are adsorbed in most pores. From 0.3 to 0.5 Pa the monolayers in each pore are almost complete. From 0.5 Pa multilayer adsorption with pore filling takes place which according to the calculations is completed at 20 Pa [29]. Macreadie et al. present the DFT-optimized location of one benzene or cyclohexane guest molecule in MOF-5, CUB-5 and 3DL-MOF-1 where the single molecule is located at the metal cluster nodes. The authors also note that aromatic MOFs favor aliphatic VOCs and vice versa due to the importance of aliphatic/aromatic C-H··· *π* interactions [43]. Yu et al. report the crystal structures of the MOF Al-tbbotb (tbbotb = 4,4’,4’’-(benzene-1,3,5-triyltris(oxy))tribenzoate) loaded with *n*-hexane or 3-methylpentane which are adsorbed in pairs along the adsorbent surface. There are close H···H contacts from the guests of 2.19 Å for *n*-hexane and 2.27 Å for 3-methylpentane to the bridging OH groups in the metal SBU [75]

We note that in the literature often only the adsorption branches are given and discussed while the desorption branch is not shown. It has been noted that desorption isotherms for vapors may be unreliable because the desorption process is accompanied by a transition from a saturated vapor or liquid state of the adsorbate to the gaseous adsorptive state [52].

Concerning the Type-II or Type-IV isotherm appearance and the presence of uptake steps in the adsorption branch at higher relative pressure this may be due to inter-particle condensation. From the cif files of the X-ray structures the solvent accessible void volume (in Å^3^/unit cell) was obtained, from which the theoretical specific pore volume could be calculated (Table S3). There is in most cases a reasonable match between the experimental pore volume and the calculated specific void volume. At the same time the vapor uptake in mg g^−1^ at p p_0_^−1^ = 0.9 can be transformed into the volume the adsorbate would use in a liquid state [cm^3^(liquid adsorbate) g^−1^(MOF)] by dividing the vapor uptake in mg g^−1^ through the density of the respective liquid at 293 K to approximate the volume of benzene, cyclohexane or *n-*hexane in the MOF (Table S3a–c). Further dividing this liquid adsorbate volume by either the experimental pore volume or the calculated specific void volume gives the degree of pore filling (Table S3). Remarkably, often a pore filling substantially above 100% is derived this way especially for those MOFs where the noted uptake steps are seen in the adsorption isotherm. Hence, we conclude that these uptake steps and concomitant larger-than-100% pore fillings are due to inter-particle condensation. The SEM images in Figures S67–S84 indicate the presence of fine powders with particle sizes below 5 µm and for the UiOs even below 0.5 µm.

### 3.2. C_6_ Uptake

The benzene adsorption capacity at p p_0_^−1^ = 0.9 and 293 K ranges between 262 to 1043 mg g^−1^ for the MOFs, with ZIF-7 (83 mg g^−1^) being a clear low-uptake outlier (Figure 2, Table S5). For cyclohexane the uptake at p p_0_^−1^ = 0.9 and 293 K extends from 227 to 1007 mg g^−1^ again with ZIF-7 (59 mg g^−1^) being significant lower (Figure 3, Table S6). For *n*-hexane under these conditions, the uptake lies between 207 to 997 mg g^−1^ (ZIF-7 at 96 mg g^−1^) (Figure 4, Table S7).

**Figure 5:**
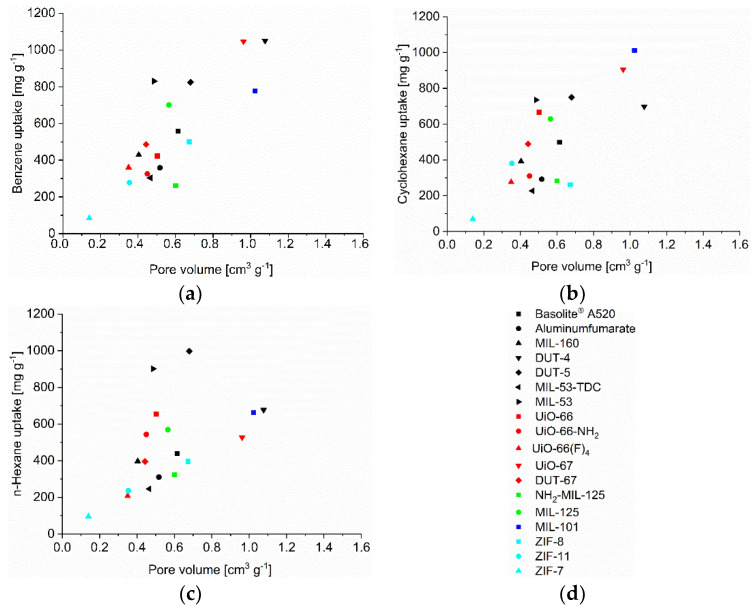
C_6_-VOC uptake at p p_0_^−1^ = 0.9 (293 K) versus pore volume (NLDFT and total pore volume, Table S2) for (**a**) benzene, (**b**) cyclohexane and (**c**) *n*-hexane with (**d**) legend to the figures.

The uptake of benzene, cyclohexane and *n*-hexane at p p_0_^−1^ = 0.9 follows roughly the experimental pore volume (Figure 5), BET surface area (Figure S85), pore window size (Figure S86) and micropore volume (Figure S87). The (total) pore volumes (Table S2) were obtained from NLDFT calculations using the ‘N_2_ at 77 K on carbon, slit pore, equilibrium’ model. This is noted above, when comparing the volume which the adsorbate would use in a liquid state to the available pore volume deviations to higher uptakes than expected from the pore volume are seen. These can be explained by inter-particle condensation and a slightly higher than expected uptake can also be due to framework transformations, adapting to the guest molecules and giving larger pores. MIL-53 with its known breathing effect is the best studied example [30]. Deviation to lower uptake can be due to solvent inaccessible pore regions which are smaller than the size of the adsorptive or where the access is prevented by gate-opening effects as in the ZIFs [31,76]. Thereby we note that the surface area and experimental pore volume have been determined from nitrogen sorption, with N_2_ having a kinetic diameter of (only) 3.64 Å. Adsorptives which are larger than N_2_ can be excluded from small pore regions which were included in the nitrogen measurements. The Connolly surface or probe accessible surface, as well as the accessible pore volume should be significantly smaller for larger adsorptives, either because pore windows become too small or pore regions with acute angles become inaccessible for bulkier molecules [53]. A simple correlation of C_6_ uptake with surface area and pore volume (from N_2_ sorption) cannot reflect this (in)accessibility of pores for larger adsorbates.

As noted in the introduction, for the adsorptive removal of C_6_ traces it is not so much the maximum uptake capacity but the uptake at low pressures which is important. Hence, Figure 2, Figure 3 and Figure 4 (cf. Tables S5–S7) also show the uptake in relative pressure regions down to p p_0_^−1^ = 0.02, in order to indicate the MOFs for the removal of C_6_ traces.

**Figure 6:**
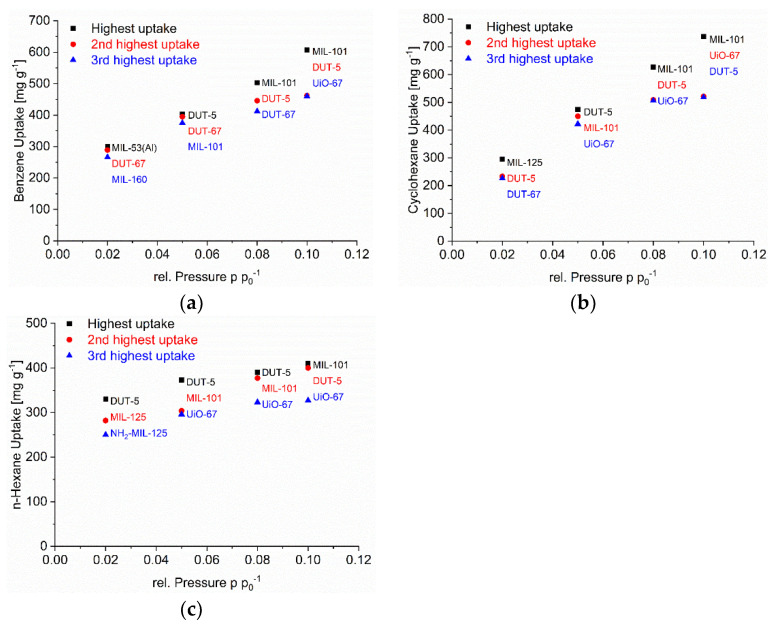
Top three MOFs with the highest C_6_ vapor at each relative pressure of p p_0_^−1^ = 0.1, 0.08, 0.05 and 0.02 for (**a**) benzene, (**b**) cyclohexane, and (**c**) *n*-hexane.

We have singled out three MOFs from Figure 2, Figure 3 and Figure 4 for each C_6_ vapor at each relative pressure of p p_0_^−1^ = 0.1, 0.08, 0.05 and 0.02 which gave the highest uptake values and collected them in Figure 6. For the C_6_ vapors a relative pressure of p p_0_^−1^ = 0.02 corresponds to an absolute pressure of 1.5 Torr (0.20 kPa) for benzene, 1.55 Torr (0.21 kPa) for cyclohexane and 2.43 Torr (0.32 kPa) for *n*-hexane (Table S8). Remarkably, in the pressure range from p p_0_^−1^ = 0.1 down to 0.05 it is pretty much the same three MOFs for each vapor. For benzene, these are DUT-5, DUT-67/UiO-67 and MIL-101(Cr), for cyclohexane and *n*-hexane it is DUT-5, UiO-67 and MIL-101(Cr). DUT-5 and MIL-101(Cr) are always among the top-3 in the range from p p_0_^−1^ = 0.1 to 0.05. The third MOF is then either DUT-67 or UiO-67.

The MOFs DUT-5 and UiO-67 have the same long biphenyl-4,4’-dicarboxylate linker (Figure 1). At the same time DUT-4 with its also long naphthalene-2,6-dicarboxylate linker does not feature a high uptake at low pressure. MIL-101(Cr) contains the common terephthalate linker which is also part of other MOFs without high uptake. DUT-5 and MIL-101(Cr) are the MOFs with the widest pore window openings/cross sections (Table S2). Notably DUT-67 (with the TDC = thiophenedicarboxylate linker) and UiO-67 already have smaller pore windows which are in the range of DUT-4, MIL-53-TDC and MIL-53 with the latter having lower uptakes in this pressure region. This comparison illustrates that the low-pressure uptake seems to be controlled by a complex combination of ligand and pore-size effect.

The interpretation of the uptake at low pressure is further complicated by the observation that at the lowest pressure of p p_0_^−1^ = 0.02 one or two other MOFs come in which were not among the top three above p p_0_^−1^ = 0.02. For benzene MIL-160, MIL-53 and DUT-67, for cyclohexane DUT-5, DUT-67 and MIL-125, for *n*-hexane DUT-5, MIL-125 and NH_2_-MIL-125 performed best at p p_0_^−1^ = 0.02. Hence, only DUT-5 and/or DUT-67 still remain in the top-3 lists at p p_0_^−1^ = 0.02. The other top three MOFs now have smaller pore cross sections of 5-6 Å (MIL-160, MIL-53 edge-edge distance, MIL-125 and NH_2_-MIL-125). This cross-section diameter corresponds to the intermediate (critical) van-der-Waals dimensions for benzene of x = 6.628 Å, cyclohexane y = 6.580 Å and *n*-hexane y = 4.536 Å (vide supra) [72]. At this cross-section range, one can assume the C_6_ molecules to have dispersive interactions with multiple sides of the molecule to the surface. Such local optima exist for adsorbent structures where the opposite Connolly surfaces are separated by the dimension of the adsorbed molecule which can then simultaneously interact with the surface at its opposite sides [53]. For the long *n*-hexane molecule this multiple-side interaction will probably involve different sections of the chain.

The low-pressure benzene sorption capacity of the top-3 MOFs here is lower than the top literature examples with the MOFs BUT-53 to BUT-58 with 193–256 mg g^−1^ (2.47–3.28 mmol g^−1^) at 298 K, <10 Pa (p p_0_^−1^ < 0.001) (Type-Ia isotherm) [77] or the benchmark materials MOF-5 with 802 mg g^−1^ at 295 K [78] or [Zn_4_O(bdc)(bpz)_2_] with 561 mg g^−1^ at 298 K and p p_0_^−1^ = 0.1 [74] to which only MIL-101(Cr) with 607 mg g^−1^ at 293 K and p p_0_^−1^ = 0.1 comes close (Table S2, Figure 2).

Absolute cyclohexane uptakes are difficult to compare to literature data, as most studies aim to achieve benzene/cyclohexane selectivities as high as possible, and thus the reported MOFs feature often very low cyclohexane uptakes as in the work of Mukherjee et al. where the M-MOF-74 analogues have nearly no cyclohexane uptake at all [51]. On the other hand, Eddaoudi et al. tested MOF-5 for cyclohexane and benzene sorption and reached values between 600 and 800 mg g^−1^ at p p_0_^−1^ = 1 for both VOCs at 295 K [79]. A cyclohexane uptake above 600 mg g^−1^ at p p_0_^−1^ = 0.9 (293 K) is reached with DUT-4, DUT-5, MIL-53, UiO-66, UiO-67, MIL-125 and MIL-101(Cr) (Table S2, Figure 3).

In the literature uptake values for *n*-hexane near p p_0_^−1^ = 1 were noted as high for MOF-5 (249 mg g^−1^ at 298 K), MIL-101(Cr) (504 mg g^−1^ at 298 K), or Cu-BTC (175 mg g^−1^ at 303 K) [80]. Here, an *n*-hexane uptake above 500 mg g^−1^ at p p_0_^−1^ = 0.9 (293 K) is seen with DUT-4, DUT-5, MIL-53, UiO-66, UiO-66-NH_2_, UiO-67, MIL-125 and MIL-101(Cr) (Table S2, Figure 4). We emphasize again that the uptake at p p_0_^−1^ = 0.9 may involve condensation in inter-particle mesopores (vide supra).

### 3.3. Comparative C_6_ Uptake

With respect to the three VOCs the comparative adsorption capacity and uptake order at, for example, p p_0_^−1^ = 0.1 is depending on the individual MOF and follows no unifying trend (Table S9, because of the possible inter-particle condensation we refrain from comparing the uptake at p p_0_^−1^ = 0.9). For the surface-specific vapor uptake at p p_0_^−1^ = 0.1 see Figure S88 and discussion in Section S9. Out of the 18 MOFs investigated here (at p p_0_^−1^ = 0.1), 10 have benzene, 5 cyclohexane and 3 *n-*hexane as VOC with the highest uptake (Tables S5–S7). Furthermore, benzene being lowest in uptake is only seen for 3 MOFs at p p_0_^−1^ = 0.1. At p p_0_^−1^ = 0.1 the most frequent order with seven MOFs is benzene > *n-*hexane > cyclohexane; for another three each it is benzene > cyclohexane > *n-*hexane and cyclohexane > benzene > *n-*hexane. In the liquid phase the densities are 0.876 g cm^−3^ for benzene, 0.779 g cm^−3^ for cyclohexane and 0.655 g cm^−3^ for *n*-hexane (at 293 K). Thus, if an available porosity is filled with the adsorbate in a liquid-like state, the specific uptake in g(adsorbate)/g(adsorbent) should be highest for benzene because of its highest density, followed by cyclohexane. Hence, only in four MOFs the uptake follows the reciprocal kinetic diameter (*n-*hexane < benzene < cyclohexane), that is, the smallest adsorbate is preferentially adsorbed only in a few materials. This confirms that the diffusion of the adsorptives in the MOF pore system is not influencing the data and that our measurement conditions ensured thermodynamic equilibrium.

The preferentially higher adsorption of benzene over cyclohexane and *n-*hexane is rationalized through the *π*···*π* or C-H···*π* interactions of benzene (Figure S128c) with the framework and its higher density in a liquid-like adsorbate state for a given pore volume. When *n*-hexane shows higher adsorption capacity at p p_0_^−1^ = 0.1 as in NH_2_-MIL-125, ZIF-8 and ZIF-7 (Table S7) it may be tempting to invoke its linear structure and a more favorable packing of adsorbed molecules [37]. However, the liquid state density for *n*-hexane is significantly lower than that for cyclohexane and benzene (vide supra). Thus, higher *n*-hexane adsorption can in our opinion and in the absence of special adsorbate-adsorbent interaction only be explained from its small critical diameter in the series (vide supra). Hence, *n*-hexane can enter (slit) pores and pore window regions which are inaccessible for larger cyclohexane and benzene molecules. The surface area and pore volume from nitrogen sorption will not be fully accessible for larger molecules [53]. However, while one may indeed have such small pore regions together with gate-opening effects in ZIF-8 and ZIF-7 with pore windows near 3 Å (Table S2, unnumbered images in Section S15) this is difficult to see in NH_2_-MIL-125 which has pore windows of 6 Å as many other MOFs (Table S2). Additionally, ZIF-11 has a 3 Å pore window and the same benzimidazole ligand as ZIF-7, but gives a higher amount of benzene than *n*-hexane uptake at p p_0_^−1^ = 0.1. Thus, an understanding of the C_6_ uptake cannot simply be achieved with surface area and pore volume (from N_2_ sorption or CO_2_ sorption) but involves the complex micropore structure of the MOF.

### 3.4. Separation–IAST Selectivity

The ideal adsorbed solution theory (IAST) model simulates mixed gas/vapor adsorption behavior and selectivity from single adsorption isotherms (see Sections S10 and S11, for details). Benzene/cyclohexane separation can be regarded as the industrially most interesting process as their separation by distillation is one of the most difficult cases due to similar boiling points and vapor pressures. For comparison equimolar mixtures are investigated of all three C_6_-VOC combinations.

**Figure 7:**
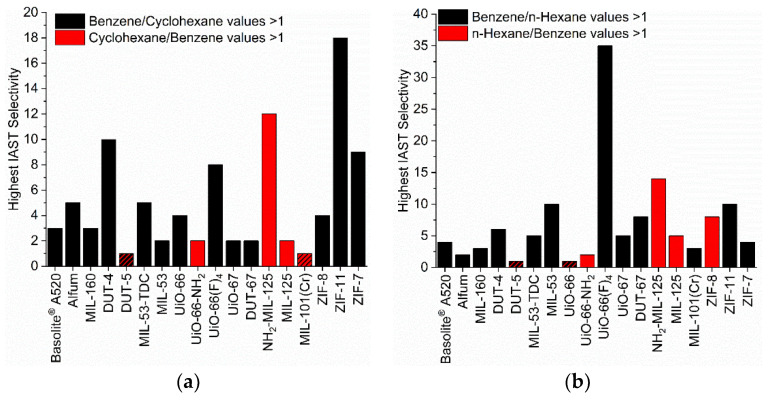

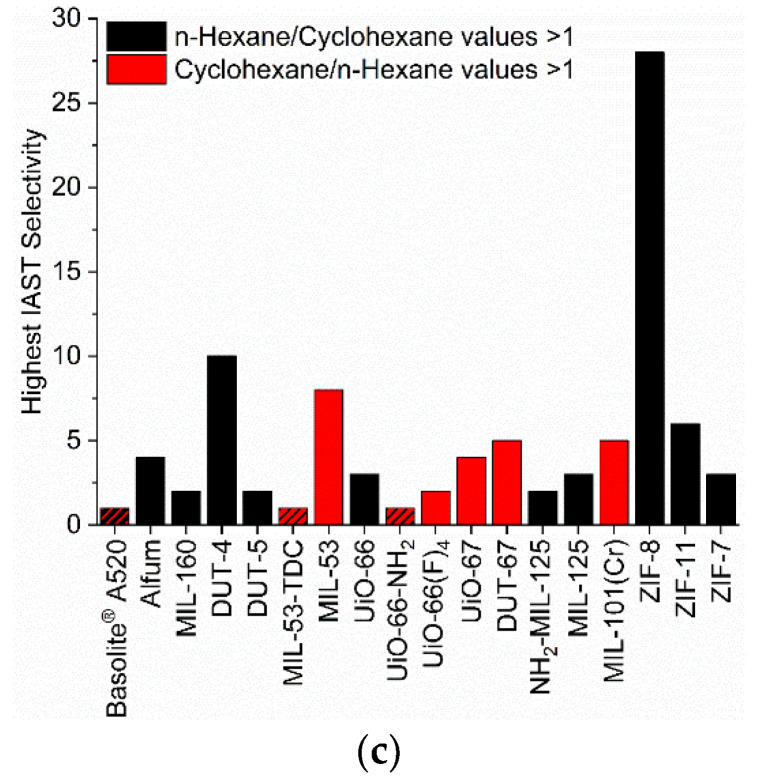
Highest values of the IAST selectivities from the three points at 0.01, 0.05 and 0.09 bar of each MOF for the different VOC pairs (50/50 molar ratio (**a**) benzene/cyclohexane, (**b**) benzene/*n-*hexane and (**c**) cyclohexane/*n*-hexane). The IAST selectivities of 1 are visualized via crosshatched bars. For the IAST-calculated selectivity dependence with pressure, see Figures S102–S112.

In the pairs benzene/cyclohexane, benzene/*n-*hexane and *n-*hexane/cyclohexane the first given adsorptive is the preferred one for most MOFs (Figure 7, black bars). The opposite selectivity, that is a preference for cyclohexane over benzene etc. is seen in fewer MOFs. At the same time, the selectivity value of most investigated MOFs is below 6, although selectivity values higher than 3 would already be sufficient for an adsorbent to be applicable in an industrial separation process [37,81].

The selectivity changes with pressure (Figures S102–S112). Figure 7 plots only the highest selectivity value from three chosen points at 0.01, 0.05 and 0.09 bar (Table S12). We note that selectivities could be much higher for some MOFs, especially for the ZIFs below 0.01 bar, but in this very low pressure range the selectivity can also be strongly influenced from isotherm fitting errors. Relative maxima in the IAST selectivity between the three pressure points were in the error range of the calculation.

When we look at selectivities larger than 10, we find NH_2_-MIL-125 with a value of 12 for cyclohexane/benzene and with a value of 14 for *n*-hexane/benzene. Obviously, this MOF discriminates well against benzene. NH_2_-MIL-125 is also the only MOF with a high preference of the alkanes over benzene. The next best candidate is ZIF-8 which however significantly (value of 8) prefers *n-*hexane over benzene only at 0.01 bar. We trace the high alkane selectivity of MIL-125-NH_2_ to the presence of the amino group in combination with the pore size. The other amino-MOF UiO-66-NH_2_ also favors the alkanes over benzene, albeit only slightly. The pore window of UiO-66-NH_2_ (7.0 × 7.0 Å^2^) is slightly larger than in NH_2_-MIL-125 (5.9 × 5.9 Å^2^) so that the effect of NH_2_ could be less pronounced. At the same time, also MIL-125 without the NH_2_ group still slightly favors the alkanes over benzene with an only somewhat larger pore window of 6.1 × 6.1 Å^2^. UiO-66 with the same pore size as NH_2_-MIL-125 favors either benzene over cyclohexane or has little to no separation effect on the *n*-hexane/benzene mixture.

Further, ZIF-11 has a selectivity of 18 for benzene/cyclohexane and UiO-66(F)_4_ of 13–35 for benzene/*n*-hexane which may be traced to the small pore window cross-sections of 3.0 × 3.0 Å^2^ and 2.4 × 4.6 Å^2^ respectively (Table S2). In view of the several times noted critical dimensions (for benzene x = 6.628 Å, for cyclohexane y = 6.580 Å, for *n*-hexane y = 4.536 Å) it is difficult to see why benzene can pass and cyclohexane or *n*-hexane are excluded. At the same time, the only slightly smaller window size in ZIF-7 of 2.9 × 2.9 Å^2^ still leads to a selectivity of 9 for benzene/cyclohexane but appears to already hinder the entry of benzene when compared to ZIF-11 (cf. Figure 2).

For a benzene/cyclohexane mixture, the MOF DUT-4 and for a benzene/*n*-hexane mixture the MOFs MIL-53 and ZIF-11 give selectivities around 10. The square-channel MOFs DUT-4 and MIL-53 have similar pore windows of 8.5 × 8.5 Å^2^. The dimensions obviously allow for the energetically favorable C-H···*π* arrangement of the benzene adsorbate with the aromatic walls of the adsorbent (Figure S128c). We note that for a benzene dimer the energy of the *π*···*π* stacking interaction is 2.73 kcal mol^−1^, whereas the C-H···*π* T-shape is more stable with an interaction energy of 2.84 kcal mol^−1^ [82]. Additionally, the crystal structures of benzene all show the herringbone packing with C-H···*π* interactions (Section S14, Figure S128).

The role of the benzimidazole linker in ZIF-11 and ZIF-7 towards possibly engaging in *π*···*π* or C-H···*π* interactions with the benzene adsorbate (Figure S128c) cannot be very significant as the selectivity of both ZIFs for benzene/*n*-hexane is much lower (10 and 4, respectively) than for benzene/cyclohexane.

A selectivity of *n*-hexane over cyclohexane is most pronounced for ZIF-8 and is at the same time also the second highest calculated selectivity in the chosen pressure region. The *n-*hexane/cyclohexane selectivity of ZIF-8 can be correlated to the small pore window size and its gate-opening effects. For DUT-4 a pronounced *n*-hexane/cyclohexane selectivity is only present in the low-pressure range.

The separation of benzene/cyclohexane, benzene/*n*-hexane etc. with selectivities of 10 and above (Figure 7 and Section S11) show again a finely tuned and difficult to predict interplay of pore window size with (critical) adsorptive size and possibly a role of electrostatics through functional groups such as NH_2_.

### 3.5. Cycling Adsorption Runs

**Figure 8:**
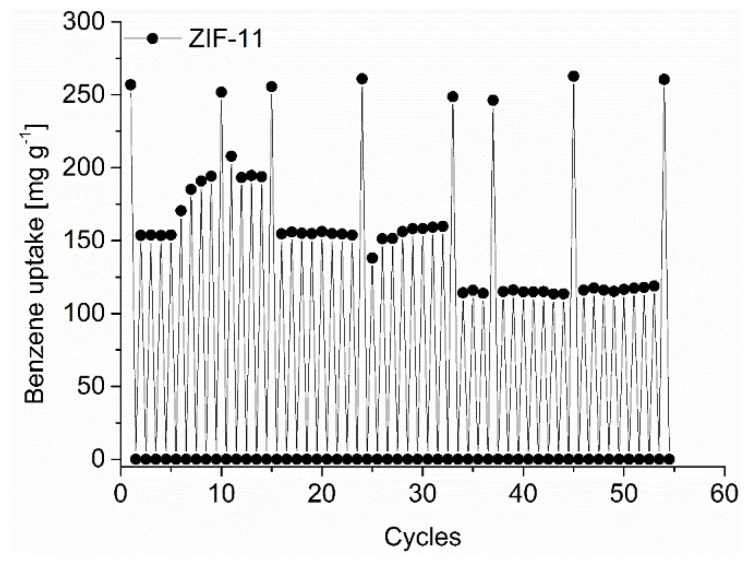
Benzene cycling test of ZIF-11 with 55 ad/desorption cycles up to a relative pressure of p p_0_^−1^ = 0.3 and a full adsorption measured every few cycles and at the end of the 55 cycles.

The MOFs were found stable towards the liquid and gaseous C_6_-VOCs from PXRD and nitrogen sorption analysis after 5 days of contact with the VOC and re-activation (see Section S12 for details). For ZIF-11 we also performed a cycling adsorption tests for benzene to check for the cycling stability and uptake consistency. (Figure 8).

To increase the rate of the cycling process the measurements were mostly performed only up to a relative pressure of p p_0_^−1^ 0.3. Within the in total 54 cycles depicted in Figure 8 we also did eight full adsorption cycles. The full adsorption measurements did not show a decrease in the initial benzene uptake of about 250 mg g^−1^. However, the more rapid short adsorption cycles with evacuation at 298 K for only 0.5 h and high dosing rates upon adsorption show a fluctuation. The short-time cycling starts with a benzene uptake of 150 mg g^−1^ under these conditions. This uptake value started to increase after 5 cycles to near 200 mg g^−1^ which could be due to an enhanced activation through residual solvent removal from the repeated activation. However, after 15 total cycles the benzene uptake returned to 150 mg g^−1^ at which value it stayed until cycle 32 (Figure 8). From cycle 34 onwards, the uptake dropped to 120 mg g^−1^ where it stayed rather constant until cycle 53. However, a subsequent full adsorption run gave the initial 250 mg g^−1^ uptake.

## 4. Conclusions

The C_6_ volatile organic compounds (VOCs) benzene, cyclohexane and *n*-hexane are important industrial products, but toxic for humans or the environment and need to be removed from water and air. This removal is an important part of industrial processes as well. Benzene is classified as a carcinogen, whereas cyclohexane causes dermatitis and *n*-hexane nerve damage. Metal–organic frameworks (MOFs) are promising candidates for toxic gas and vapor removal. In this work, for 18 different MOFs and ZIFs (15 MOFs and 3 ZIFs) the maximum VOC uptake capacity and the long-term stability were tested and correlated with the properties of the different MOFs. The analyzed properties were metal-source, linker, pore volume, micropore volume, pore window and BET surface area. The uptake capacity was tested via volumetric adsorption measurements at 293 K and the long-term stability was tested under liquid and vapor conditions at room temperature. All investigated MOFs proved stable against the C_6_-VOCs. Out of the 18 MOFs investigated here, 15 gave desorption isotherms with a wide or very wide hysteresis to the adsorption branch, at least for one of the vapors. Most MOF-adsorbate pairs reach at least half of their maximum uptake at p p_0_^−1^ = 0.9 already at p p_0_^−1^ = 0.1 which is the upper end for the uptake of vapor traces. Already above a relative pressure (p p_0_^−1^) of about 0.45 interparticle condensation takes place. Even at lower relative pressure—which is important for trace vapor removal—there is no single parameter, such as surface area, pore volume or pore dimensions, which correlates with the uptake or selectivity. From the 18 MOFs, 10 have benzene, 5 have cyclohexane and 3 have *n-*hexane as VOC with the highest uptake (at p p_0_^−1^ = 0.1). The VOC uptake correlates only roughly with pore volume, BET surface area or pore window and micropore volume (obtained from N_2_ sorption). From a comparison of the MOF pore volume with the approximated volume of the adsorbate in a liquid state, a pore filling substantially above 100% is seen, which is explained by inter-particle condensation. The best correlation is between the benzene uptake and pore volume. The widest spread is the correlation between VOC uptake and BET surface area. Another interesting correlation is between the pore window and the uptake. While it could be expected that large pore cross sections and large pore volumes generate a high uptake, it was remarkable to see that even MOFs with pore window sizes smaller than the diameter of the adsorbent molecules can give a sizeable uptake. For a good selectivity, MOFs in particular with such a small pore window cross section feature prominently.

To a surprise, MOF with an amino group on the ligand was also highly discriminating against benzene, that is, it gave higher cyclohexane and *n*-hexane uptakes. No uptake is seen in ZIF-7 for benzene and cyclohexane; only the smaller *n*-hexane has access to the pore. The influence of the metal does not affect the sorption properties, as was to be expected and was not further investigated. The simulated IAST selectivities of the MOFs show a necessity towards small pore windows or diameters for high selectivity values, as found for ZIFs (ZIF-8, ZIF-11 and ZIF-7). However, the interplay between pore window size and (critical) size of the adsorbate and possibly the role of functional groups such as NH_2_ require more in-depth investigations for a more complete understanding.

## Data Availability

The data presented in this study are available on request from the corresponding author.

## Supplementary Materials

The following supporting information can be downloaded at: https://www.mdpi.com/article/10.3390/nano12203614/s1. S1 Materials and equipment; S2 MOF syntheses, crystal structures and MOF parameters; S3 Powder X-ray diffraction (PXRD) measurements; S4 Nitrogen sorption experiments (T = 77 K); S5 Carbon dioxide experiments (T = 273 K); S6 Vapor sorption experiments (T = 293 K); S7 Thermogravimetric analysis (TGA); S8 Scanning electron microscopy (SEM); S9 VOC uptake versus BET surface, pore window size, micropore volume; S10 VOC sorption studies; S11 Ideal Adsorbed Solution Theory, IAST-Selectivity; S12 Stability tests; S13 Gas Sorption at 293 K; S14 Crystal structures of benzene; S15 Images from ‘Mercury’ – Display Voids’ calculation; S16 References. References [83,84,85,86,87,88,89,90,91,92,93,94,95,96,97,98,99,100,101,102,103,104,105,106,107,108,109,110] are cited in the Supplementary Materials.
