# Supplementary material for: The Complexity of Comparative Adsorption of C6 Hydrocarbons (Benzene, Cyclohexane, n-Hexane) at Metal–Organic Frameworks
Source: Nanomaterials (Basel). 2022 Oct 15;12(20):3614. doi: 10.3390/nano12203614 (PMC9610754; doi:10.3390/nano12203614)
Supplement: Supplementary file 1 [file nanomaterials-12-03614-s001.zip › nanomaterials-1923607-supplementary.pdf]

Electronic Supporting Information (ESI)

The Complexity of Comparative Adsorption of C_6 Hydrocarbons (Benzene, Cyclohexane, *n*-Hexane) at Metal–Organic Frameworks

Christian Jansen [1], Nabil Assahub [1], Alex Spieß [1], Jun Liang [1], Alexa Schmitz [1], Shanghua Xing [1], Serkan Gökpinar [2] and Christoph Janiak [1,*]

[1] Institut für Anorganische Chemie und Strukturchemie, Heinrich-Heine-Universität, D-40225 Düsseldorf, Germany
[2] Microtrac Retsch GmbH, Retsch-Allee 1-5, D-42781 Haan, Germany
* Correspondence: janiak@hhu.de

Keywords

Metal-organic frameworks (MOFs), zeolitic imidazolate frameworks (ZIFs), vapor adsorption, C_6 volatile organic compounds (VOCs), adsorption, stability, IAST selectivity

Table of Contents

S1. Materials and equipment 2

S2. MOF syntheses, crystal structures and MOF parameters 3

S3 Powder X-ray diffraction (PXRD) measurements 16

S4 Nitrogen sorption experiments (T = 77 K) 20

S5 Carbon dioxide sorption experiments (T = 273 K) 23

S6 Vapor sorption experiments (T = 293 K) 24

S7 Thermogravimetric analysis (TGA) 43

S8 Scanning electron microscopy (SEM) 45

S9 VOC uptake versus BET surface, pore window size, micropore volume 51

S10 VOC sorption studies 56

S11 Ideal Adsorbed Solution Theory, IAST-Selectivity 64

S12 Stability tests 71

S13 Gas Sorption at 293 K 77

S14 Crystal structures of benzene 78

S15 Images from 'Mercury – Display Voids' calculation 78

S16 References 93

Citation: Jansen, C.; Assahub, N.; Spieß, A.; Liang, J.; Schmitz, A.; Xing, S.; Gökpinar, S.; Janiak, C. The Complexity of Comparative Adsorption of C_6 Hydrocarbons (Benzene, Cyclohexane, *n*-Hexane) at Metal–Organic Frameworks. *Nanomaterials* **2022**, *12*, 3614. https://doi.org/10.3390/nano12203614

Academic Editors: Bin Li and Hui-Min Wen

Received: 2 September 2022
Accepted: 10 October 2022
Published: 15 October 2022

Publisher's Note: MDPI stays neutral with regard to jurisdictional claims in published maps and institutional affiliations.

Copyright: © 2022 by the authors. Submitted for possible open access publication under the terms and conditions of the Creative Commons Attribution (CC BY) license (https://creativecommons.org/licenses/by/4.0/).

S1. Materials and equipment

All chemicals were used as received by the supplier (*cf.* Table S1).

Table S1 *Used chemicals, supplier and purities.*

**Chemical**	**CAS-Nr.:**	**Supplier**	**Purity [%]**
Acetic acid	64-19-7	Sigma Aldrich	≥ 99.8
Aluminum chloride hexahydrate	7784-13-6	Alfa Aesar	99
Aluminumfumarate (Basolite® A520)	not specified	BASF	not specified
Aluminum nitrate nonahydrate	7784-27-2	Carl-Roth	≥ 98
Aluminum sulfate octadecahydrate	7784-31-8	Sigma Aldrich	≥ 98
Ammonia hydroxide	7664-41-7	VWR Chemicals	25
Benzene	71-43-2	AppliChem	p.a.
Benzimidazole	51-17-2	Sigma Aldrich	98
Biphenyl-4,4´-dicarboxylic acid	787-70-2	Abcr GmbH	98
Chromium nitrate nonahydrate	7789-02-8	Acros Organics	99
Cyclohexane	110-82-7	AppliChem	p.a.
Dichloromethane	75-09-2	Fischer Chemicals	≥ 99.8
Ethanol	64-17-5	Merck	p.a.
Fumaric acid	110-17-8	TCI	≥ 99
n-Hexane	110-54-3	VWR Chemicals	98
Hydrochloric acid	7647-01-0	Sigma Aldrich	≥ 37
Methanol	67-56-1	Sigma Aldrich	≥ 99.8
Nitric acid	7697-37-2	Fischer Chemicals	65
N-Methyl-2-pyrrolidione	872-50-4	Carl-Roth	≥ 99.8
N,N-Dimethylformamide	68-12-2	Honeywell	≥ 99.8
Sodium hydroxide microgranulate	1310-73-2	Chemsolute	99.5
Terephthalic acid	100-21-0	Alfa Aesar	98+
Toluene	108-88-3	Sigma Aldrich	99.8
Titanium(IV)isopropoxide	546-68-9	Sigma Aldrich	97
Zinc acetate dihydrate	5970-45-6	Carl-Roth	98+
Zinc nitrate hexahydrate	10196-18-6	Acros Organics	98
Zirconium chloride	10026-11-6	Chempur	98
2-Aminoterephthalic acid	10312-55-7	Acros Organics	99
2-Methylimidazole	693-98-1	Acros Organics	99
2,3,5,6-Tetrafluoroterephthalic acid	652-36-8	BLDpharm	97
2,5-Furandicarboxylic acid	3238-40-2	BLDpharm	99.25
2,5-Thiophenedicarboxylic acid	4282-31-9	BLDpharm	98.14
2,6-Naphthalenedicarboxylic acid	1141-38-4	Alfa Aesar	98+

S2. MOF syntheses, crystal structures and MOF parameters

Al-MOFs

Aluminum-MOFs show in general a good thermal and chemical stability against water and other chemicals, which makes them suitable candidates for sorption applications.

Basolite® A520 (commercial Aluminumfumarate) was purchased by BASF (BET = 988 $m^2 g^{-1}$).

The synthesis of **Aluminumfumarate** was carried out according to the procedure of Alvarez *et al.*[1] $Al_2(SO_4)_3$ $18H_2O$ (7.00 g, 10.50 mmol, 1 eq), sodium hydroxide (2.52 g, 63 mmol, 6 eq) and fumaric acid (3.36 g, 28.95 mmol, 3 eq) were educts. In a first step, the $Al_2(SO_4)_3$ $18H_2O$ was dissolved in 30 mL deionized water at 60 °C. while stirring. The fumaric acid and sodium hydroxide were dissolved in 36 mL of deionized water. In a further step, the fumaric acid-NaOH solution was added dropwise to the $Al_2(SO_4)_3$ $18H_2O$ solution over the period of 30 minutes. The reaction mixture was stirred for a further two hours at 60 °C. After that the temperature was turned off and the solution was cooled while stirring. The solid was washed three times with 60 mL deionized water (one time overnight). The product was centrifuged (10000 U min^{-1}, 15 min), decanted and placed in the vacuum oven (60 ° C, 1-10 mbar) to dry overnight. Yielding a white powder (3.38 g yield, BET = 1035 $m^2 g^{-1}$).

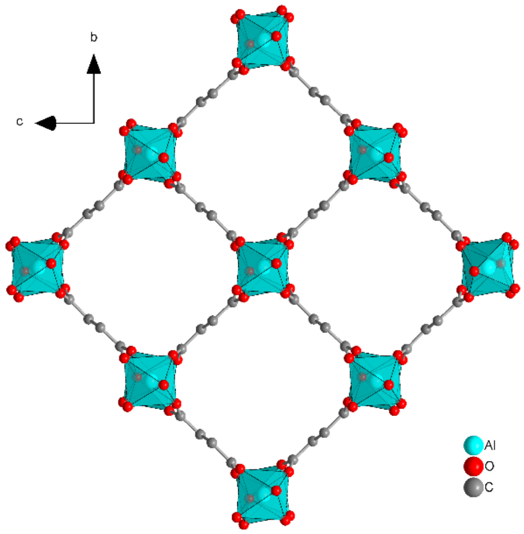

Figure S1 *The 3D framework structure of Alfum exhibits square shaped one-dimensional channels. Graphic produced by software Diamond[2] from cif-file for Aluminumfumarate (CSD-Number: 1051975).[1]*

The synthesis of **MIL-160** was carried out following a modified protocol of Cadiau *et al.*[3] who used 1 eq NaOH, whereas we used 2 eq NaOH to deprotonate the linker fully: 2,5-furandicarboxylic acid (4.6814 g, 30.0 mmol, 1 eq) and sodium hydroxide (2.4032 g, 60.08 mmol, 2 eq) were converted in water (150 mL, 1.5 h). $AlCl_3{\cdot}6H_2O$ (7.2457 g, 30.01 mmol, 1 eq) was added and reflux (24 h, 100 °C) was initiated. After decantation, the product was washed with water three times (160 mL each), centrifuged (10000 U min^{-1}, 30 min), decanted and re-dispersed each time. Subsequently, the product was dried overnight (80 °C, 1 – 10 mbar), yielding a white powder (3.7861 g yield, BET = 1161 $m^2 g^{-1}$).

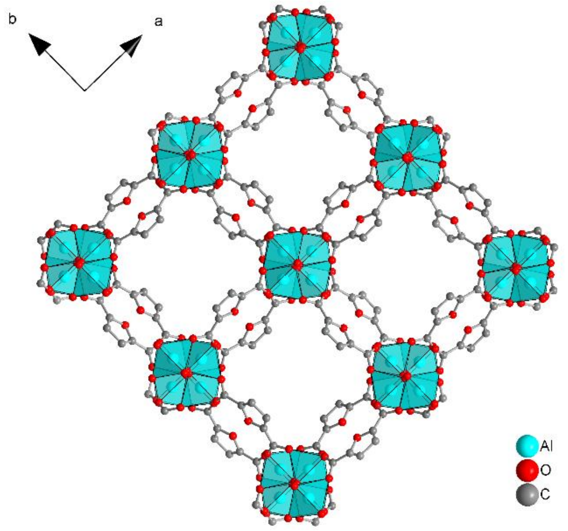

Figure S2 *The 3D framework structure of MIL-160 exhibits square shaped one-dimensional channels. Graphic produced by software Diamond[2] from cif-file for MIL-160 (CSD-Number: 1828694).[4]*

The synthesis of **DUT-4** was carried out in accordance with a report by Senkovska *et al.*[5] 2,6-naphthalenedicarboxylic acid (0.2603 g, 1.2 mmol, 1 eq) was dissolved in 30 ml *N,N*-dimethylformamide DMF. $Al(NO_3)_3 \cdot 9H_2O$ (0.5235 g, 1.4 mmol, 1.167 eq) was added and the mixture was filled in a 40 ml Teflon liner, placed in an autoclave, heated to 120 °C for 24 h and cooled to room temperature. After the product was separated by centrifugation, the sediment was washed with DMF (30 ml, each) for three times. Subsequently, the product was dried overnight (80 °C, 1 – 10 mbar), yielding a white powder (0.3505 g yield, BET = 1764 $m^2\,g^{-1}$).

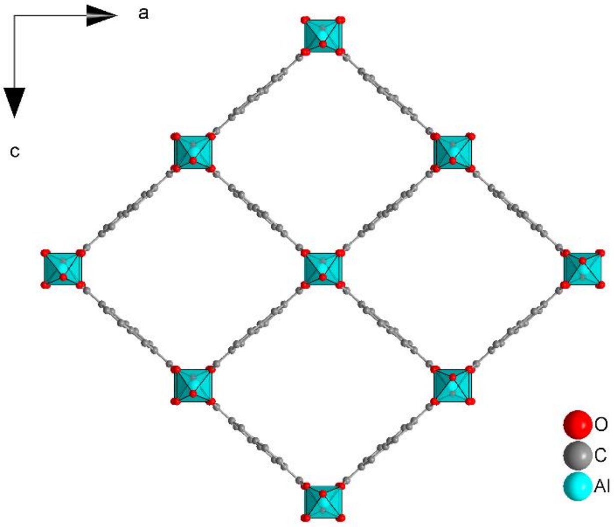

Figure S3 *The 3D framework structure of DUT-4 exhibits square shaped one-dimensional channels. Graphic produced by software Diamond[2] from cif-file for DUT-4 (CSD-Number: 691978).[5]*

The synthesis of **DUT-5** was carried out following a modified protocol of Gotthardt *et al.*[6] Biphenyl-4,4'-dicarboxylic acid (0.36058 g, 1.5 mmol, 1 eq) was dissolved in 40 mL DMF at 120 °C. A solution of $Al(NO_3)_3 \cdot 9H_2O$ (0.7620 g, 3 mmol, 2 eq) in 20 mL DMF was added over a period of 1.5 h. The reaction mixture was stirred for 24 h under reflux at 120 °C After the product was separated by centrifugation, the sediment was washed with DMF (60 ml, each) for three times. Subsequently, the product was dried overnight (80 °C, 1 – 10 mbar), yielding a white powder (0.6447 g yield, BET = 1323 $m^2\,g^{-1}$).

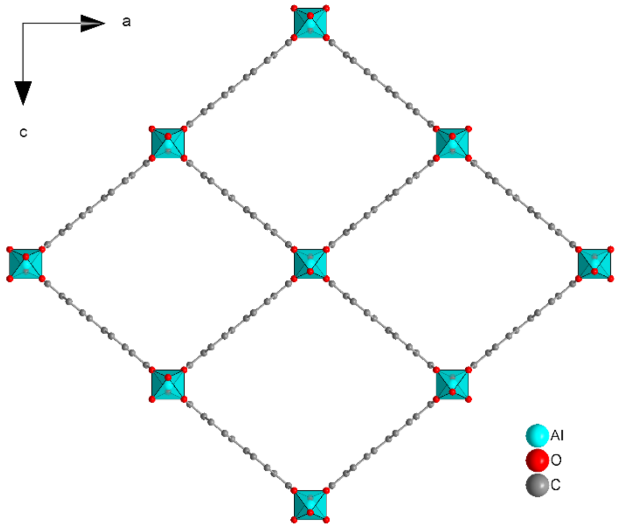

Figure S4 *The 3D framework structure of DUT-5 exhibits square shaped one-dimensional channels. Graphic produced by software Diamond*[2] *from cif-file for DUT-5 (CSD-Number: 691979).*[5]

The synthesis of **MIL-53-TDC** was carried out following a protocol of Tannert *et al*.[7] For the batch, $Al_2(SO_4)_3$ $18H_2O$ (3.79 g, 5.69 mmol, 1 eq), 2,5-thiophenedicarboxylic acid (0.86 g, 5 mmol, 1 eq) were dissolved in a mixture of *N,N*-dimethylformamide (8 mL) and deionized water (32 mL) and refluxed at 135 °C for 24 hours. The solid was centrifuged off (10000 U min^{-1}, 15 min) and the solvent was decanted off. The product was washed three times with 100 mL deionized water. Another fourth wash was carried out overnight. In contrast to the literature, the product was also refluxed in 100 mL ethanol for 24 hours at 80 °C. The end product was centrifuged off (10000 U min^{-1}, 15 min) and dried overnight in a vacuum oven (60 °C, 1-10 mbar), yielding a white powder (1.11 g yield, BET = 1015 $m^2\,g^{-1}$).

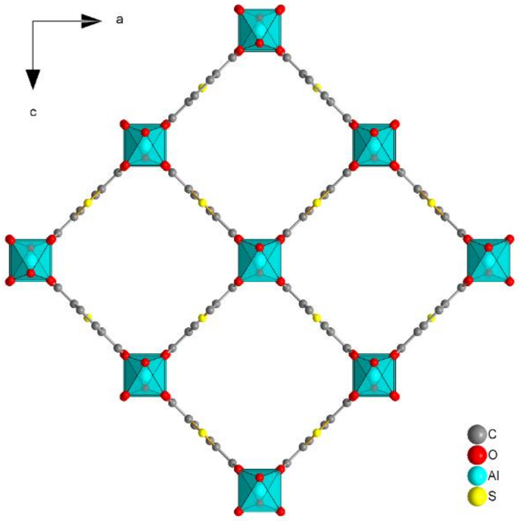

Figure S5 *The 3D framework structure exhibits of MIL-53-TDC square shaped one-dimensional channels. Graphic produced by software Diamond*[2] *from cif-file for MIL-53-TDC (The graphics were produced from a cif-filethat was generously provided by Serre and co-workers.).*

The synthesis of **MIL-53** was carried out following a protocol of Zi *et al*.[8] First, terephthalic acid (1.15 g, 6.92 mmol, 0.5 eq) was dissolved in deionized water (30 mL) and then the $Al(NO_3)_3{\cdot}9H_2O$ (5.21 g, 13.89 mmol, 1 eq) was added and dissolved with stirring. The reaction mixture was transferred to a steel autoclave with a Teflon inlay and placed in the oven at 200 °C for 24 h. The solid was centrifuged off (10000 U min^{-1}, 15 min). The washing and activation of the MOF was carried out according to Rallapalli et al.[9] The product was washed four times with deionized water (4x50 mL) until the filtrate reached a pH value of 7. The product was washed once at RT overnight in DMF (50 ml) with stirring and twice overnight under reflux (155 °C) in DMF (2 × 50 ml). After the product was centrifuged off, it was washed with methanol (50 mL), centrifuged again and placed in

the vacuum oven (60 °C, 1-10 mbar) for two hours. Finally, it was washed twice again with methanol (2 × 50 mL), centrifuged off and dried in a vacuum oven (60 °C, 1-10 mbar), yielding a white powder (0.72 g yield, BET = 1325 $m^2 g^{-1}$)

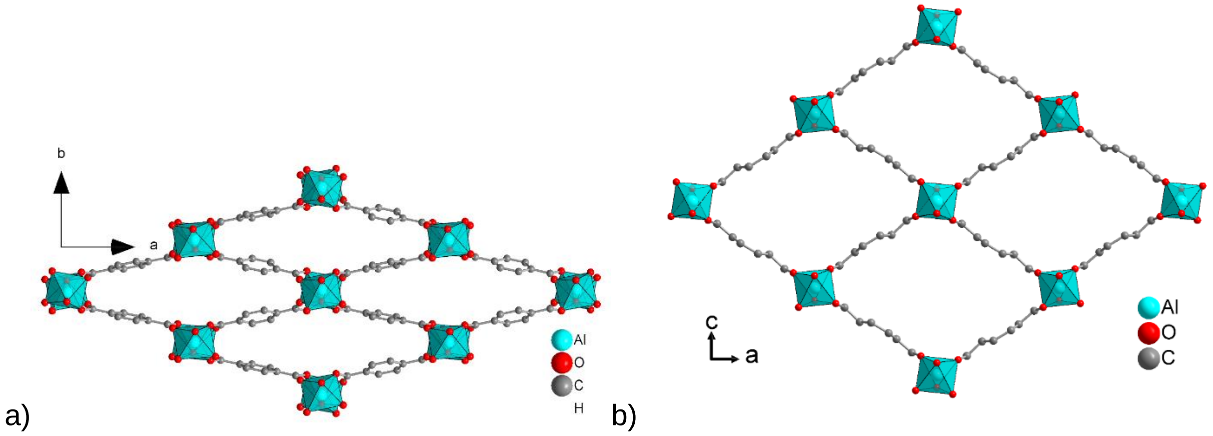

Figure S6 *The 3D framework structure of MIL-53 exhibits one-dimensional channels, with the dimensions depending on the breathing-effect. (a) Narrow-pore form, (b) Wide-pre form.Graphic produced by software Diamond[2] from cif-file for MIL-53 (a) CSD-Number: 1007172[10], (b) CSD-Number 220475, CSD-Refcode SABVOH[11]*).

Zr-MOFs

Zirconium-MOFs also show extremely high chemical stability, especially the UiO-series, which follows the isoreticular principle and allows the use of functionalized linkers. Thereby, the influence of linker functionalization on a gas or vapor uptake can be assessed. The zirconium MOF DUT-67 does not belongs to the UiO-series, but has the same thiophenedicarboxylate (TDC) linker as the aluminum-MOF MIL-53-TDC.

The synthesis of **UiO-66** was carried out following a modified protocol of Aguilera-Sigalat *et al.*[12] $ZrCl_4$ (0.2027 g, 0.87 mmol, 1 eq) and terephthalic acid (0.1145 g, 0.87 mmol, 1 eq) were dissolved in DMF (50 ml) with acetic acid (1.5 ml) and the mixture was sonicated for 10 min. The mixture was filled in a 100 ml Schott glas, heated to 120 °C for 24 h and cooled to room temperature. After the product was separated by centrifugation, the sediment was washed with DMF (50 ml, each) for three times and with ethanol (50 ml, each) for three times. Subsequently, the product was dried overnight (80 °C, 1 – 10 mbar), yielding a white powder (0.1007 g yield, BET = 1178 $m^2 g^{-1}$).

The synthesis of **UiO-66-NH$_2$** was carried out in accordance with a report by Aguilera-Sigalat *et al.*[12] $ZrCl_4$ (0.2023 g, 0.87 mmol, 1 eq) and 2-aminoterephthalic acid (0.1574 g, 0.87 mmol, 1 eq) were dissolved in DMF (50 ml) with acetic acid (1.5 ml) and the mixture was sonicated for 10 min. The mixture was filled in a 100 ml Schott glas, heated to 120 °C for 24 h and cooled to room temperature. After the product was separated by centrifugation, the sediment was washed with DMF (50 ml, each) for three times and with ethanol (50 ml, each) for three times. Subsequently, the product was dried overnight (80 °C, 1 – 10 mbar), yielding a yellow powder (0.2121 g yield, BET = 1127 $m^2 g^{-1}$).

The synthesis of **UiO-66(F)$_4$** was carried out in accordance with a report by Hu *et al.*[13] $ZrCl_4$ (1.653 g, 5 mmol, 1 eq) and tetrafluoroterephthalic acid (1.1907 g, 5 mmol, 1 eq) were dissolved in DMF (30 ml) with acetic acid (20 ml) and the mixture was heated to reflux for 24 h and cooled to room temperature. After the product was separated by centrifugation, the sediment was washed with methanol and dichloromethane for three days. Subsequently, the product was dried overnight (80 °C, 1 – 10 mbar), yielding a white powder (1.1639 g yield, BET = 346 $m^2 g^{-1}$).

The synthesis of **UiO-67** was carried out in accordance with a report by Katz *et al.*[14] $ZrCl_4$ (0.2517 g, 1.08 mmol, 1 eq) was dissolved in DMF (20 ml) with HCl (2 ml) and the mixture was sonicated for 10 min. Then biphenyl-4,4'-dicarboxylic acid (0.3682 g, 1.52 mmol, 1.41 eq) and DMF (40 ml) were added and sonicated for another 20 min. The mixture was filled in a 100 ml Schott glas, heated to 80 °C for 24 h and cooled to room temperature. After the product was separated by centrifugation, the sediment was washed with DMF (60 ml, each) for three times and with ethanol (60 ml, each) for

three times. Subsequently, the product was dried overnight (80 °C, 1 – 10 mbar), yielding a white powder (0.3596 g yield, BET = 2317 $m^2 g^{-1}$).

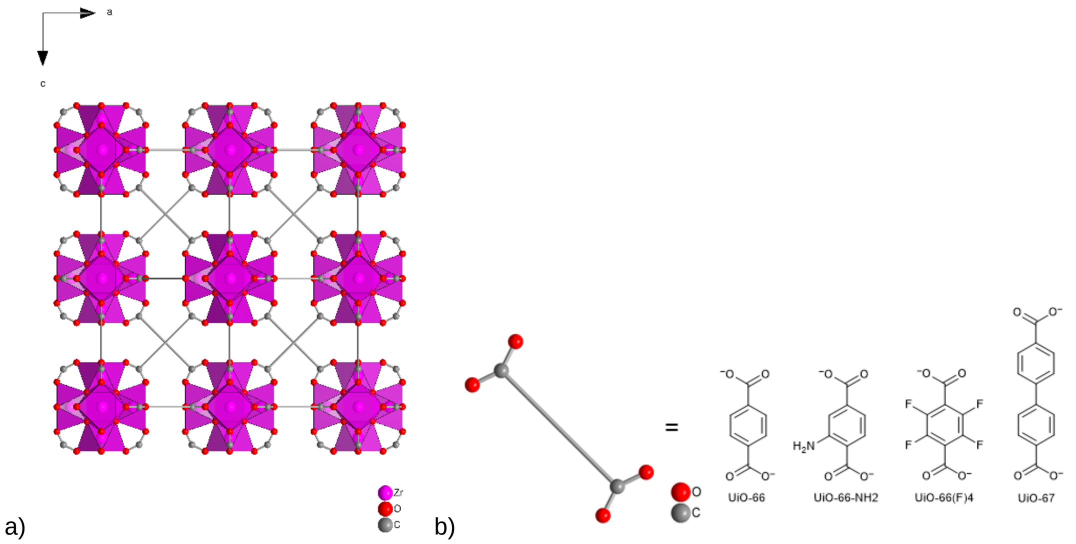

Figure S7 *The 3D framework structure simulated for UiO-type materials (a) and their linker (b). Graphic produced by software Diamond[2] from cif-file for UiO-66 (CSD-Number: 733458).[15]*

The synthesis of **DUT-67** was carried out in accordance with a report by Drache *et al.*[16] $ZrCl_4$ (1.3822 g, 6 mmol, 1.5 eq) was dissolved in DMF (75 ml) and *N*-methyl-2-pyrrolidione (NMP) (75 ml) and the mixture was sonicated for 10 min. Then 2,5-thiophendicarboxylic acid (0.6651 g, 4 mmol, 1 eq) was added and sonicated for another 5 min and finally formic acid (26 ml) was added. The mixture was filled in a 250 ml Schott glas, heated to 120 °C for 48 h and cooled to room temperature. After the product was separated by centrifugation, the sediment was washed with DMF (120 ml, each) for three times and with (120 ml, each) for three times. Subsequently, the product was dried overnight (80 °C, 1 – 10 mbar), yielding a yellow powder (0.9432 g yield, BET = 1178 $m^2 g^{-1}$).

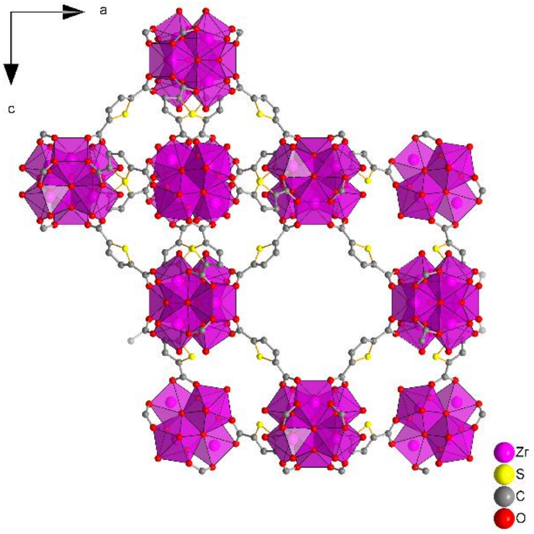

Figure S8 *The 3D framework structure simulated for DUT-67 with both pore types. Graphic produced by software Diamond[2] from cif-file for DUT-67 (CSD-Number: 921644).[17]*

Ti-MOFs

NH_2-MIL-125 has a higher chemical stability than unfunctionalized MIL-125 and the aminoterephthalate linker is the same as in UiO-66-NH_2. MIL-125 has the same terephthalate or benzene-1,4-dicarboxylate linker, as MIL-53, UiO-66 and MIL-101(Cr).

The synthesis of **NH_2-MIL-125** was carried out following a modified protocol of Sohail *et al.*[18] 2-aminoterphthalic acid (1.0856 g, 5.99 mmol, 4.02 eq) was dissolved in DMF (3.5 ml) and methanol (3.5 ml) *via* sonication for 5 min and stirred for 20 h. $Ti[OCH(CH_3)_2]_4$ (0.44 ml, 0.4224 g, 1.49 mmol, 1 eq) was added to the solution and transferred to aTeflon liner, placed in an autoclave, heated to 150 °C for 16 h and cooled to room temperature. After the product was separated by centrifugation, the sediment was washed with DMF (30 ml, each) for three times and with ethanol (30 ml, each) for three times. Subsequently, the product was dried overnight (80 °C, 1 – 10 mbar), yielding a yellow powder (0.3503 g yield, BET = 1570 $m^2 g^{-1}$).

The synthesis of **MIL-125** was carried out according to the procedure in Santaclara *et al.*[19] For the batch, $Ti[OCH(CH_3)_2]_4$ (4.2 mL, 14.19 mmol, 1 eq) and terephthalic acid (3.53 g, 21.25 mmol, 1.5 eq) were reacted in DMF (56 mL) and methanol (14 mL). In a first step, the terephthalic acid was dissolved in DMF under reflux (105 °C, 1 h). Then methanol was added. After a further hour under reflux, the titanium isopropoxide was added and the mixture was stirred under reflux (100 °C, 72 h). After the mixture had cooled to RT, the solid was centrifuged off (10000 U min^{-1}, 15 min). For washing, the product was refluxed in 60 mL DMF (155 °C, 24 h) and then in methanol (100 °C, 24 h). The product was dried overnight in a vacuum oven (60 °C., 1-10 mbar), yielding a white powder (3.72 g yield, BET = 1425 $m^2 g^{-1}$).

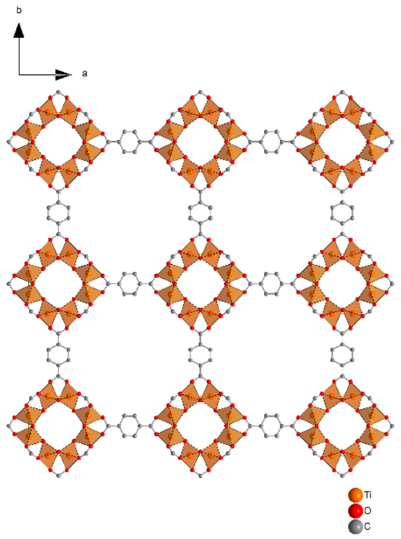

Figure S9 *The 3D framework structure simulated for MIL-125, structure is simular to NH_2-MIL-125. Graphic produced by software Diamond[2] from cif-file for MIL-125 (CSD-Number: 751157).[20]*

Cr-MOF

MIL-101(Cr) was chosen because of the high surface area and pore volume together with unsurpassed hydrothermal and chemical stability. MIL-101(Cr) often gives a maximum absorption capacity through its large pores and is a benchmark material, which is often noted in the literature.

The synthesis of **MIL-101(Cr)** was carried out in accordance with a report by Zhao *et al.*[21] $Cr(NO_3)_3 \cdot 9H_2O$ (0.4038 g, 1 mmol, 1 eq) and terephthalic acid (0.1645 g, 1 mmol, 1 eq) were solved in water (5 ml) and nitric acid (69.7 µl, 1 mmol). The mixture was transferred to a Teflon liner, placed in an autoclave, heated to 220 °C for 8 h and cooled to room temperature. After the product was separated by centrifugation, the sediment was washed with DMF (30 ml) and with water (30 ml) and ethanol (30 ml). Subsequently, the product was dried overnight (80 °C, 1 – 10 mbar), yielding a green powder (0.2447 g yield, BET = 2002 $m^2 g^{-1}$).

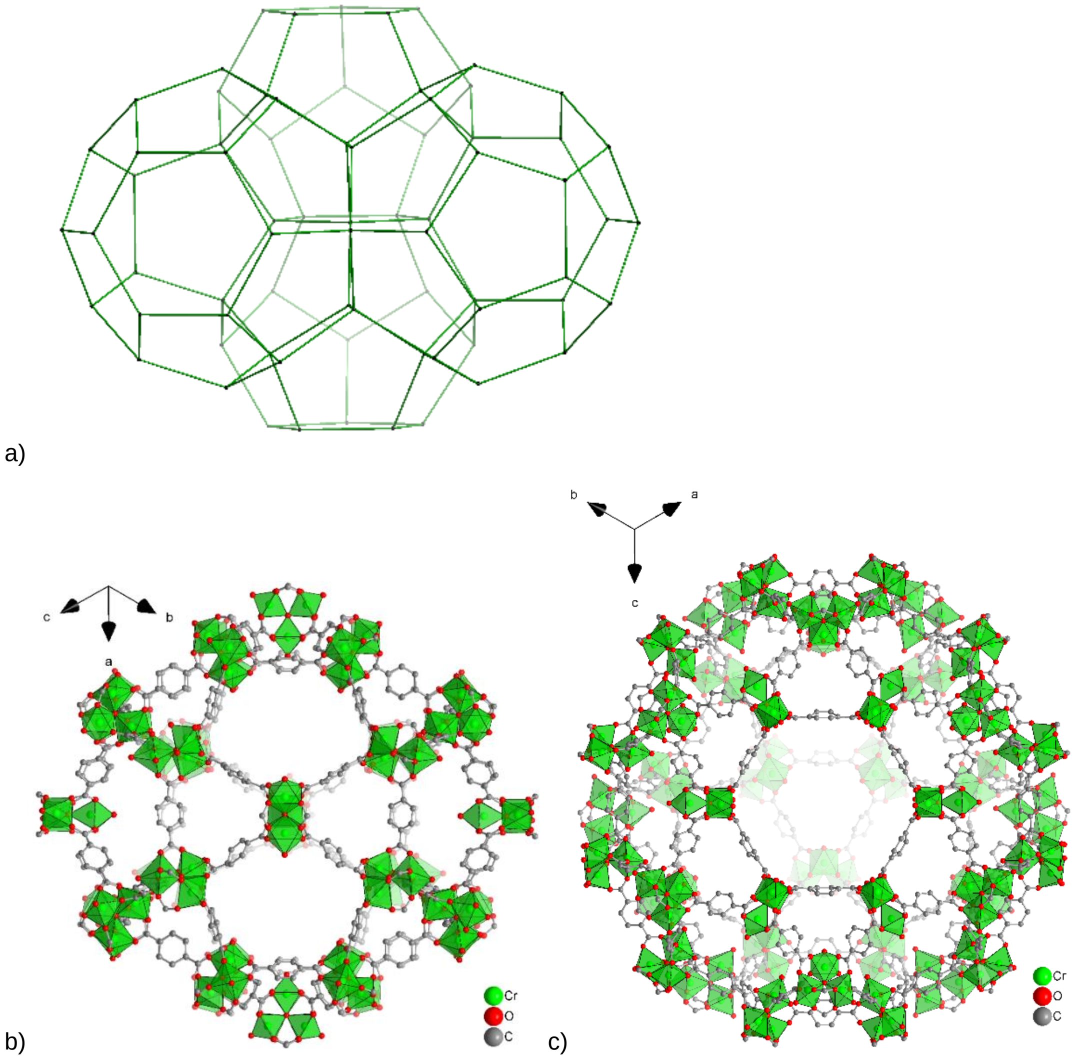

Figure S10 *(a) The 3D zeotypic framework structure for MIL-101(Cr) with (b) small (pentagonal windows only) and (c) large cages (pentagonal and hexagonal windows). Graphic produced by software Diamond*[2] *from cif-file for MIL-101(Cr) (CSD-Number: 605510).*[22]

ZIFs

ZIF-8 is a hydrophobic MOF with the 2-methylimidazolate linker, which is well investigated and easy to synthesize and has a high thermal and chemical stability, which makes it interesting for industrial applications. ZIF-11 and ZIF-7 have the same benzimidazolate linker but different topologies and therefore different pore sizes and surface area, with ZIF-11 being a **rho** and ZIF-7 a **sod** network.

The synthesis of **ZIF-8** was carried out in accordance with a report by Kida *et al.*[23] $Zn(NO_3)_3 \cdot 6H_2O$ (0.7520, 2.5 mmol, 1 eq) was dissolved in water (10 ml) and 2-methylimidazole (12.3033 g, 150 mmol, 60 eq) was dissolved in water (90 ml). The mixture was stirred for 24 h at room temperature. After the product was separated by centrifugation, the sediment was washed with ethanol (100 ml, each) for three times. Subsequently, the product was dried overnight (80 °C, 1 – 10 mbar), yielding a white powder (0.5198 g yield, BET = 1615 $m^2\,g^{-1}$).

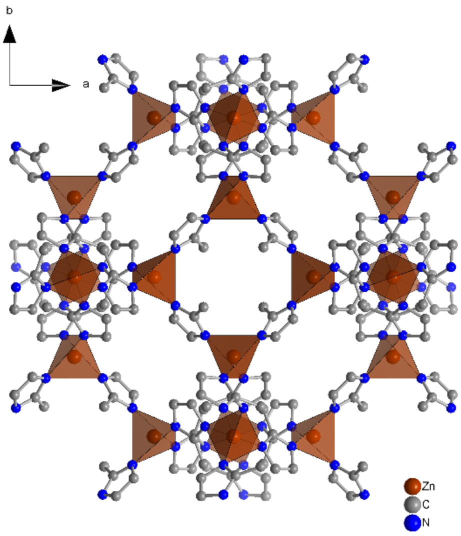

Figure S11 *The 3D framework structure simulated for ZIF-8. Graphic produced by software Diamond*[2] *from cif-file for ZIF-8 (CSD-Number: 864309).*[24]

The synthesis of **ZIF-11** was carried out in accordance with a report by He *et al.*[25] Benzimidazole (0.4723 g, 4 mmol, 2 eq) was dissolved in methanol (24.33 ml), and then toluene (21.18 ml) and ammonia hydroxide (0.3 ml) were added under stirring at room temperature. $Zn(CH_3COO)_2 \cdot 2H_2O$ (0.4394 g, 2 mmol, 1 eq) were added to this mixture and stirred for 3 h at room temperature. After the product was separated by centrifugation, the sediment was washed with methanol (60 ml, each) for three times. Subsequently, the product was dried overnight (80 °C, 1 – 10 mbar), yielding a white powder (0.5609 g yield, BET = 448 $m^2 g^{-1}$).

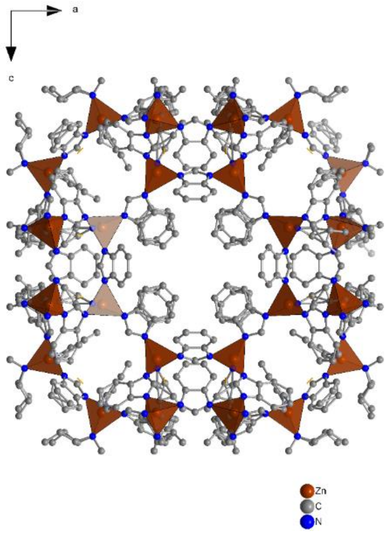

Figure S12 *The 3D building block structure simulated for ZIF-11. Graphic produced by software Diamond*[2] *from cif-file for ZIF-11 (CSD-Number: 602545).*[26]

The synthesis of **ZIF-7** was carried out following a modified protocol of Kang *et al.*[27] and Li *et al.*[28] DMF (20 ml) was added to a solid mixture of $Zn(NO_3)_2 \cdot 6H_2O$ (0.3813 g, 1.27 mmol, 1 eq) and benzimidazole (0.2002 g, 1.69 mmol, 1.33 eq) and stirred till a clear solution was made. The mixture was transferred to a Teflon liner, placed in an autoclave, heated to 160 °C for 24 h and cooled to room temperature. After the product was separated by centrifugation, the sediment was washed with methanol (30 ml, each) for six times. Subsequently, the product was dried overnight (80 °C, 1 – 10 mbar), yielding a white powder (0.1712 g yield, BET = 275 $m^2 g^{-1}$).

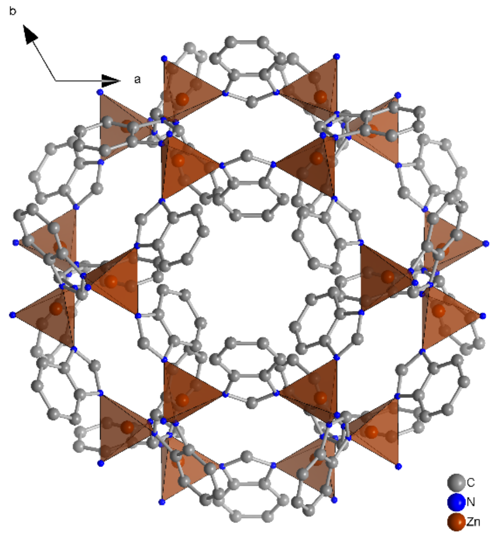

Figure S13 *The 3D framework structure simulated for ZIF-7. Graphic produced by software Diamond*[2] *from cif-file for ZIF-7 (CSD-Number:602541).*[26]

MOF parameters

Table S2 *MOF parameters (BET-surface area, (total) pore volume (DFT), micropore volume, pore window).*[a]

**MOF**	**BET-Surface area [m^2 g^{-1}]**	**Pore volume (DFT) [cm^3 g^{-1}]** [c]	**Micropore volume [cm^3 g^{-1}]** [d]		**Pore window [Å^2]**	**Literature (with respect to Pore Window)**
Basolite® A520 (Aluminumfumarate)	988	0.615	0.33		5.7 x 6.0	Alvarez *et al.*[1]
Aluminumfumarate	1035	0.517	0.354		5.7 x 6.0	Alvarez *et al.*[1]
MIL-160	1161	0.404	0.426		5.5 x 5.5	Wahiduzzaman *et al.*[4]
DUT-4	1764	1.077	0.92		8.5 x 8.5	Senkovska *et al.*[5]
DUT-5	1323	0.68	0.409		11.1 x 11.1	Senkovska *et al.*[5]
MIL-53-TDC	1015	0.466	0.33		8 x 8.2	Tschense *et al.*[29]
MIL-53(Al)	1325	0.486	0.462		8.5 x 8.5	Loiseau *et al.*[11]
UiO-66	1178	0.503	0.414		5.9 x 5.9	Lin *et al.*[30]
UiO-66-NH_2	1127	0.45	0.384		7.0 x 7.0	Chen *et al.*[31]
UiO-66$(F)_4$	346	0.35	0.091		2.4 x 4.6	Reinsch *et al.*[32]
UiO-67	2317	0.962	0.858		8.3 x .8.3	Lin *et al.*[30]
DUT-67	1178	0.443	0.403		6.7 x 6.7	Lin *et al.*[30]
NH_2-MIL-125	1570	0.601	0.559		5.9 x 5.9	Kim *et al.*[33]
MIL-125	1425	0.565	0.493		6.1 x 6.1	Kim *et al.*[33]
MIL-101(Cr)	2002	1.024	0.604		12 x 12	Ferey *et al.*[22]
ZIF-8	1615	0.673	0.632		3.4 x 3.4	Novakovic *et al.*[34]
ZIF-11	491	0.354	0.014		3.0 x 3.0	He *et al.*[25]
ZIF-7	275 (from CO_2)[b]	0.1405[e]	0		2.9 x 2.9	He *et al.*[25]

[a] BET surface area, pore volume and micropore volume were derived from our N_2 sorption isotherm measurements at 77 K of the synthesized samples.

[b] ZIF-7 does not show an N_2 adsorption at 77 K, hence the BET surface area was derived from CO_2 adsorption (3.03 mmol CO_2) at 273 K.

[c] Total pore volumes were calculated from the N_2-sorption isotherm at $p\,p_0^{-1} = 0.90$ for pore sizes ≤ 20 nm. NLDFT calculations were done with the native *NovaWin 11.03* software using the ‘N_2 at 77 K on carbon, slit pore, NLDFT equilibrium’ model. Thickness model for calculation of micropore volumes and micropore areas was set to ‘De Boer’.

[d] Micropore volumes were calculated from the N_2 adsorption isotherm at p p_0^{-1} = 0.2-0.3 for pores with d ≤ 2 nm (20 Å). Micropore volumes (V_{micro}) were calculated by the t-plot method ('De Boer' model).

[e] Total pore volume was calculated from the CO_2 adsorption isotherm at p p_0^{-1}.

Table S3 *MOF parameters exp. pore volume (DFT), calculated void volume and specific void volume, and percent of pore filling with the C_6-VOCs.* [a]

MOF	Exp. pore volume (DFT) [cm^3 g^{-1}] [b]	Calc. void volume, $V_{unit\ cell}$ [$Å^3$] [c]	calc. Specific void volume [cm^3 g^{-1}] [d]	Pore filling C_6H_6 [%] [i]	Pore filling C_6H_{12} [%] [i]	Pore filling C_6H_{14} [%] [i]	Z, $M_{asym\ unit}$ [--], [g mol^{-1}]
Basolite® A520 (commercial Alfum)	0.615	481	0.46	-	-	-	Z = 4,<br>M = 158
Aluminumfumarate, Alfum	0.517	481	0.46	79<br>89	71<br>80	91<br>102	Z = 4,<br>M = 158
MIL-160	0.404	2217	0.42	121<br>116	121<br>116	151<br>145	Z = 16, $C_6H_3O_6Al$,<br>M = 198
DUT-4	1.077	1320	0.77	110<br>153	84<br>117	96<br>134	Z = 4, $C_{12}H_7O_5Al$,<br>M = 258
DUT-5	0.68	1917	1.02	138<br>92	141<br>94	224<br>149	Z = 4, $C_{14}H_9O_5Al$,<br>M = 284
MIL-53-TDC	0.466	746	0.52	73<br>65	62<br>56	82<br>73	Z = 4, C6H3O5S-Al,<br>M = 214
MIL-53(Al)	0.486	678	0.49	195<br>194	191<br>190	283<br>282	Z = 4, $C_8H_5O_5Al$,<br>M = 208
UiO-66	0.503	4807	0.43	95<br>112	169<br>198	199<br>233	Z = 4<br>$Zr_6O_4(OH)_4(C_8H_4O4)_6$ =<br>$C_{48}H_{28}O_{32}Z_{r6}$,<br>M = 1664
UiO-66-NH_2 [e]	0.45	3671	0.32	82<br>116	87<br>122	184<br>259	Z = 4<br>$Zr_6O_4(OH)_4(C_8H_5NO_4)_6$ =<br>$C_{48}H_{34}N_6O_{32}Zr_6$,<br>M = 1754
UiO-66(F)$_4$ [f]	0.35	3671	0.26	117<br>158	100<br>135	91<br>123	--
UiO-67	0.962	12442	0.83	124<br>143	119<br>137	83<br>96	Z = 4,<br>$Zr_6O_4(OH)_4(C_{14}H_8O_4)_6$ =<br>$C_{96}H_{52}O_{32}Zr_6$,<br>M = 2265
DUT-67	0.443	39628	0.66	124<br>83	142<br>96	135<br>91	Z = 24,<br>$Zr_6O_4(OH)_4(OH)_4(H_2O)_4$-<br>$(C_6H_2O_4S)_4$ =<br>$C_{24}H_{24}O_{32}S_4Zr_6$,<br>M = 1500
NH_2-MIL-125 [g]	0.601	4157	0.80	50<br>38	60<br>45	82<br>61	--
MIL-125	0.565	4157	0.80	141<br>100	142<br>100	154<br>109	Z = 2, $Ti_8(OH)_{12}$-$(C_8H_4O_4)_6$<br>$C_{48}H_{36}O_{36}Ti_8$, M = 1572
MIL-101(Cr)	1.024	n.a.	n.a.	n.a.	n.a	n.a.	too large for calculation
ZIF-8	0.673	2329	0.51	85<br>112	51<br>67	89<br>118	Z = 4, $C_{24}H_{30}N_{12}Zn_3$<br>M = 683
ZIF-11 [h]	0.354	10391	0.43	90<br>74	136<br>112	102<br>84	Z = 12, $C_{56}H_{40}N_{16}Zn_4$,<br>M = 1199
ZIF-7	0.1405	1819	0.20	64<br>45	57<br>40	107<br>75	Z = 18, $C_{14}H_{10}N_4Zn$,<br>M = 308

[a] BET surface area, pore volume and micropore volume were derived from our N_2 sorption isotherm measurements at 77 K of the synthesized samples.

[b] Total pore volumes were calculated from the N_2-sorption isotherm at p/p_0 = 0.90 for pore sizes ≤ 20 nm. NLDFT calculations were done with the native *NovaWin 11.03* software using the 'N_2 at 77 K on carbon, slit pore, NLDFT equilibrium' model. Thickness model for calculation of micropore volumes and micropore areas was set to 'De Boer'.

[c] Void volume in Å^3 calculated by a 'Void' calculation with Mercury[35,36] using a probe radius of 1.5 Å for the contact surface. Any solvent of crystallization was removed from the cif file. Not given H atoms were added. If possible, a disorder was removed (e.g. in ZIF-11).

[d] Specific pore volume in cm^3 g^{-1} calculated according to ($V_{unit\,cell} \times N_A$)/($Z \times M_{asym\,unit}$); N_A = Avogadro's constant, 6.022 · 10^{23} mol^{-1}, Z = number of asymmetric formula units, $M_{asym\,unit}$ = molecular weight of asymmetric formula unit (in g mol^{-1}). As a help to reproduce the specific number the values for *Z* and $M_{asym\,unit}$ from the X-ray structures are given in the last column. Note that the given values were obtained from Checkcif (checkcif.iucr.org) with the deposited or from a drawing of the unit cell with the program Diamond.[2] In case no H atoms were included in the cif, these were added to give $M_{asym\,unit}$. The Z and related molecular weight in the cif could differ due to crystal solvent or due to non-corrected unit cell content from the structure solution and refinement.

[e] In UiO-66-NH_2 there is an N atom at each C atom of the benzene ring, induced by the crystallographic symmetry. This crystallographically-induced disorder cannot be removed. Thus, the actual available void volume of UiO-66-NH_2 will be somewhat larger and lie in-between the given calculated value and the value for UiO-66.

[f] No X-ray structure available, but isostructural to UiO-66 and UiO-66-NH_2 and UiO-66-$(F)_4$ should be close in the calculated void volume of UiO-66-NH_2 due to the crystallographically-induced N atom (in place of F) at each C atom.

[g] No X-ray structure available, but isostructural to MIL-125 with values taken from there.

[h] Disorder (part A) in the linker was removed.

[i] Pore filling calculated from vapor uptake in [mg g^{-1}] (293 K) at p p_0^{-1} = 0.9 divided by the density ρ of the liquid (at 293 K) to approximate the volume of liquid benzene, cyclohexane or *n*-hexane adsorbed in the MOFs at p p_0^{-1} = 0.9 ($\rho_{benzene}$ = 0.876 g cm^{-3}; $\rho_{cyclohexane}$ = 0.779 g cm^{-3}; ρ_{hexane} = 0.655 g cm^{-3}) (the respective values are summarized in Table S3a-c on the next page) and further divided by the experimental pore volume (lower line) and calculated specific void volume (upper line) and multiplied by 100%.

Interpretation to Table S3:

There is in most cases a reasonable match between the experimental pore volume and the calculated specific void volume. Substantial discrepancies are only seen for DUT-4 and DUT-5 which are explained by the low surface area of DUT-5, due to the synthesis.

In cases where the calculated specific void volume is larger than the experimental pore volume a larger probe radius would give a smaller void volume. The largest possible probe radius in Mercury is 2.0 Å (cf. Table S3), the standard probe radius is normally 1.2 Å. A larger calculated specific void volume than experimental pore volume can signal either incomplete activation or inaccessible pore regions for the N_2 adsorbate (kinetic diameter 3.64 Å).

In cases where the calculated specific void volume is smaller than the experimental pore volume, as for UiO-66, UiO-66-NH_2 and UiO-67, this can be traced to the known defect formation in UiOs.[37] For the larger experimental pore volume in ZIF-8 the known gate effect can be operating which does not work in the static theoretical void volume calculation.[34]

***Table S3a** Experimental benzene vapor uptake in [mg g–1] and [cm3 g–1].*

MOF	benzene uptake p p_0^{-1} = 0.9 (293 K)	
	[mg g^{-1}]	cm^3 liq. benzene/gMOF [a]
Alfum (Basolite® A520)	551	0.63
Aluminumfumarate	358	0.41
MIL-160	429	0.49
DUT-4	1031	1.18
DUT-5	820	0.94
MIL-53-TDC	298	0.34
MIL-53(Al)	830	0.95
UiO-66	419	0.48
UiO-66-NH_2	323	0.37
UiO-66(F)$_4$	359	0.41
UiO-67	1043	1.19
DUT-67	483	0.55
NH_2-MIL-125	262	0.30
MIL-125	701	0.80
MIL-101(Cr)	776	0.89
ZIF-8	498	0.57
ZIF-11	277	0.32
ZIF-7	83	0.09

[a] benzene uptake / $\rho_{benzene}$ = cm^3 liq. benzene/gMOF with density $\rho_{benzene}$ = 0.876 g cm^{-3} of liquid C_6H_6 at 293 K to approximate the volume of benzene adsorbed in the MOFs at p p_0^{-1} = 0.9 and 293 K

***Table S3b** Experimental cyclohexane vapor uptake in [mg g–1] and [cm3 g–1].*

MOF	cyclohexane uptake p p_0^{-1} = 0.9 (293 K)	
	[mg g^{-1}]	cm^3 liq. cyclo-hexane/gMOF [a]
Alfum (Basolite® A520)	485	0.62
Aluminumfumarate	291	0.37
MIL-160	385	0.49
DUT-4	698	0.90
DUT-5	750	0.96
MIL-53-TDC	227	0.29
MIL-53(Al)	726	0.93
UiO-66	666	0.85
UiO-66-NH_2	307	0.39
UiO-66(F)$_4$	276	0.35
UiO-67	890	1.14
DUT-67	487	0.63
NH_2-MIL-125	279	0.36
MIL-125	627	0.80
MIL-101(Cr)	1007	1.29
ZIF-8	262	0.34
ZIF-11	376	0.48
ZIF-7	59	0.08

[a] cyclohexane uptake / $\rho_{cyclohexane}$ = cm^3 liq. cyclohexane/gMOF with density $\rho_{cyclohexane}$ = 0.779 g cm^{-3} of liquid cyclohexane at 293 K to approximate the volume of cyclohexane adsorbed in the MOFs at p p_0^{-1} = 0.9 and 293 K

***Table S3c** Experimental n-hexane vapor uptake in [mg g–1] and [cm3 g–1].*

MOF	*n*-Hexane uptake p p_0^{-1} = 0.9 (293 K)	
	[mg g^{-1}]	cm^3 liq. hexane/gMOF [a]
Alfum (Basolite® A520)	438	0.67
Aluminiumfumarate	310	0.47
MIL-160	397	0.61
DUT-4	677	1.03
DUT-5	997	1.52
MIL-53-TDC	246	0.38
MIL-53(Al)	901	1.38
UiO-66	654	1.00
UiO-66-NH_2	543	0.83
UiO-66(F)$_4$	207	0.32
UiO-67	527	0.80
DUT-67	395	0.60
NH_2-MIL-125	323	0.49
MIL-125	569	0.87
MIL-101(Cr)	663	1.01
ZIF-8	395	0.60
ZIF-11	236	0.36
ZIF-7	96	0.15

[a] hexane uptake / ρ_{hexane} = cm^3 liq. hexane/gMOF with density ρ_{hexane} = 0.655 g cm^{-3} of liquid cyclohexane at 293 K to approximate the volume of hexane adsorbed in the MOFs at p p_0^{-1} = 0.9 and 293 K

S3 Powder X-ray diffraction (PXRD) measurements

Al-MOFs

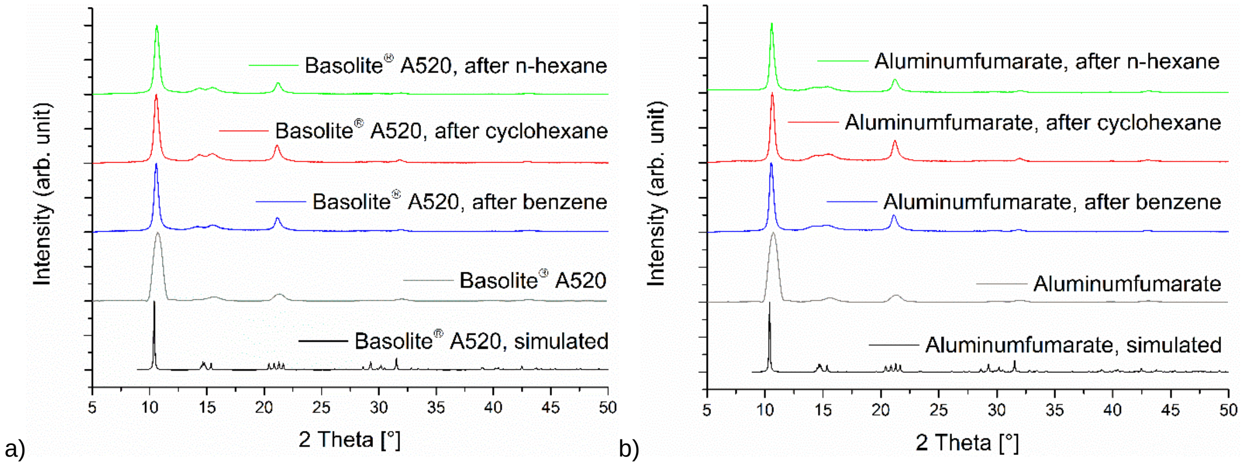

Figure S14 *(a) PXRDs of Basolite® A520 after VOC-sorption, compared with MOF before sorption and with simulated pattern (CSD-Number: 1051975).[1] (b) PXRDs of Aluminumfumarate after VOC-sorption, compared with MOF before sorption and with simulated pattern (CSD-Number: 1051975).[1]*

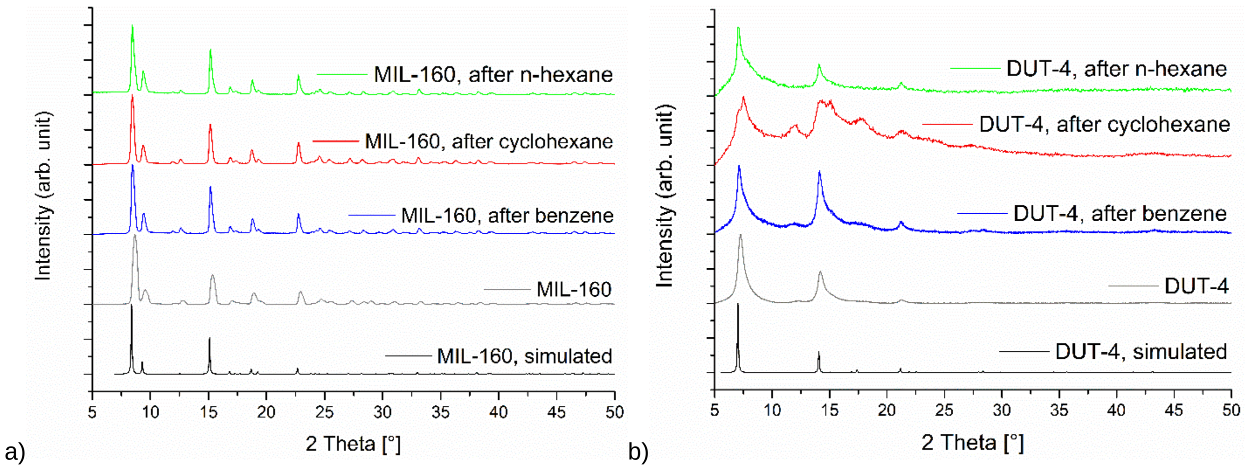

Figure S15 *(a) PXRDs of MIL-160 after VOC-sorption, compared with MOF before sorption and with simulated pattern (CSD-Number: 1828696).[4] (b) PXRDs of DUT-4 after VOC-sorption, compared with MOF before sorption and with simulated pattern (CSD-Number: 691978).[5]*

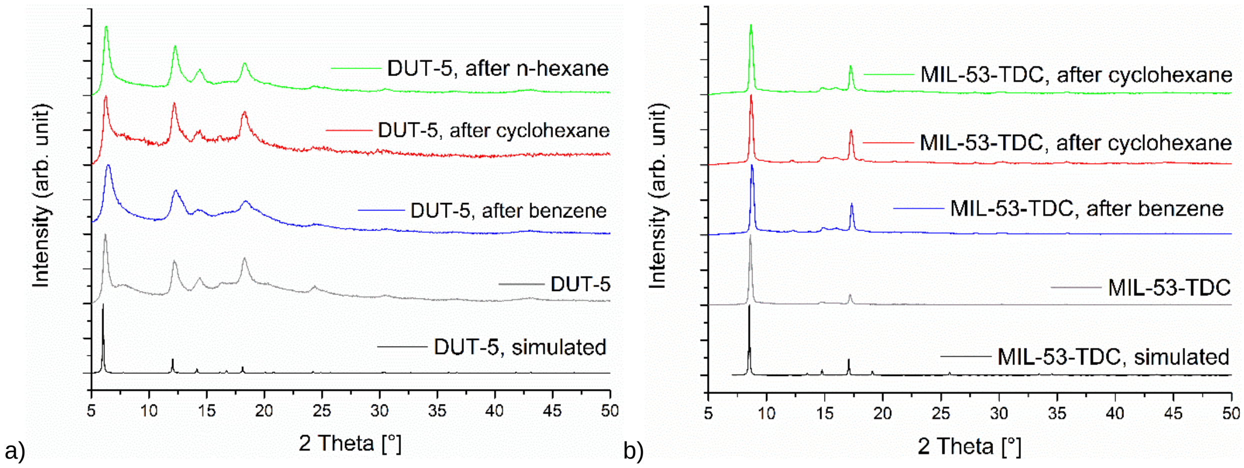

Figure S16 *(a) PXRDs of DUT-5 after VOC-sorption, compared with MOF before sorption and with simulated pattern (CSD-Number: 691979).[5] (b) PXRDs of MIL-53-TDC after VOC-sorption, compared with MOF before sorption and with simulated pattern (CSD-Number: Serre and Co-Workers).*

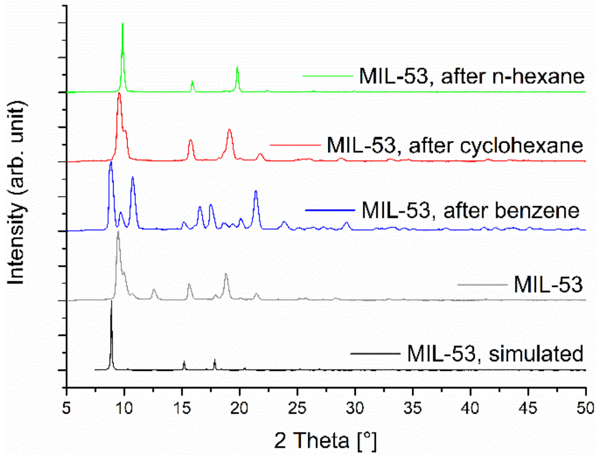

Figure S17 *PXRDs of MIL-53 after VOC-sorption, compared with MOF before sorption and with simulated pattern (CSD-Number: 1007172).*[10]

Zr-MOFs

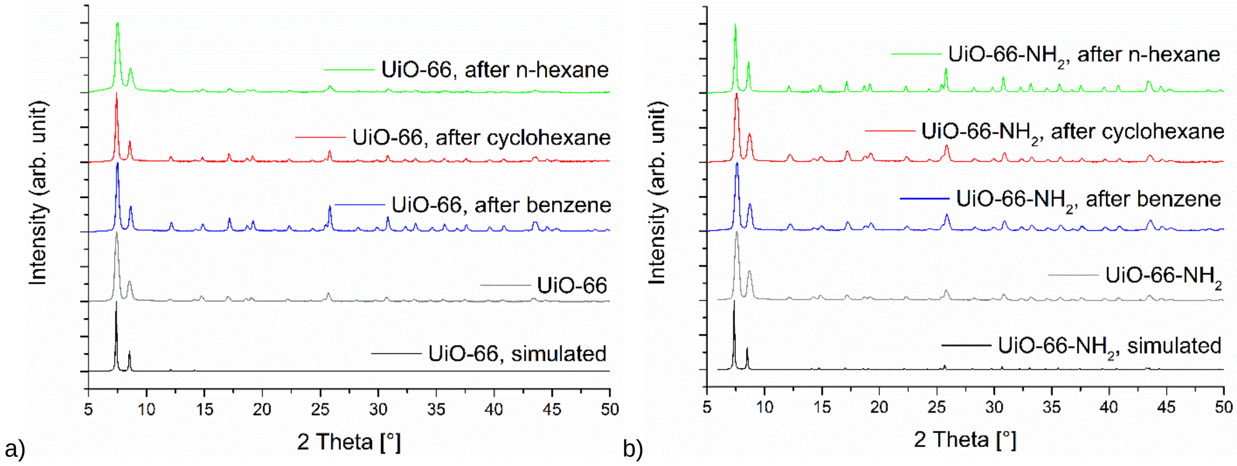

Figure S18 *(a) PXRDs of UiO-66 after VOC-sorption, compared with MOF before sorption and with simulated pattern (CSD-Number: 733458).*[15] *(b) PXRDs of UiO-66-NH$_2$ after VOC-sorption, compared with MOF before sorption and with simulated pattern (CSD-Number: 1405751).*[38]

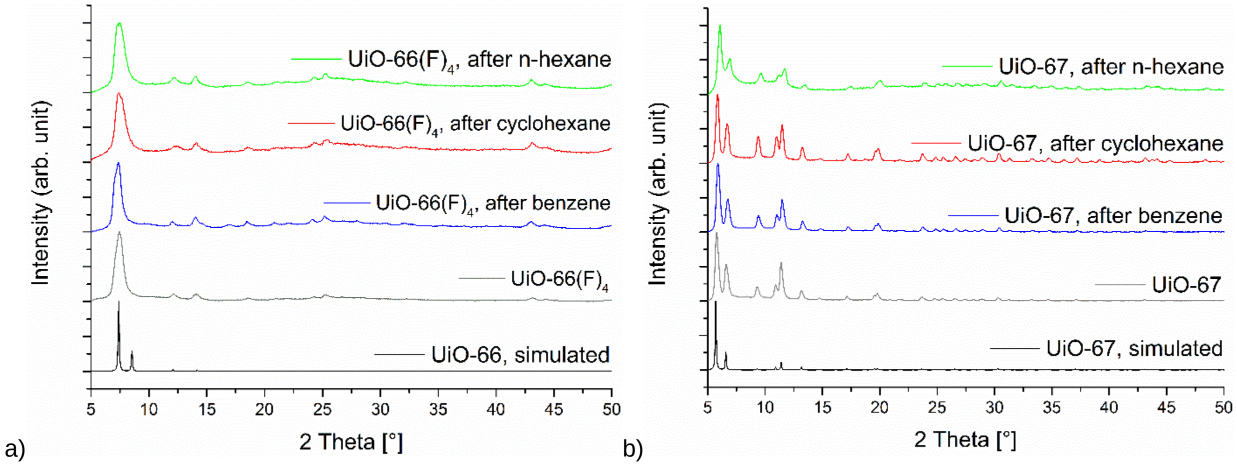

Figure S19 *(a) PXRDs of UiO-66(F)$_4$ after VOC-sorption, compared with MOF before sorption and with simulated pattern (CSD-Number: 733458).*[15] *(b) PXRDs of UiO-67 after VOC-sorption, compared with MOF before sorption and with simulated pattern (CSD-Number: 1018032).*[39]

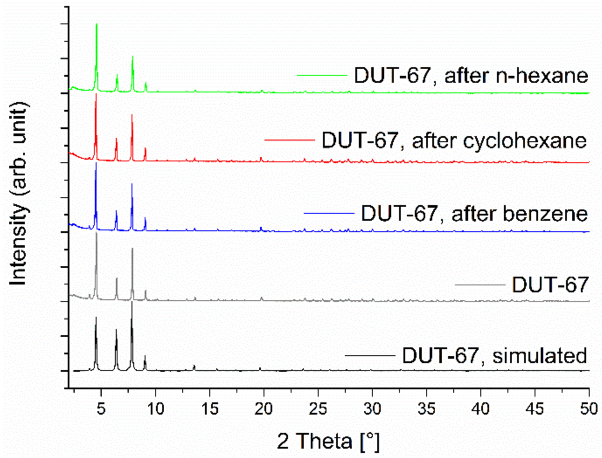

Figure S20 *PXRDs of DUT-67 after VOC-sorption, compared with MOF before sorption and with simulated pattern (CSD-Number: 921644).*[17]

Ti-MOFs

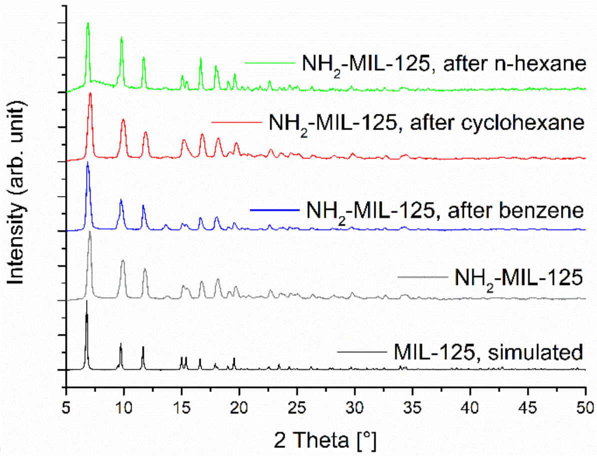

a)

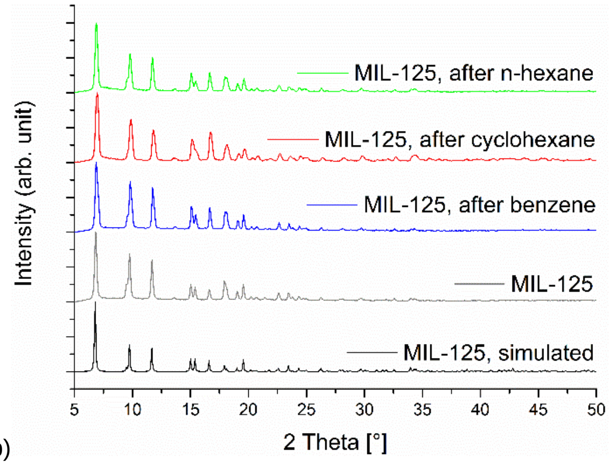

b)

Figure S21 *(a) PXRDs of NH_2-MIL-125 after VOC-sorption, compared with MOF before sorption and with simulated pattern (CSD-Number: 751157).*[20] *(b) PXRDs of MIL-125 after VOC-sorption, compared with MOF before sorption and with simulated pattern (CSD-Number: 751157).*[20]

Cr-MOF

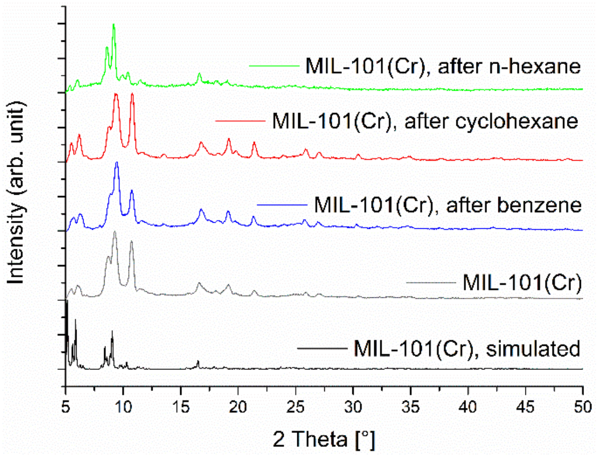

Figure S22 *PXRDs of MIL-101(Cr) after VOC-sorption, compared with MOF before sorption and with simulated pattern (CSD-Number: 605510).*[22]

ZIFs

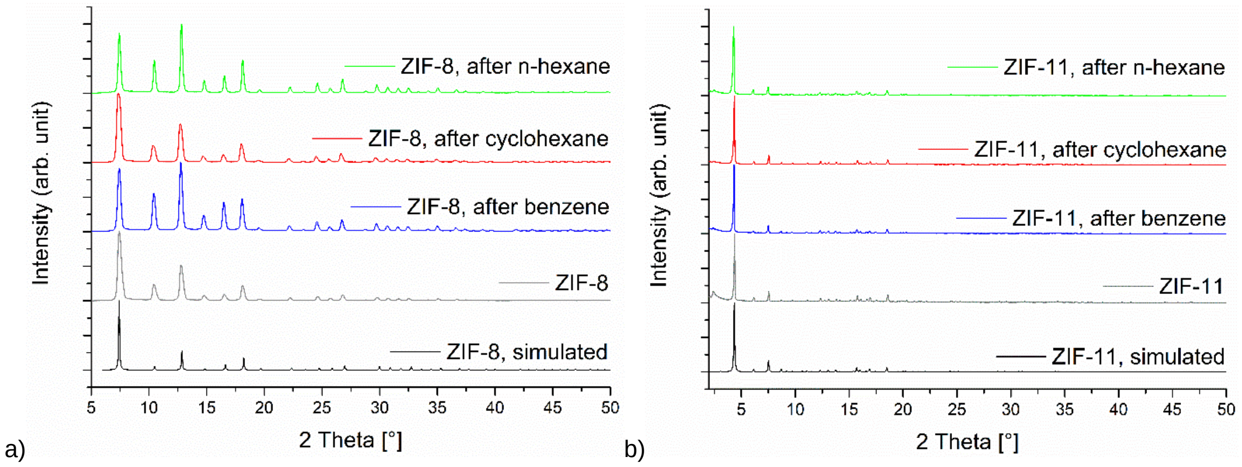

Figure S23 *(a) PXRDs of ZIF-8 after VOC-sorption, compared with MOF before sorption and with simulated pattern (CSD-Number: 864309).[24] (b) PXRDs of ZIF-11 after VOC-sorption, compared with MOF before sorption and with simulated pattern (CSD-Number: 602545).[26]*

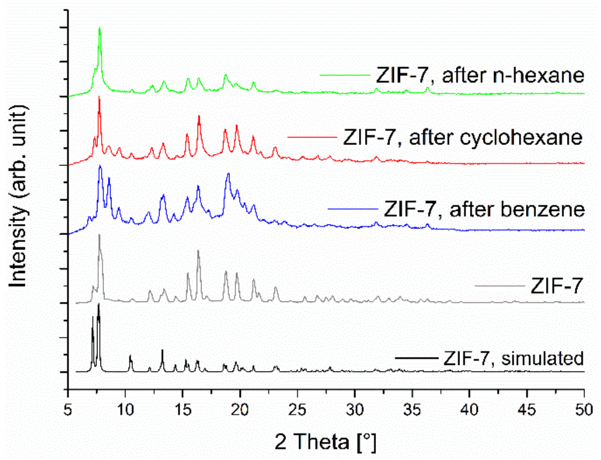

Figure S24 *PXRDs of ZIF-7 after VOC-sorption, compared with MOF before sorption and with simulated pattern (CSD-Number: 1036075).[40]*

S4 Nitrogen sorption experiments (T = 77 K)

Al-MOFs

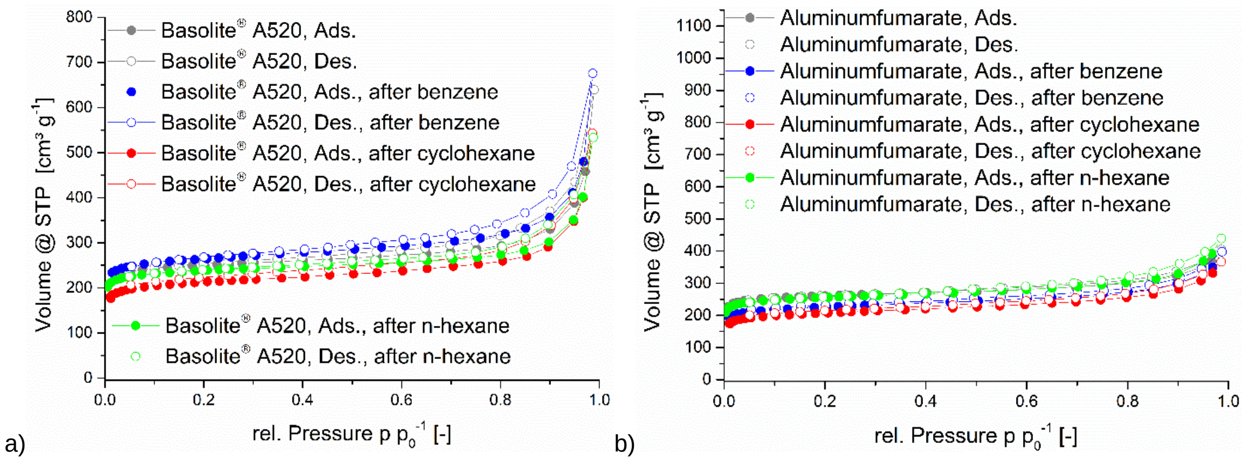

Figure S25 *(a) Nitrogen sorption of Basolite® A520 after VOC-sorption, compared with MOF before sorption. (b) Nitrogen sorption of Aluminumfumarate after VOC-sorption, compared with MOF before sorption.*

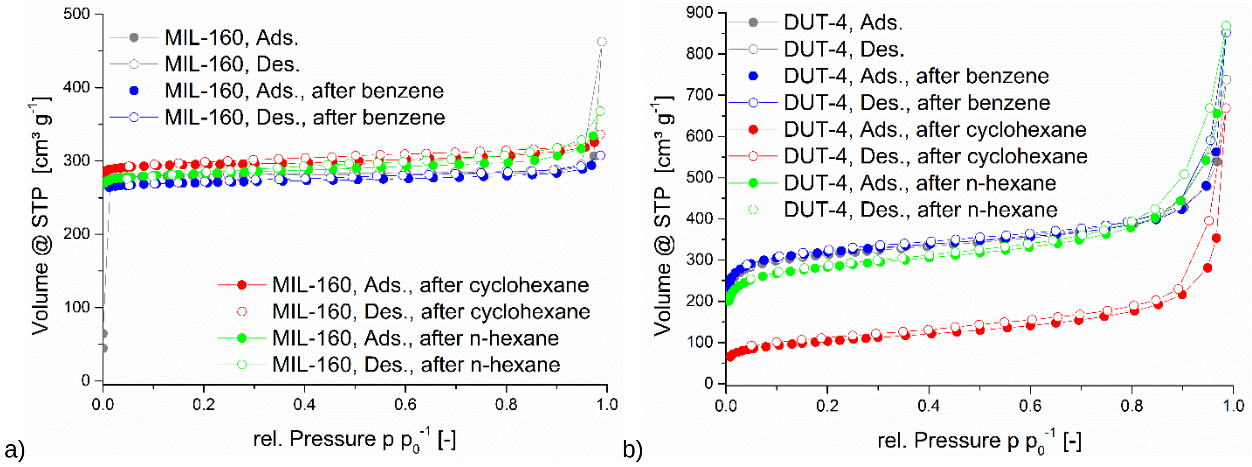

Figure S26 *(a) Nitrogen sorption of MIL-160 after VOC-sorption, compared with MOF before sorption. (b) Nitrogen sorption of DUT-4 after VOC-sorption, compared with MOF before sorption.*

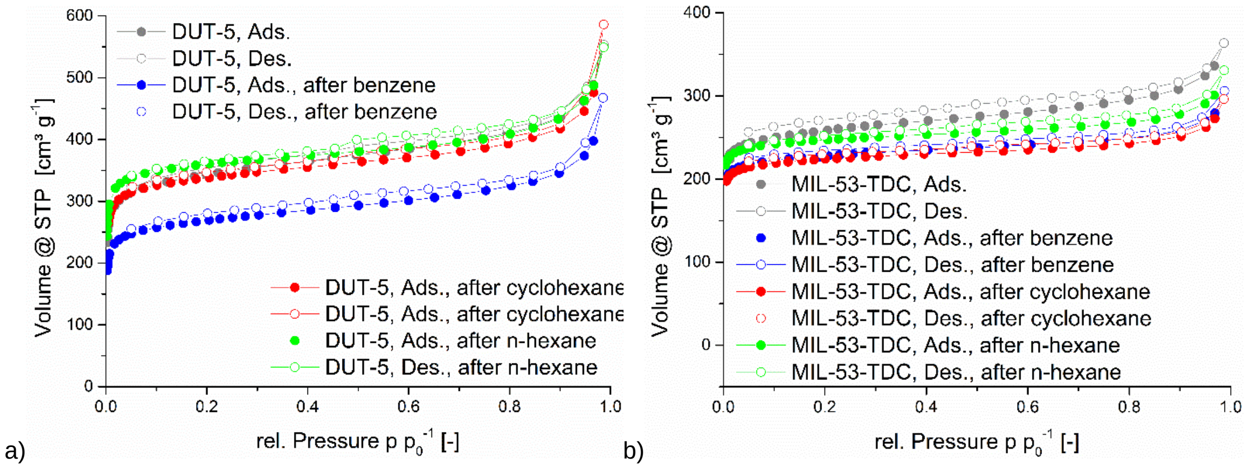

Figure S27 *(a) Nitrogen sorption of DUT-5 after VOC-sorption, compared with MOF before sorption. (b) Nitrogen sorption of MIL-53-TDC after VOC-sorption, compared with MOF before sorption.*

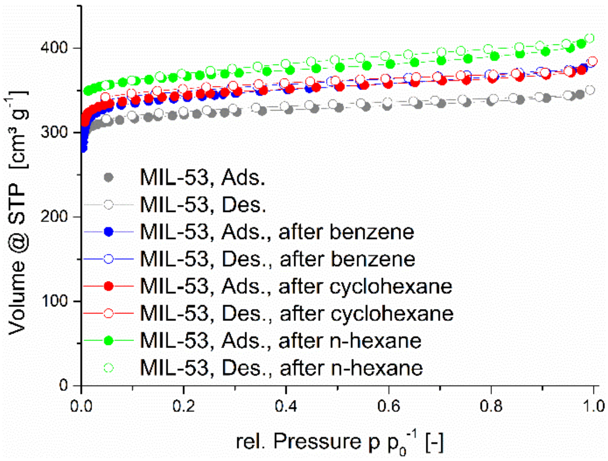

Figure S28 *Nitrogen sorption of MIL-53 after VOC-sorption, compared with MOF before sorption.*

Zr-MOFs

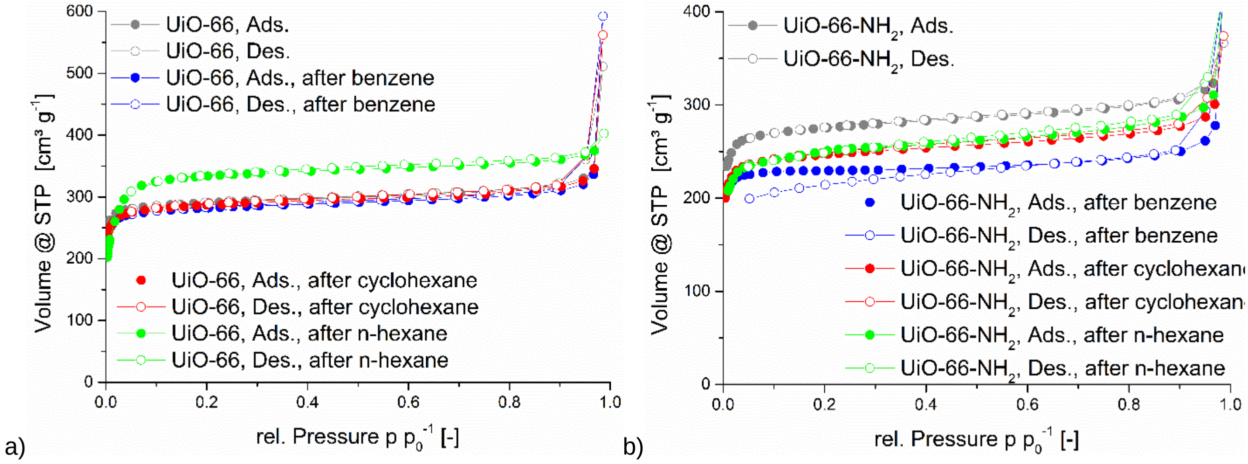

Figure S29 *(a) Nitrogen sorption of UiO-66 after VOC-sorption, compared with MOF before sorption. (b) Nitrogen sorption of UiO-66-NH_2 after VOC-sorption, compared with MOF before sorption.*

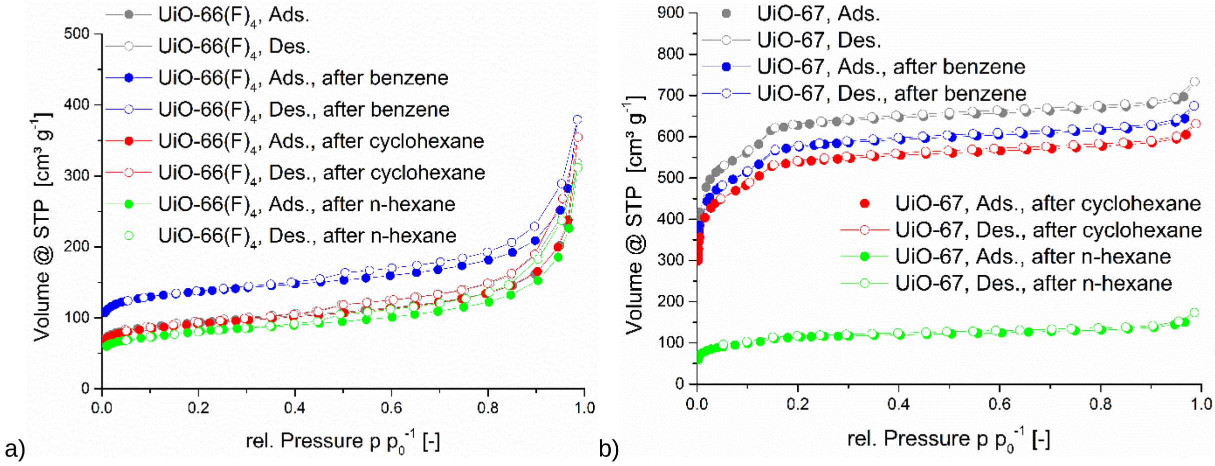

Figure S30 *(a) Nitrogen sorption of UiO-66(F)$_4$ after VOC-sorption, compared with MOF before sorption. (b) Nitrogen sorption of UiO-67 after VOC-sorption, compared with MOF before sorption.*

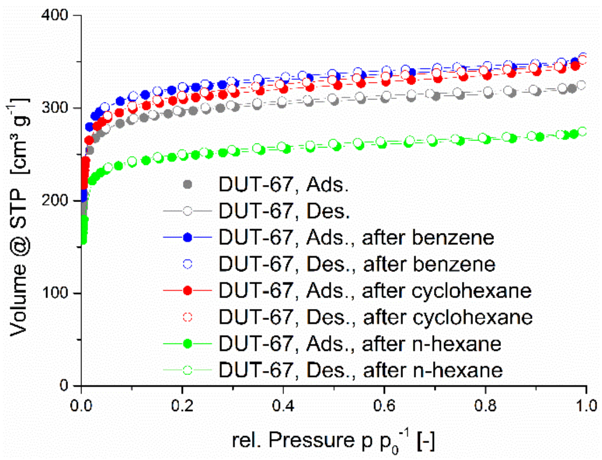

Figure S31 *Nitrogen sorption of DUT-67 after VOC-sorption, compared with MOF before sorption.*

Ti-MOFs

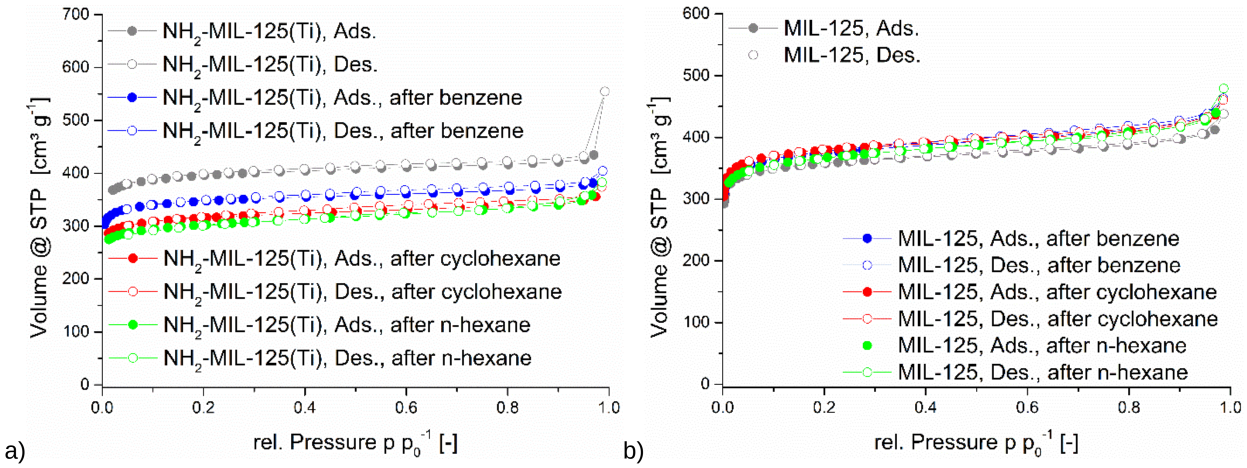

Figure S32 *(a) Nitrogen sorption of NH_2-MIL-125 after VOC-sorption, compared with MOF before sorption. (b) Nitrogen sorption of MIL-125 after VOC-sorption, compared with MOF before sorption.*

Cr-MOF

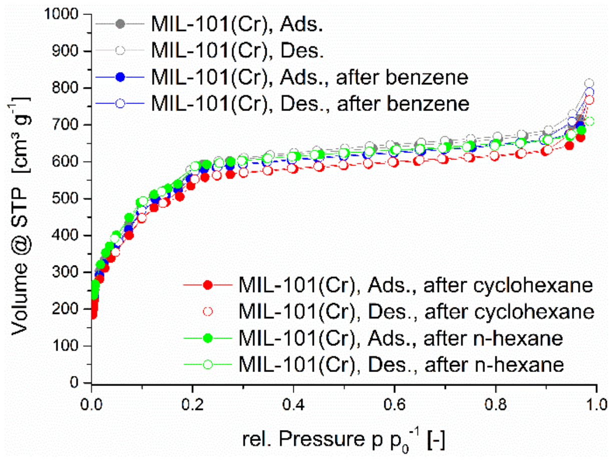

Figure S33 *Nitrogen sorption of MIL-101(Cr) after VOC-sorption, compared with MOF before sorption.*

ZIFs

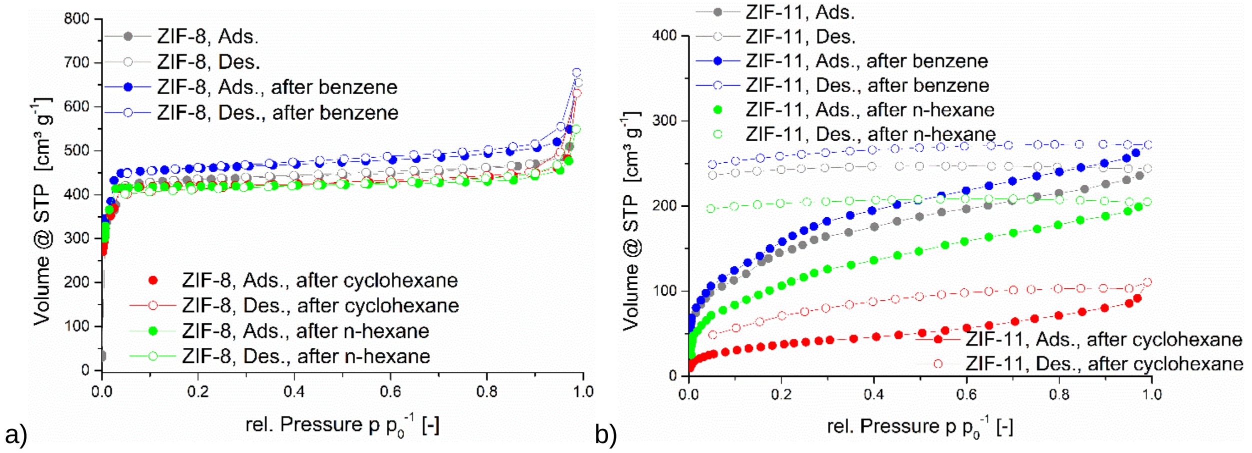

***Figure S34** (a) Nitrogen sorption of ZIF-8 after VOC-sorption, compared with MOF before sorption. (b) Nitrogen sorption of ZIF-11 after VOC-sorption, compared with MOF before sorption.*

S5 Carbon dioxide sorption experiments (T = 273 K)

ZIF-7

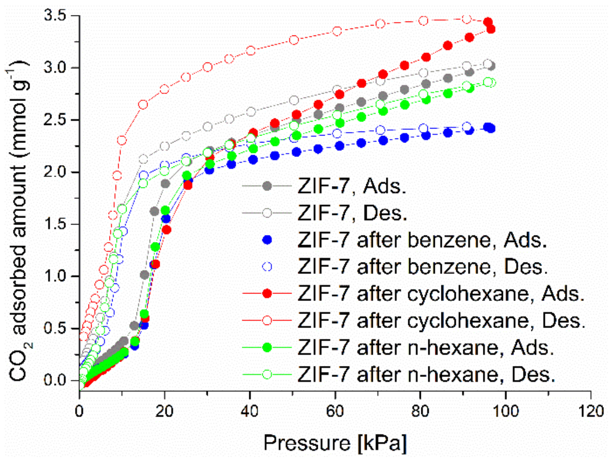

***Figure S35** Carbon dioxide sorption of ZIF-7 after VOC-sorption, compared with MOF before sorption.*

S6 Vapor sorption experiments (T = 293 K)

Equilibrium settings:

For each VOC measurement point the equilibrium is determined by three settings: maximum equilibrium points number (*P*), maximum equilibrium points interval time (*Int* in seconds) and maximum pressure-change rate limit (in *Torr* min^{-1}).
The equilibrium point numbers are the maximum numbers of individual points, which are used to measure the change of pressure. In our case these criteria were set to 10 points. The equilibrium points interval time describes the maximum time in seconds between the relevant points and was adapted due to the different pressure ranges. The last of the three settings is the pressure-change rate limit and it determines the maximum rate of pressure change per minute (Torr min^{-1}). Only values below this criterion will be considered as points in equilibrium.
These are the following conditions for the different VOCs due to their different relative pressure range:

Benzene

$p\ p_0^{-1}$ 0.001-0.4: *P*: 10, *Int*: 120, *Torr* min^{-1}: 0.001

$p\ p_0^{-1}$ 0.45-0.7: *P*: 10, *Int*: 180, *Torr* min^{-1}: 0.005

$p\ p_0^{-1}$ 0.8-0.9: *P*: 10, *Int*: 120, *Torr* min^{-1}: 0.01

$p\ p_0^{-1}$ 0.9-0.6: *P*: 10, *Int*: 90, *Torr* min^{-1}: 0.01

$p\ p_0^{-1}$ 0.5-0.01: *P*: 10, *Int*: 120, *Torr* min^{-1}: 0.005

Cyclohexane

$p\ p_0^{-1}$ 0.001-0.4: *P*: 10, *Int*: 120, *Torr* min^{-1}: 0.001

$p\ p_0^{-1}$ 0.45: *P*: 10, *Int*: 180, *Torr* min^{-1}: 0.001

$p\ p_0^{-1}$ 0.5-0.7: *P*: 10, *Int*: 180, *Torr* min^{-1}: 0.005

$p\ p_0^{-1}$ 0.8-0.9: *P*: 10, *Int*: 120, *Torr* min^{-1}: 0.01

$p\ p_0^{-1}$ 0.9-0.6: *P*: 10, *Int*: 90, *Torr* min^{-1}: 0.01

$p\ p_0^{-1}$ 0.5-0.01: *P*: 10, *Int*: 120, *Torr* min^{-1}: 0.005

n-Hexane

$p\ p_0^{-1}$ 0.001-0.45: *P*: 10, *Int*: 120, *Torr* min^{-1}: 0.01

$p\ p_0^{-1}$ 0.5-0.9: *P*: 10, *Int*: 180, *Torr* min^{-1}: 0.02

$p\ p_0^{-1}$ 0.9-0.6: *P*: 10, *Int*: 90, *Torr* min^{-1}: 0.01

$p\ p_0^{-1}$: 0.06-0.01: *P*: 10, *Int*: 120, *Torr* min^{-1}: 0.01

For each measured pressure point, the program performs a linear fit over a maximum of 10 points (to make it comparable), where each point is apart the maximum number of seconds as determined by the equilibrium points interval time.
If the slope of the fitted internal curve is below the pressure-change rate limit, the measurement will be defined to be in equilibrium. Due to the huge amount of data, these data were not given in the Supplementary Information.
The VOC *n*-hexane has the highest absolute pressure, compared to benzene and cyclohexane and therefore the measurement settings were adapted, by shortened equilibrium range.

Al-MOFs

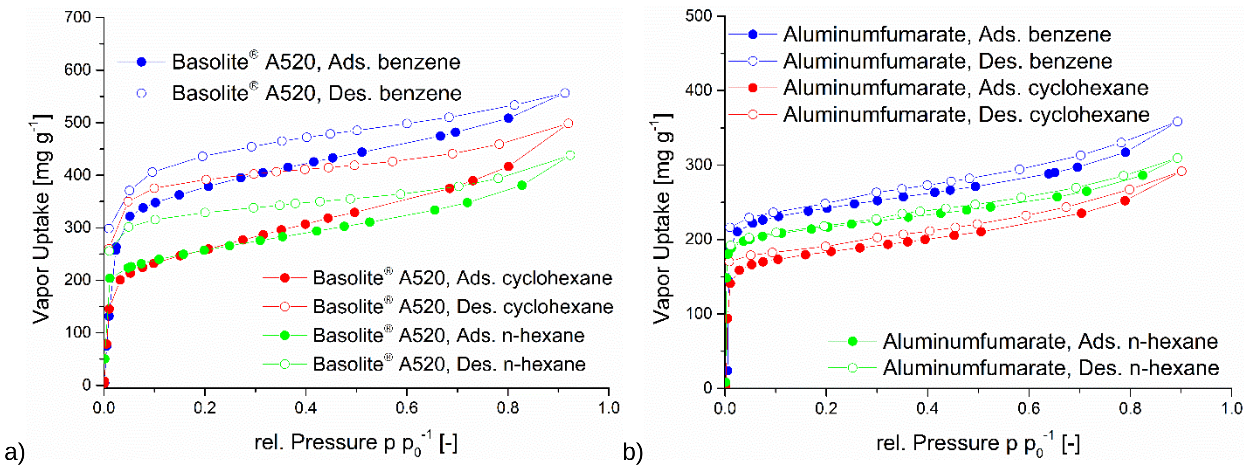

Figure S36 *(a) VOC sorption of Basolite® A520 for benzene/cyclohexane/n-hexane. (b) VOC sorption of Aluminumfumarate for benzene/cyclohexane/n-hexane.*

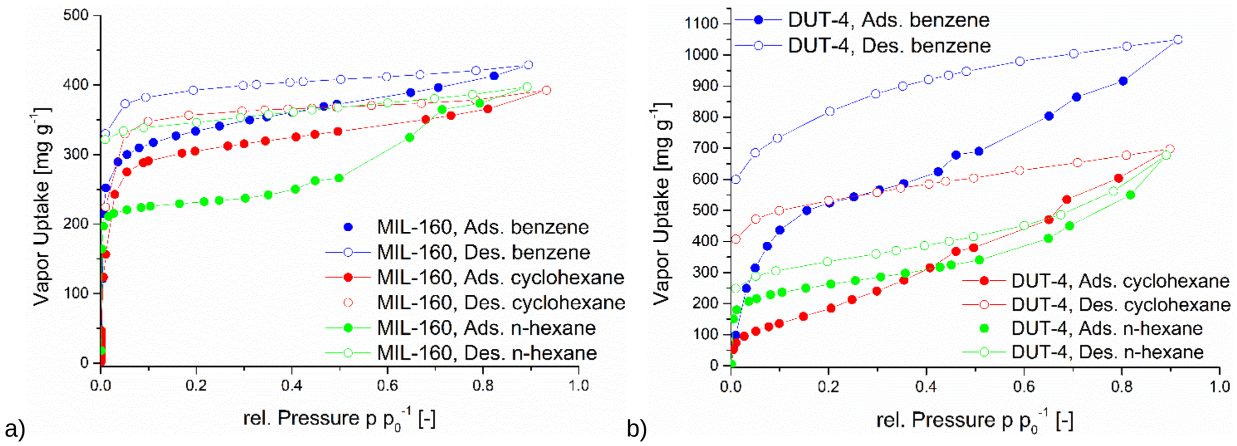

Figure S37 *(a) VOC sorption of MIL-160 for benzene/cyclohexane/n-hexane. (b) VOC sorption of DUT-4 for benzene/cyclohexane/n-hexane.*

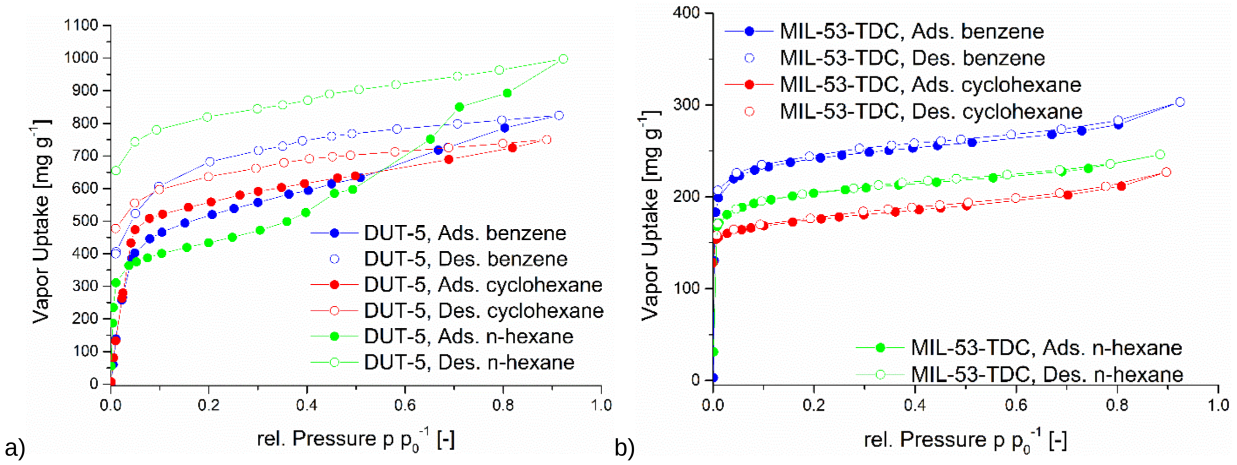

Figure S38 *(a) VOC sorption of DUT-5 for benzene/cyclohexane/n-hexane. (b) VOC sorption of MIL-53-TDC for benzene/cyclohexane/n-hexane.*

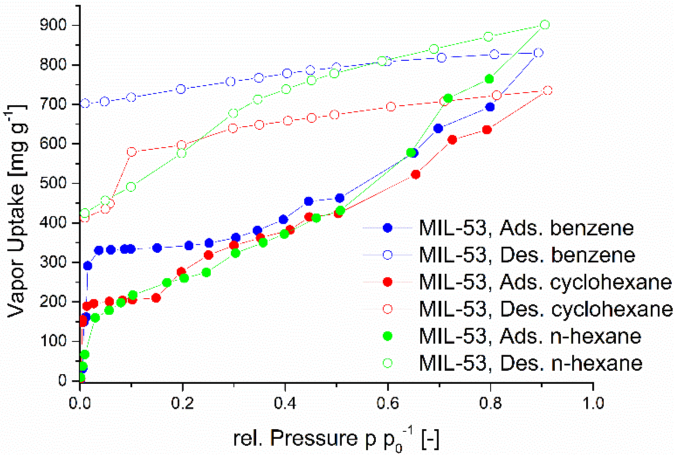

Figure S39 *VOC sorption of MIL-53 for benzene/cyclohexane/n-hexane.*

The aluminum-MOFs all show an early uptake in the low relative pressure range and uptakes between 303 mg g^{-1} and 1050 mg g^{-1} for benzene, 227 mg g^{-1} to 750 mg g^{-1} for cyclohexane and 246 mg g^{-1} and 997 mg g^{-1} for *n*-hexane. For all Al-MOFs, the first uptake step is completed in a pressure range below 0.1 p p_0^{-1}. For DUT-4 and MIL-53 a further additional continuous uptake takes place over the entire pressure range. MIL-160 and DUT-5 show only minor differences in the adsorption of benzene and cyclohexane, both, in the maximum uptake and over the entire relative pressure range. In the case of Basolite® A520 and Aluminumfumarate, the benzene uptake is higher over the entire pressure ranger than for cyclohexane and *n*-hexane. Interesting is the difference between Aluminumfumarate and Basolite® A520 in their adsorption capacity and hysteresis, because both materials are chemically the same MOF and the only difference is that Basolite® A520 is the industrial product, albeit with different surface area and porosity characteristics. Only for DUT-4 is the amount of benzene which is absorbed significantly higher than for cyclohexane and *n*-hexane over the entire measuring range and the maximum absorption capacity is over 350 mg g^{-1} more for benzene. The MOFs all show a very large hysteresis for benzene, cyclohexane and *n*-hexane, except for Aluminumfumarate and MIL-53-TDC.

Zr-MOFs

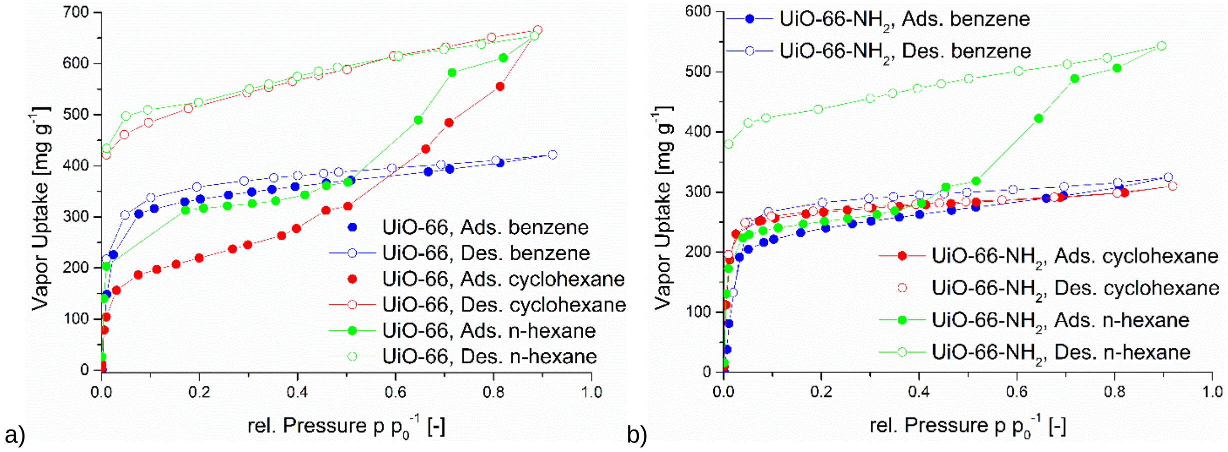

Figure S40 *(a) VOC sorption of UiO-66 for benzene/cyclohexane/n-hexane. (b) VOC sorption of UiO-66-NH$_2$ for benzene/cyclohexane/n-hexane.*

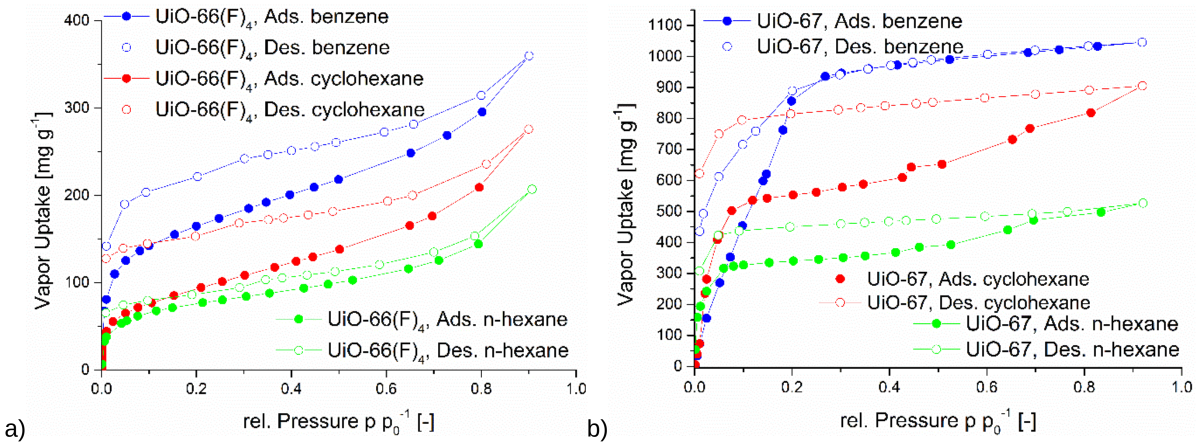

Figure S41 *(a) VOC sorption of UiO-66(F)$_4$ for benzene/cyclohexane/n-hexane. (b) VOC sorption of UiO-67 for benzene/cyclohexane/n-hexane.*

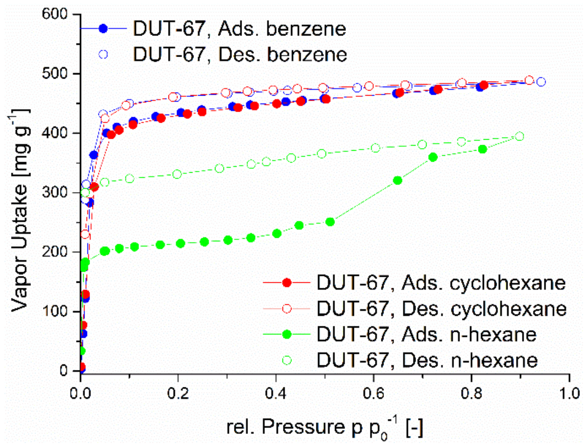

Figure S42 *VOC sorption of DUT-67 for benzene/cyclohexane/n-hexane.*

The zirconium MOFs show a high uptake in the low-pressure range and uptakes between 325 mg g^{-1} and 1046 mg g^{-1} for benzene, 276 mg g^{-1} to 905 mg g^{-1} for cyclohexane and 207 mg g^{-1} and 654 mg g^{-1} for *n*-hexane. The isotherm shape differs considerably, as well, as the occurrence of a hysteresis. A large hysteresis is significantly more common in the adsorption of cyclohexane compared to benzene and way more common for *n*-hexane. For example, only UiO-66-NH$_2$, UiO-66(F)$_4$ and UiO-67 show a hysteresis in the low-pressure range for benzene, but still small compared to cyclohexane and *n*-hexane sorption isotherms. Only with UiO-66-NH$_2$ cyclohexane shows no hysteresis. For the *n*-hexane sorptions only UiO-66(F)$_4$ has no significant hysteresis, but at the same time a low uptake. The absolute VOC uptakes for benzene and cyclohexane do not differ that much for the zirconium MOFs, except for UiO-66 where there is a difference of over 240 mg g^{-1} in favor of cyclohexane. The uptake of *n*-hexane and the shape of the isotherms are different compared to benzene and cyclohexane, but it is no clear trend recognizable. For UiO-66 and UiO-66-NH$_2$ the uptake for *n*-hexane is the same (UiO-66) or higher (UiO-66-NH$_2$) than the other VOCs. The other three zirconium MOFs have a lower *n*-hexane uptake.
All MOFs show a high affinity for *n*-hexane, too, but the shape of the isotherm is different to benzene and cyclohexane for the most MOFs, as mentioned before. For cyclohexane and *n*-hexane is a additional uptake recognizable for UiO-66 and UiO-66-NH$_2$. Benzene and cyclohexane show almost identical uptakes in UiO-66-NH$_2$ and in DUT-67. For the other Zr-MOFs the difference is in a small range, except for UiO-66.

The desorption process for *n*-hexane is only for UiO-66(F)$_4$ completed, all other four MOFs show an open, non-closed hysteresis. Due to this fact *n*-hexane still remains in the pores. This phenomenon exists as well for cyclohexane in UiO-66 and UiO-67 in an extended way.

Ti-MOFs

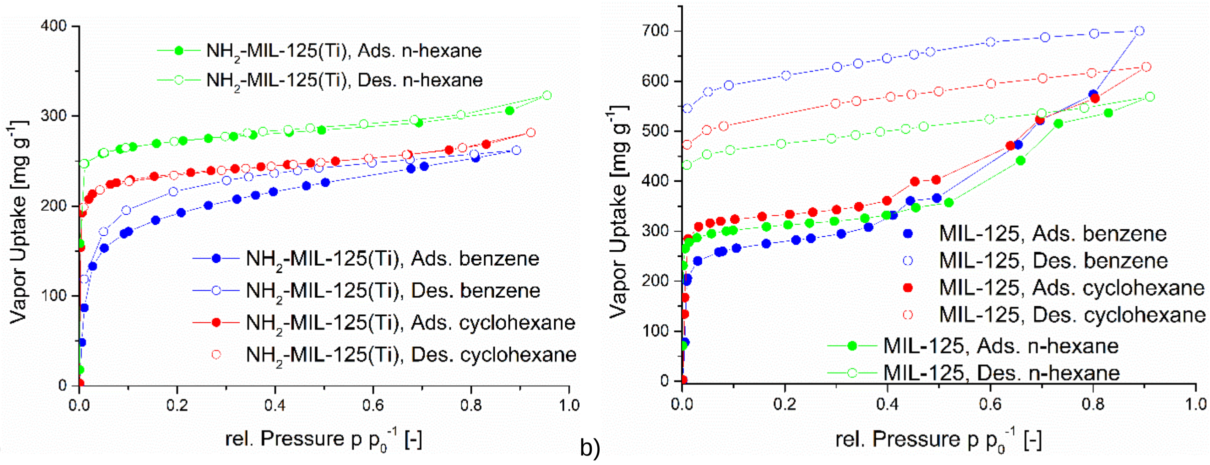

Figure S43 *(a) VOC sorption of NH_2-MIL-125 for benzene/cyclohexane/n-hexane. (b) VOC sorption of MIL-125 for benzene/cyclohexane/n-hexane.*

Like the zirconium MOFs, NH_2-MIL-125 shows no major difference in the final uptake of benzene, cyclohexane or *n*-hexane, albeit the uptake values are smaller than for UiO-66-NH_2. Analogous to UiO-66-NH_2, the hysteresis is larger for benzene than for cyclohexane or *n-hexane*. The initial uptake also takes place at a lower pressure for cyclohexane and *n*-hexane, compared to benzene. Between NH_2-MIL-125 and MIL-125 the isotherm shape, hysteresis and uptakes differ considerably. In MIL-125 all three VOC isotherms have a similar shape and a second uptake step after a relative pressure of 0.5 p p_0^{-1}. Furthermore, all VOCs have a large non-closen hysteresis. MIL-125 has an opposite maximum uptake order (benzene > cyclohexane > *n*-hexane) compared to NH_2-MIL-125 (*n*-hexane > cyclohexane > benzene).

Cr-MOF

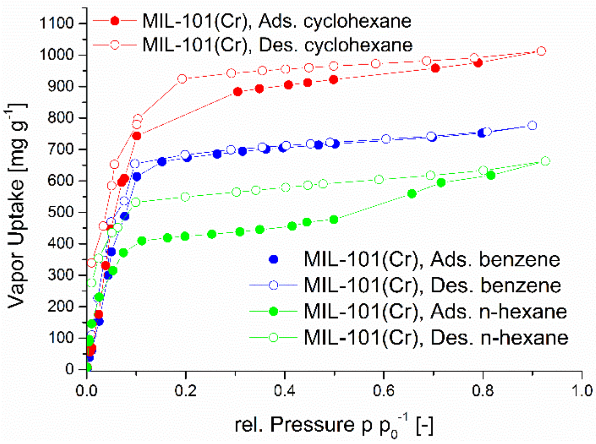

Figure S44 *VOC sorption of MIL-101(Cr) for benzene/cyclohexane/n-hexane.*

MIL-101(Cr) has the same terephthalate or benzene-1,4-dicarboxylate linker as MIL-53, UiO-66, MIL-125. MIL-101(Cr) it is no longer an only microporous MOF but also has mesopores (Figure S10). The uptake occurs in the low-pressure range, nearly the same as with the microporous MOFs. There is also a hysteresis with cyclohexane, compared to the MOF with the same linker UiO-66 and a larger hysteresis for *n*-hexane. Whereas there is almost no hysteresis for the benzene sorption. The maximum adsorption for cyclohexane is 236 mg g^{-1} more than the adsorption capacity for benzene. *n*-Hexane has the lowest amount with 663 mg g^{-1}. The large pores of MIL-101(Cr) are suitable for a high capacity.

ZIFs

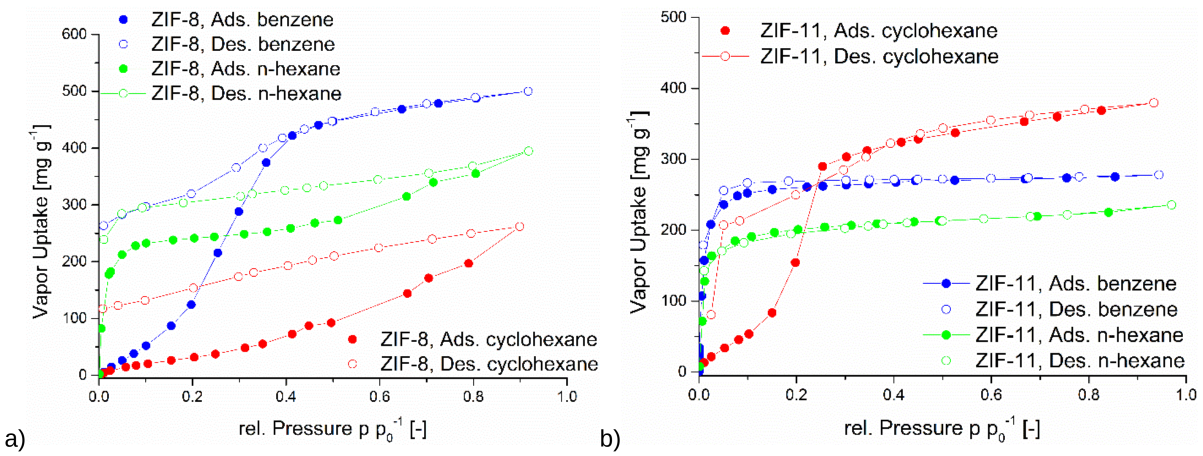

Figure S45 *(a) VOC sorption of ZIF-8 for benzene/cyclohexane/n-hexane. (b) VOC sorption of ZIF-11 for benzene/cyclohexane/n-hexane. The drop in the desorption curve below the adsorption branch for cyclohexane represent a measurement error.*

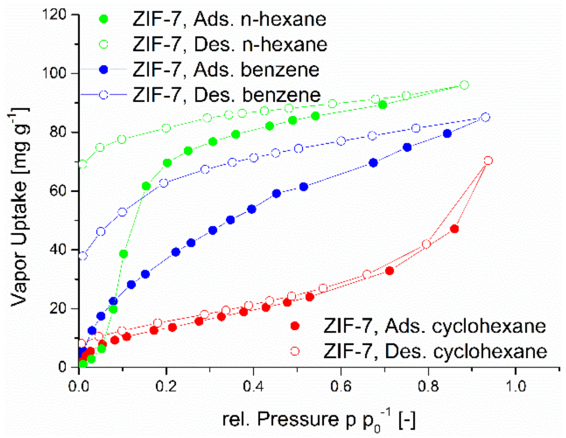

Figure S46 *VOC sorption of ZIF-7 for benzene/cyclohexane/n-hexane.*

ZIF-8 shows a clear difference in the maximum uptake of benzene, cyclohexane and *n*-hexane and the relative pressure range, where the uptake takes place. The benzene uptake occurs with an S-shaped uptake step between 0.1 and 0.5 p p_0^{-1} and increases to almost 500 mg g^{-1}. The uptake of cyclohexane is significantly lower and takes place constantly over the entire relative pressure range. The *n*-hexane uptake starts immediately at the beginning at a relative pressure below 0.04 p p_0^{-1}. All three adsorbates show a large hysteresis, the hysteresis with benzene only occurring at a relative pressure below 0.5 p p_0^{-1}.

The sorption isotherms of ZIF-11 and ZIF-7 differ significantly, not only between the adsorbates, but also between both ZIFs, despite the same metal and linker. In the case of ZIF-11, all three adsorbates show a pronounced uptake step, but in different relative pressure ranges. The uptake of benzene and *n*-hexane is in a low-pressure range of less than 0.1 p p_0^{-1} and has no additional uptake thereafter, as with type-I isotherms. [41] The increase in cyclohexane adsorption is from a relative pressure range of 0.1 to 0.3 p p_0^{-1} and then flattens off significantly with a small increase of less than 100 mg g^{-1} up to 0.9 p p_0^{-1}.

For ZIF-7, an S-shaped uptake is seen for *n*-hexane which above 0.1 p p_0^{-1} also exceeds the benzene and cyclohexane uptake. Yet, the maximum *n*-hexane uptake compared with the other MOFs is still low (Table S7). For cyclohexane ZIF-7 gives only a low constant uptake. Compared to cyclohexane, the benzene uptake is already significantly higher. Compared to ZIF-11, all VOC uptake values in ZIF-7 are significantly lower. The pores of ZIF-11 are larger than those of ZIF-7. The inner pore diameter of ZIF-11 is 14.6 Å and of ZIF-7 only 4.3 Å.[25]

Table S4 *Adsorption isotherm and hysteresis type for C_6 vapor adsorption in MOFs.*[a]

**MOF**	**Adsorption isotherm type (desorption hysteresis at 293 K)** [b]			**Comment**
	**benzene**	**cyclohexane**	***n*-hexane**	
Basolite® A520 (commercial Alfum)	Ib+II (H4 wide)	Ib+II (H4 wide)	Ia+II (H4 wide)	
Alfum (own synthesis)	Ia+II (H4 narrow)	Ia+II (H4 narrow)	Ia+II (H4 narrow)	
MIL-160	Ib+II (H4 wide)	Ib+II (H4 wide)	IVa (H2a very wide)	steps in *n*-hexane ads. at $p\ p_0^{-1}$ ~0.5 and ~0.7
DUT-4	F-I (H2a or H4 very wide	II (H3 very wide)	Ia+II (H4 wide)	two small steps in benzene and cyclohexane ads. at $p\ p_0^{-1}$ ~0.45 and ~0.7
DUT-5	Ib+II (H4 wide)	Ib+II (H4 wide)	Ia+II (H4 very wide)	two small steps in *n*-hexane ads. at $p\ p_0^{-1}$ ~0.45 and ~0.7
MIL-53-TDC	Ia(+II) (H4 very narrow)	Ia(+II) (H4 very narrow)	Ia(+II) (no hysteresis)	only very slight increase of uptake with $p\ p_0^{-1}$, very small hystereses
MIL-53(Al)	F-I (H2a very wide)	F-I (H2a very wide)	F-I (H2a very wide)	F-I based on the known flexible character; steps in ads. of all vapors, for cyclohexane already at $p\ p_0^{-1}$ ~0.2; hysteresis steps for *n*-hexane and cyclohexane; step in hysteresis for cyclohexane and *n*-hexane at low $p\ p_0^{-1}$
UiO-66	Ib (H4, narrow)	Ib+II (H2b very wide)	F-I or IV (H2b very wide)	only very slight increase of benzene uptake with $p\ p_0^{-1}$, very small benzene hysteresis; steps in cyclohex. and *n*-hexane ads. after $p\ p_0^{-1}$ ~0.45
UiO-66-NH_2	Ia+II (H4 narrow)	Ia (no hysteresis)	F-I or IV (very wide)	steps in *n*-hexane ads. after $p\ p_0^{-1}$ ~0.45
UiO-66(F)$_4$	Ib+II (H4 wide)	Ia+II (H4 wide)	Ia+II (H4 narrow)	
UiO-67	Ib (wide hyst. <0.3 $p\ p_0^{-1}$)	F-I or Ib+II (H2b very wide)	F-I or IV (H2b wide)	no hysteresis for benzene >0.3 $p\ p_0^{-1}$; steps in cyclohex. and *n*-hexane ads. at $p\ p_0^{-1}$ ~0.45
DUT-67	Ib(+II) (H4 narrow)	Ib(+II) (H4 narrow)	F-I or IV (H2b wide)	isotherms of cyclohex. and benzene almost super-imposible); steps in *n*-hexane ads. after $p\ p_0^{-1}$ ~0.45
NH_2-MIL-125	Ib+II (H4 narrow)	Ia(+II) (no hysteresis)	Ia(+II) (almost no hyst.)	
MIL-125	Ia+II (H2b very wide)	F-I or IV (H2b very wide)	F-I or IV (H2b very wide)	steps in ads. of all vapors, most prominently for benzene and cyclohex. at $p\ p_0^{-1}$ ~0.45
MIL-101(Cr)	Ib (narrow hyst. <0.2 $p\ p_0^{-1}$)	Ib+II (H2 narrow)	F-I or IV (H2b wide)	hysteresis for benzene only below 0.2 $p\ p_0^{-1}$
ZIF-8	F-I (wide hyst. <0.4 $p\ p_0^{-1}$)	F-III or III (H2b wide)	F-I or IV (H2b wide)	hysteresis for benzene only below 0.5 $p\ p_0^{-1}$ with step in

				hysteresis for benzene at low $p\,p_0^{-1}$; S-shaped benzene ads. with inflection point at $p\,p_0^{-1}$ ~0.3
ZIF-11	Ib (hyst. <0.4 $p\,p_0^{-1}$)	F-I or IV (H2a wide)	Ib (no hysteresis)	S-shaped cyclohex. ads. with inflection point at $p\,p_0^{-1}$ ~0.25
ZIF-7	Ib (H2b wide)	II (H3 narrow)	F-III or V (H2b wide)	S-shaped hexane ads. with inflection point at $p\,p_0^{-1}$ ~0.1

[a] The type was chosen according to the classifications given in ref. 41 and 42 to represent visually the best approximation.
[b] wide and narrow with respect to the hysteresis describe a large and small, respectively, separation between the ad- and desorption branch.

Classification of adsorption isotherms

Type I isotherms are reversible and have a steep uptake at very low-pressure after which almost no further uptake takes place. Thus, the maximum uptake is reached before $p\,p_0^{-1} = 1$. A steep uptake at very low $p\,p_0^{-1}$ is due to strong adsorbate-adsorbent interactions. Type Ia has sharp knee (the point where the isotherm turns from almost vertical to horizontal line). Type Ib has a more gradual, less distinctive knee.

Type II isotherms derive from the adsorption on non- to macroporous adsorbents with multilayer formation at high $p\,p_0^{-1}$. The increasing uptake in a Type II branch towards $p\,p_0^{-1} = 1$ can come from the adsorption on the outer surface of a fine powder and from the filling of interparticle voids.

Standard Type IV isotherms are seen with mesoporous adsorbents where initially (at low $p\,p_0^{-1}$) the adsorbate-adsorbent interaction on the pore walls and at higher $p\,p_0^{-1}$ the pore condensation with multilayer formation determines the isotherm shape. An important aspect of Type IV isotherms is the saturation plateau at high $p\,p_0^{-1}$.[41]

A Type F-I isotherm reflects the uptake in a flexible microporous material with a structure transformation through a gradual opening of a small pore to a large pore.[42] It can be noted that from the isotherm shape alone Type F-I and Type IV appear identical. The F-I or IV isotherms have a similar uptake in the low and higher pressure regions.

Origin of hysteresis

Hysteresis in physisorption originates from kinetic effects of adsorption metastability, network effects and pore blocking, such that wide pores are only accessible through narrow pore windows. An H4 hysteresis is seen for adsorption isotherms which are a composite of Type I and II. If the isotherm is fully in equilibrium its shape only reflects the thermodynamics of the ad- and desorption. Thus, a gradual uptake or release cannot be descibed as 'slow'. As noted above, a hysteresis is, however, indicative of kinetic effects. Such a kinetic effect can be observed for larger adsorbates and relative smaller pore (window or neck) diameters. Only when the adsorption is driven by larger adsorption energies at the border of physisorption towards chemisorption the hysteresis will be due to thermodynamic effects of strong adsorbate-adsorbent interactions. Strong hydrogen-bonding and strong $\pi\cdots\pi$ or C-H$\cdots\pi$ interactions or adsorbate interactions with open-metal sites,[43] then allow the guest only to be removed at the end of the desorption branch, that is at very low $p\,p_0^{-1}$ and possibly even under additional heating.[44] In this respect we note that all of the desorption branches with a wide and very wide hysteresis did not close until until very low $p\,p_0^{-1}$. While for benzene this non-closure could have been due to strong $\pi\cdots\pi$ interactions, any strong supramolecular interactions can be ruled out for *n*-hexane and cyclohexane. This leaves then only kinetic effects based on the relative guest-pore size for the latter and, in turn, also suggests the same for benzene. In general it can be envisioned that the pore structures of frameworks limit the diffusion of molecules of the size of benzene, cyclohexane and *n*-hexane more strongly than that of small gas molecules such as N_2, CO_2 etc.[44]

Description of C_6-VOC isotherms

The categorization in Table S4 may not reflect all small features of the isotherms.

In general, the thermodynamics of the physisorption of gases and solvent vapors depend on absorbate-absorbate and absorbate-absorbent interactions with the kinetics being determined mainly by the molecular size of the adsorptive and the pore size of the adsorbent. (In physisorption the host network is the adsorbent. The guest in the fluid phase is the adsorptive; the guest in the adsorbed state is the adsorbate. Often the terms adsorptive and adsorbate seem to be used indiscriminately.[41]) The magnitude of guest-host (absorbate-absorbent) interactions in physisorption depends on the possible supramolecular interactions between the guest molecules and the pore walls of the framework, giving rise to the different isotherm types of adsorption processes.[44] Benzene adsorption in MOFs may be expected to follow a Type-I isotherm, since in the low $p\,p_0^{-1}$ region benzene adsorption can reach saturation due to $\pi\cdots\pi$-interactions with the often aromatic core of the linker in the MOF. Cyclohexane and *n*-hexane can only enter into weaker van-der-Waals interactions with the framework so that a gradually increasing adsorption with the increase in $p\,p_0^{-1}$ of the absorbate could be foreseen.[44]

UiO-66, MIL-53 and MIL-125 show the same isotherm shape and a large hysteresis and all of them have the same linker (terephthalate). MIL-101(Cr) shows a different isotherm shape, without the additional step already mentioned above, but with the same linker. This may be attributed to the large pores (mesoporous) and thus lower interaction. The additional uptake in the three MOFs can be explained by VOC-VOC interaction, which can be better formed in a smaller pore or pore condensation. In addition, MIL-53 may still have a breathing effect in the pore and promote additional uptake.

This additional uptake step (MIL-53, UiO-66, MIL-125) may also be a reason for the poor desorption and the associated large hysteresis. Both, the amine functionalization and complete fluorination of the linker result in lower total uptake, as the pores and the pore window of the MOFs are smaller, but favor better desorption and the formation of a smaller hysteresis. When the π-system and thus the pores become larger, as in DUT-4, DUT-5 and UiO-67, the isotherms also show further steps and larger hysteresis. These MOFs have larger pores than the terephthalate MOFs, but are still microporous, in contrast to MIL-101(Cr). DUT-5 is an exception here with a lower second uptake step and smaller hysteresis, although this can also be explained by the BET surface area, which is lower than the literature values and lower than the surface area of DUT-4.

The functionalization of the MOFs like in UiO-66-NH_2, UiO-66$(F)_4$ and NH_2-MIL-125 reduces the pore window and the pore size/diameter. This has an influence on the sorption capacity and the isotherm shape of the MOFs, in comparison with their unfunctionalized derivatives UiO-66 and MIL-125. This reduction of the MOF pores leads to a no longer existing second uptake step for most of the MOF-VOC combinations and a smaller to nearly non-existing hysteresis. The reduced pore size could lead to a different arrangement of VOC molecules in the pore and less VOC-VOC interactions, which would lead to a second uptake step and to a strong hysteresis.

The MOFs MIL-160, MIL-53-TDC and DUT-67 have all a heteroatom inside their ligand, but different pore windows (cf. Table S2). MIL-160 and DUT-67 have a smaller pore than MIL-53-TDC, but DUT-67 and MIL-53-TDC have the same thiophenedicarboxylate ligand (cf. Figure 1 in the main text). All three MOFs show no second uptake step and a very small hysteresis for benzene and cyclohexane. The sorption isotherms for *n*-hexane are different than for benzene and cyclohexane. The two MOFs with the smaller pore window (MIL-160 and DUT-67) have a second uptake step and a huge hysteresis for the *n*-hexane sorption. The different structure and linker of the two MOFs suggests that this sorption effect may be related to the pore window. Possibly this window size is a bottleneck, both in adsorption and desorption, and prevents uniform uptake, as in MIL-53-TDC, which has a much larger pore window. MIL-53-TDC has the same isotherm shape for all three VOCs and a small closed hysteresis. The difference between the two ligands furandicarboxylate and thiophenedicarboxylate is the heteroatom and furandicarboxylate is the ligand with the weaker aromatic system. This can be seen in the slightly different isotherm shape, as MIL-53-TDC has an even earlier and more linear uptake step (more type Ia) than MIL-160 for the VOCs.

The ZIFs with their very small pore windows and gate-opening effects related to the linkers methylimidazolate and benzimidazolate show a different phenomenon, which is much more dependent on the particular VOC. The bulkiest VOC of these three is cyclohexane and the gate-opening effect blocks the adsorption in ZIF-8 and ZIF-7. In particular, uptake in ZIF-7 is extremely hindered or only occurs at high relative pressures around 0.8 p p_0^{-1}, and could be attributed to condensation of VOCs on the surface. For ZIF-8, the cyclohexane uptake rises over the entire pressure range, but remains very low relative to the other two VOCs. For benzene and *n*-hexane, isotherms with a clear uptake step are observed, but in different relative pressure ranges and an affinity of the ZIFs towards the different VOCs is clearly visible. Especially the clearly different isotherm curves for ZIF-11 and ZIF-7 are very interesting, since these are two MOFs with identical metal-linker composition and only a different 3D crystal packing. It can be seen that not only the metal and the linker have a significant influence, as in the case of the MOFs mentioned above, but also the structural composition of the porous compounds plays a major role.

Tabular summary of C_6-VOC uptake at different relative pressures, including BET-surface areas before and after VOC sorption.

Table S5 *Benzene sorption results at different relative pressures, including BET-surface areas before and after benzene sorption.*

MOF	BET-surface [m² g^{-1}]		benzene uptake [mg g^{-1}] (293 K)		
	before benzene sorption	after benzene sorption	p p_0^{-1} = 0.1	p p_0^{-1} = 0.3	p p_0^{-1} = 0.9
Alfum (Basolite® A520)	988	1034	347	401	551
Aluminumfumarate	1035	879	230	252	358
MIL-160	1161	1120	315	348	429
DUT-4	1764	1233	436	564	1031
DUT-5	1323	1038	462	557	820
MIL-53-TDC	1015	912	231	248	298
MIL-53(Al)	1325	1375	334	362	830
UiO-66	1178	1150	314	348	419
UiO-66-NH_2	1127	940	221	252	323
UiO-66$(F)_4$	346	521	142	183	359
UiO-67	2317	2046	460	946	1043
DUT-67	1178	1271	417	444	483
NH_2-MIL-125	1570	1390	172	205	262
MIL-125	1425	1471	265	293	701
MIL-101(Cr)	2002	2117	607	692	776
ZIF-8	1615	1785	52	290	498
ZIF-11	491	408	253	264	277
ZIF-7	3.03 mmol CO2 (275)	2.43 mmol CO2 (223)	25	47	83

MOF	benzene uptake [mg g^{-1}] (293 K)		
	p p_0^{-1} = 0.02	p p_0^{-1} = 0.05	p p_0^{-1} = 0.08
Alfum (Basolite® A520)	226	319	339

Aluminumfumarate	204	220	227
MIL-160	266	297	309
DUT-4	168	315	395
DUT-5	232	403	446
MIL-53-TDC	206	222	229
MIL-53(Al)	300	331	333
UiO-66	200	265	307
UiO-66-NH_2	128	204	215
UiO-66$(F)_4$	97	125	136
UiO-67	130	263	380
DUT-67	289	395	412
NH_2-MIL-125	113	152	165
MIL-125	223	249	260
MIL-101(Cr)	122	375	503
ZIF-8	11	26	41
ZIF-11	193	235	249
ZIF-7	9	17	23

Table S6 *Cyclohexane sorption results at different relative pressures, including BET-surface areas before and after cyclohexane sorption.*

**MOF**	**BET-surface [m² g^{-1}]**		**cyclohexane uptake [mg g^{-1}] (293 K)**		
	**before cyclohexane sorption**	**after cyclohexane sorption**	**p p_0^{-1} = 0.1**	**p p_0^{-1} = 0.3**	**p p_0^{-1} = 0.9**
Alfum (Basolite® A520)	988	825	233	283	485
Aluminumfumarate	1035	802	173	192	291
MIL-160	1161	1201	291	316	385
DUT-4	1764	372	136	241	698
DUT-5	1323	1301	518	591	750
MIL-53-TDC	1015	888	168	181	227
MIL-53(Al)	1325	1371	205	343	726
UiO-66	1178	1158	193	246	666
UiO-66-NH_2	1127	994	256	273	307
UiO-66$(F)_4$	346	339	76	108	276
UiO-67	2317	1928	521	577	890
DUT-67	1178	1238	412	441	487
NH_2-MIL-125	1570	1259	229	241	279
MIL-125	1425	1510	323	343	627
MIL-101(Cr)	2002	2054	737	880	1007

ZIF-8	1615	1650	20	46	262
ZIF-11	491	140	53	303	376
ZIF-7	3.03 mmol CO2 (275)	3.47 mmol CO2 (326)	10	16	59

MOF	cyclohexane uptake [mg g^{-1}] (293 K)		
	p p_0^{-1} = 0.02	p p_0^{-1} = 0.05	p p_0^{-1} = 0.08
Alfum (Basolite® A520)	170	212	225
Aluminumfumarate	151	165	171
MIL-160	200	269	285
DUT-4	86	110	127
DUT-5	233	474	509
MIL-53-TDC	158	163	167
MIL-53(Al)	192	200	203
UiO-66	130	169	188
UiO-66-NH_2	212	241	253
UiO-66$(F)_4$	52	65	72
UiO-67	216	422	506
DUT-67	226	365	406
NH_2-MIL-125	208	220	226
MIL-125	295	315	321
MIL-101(Cr)	145	450	627
ZIF-8	7	13	17
ZIF-11	19	33	45
ZIF-7	5	7	9

Table S7 *n-Hexane sorption results at different relative pressures, including BET-surface areas before and after n-hexane sorption.*

MOF	BET-Surface [m² g^{-1}]		*n*-Hexane uptake [mg g^{-1}] (293 K)		
	before *n*-hexane sorption	after *n*-hexane sorption	p p_0^{-1} = 0.1	p p_0^{-1} = 0.3	p p_0^{-1} = 0.9
Alfum (Basolite® A520)	988	944	240	276	438
Aluminumfumarate	1035	1010	209	225	310
MIL-160	1161	1178	226	237	397
DUT-4	1764	1076	236	286	677
DUT-5	1323	1418	400	472	997
MIL-53-TDC	1015	997	197	210	246
MIL-53(Al)	1325	958	217	323	901
UiO-66	1178	1355	313	326	654
UiO-66-NH_2	1127	1010	240	263	543

UiO-66(F)$_4$	346	290	68	84	207
UiO-67	2317	396	327	351	527
DUT-67	1178	992	209	220	395
NH_2-MIL-125	1570	1204	266	277	323
MIL-125	1425	1455	302	320	569
MIL-101(Cr)	2002	2159	410	438	663
ZIF-8	1615	2340	233	249	395
ZIF-11	491	418	182	203	236
ZIF-7	3.03 mmol CO2 (275)	2.85 mmol CO2 (215)	39	77	96

**MOF**	***n*-Hexane uptake [mg g^{-1}] (293 K)**		
	**p p_0^{-1} = 0.02**	**p p_0^{-1} = 0.05**	**p p_0^{-1} = 0.08**
Alfum (Basolite® A520)	208	224	233
Aluminumfumarate	191	200	205
MIL-160	212	220	223
DUT-4	189	215	228
DUT-5	330	373	390
MIL-53-TDC	176	187	193
MIL-53(Al)	115	174	198
UiO-66	210	230	251
UiO-66-NH_2	191	228	236
UiO-66(F)$_4$	43	56	62
UiO-67	225	296	323
DUT-67	188	202	206
NH_2-MIL-125	250	259	263
MIL-125	282	293	299
MIL-101(Cr)	200	304	377
ZIF-8	171	213	228
ZIF-11	149	174	186
ZIF-7	2	6	21

Table S8 *Relative pressure calculated into absolute pressure in torr and kPa with the different saturation pressures of the VOCs.*

	**benzene**		**cyclohexane**		***n*-hexane**	
	**p_0= 75.26 Torr**	**p_0= 10.03 kPa**	**p_0= 77.51 Torr**	**p_0= 10.33 kPa**	**p_0= 121.53 Torr**	**p_0= 16.20 kPa**
**p p_0^{-1}**	**p [Torr]**	**p [kPa]**	**p [Torr]**	**p [kPa]**	**p [Torr]**	**p [kPa]**
0.02	1.50	0.20	1.55	0.21	2.43	0.32
0.05	3.76	0.50	3.88	0.52	6.08	0.81
0.08	6.02	0.80	6.20	0.83	9.72	1.30
0.1	7.53	1.00	7.75	1.03	12.15	1.62

0.3	22.58	3.00	23.25	3.10	36.46	4.86
0.9	67.73	9.00	69.76	9.30	109.38	14.58

Order of the VOC affinity of each MOF at different relative pressures.

Table S9 *Order of the volatile organic compounds at two different relative pressures (0.1 and 0.9) (293 K).*

**MOF**	**VOC-Order @ 0.1 p p_0^{-1}**	**VOC-Order @ 0.9 p p_0^{-1}**
Alfum (Basolite® A520)	**B > n-H ≈ C**	**B > C > n-H**
Aluminumfumarate	**B > n-H > C**	**B > n-H > C**
MIL-160	**B > C > n-H**	**B > n-H ≈ C**
DUT-4	**B > n-H > C**	**B > C ≈ n-H**
DUT-5	**C > B > n-H**	**n-H > B > C**
MIL-53-TDC	**B > n-H > C**	**B > n-H > C**
MIL-53(Al)	**B > n-H ≈ C**	**n-H > B > C**
UiO-66	**B > n-H > C**	**C ≈ n-H > B**
UiO-66-NH_2	**C > n-H > B**	**n-H > B ≈ C**
UiO-66(F)$_4$	**B > C ≈ n-H**	**B > C > n-H**
UiO-67	**C > B > n-H**	**B > C > n-H**
DUT-67	**B ≈ C > n-H**	**C ≈ B > n-H**
NH_2-MIL-125	**n-H > C > B**	**n-H > C > B**
MIL-125	**C > n-H > B**	**B > C > n-H**
MIL-101(Cr)	**C > B > n-H**	**C > B > n-H**
ZIF-8	**n-H > B > C**	**B > n-H > C**
ZIF-11	**B > n-H > C**	**C > B > n-H**
ZIF-7	**n-H > B > C**	**n-H > B > C**

Different orders are highlighted by color code for clarity:

B > n-H > C   **B > C > n-H**

C > B > n-H   **C > n-H > B**

n-H > B > C   **n-H > C > B**

Vapor sorption isotherms divided by metals: full isotherms and low relative pressure

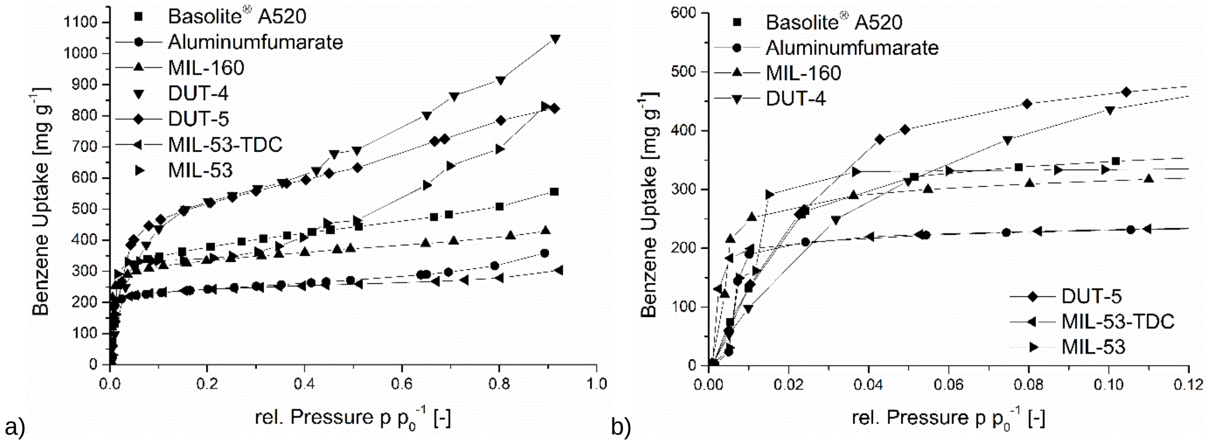

Figure S47 *Al-MOFs with benzene vapor isotherms 0-1 p p_0^{-1} (a) and 0-0.12 p p_0^{-1} (b) (293 K).*

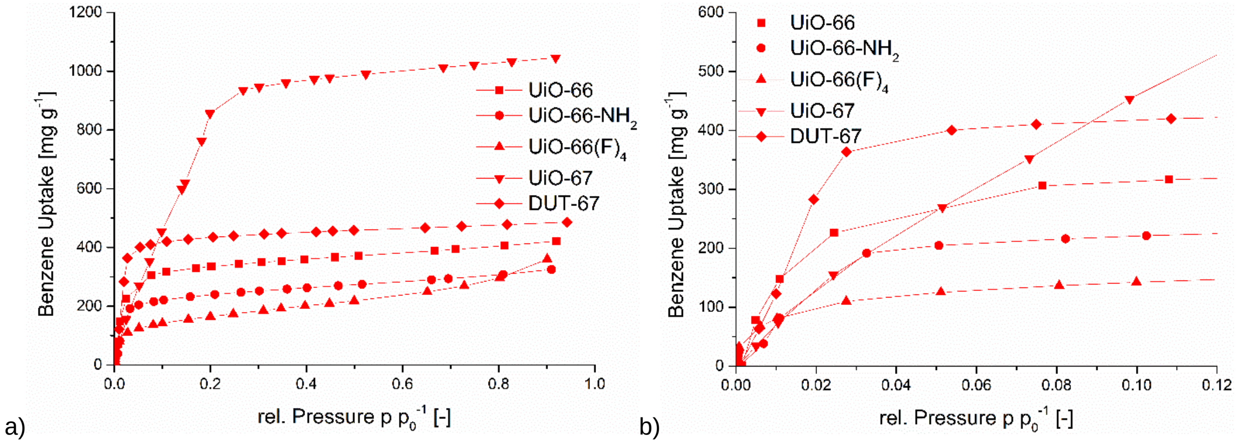

Figure S48 *Zr-MOFs with benzene vapor isotherms 0-1 p p_0^{-1} (a) and 0-0.12 p p_0^{-1} (b) (293 K).*

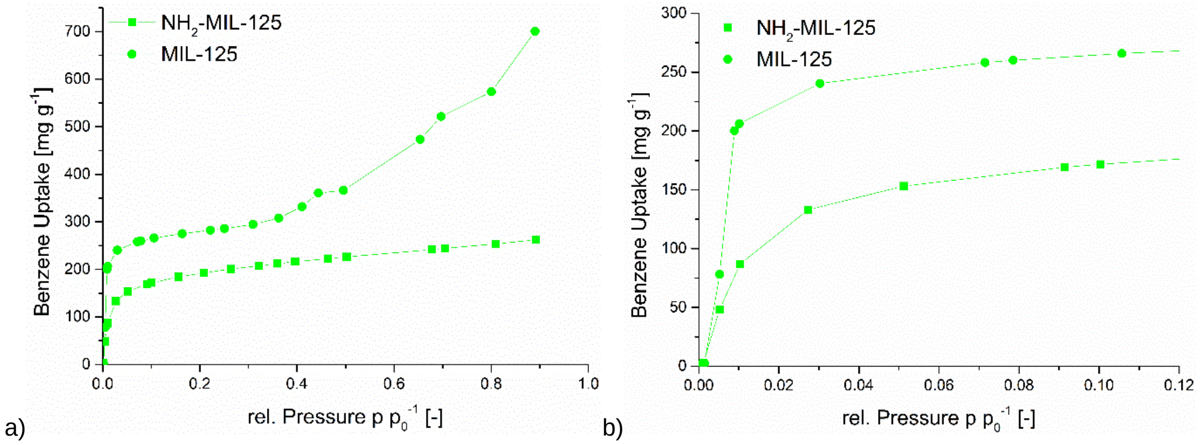

Figure S49 *Ti-MOFs with benzene vapor isotherms 0-1 p p_0^{-1} (a) and 0-0.12 p p_0^{-1} (b) (293 K).*

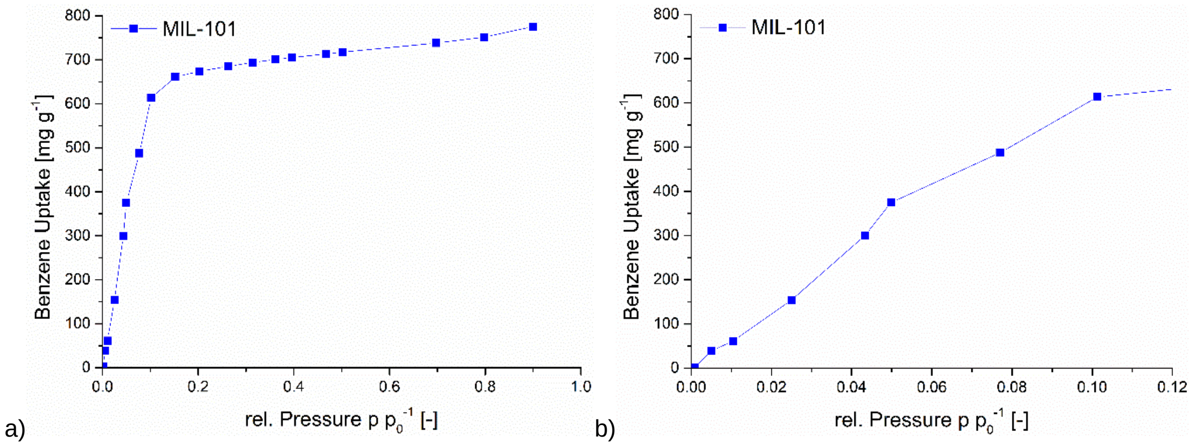

Figure S50 *Cr-MOF with benzene vapor isotherms 0-1 p p_0^{-1} (a) and 0-0.12 p p_0^{-1} (b) (293 K).*

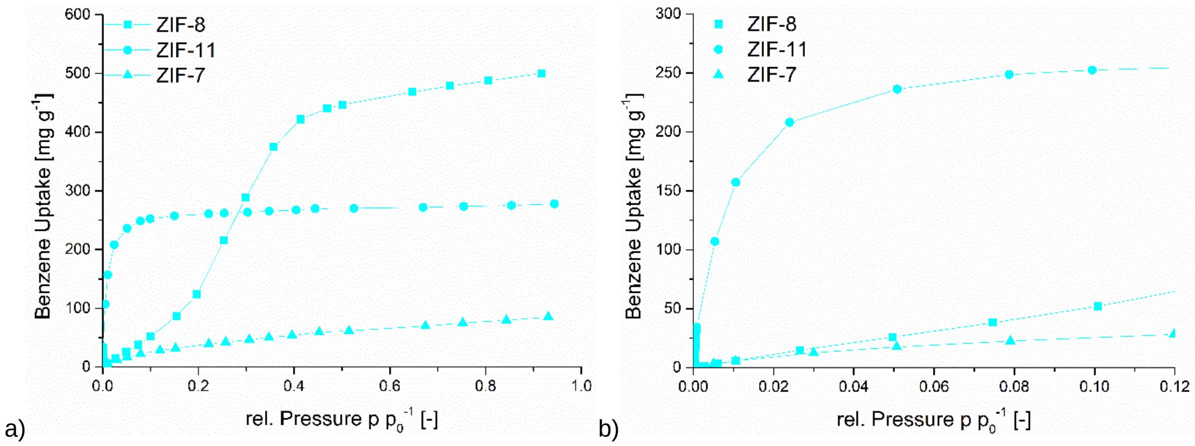

Figure S51 *ZIFs with benzene vapor isotherms 0-1 p p_0^{-1} (a) and 0-0.12 p p_0^{-1} (b) (293 K).*

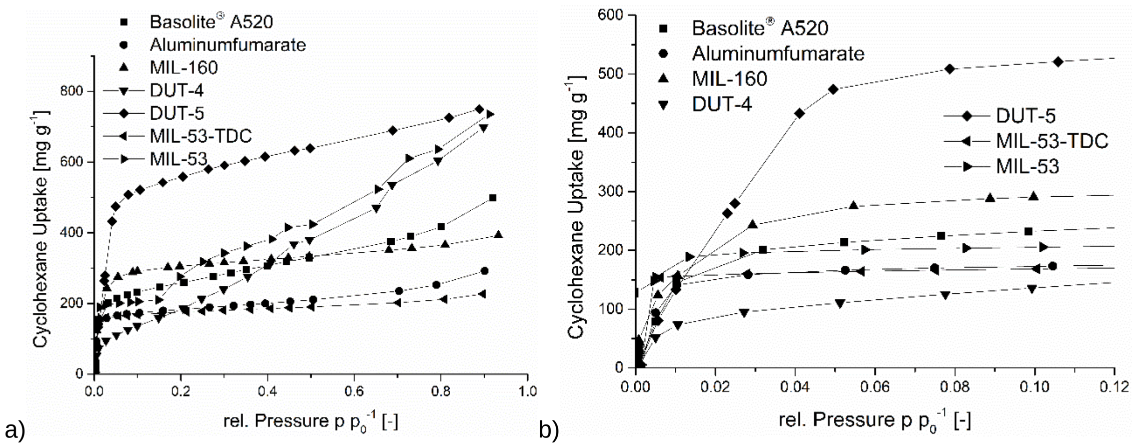

Figure S52 *Al-MOFs with cyclohexane vapor isotherms 0-1 p p_0^{-1} (a) and 0-0.12 p p_0^{-1} (b) (293 K).*

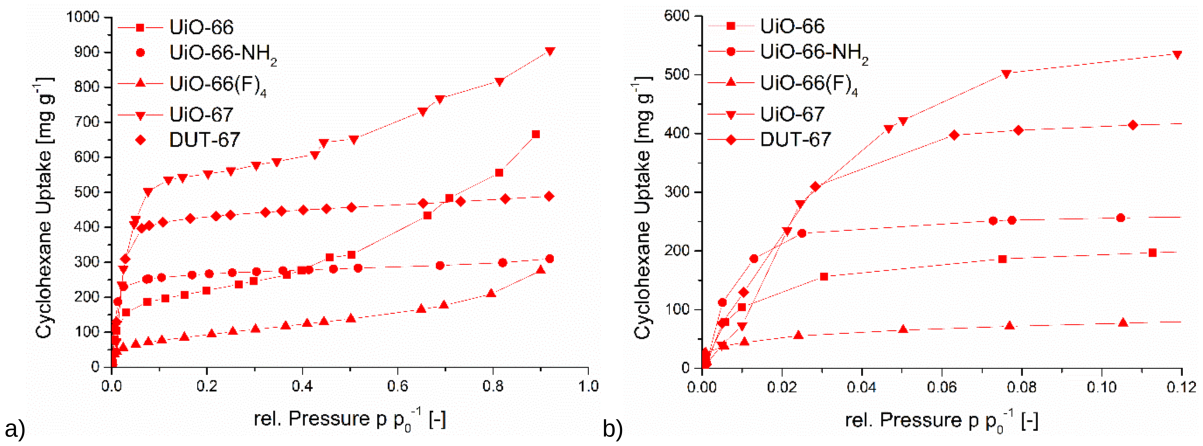

Figure S53 *Zr-MOFs with cyclohexane vapor isotherms 0-1 p p_0^{-1} (a) and 0-0.12 p p_0^{-1} (b) (293 K).*

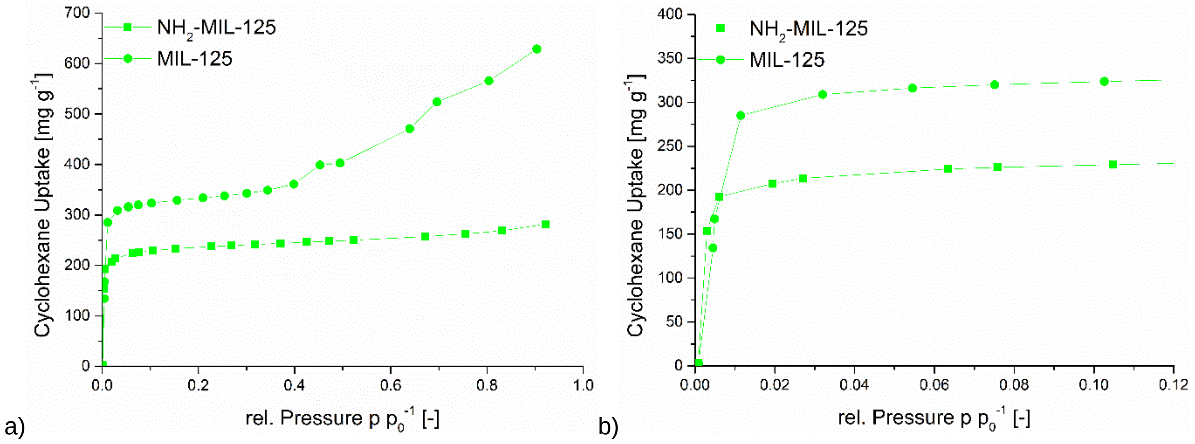

Figure S54 *Ti-MOFs with cyclohexane vapor isotherms 0-1 p p_0^{-1} (a) and 0-0.12 p p_0^{-1} (b) (293 K).*

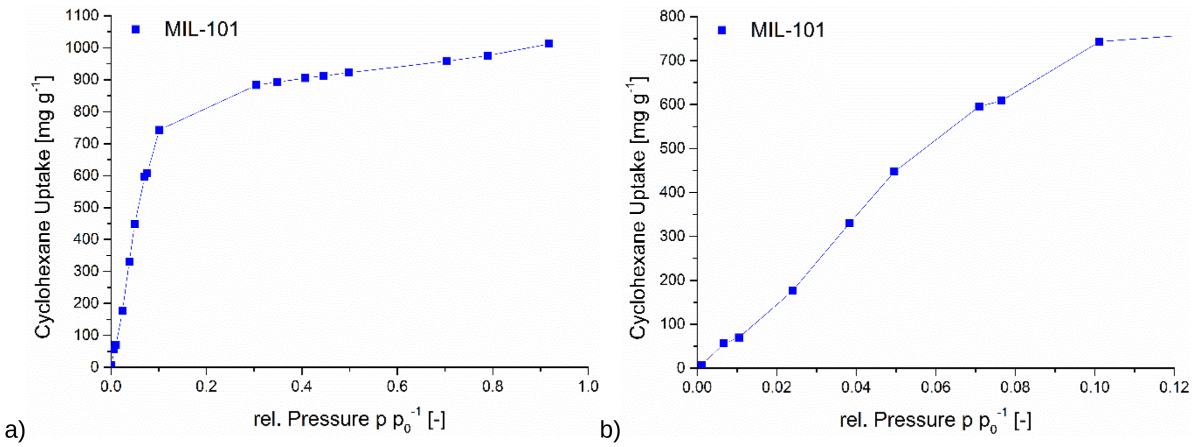

Figure S55 *Cr-MOF with cyclohexane vapor isotherms 0-1 p p_0^{-1} (a) and 0-0.12 p p_0^{-1} (b) (293 K).*

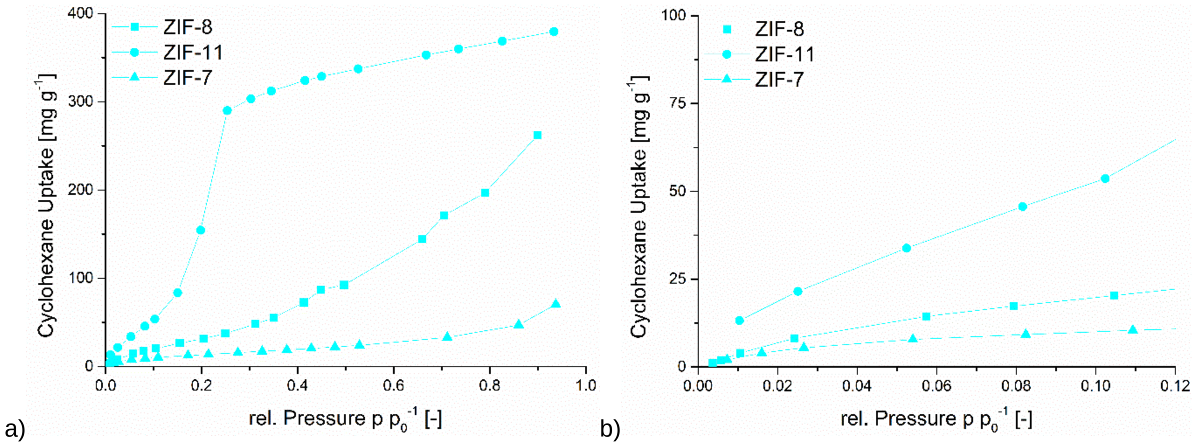

Figure S56 *ZIFs with cyclohexane vapor isotherms 0-1 p* p_0^{-1} *(a) and 0-0.12 p* p_0^{-1} *(b) (293 K).*

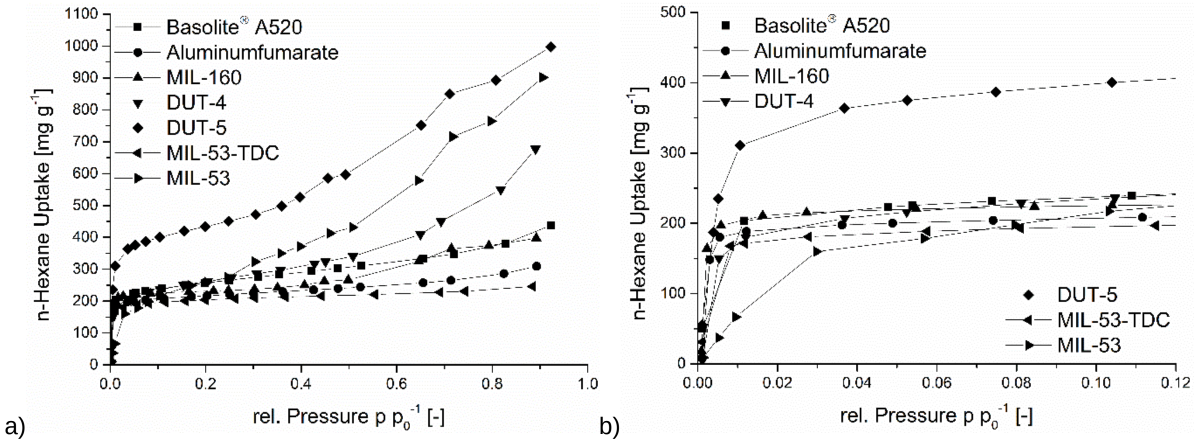

Figure S57 *Al-MOFs with n-hexane vapor isotherms 0-1 p* p_0^{-1} *(a) and 0-0.12 p* p_0^{-1} *(b) (293 K).*

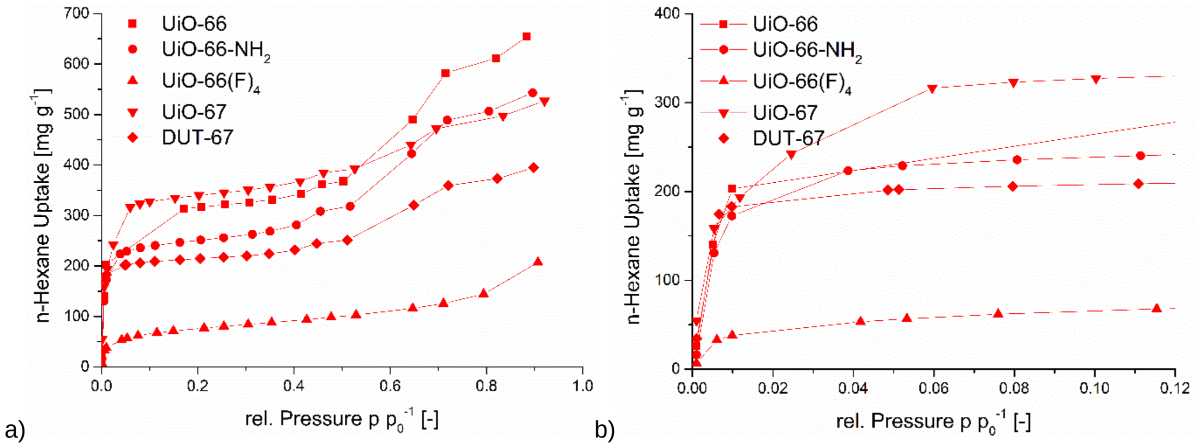

Figure S58 *Zr-MOFs with n-hexane vapor isotherms 0-1 p* p_0^{-1} *(a) and 0-0.12 p* p_0^{-1} *(b) (293 K).*

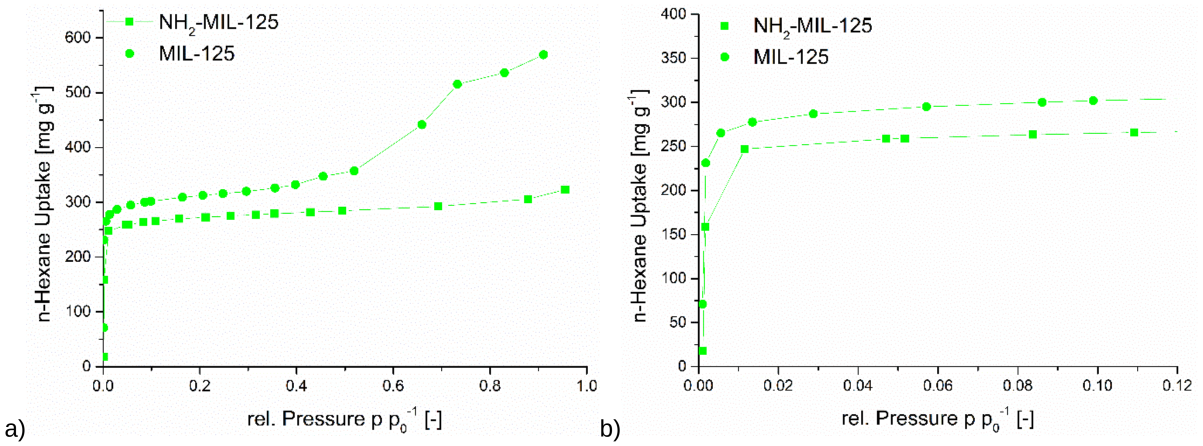

Figure S59 *Ti-MOFs with n-hexane vapor isotherms 0-1 p p_0^{-1} (a) and 0-0.12 p p_0^{-1} (b) (293 K).*

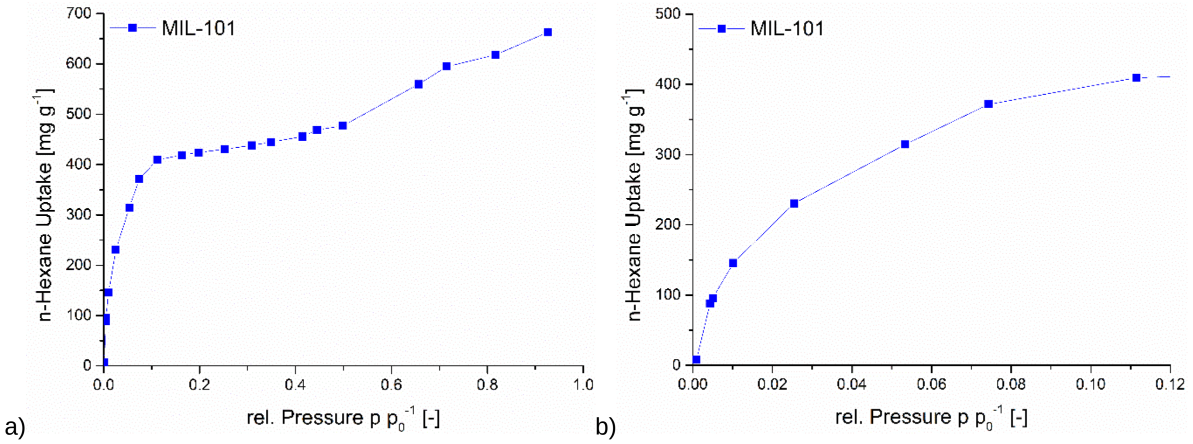

Figure S60 *Cr-MOF with n-hexane vapor isotherms 0-1 p p_0^{-1} (a) and 0-0.12 p p_0^{-1} (b) (293 K).*

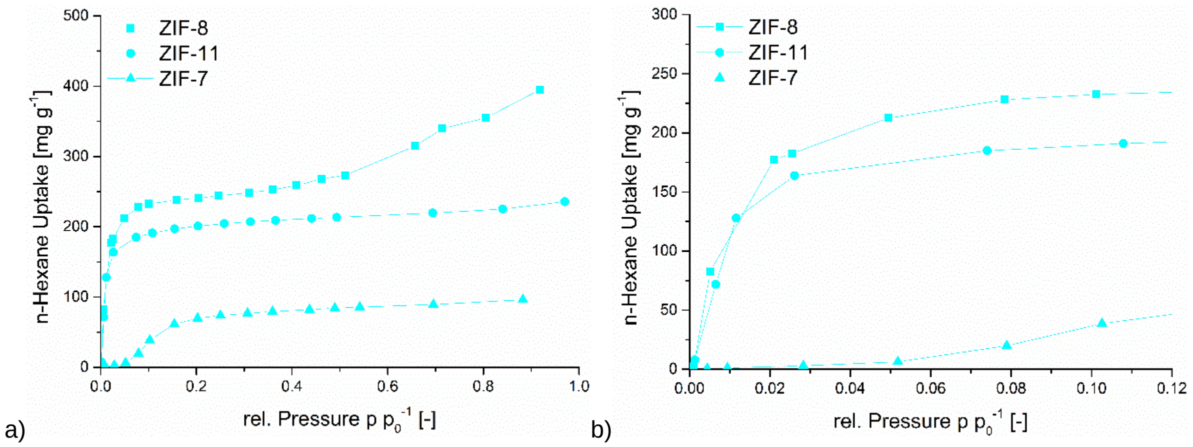

Figure S61 *ZIFs with n-hexane vapor isotherms 0-1 p p_0^{-1} (a) and 0-0.12 p p_0^{-1} (b) (293 K).*

S7 Thermogravimetric analysis (TGA)

Al-MOFs

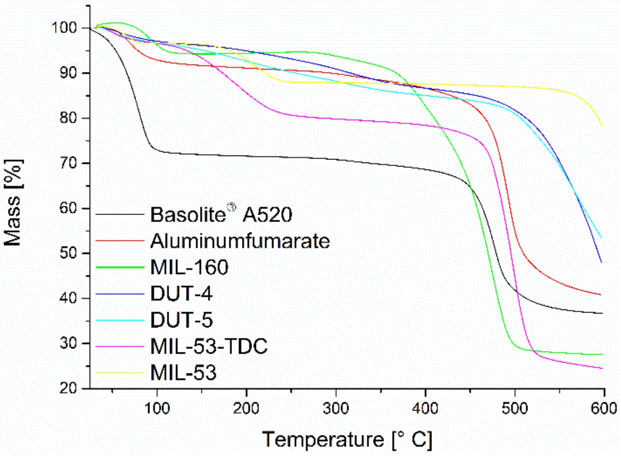

Figure S62 *TGA curves for the Al-MOFs under nitrogen atmosphere.*

Zr-MOFs

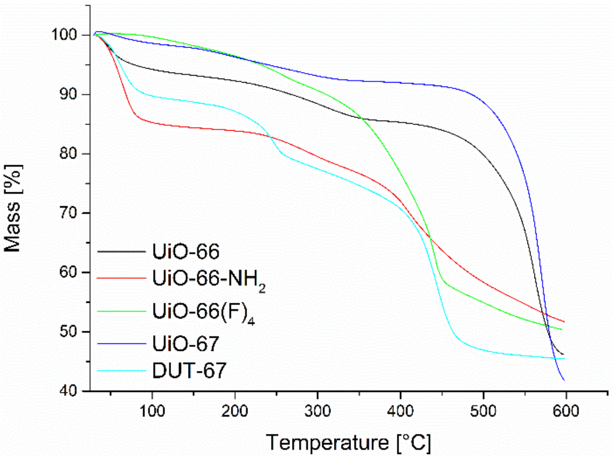

Figure S63 *TGA curves for the Zr-MOFs under nitrogen atmosphere.*

Ti-MOFs

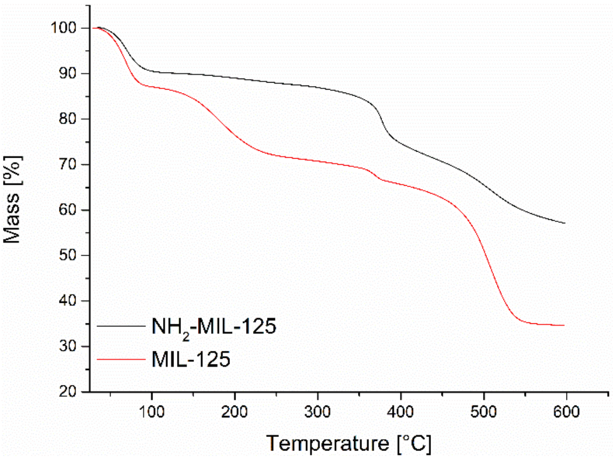

Figure S64 *TGA curves for the Ti-MOFs under nitrogen atmosphere.*

Cr-MOF

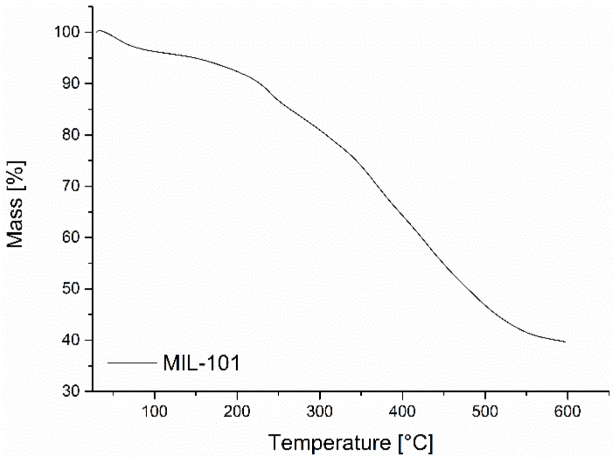

Figure S65 *TGA curve for the Cr-MOF under nitrogen atmosphere.*

ZIFs

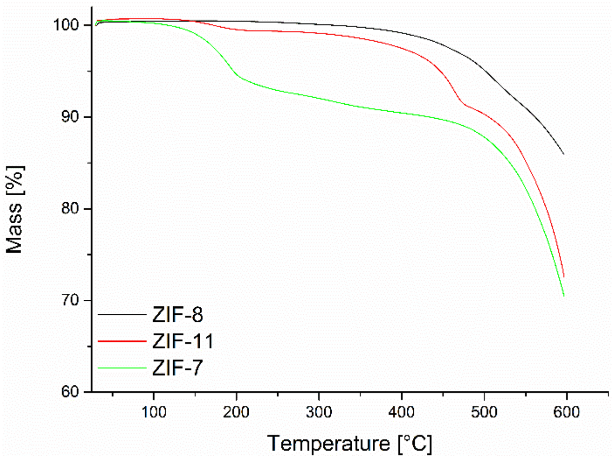

Figure S66 *TGA curves for the ZIFs under nitrogen atmosphere.*

S8 Scanning electron microscopy (SEM)

Al-MOFs

Basolite® A520

a) b)

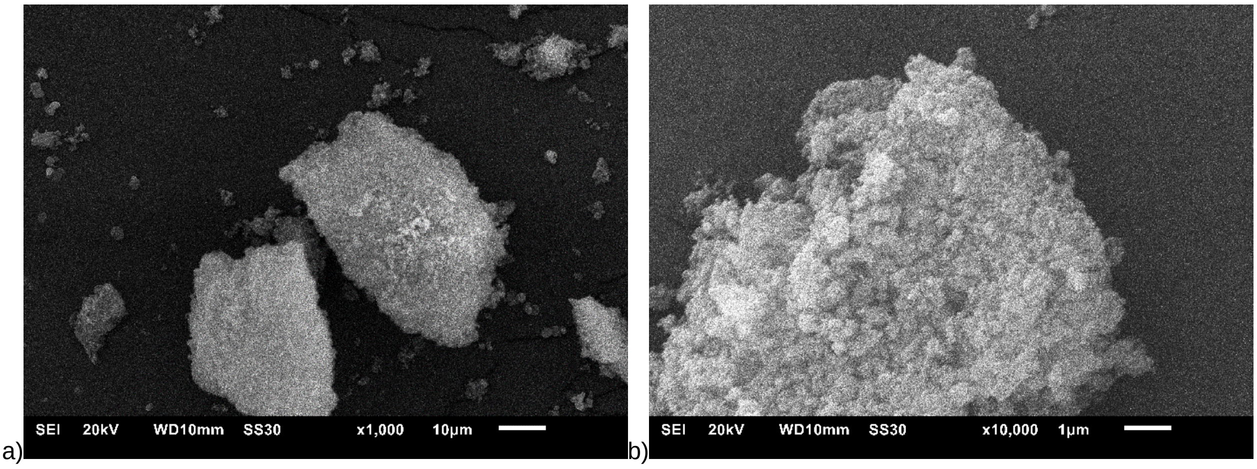

Figure S67 *SEM images of Basolite® A520 at different magnifications ((a) overview, (b) close-up).*

Aluminumfumarate

a) b)

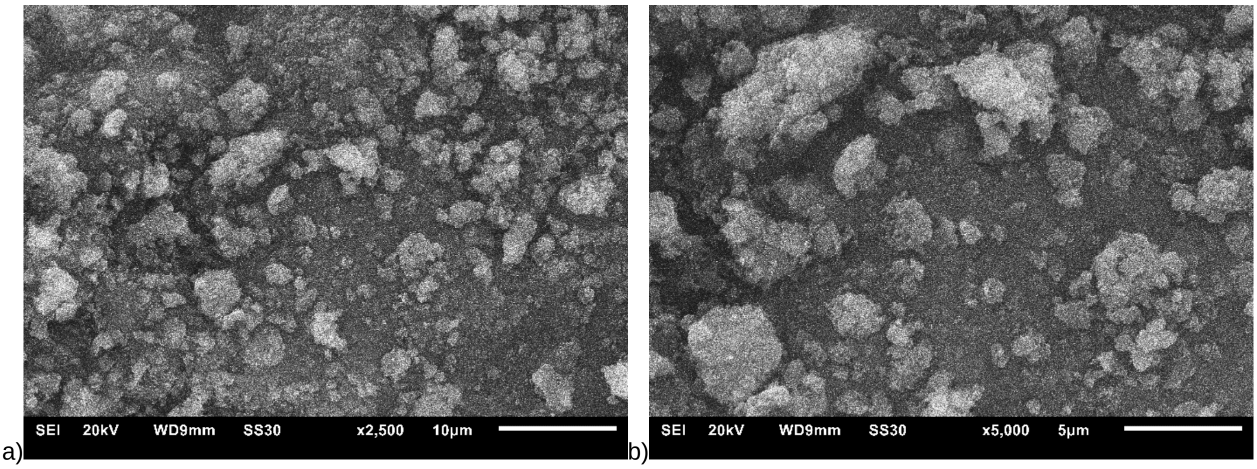

Figure S68 *SEM images of Aluminumfumarate at different magnifications ((a) overview, (b) close-up).*

MIL-160

a) b)

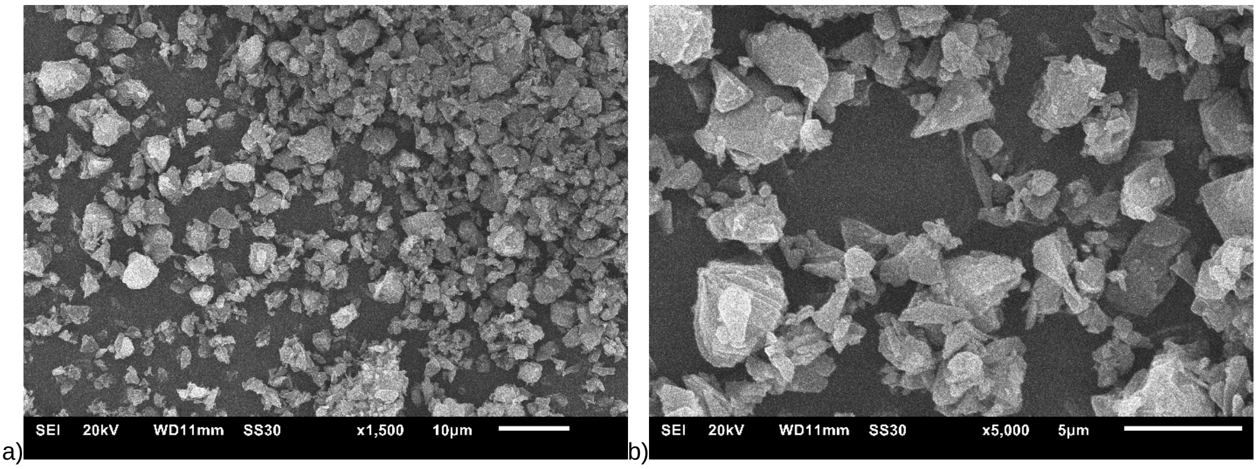

Figure S69 *SEM images of MIL-160 at different magnifications ((a) overview, (b) close-up).*

DUT-4

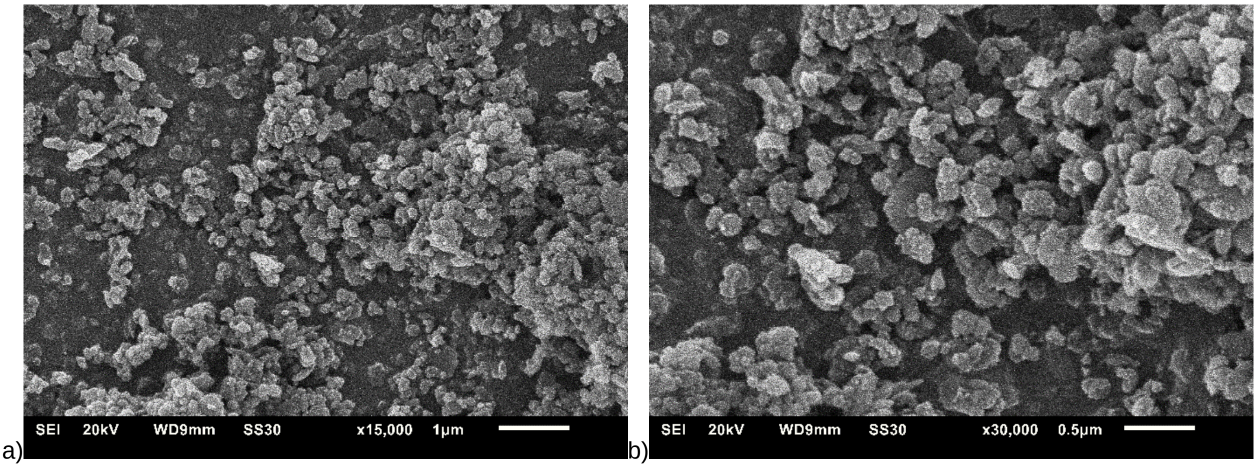

a) b)

Figure S70 *SEM images of DUT-4 at different magnifications ((a) overview, (b) close-up).*

DUT-5

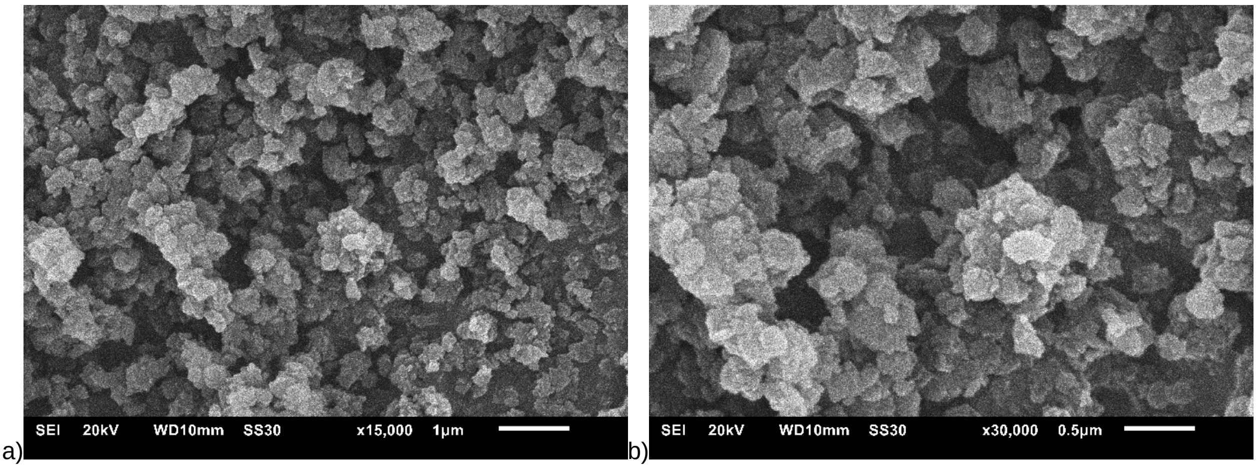

a) b)

Figure S71 *SEM images of DUT-5 at different magnifications ((a) overview, (b) close-up).*

MIL-53-TDC

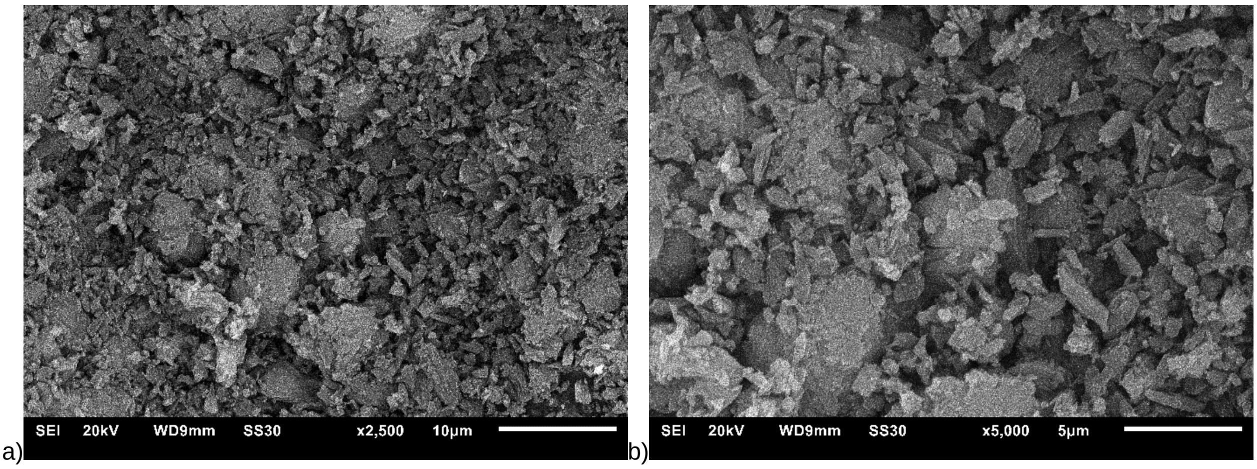

a) b)

Figure S72 *SEM images of MIL-53-TDC at different magnifications ((a) overview, (b) close-up).*

MIL-53

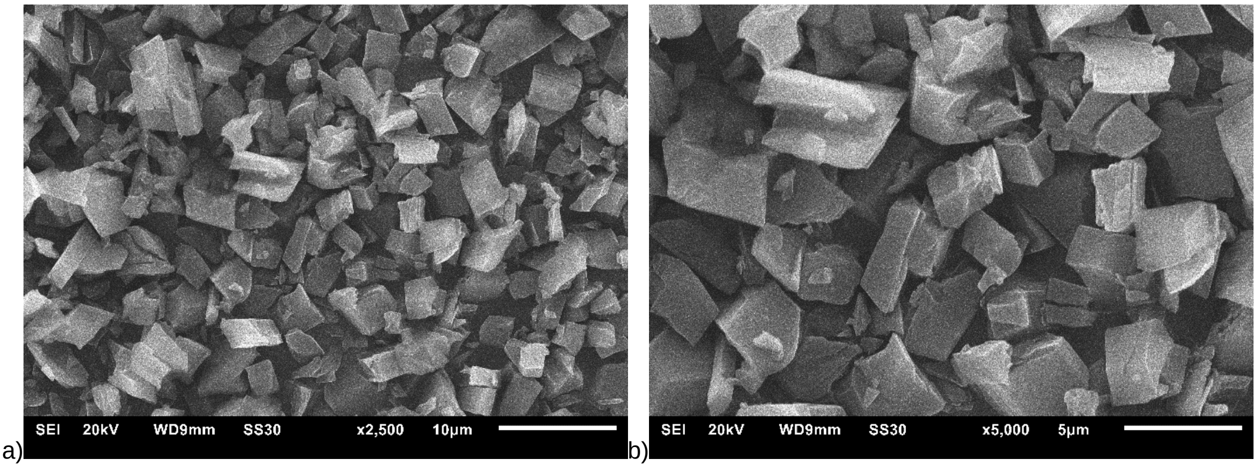

a) b)

Figure S73 *SEM images of MIL-53 at different magnifications ((a) overview, (b) close-up).*

Zr-MOFs

UiO-66

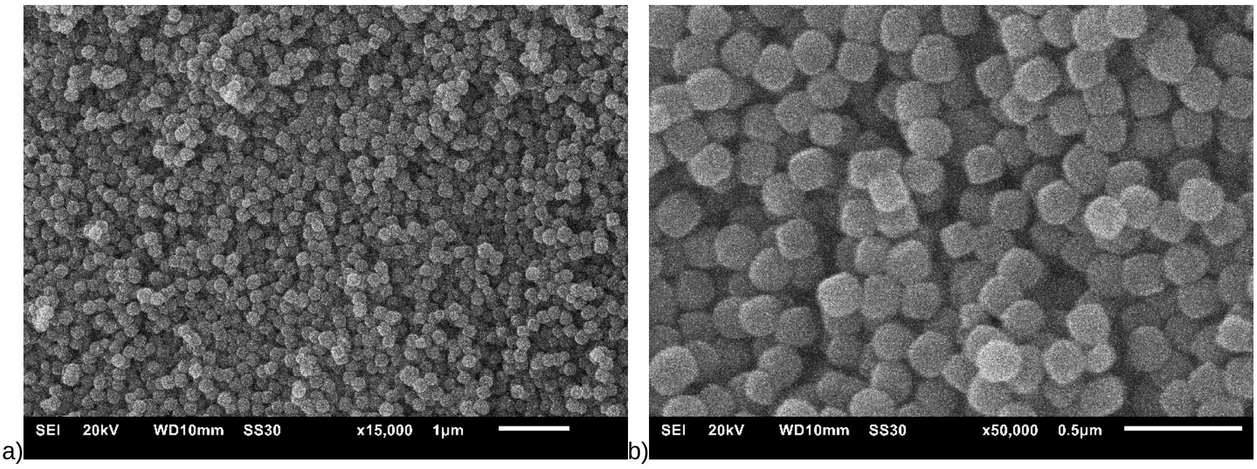

a) b)

Figure S74 *SEM images of UiO-66 at different magnifications ((a) overview, (b) close-up).*

UiO-66-NH_2

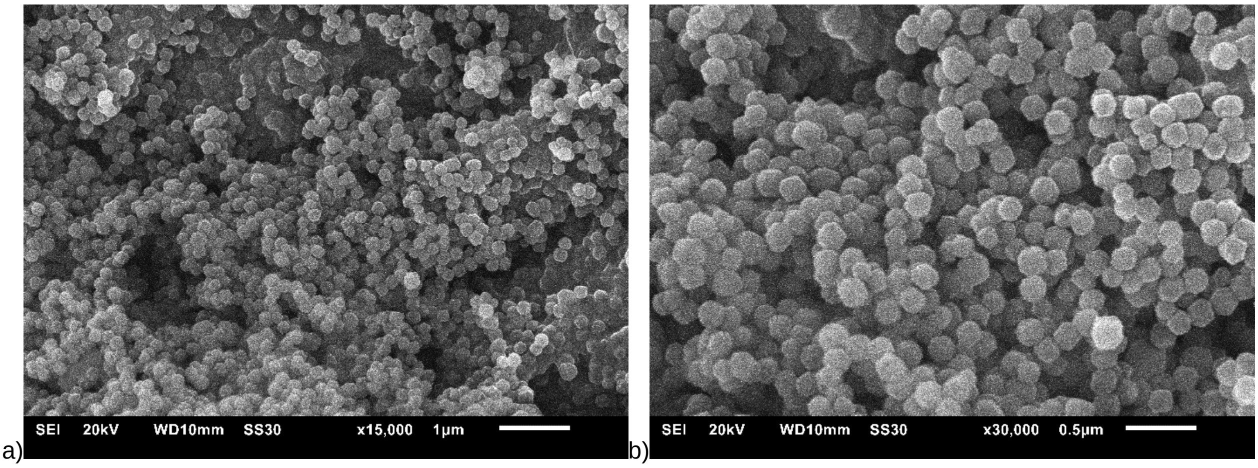

a) b)

Figure S75 *SEM images of UiO-66-NH_2 at different magnifications ((a) overview, (b) close-up).*

UiO-66(F)$_4$

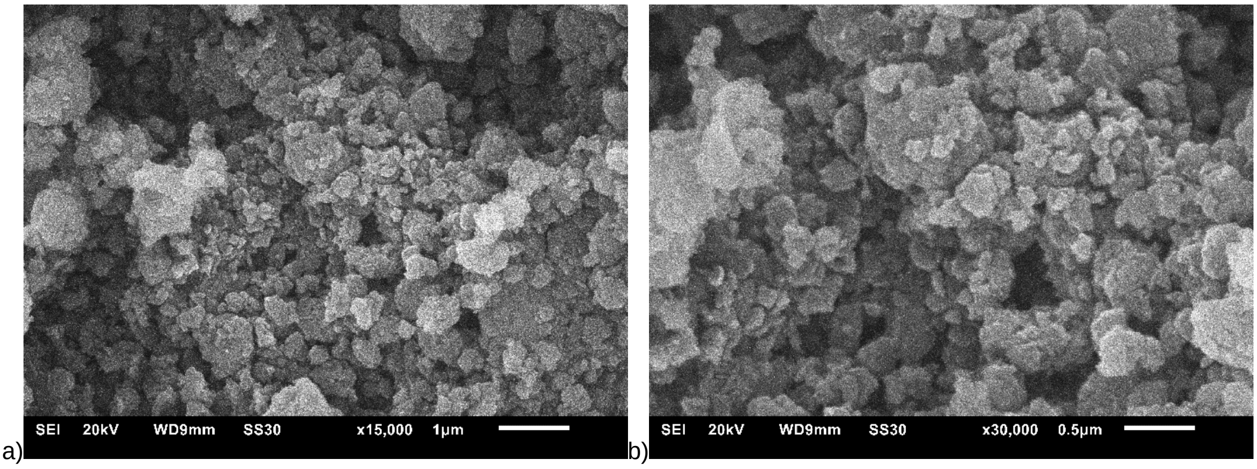

Figure S76 *SEM images of UiO-66(F)$_4$ at different magnifications ((a) overview, (b) close-up).*

UiO-67

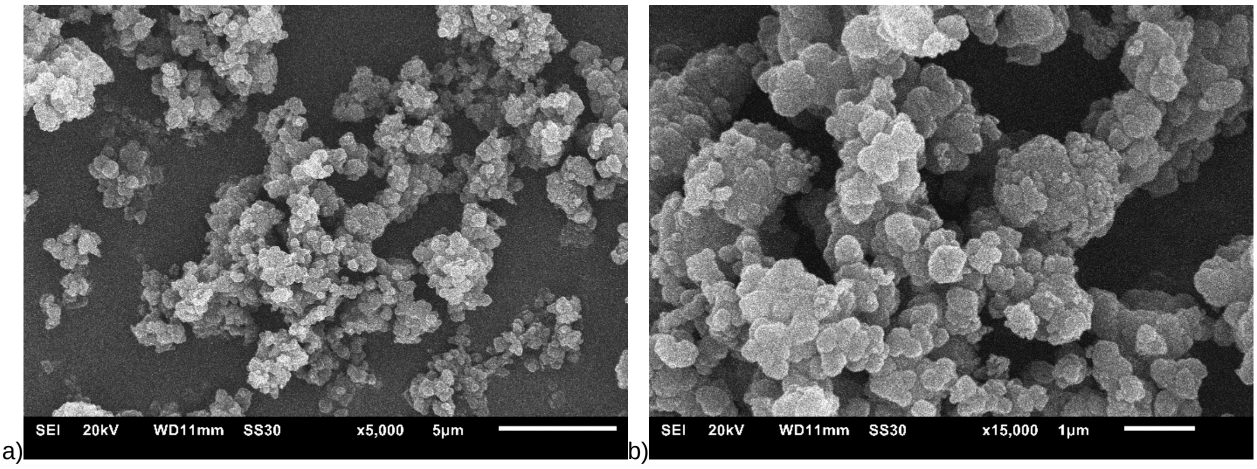

Figure S77 *SEM images of UiO-67 at different magnifications ((a) overview, (b) close-up).*

DUT-67

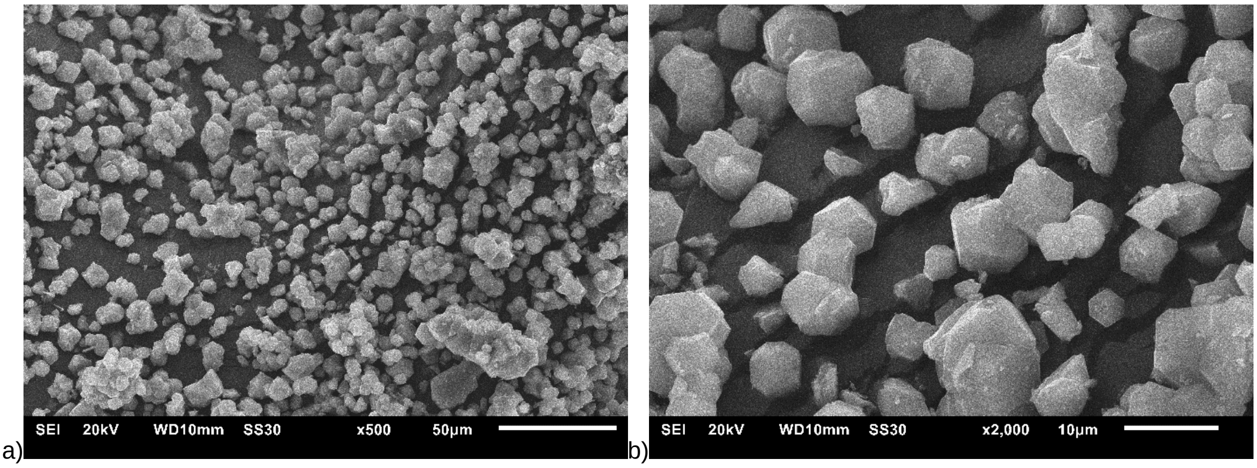

Figure S78 *SEM images of DUT-67 at different magnifications ((a) overview, (b) close-up).*

Ti-MOFs

NH$_2$-MIL-125

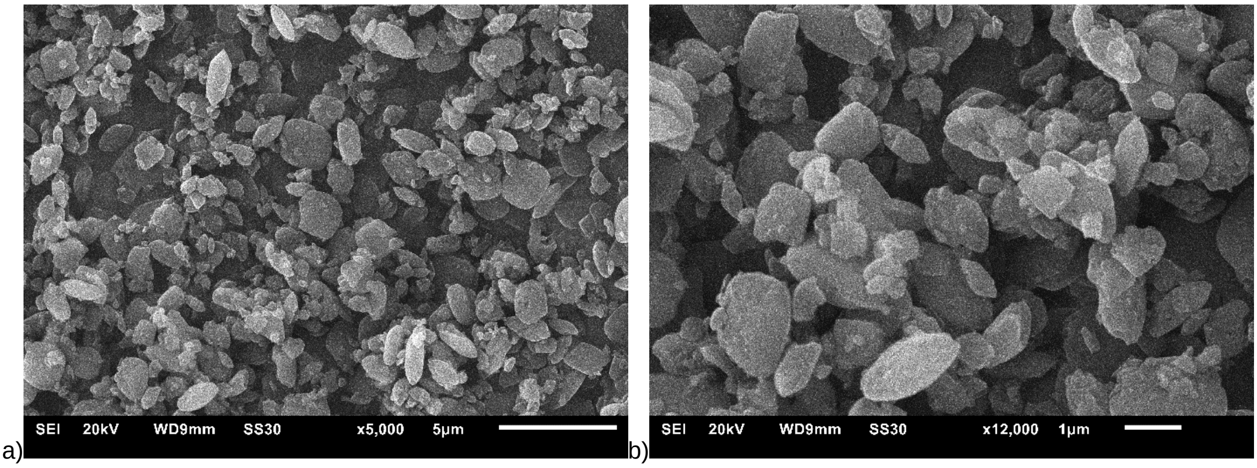

Figure S79 *SEM images of NH$_2$-MIL-125 at different magnifications ((a) overview, (b) close-up).*

MIL-125

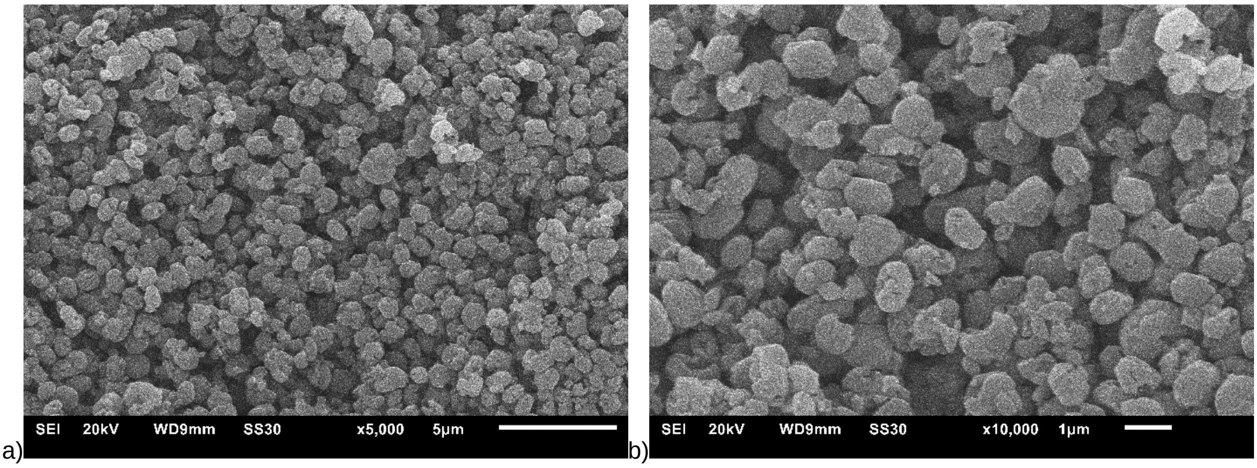

Figure S80 *SEM images of MIL-125 at different magnifications ((a) overview, (b) close-up).*

Cr-MOF

MIL-101(Cr)

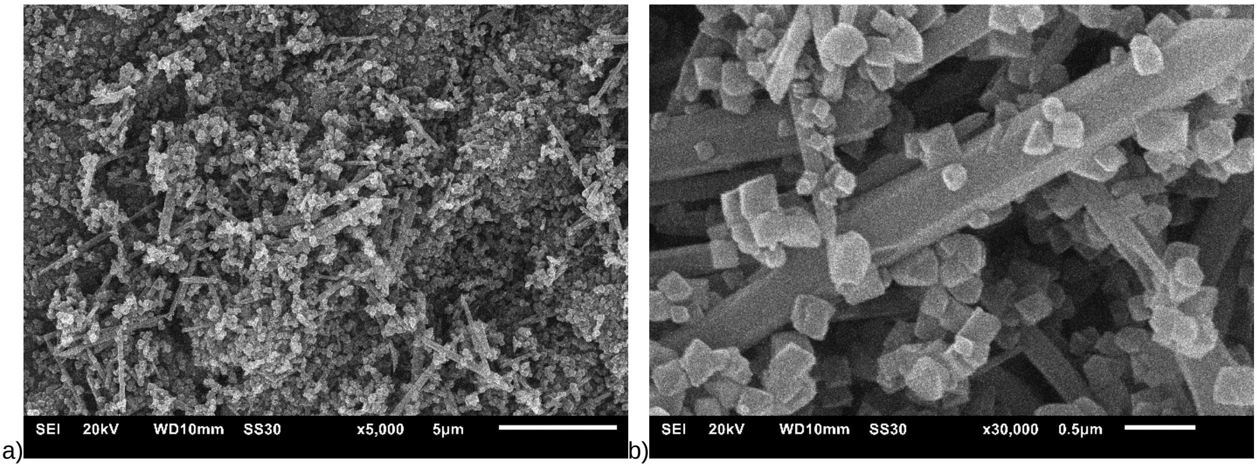

Figure S81 *SEM images of MIL-101(Cr) at different magnifications ((a) overview, (b) close-up).*

ZIFs

ZIF-8

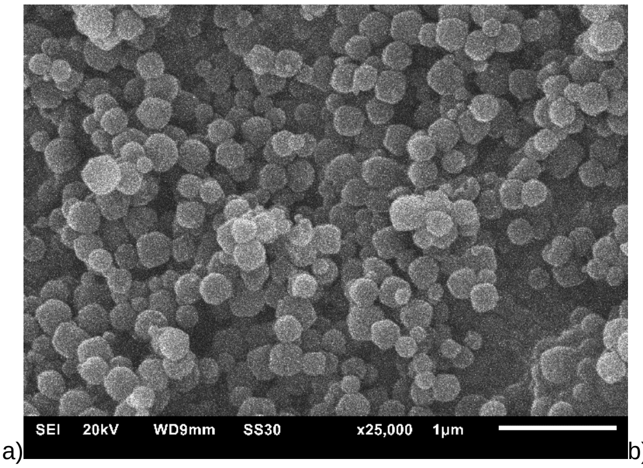

a)

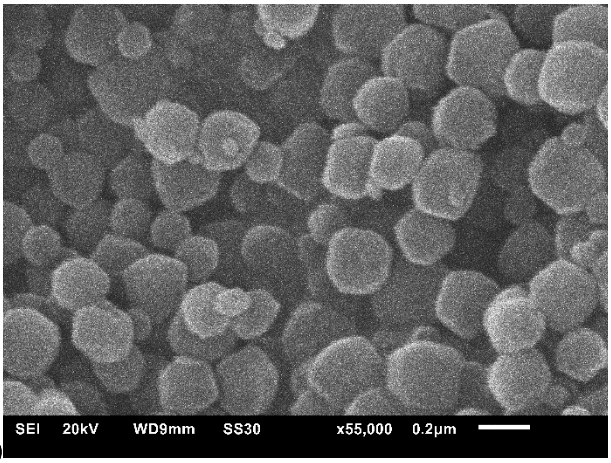

b)

Figure S82 *SEM images of ZIF-8 at different magnifications ((a) overview, (b) close-up).*

ZIF-11

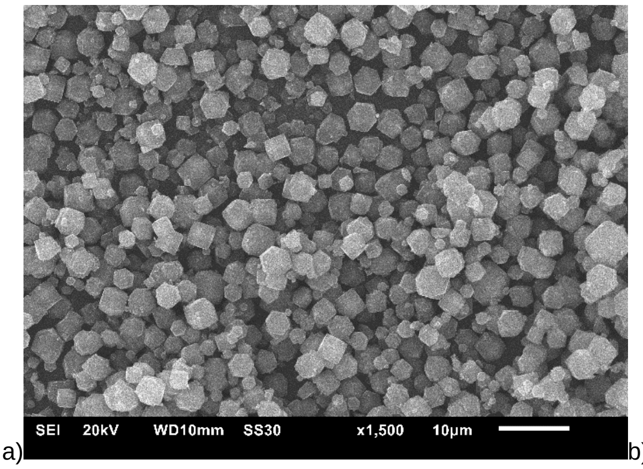

a)

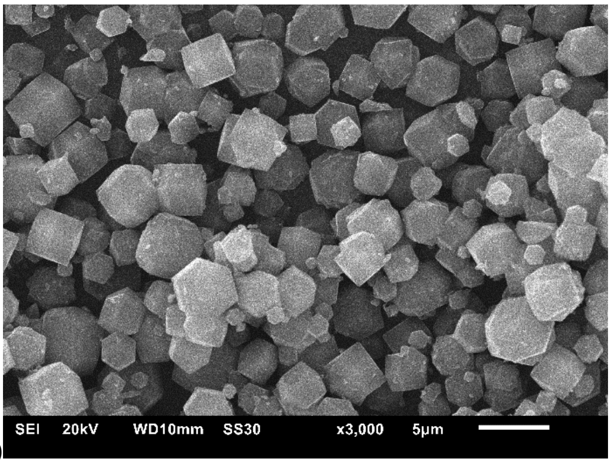

b)

Figure S83 *SEM images of ZIF-11 at different magnifications ((a) overview, (b) close-up).*

ZIF-7

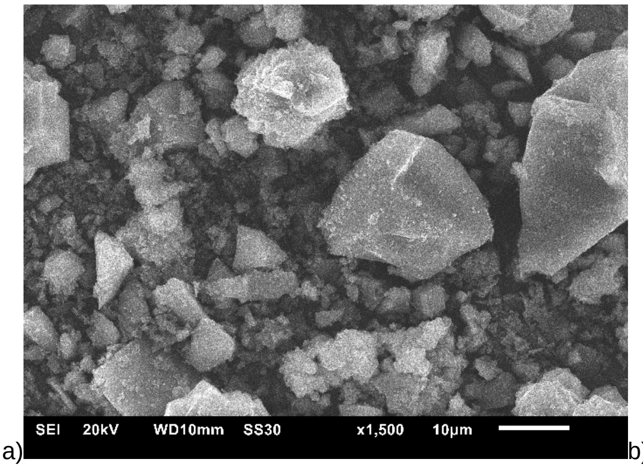

a)

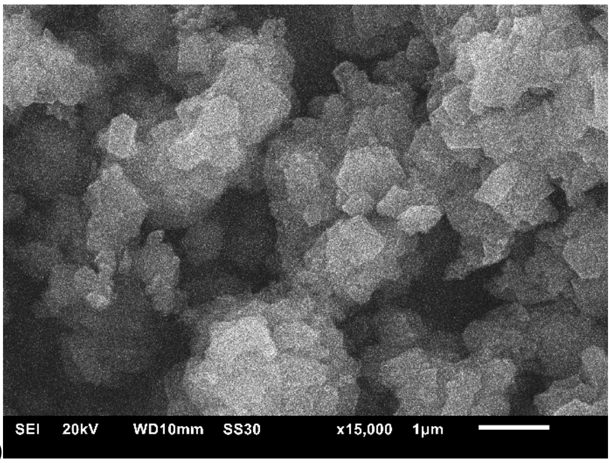

b)

Figure S84 *SEM images of ZIF-7 at different magnifications ((a) overview, (b) close-up).*

S9 VOC uptake versus BET surface, pore window size, micropore volume

To analyze the influence of the MOF microstructure on the sorption capacity different correlations were used. The uptake vs. pore volume correlation is given in Figure 5 in the main text. Here additional correlations of uptake vs. surface area, pore window size and micropore volume are depicted.

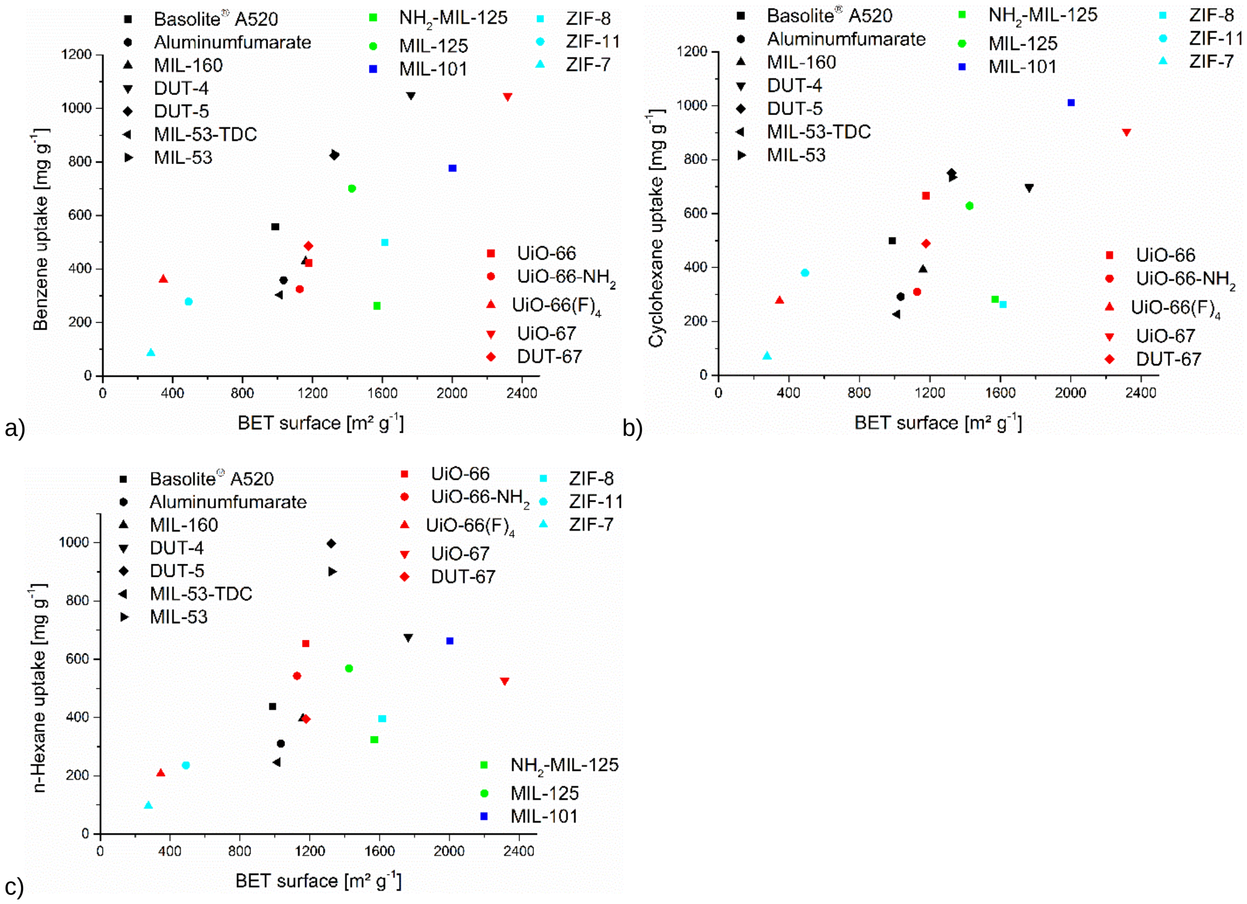

Figure S85 *C_6-VOC uptake at $p\,p_0^{-1}$ = 0.9, 293 K versus BET surface area for (a) benzene, (b) cyclohexane and (c) n-hexane.*

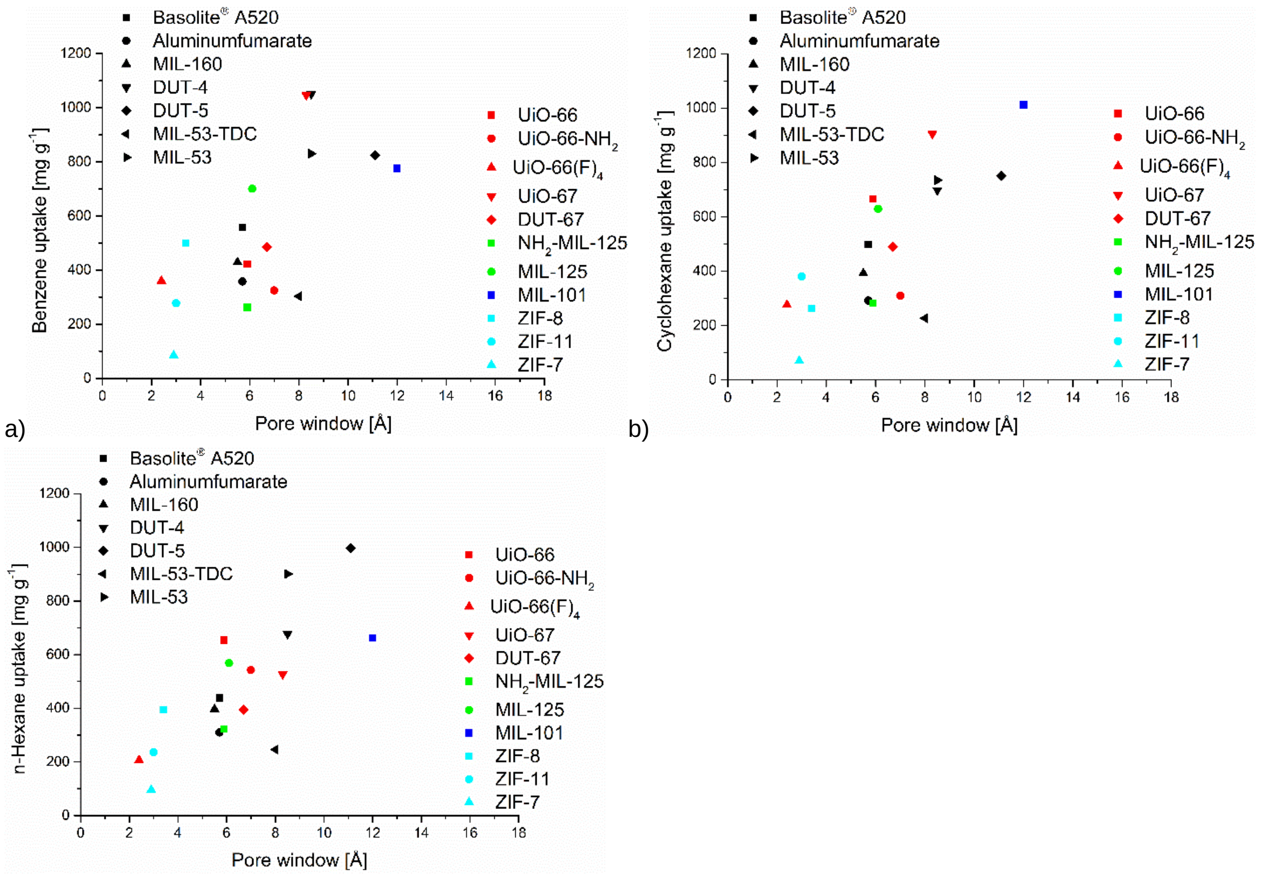

Figure S86 *C_6-VOC uptake at $p\ p_0^{-1}$ = 0.9, 293 K versus Pore window (cf. Table S2) for (a) benzene, (b) cyclohexane, (c) n-hexane.*

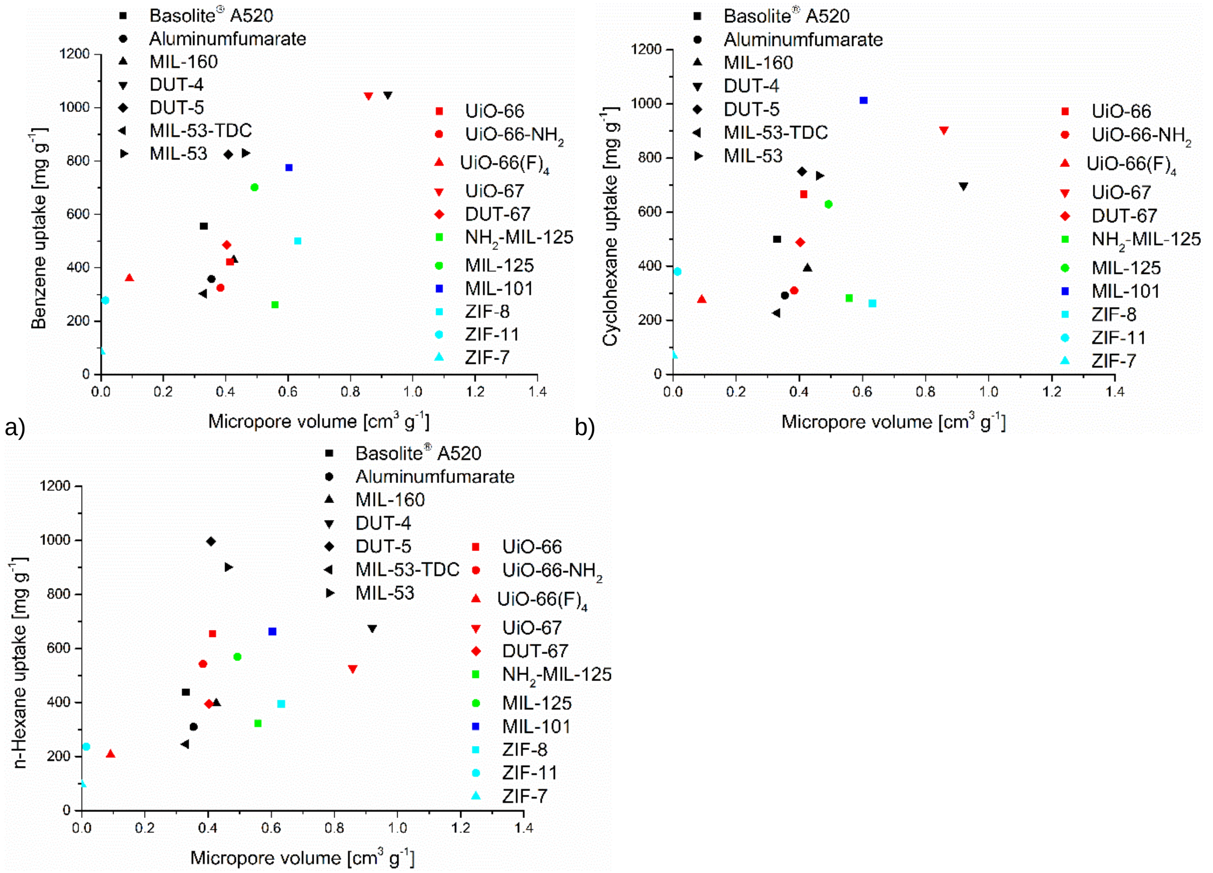

Figure S87 *C_6-VOC uptake at $p\ p_0^{-1}$ = 0.9, 293 K versus Micropore volume (cf. Table S2) for (a) benzene, (b) cyclohexane, (c) n-hexane.*

Surface-specific uptake and pore limiting diameter

Often the adsorption capacity is normalized to the amount adsorbed on per unit area of the MOF adsorbent (see Table S2 for BET surface area).[45,46] This can give useful information regarding the adsorption affinity or a pore limiting diameter (PLD), for example the pore window as the limiting diameter (Table S2). In order to elucidate the adsorption affinity of relevant MOFs for C_6 trace adsorption, we used the uptake at p p_0^{-1} = 0.1 for the normalization (Figure S88).

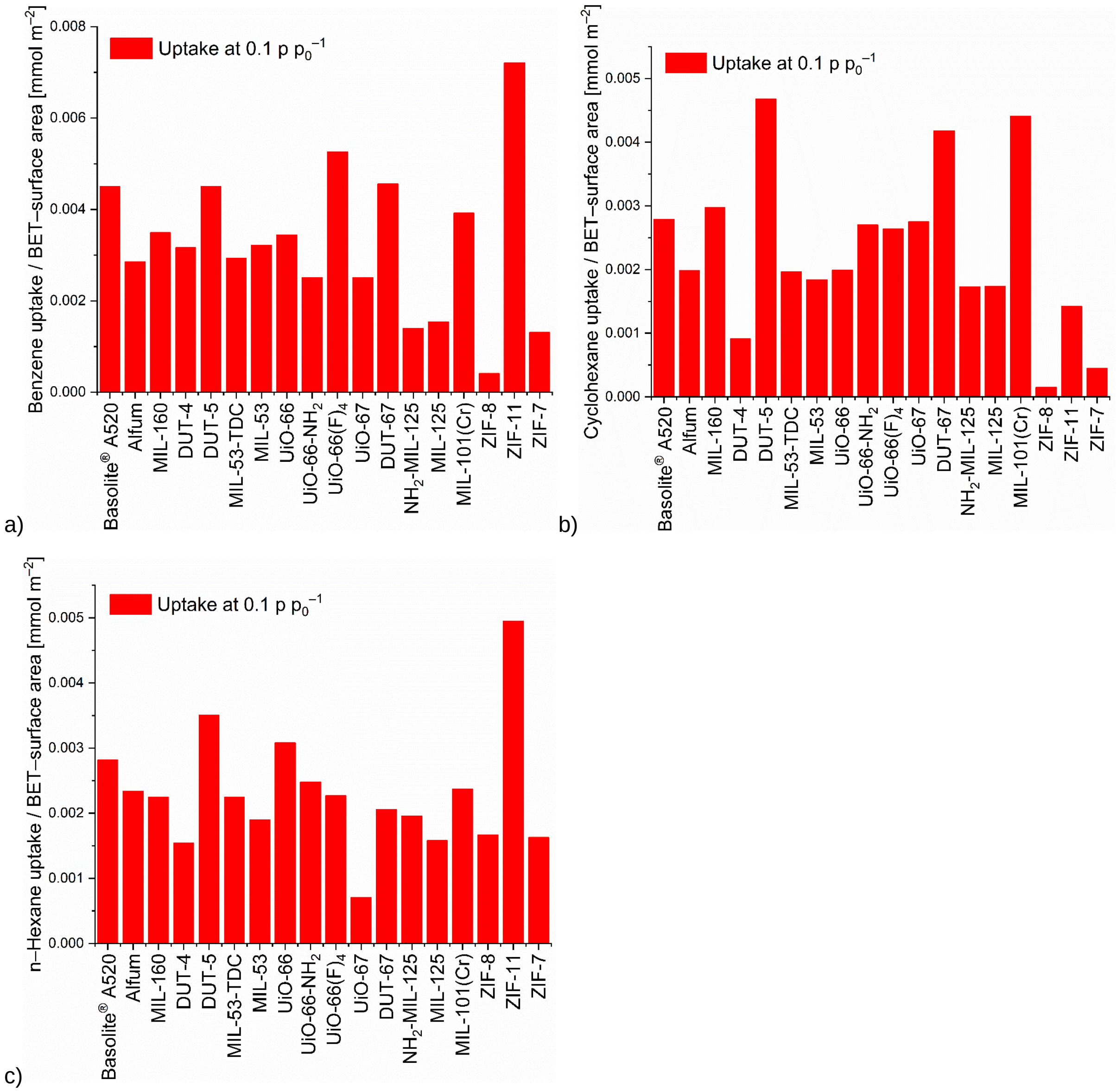

Figure S88 *Surface-specific benzene (a), cyclohexane (b) and n-hexane (c) uptake at p p_0^{-1} = 0.1 (293 K), which is the uptake in mmol g^{-1} at this pressure divided by the BET surface area.*

The surface-normalized, i.e. surface-specific benzene adsorption capacity at p p_0^{-1} = 0.1 (Figure S88) is highest for ZIF-11 and still higher than average for Basolite A520, DUT-5, UiO-66(F)$_4$, DUT-67 and MIL-101(Cr). For cyclohexane the highest surface normalized adsorption is reached by DUT-5, DUT-67 and MIL-101(Cr). For *n*-hexane, ZIF-11 has again and by far the highest surface-specific adsorption, with DUT-5 and UiO-66 still somewhat higher than average.

The high surface-specific uptake values originate from a substantial early uptake *together* with a relatively low surface area. High values for the surface-normalized uptake at p p_0^{-1} = 0.1 may contrast with low absolute specific C_6 uptakes, as is the case for ZIF-11 due to its overall small pore volume, size and surface area. Also, it has to be kept in mind that the surface normalization will not reflect if low uptake is due to inaccessible regions.

A plot of the surface-specific uptake *vs* pore limiting diameter (PLD) at 0.01 and 0.1 p p_0^{-1} shows invariance and does not give a clear maximum (Figure S89). The PLD is the smallest pore (window) diameter in a framework, as *e. g.* Brandt *et al.* described in 2021 and correlated the optimum SO_2 sorption capacity of their MOFs with the molecular size of SO_2.[47] At an optimal PLD the adsorptive would have dispersive interactions with several positions ("both ends, both sides") of the molecule to the surface. Local PLD optima exist for adsorbent structures where the distance between its Connolly surfaces corresponds to the length of the adsorbed molecule. The latter can then simultaneously interact with different atoms with the accessible surface. [41] The high surface-specific uptake values for benzene for UiO-66(F)$_4$ and ZIF-11 (at p p_0^{-1} 0.1) *vs.* a PLD of 2.4x4.6 and 3.0x3.0 Å (Table S2) may correlate with the 'thickness' of benzene. Yet, this contrasts with the below-average uptake values for ZIF-7 and ZIF-8, having similar PLDs or 2.9x2.9 and 3.0x3.0 Å (Table S2). Also, the PLD of MIL-160 with 5.5x5.5 Å is not much higher, yet the surface-specific uptake is average. This does not suggest an optimal pore diameter for benzene adsorption. For cyclohexane and for hexane (Figure S89) a first maximum is discernible from 5-7 Å, which could correlate with the dimensions of cyclohexane but not really with those of *n*-hexane. The surface-specific uptake at 0.9 p p_0^{-1} (Figure S90) shows a high value around 4 Å for UiO-66(F_4) for benzene, 3-4 Å for ZIF-11 and UiO-66(F_4) for cyclohexane and at 3 Å for ZIF-7 for *n*-hexane. Yet, MOFs with similar PLDs have at the same time much lower surface-specific uptakes. This rules out a significant correlation and suggests that the C_6 uptake is controlled by interactions of the individual VOCs other than a simple physisorption at opposite pore walls.

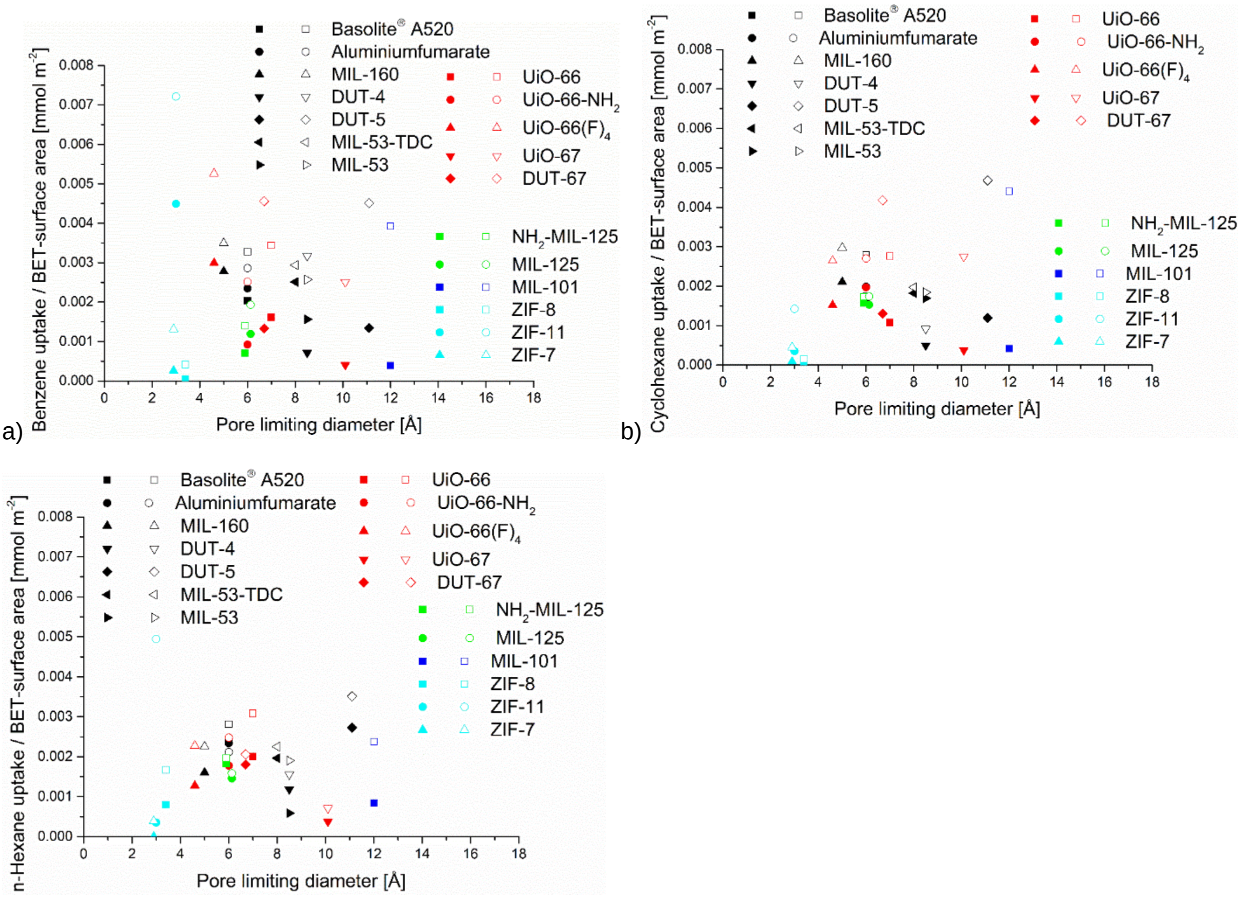

***Figure S89** Surface-specific uptake vs. pore limiting diameter (PLD) at 0.1 (blank) and 0.01(filled) p p_0^{-1} for (a) benzene, (b) cyclohexane, and (c) n-hexane.*

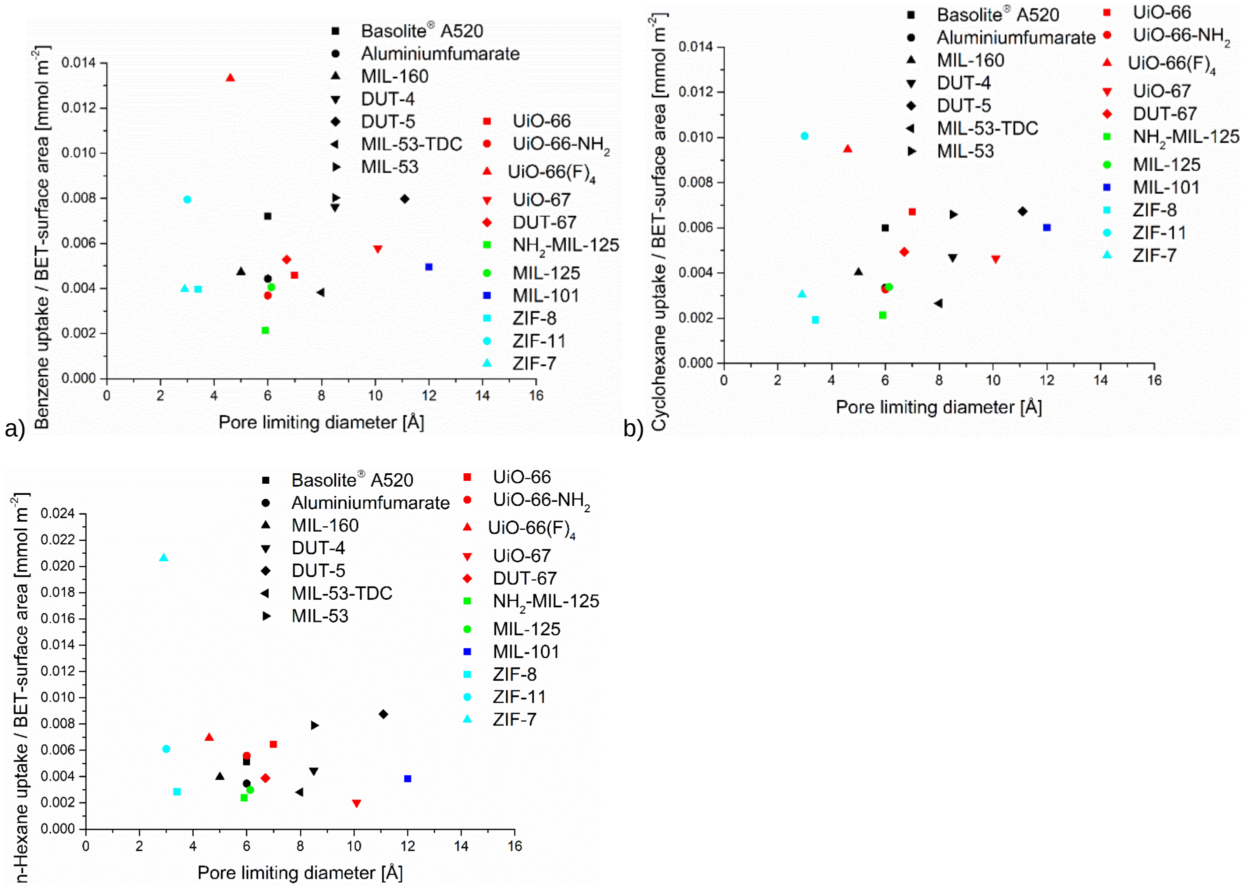

***Figure S90** Surface-specific uptake vs. pore limiting diameter (PLD) at 0.9 p p_0^{-1} for (a) benzene, (b) cyclohexane, and (c) n-hexane.*

S10 VOC sorption studies

Method of isotherm fitting

The measurement of adsorption isotherms of benezene, cyclohexane and n-hexane under the same conditions are used to examine sorption capacity and affinity and are further used to calculate the IAST-selectivity (S11). Fitting-simulations were calculated using 3P sim software.[48] We applied several commonly used models on our isotherm data. The best comparability for all three adsorption isotherms is obtained by applying the same model on all isotherm data and Dual-site Langmuir Sips was used. Fitting parameters are shown in Table S10 and Table S11.

DSLAISips

$$q_{eq} = q_{max} * \left(\frac{K_1 * p}{1 + K_1 * p} + \frac{(K_2 * p)^t}{(1 + K_2 * p)^t} \right)$$

q_{eq}= amount adsorbed [mmol g^{-1}]
q_{max} = maximum adsorption capacity [mmol g^{-1}]
K = affinity constant for adsorption [1 bar^{-1}]
p = pressure [bar]
t = index of heterogeneity

Table S10 *Model and R^2 of the fitted isotherms and simulated pressure range.*

MOF	VOC (X_1 and X_2)	Model	R^2(X1)=	R^2(X2)=	Simulated pressure range up to xx bar
Basolite® A520	Benzene and Cyclohexane	DSLAISips	0.994	0.984	0.09
	Benzene and n-Hexane	DSLAISips	0.994	0.991	0.09
	n-Hexane and Cyclohexane	DSLAISips	0.984	0.991	0.09
Aluminumfumarate	Benzene and Cyclohexane	DSLAISips	0.977	0.990	0.08
	Benzene and n-Hexane	DSLAISips	0.977	0.991	0.09
	n-Hexane and Cyclohexane	DSLAISips	0.990	0.991	0.08
MIL-160	Benzene and Cyclohexane	DSLAISips	0.979	0.997	0.09
	Benzene and n-Hexane	DSLAISips	0.979	0.995	0.09
	n-Hexane and Cyclohexane	DSLAISips	0.997	0.995	0.09
DUT-4	Benzene and Cyclohexane	DSLAISips	0.991	0.986	0.09
	Benzene and n-Hexane	DSLAISips	0.991	0.997	0.08
	n-Hexane and Cyclohexane	DSLAISips	0.986	0.997	0.08
DUT-5	Benzene and Cyclohexane	DSLAISips	0.996	0.992	0.09
	Benzene and n-Hexane	DSLAISips	0.996	0.993	0.09
	n-Hexane and Cyclohexane	DSLAISips	0.992	0.993	0.09
MIL-53-TDC	Benzene and Cyclohexane	DSLAISips	0.986	0.978	0.08
	Benzene and n-Hexane	DSLAISips	0.986	0.997	0.09
	n-Hexane and Cyclohexane	DSLAISips	0.978	0.997	0.08

MIL-53(Al)	Benzene and Cyclohexane	DSLAISips	0.961	0.965	0.05
	Benzene and *n*-Hexane	DSLAISips	0.961	0.990	0.09
	Cyclohexane and *n*-Hexane	DSLAISips	0.965	0.990	0.05
UiO-66	Benzene and Cyclohexane	DSLAISips	0.995	0.985	0.08
	Benzene and *n*-Hexane	DSLAISips	0.995	0.997	0.09
	n-Hexane and Cyclohexane	DSLAISips	0.985	0.997	0.08
UiO-66-NH_2	Cyclohexane and Benzene	DSLAISips	0.983	0.987	0.09
	n-Hexane and Benzene	DSLAISips	0.983	0.992	0.09
	Cyclohexane and *n*-Hexane	DSLAISips	0.987	0.992	0.09
UiO-66(F)$_4$	Benzene and Cyclohexane	DSLAISips	0.978	0.960	0.09
	Benzene and *n*-Hexane	DSLAISips	0.978	0.993	0.09
	Cyclohexane and *n*-Hexane	DSLAISips	0.960	0.993	0.09
UiO-67	Benzene and Cyclohexane	DSLAISips	0.998	0.987	0.09
	Benzene and *n*-Hexane	DSLAISips	0.998	0.993	0.09
	Cyclohexane and *n*-Hexane	DSLAISips	0.987	0.993	0.09
DUT-67	Benzene and Cyclohexane	DSLAISips	0.995	0.994	0.09
	Benzene and *n*-Hexane	DSLAISips	0.995	0.984	0.09
	Cyclohexane and *n*-Hexane	DSLAISips	0.994	0.984	0.09
NH_2-MIL-125	Cyclohexane and Benzene	DSLAISips	0.998	0.987	0.09
	n-Hexane and Benzene	DSLAISips	0.998	0.992	0.09
	n-Hexane and Cyclohexane	DSLAISips	0.987	0.992	0.09
MIL-125	Cyclohexane and Benzene	DSLAISips	0.971	0.982	0.08
	n-Hexane and Benzene	DSLAISips	0.971	0.989	0.08
	n-Hexane and Cyclohexane	DSLAISips	0.982	0.989	0.09
MIL-101(Cr)	Cyclohexane and Benzene	DSLAISips	0.997	0.998	0.09
	Benzene and *n*-Hexane	DSLAISips	0.997	0.996	0.09
	Cyclohexane and *n*-Hexane	DSLAISips	0.998	0.996	0.09
ZIF-8	Benzene and Cyclohexane	DSLAISips	1.000	0.998	0.09
	n-Hexane and Benzene	DSLAISips	1.000	0.996	0.09
	n-Hexane and Cyclohexane	DSLAISips	0.998	0.996	0.09

ZIF-11	Benzene and Cyclohexane	DSLAISips	0.999	0.998	0.09
	Benzene and *n*-Hexane	DSLAISips	0.999	0.998	0.09
	n-Hexane and Cyclohexane	DSLAISips	0.998	0.998	0.09
ZIF-7	Benzene and Cyclohexane	DSLAISips	0.999	0.986	0.09
	Benzene and *n*-Hexane	DSLAISips	0.999	0.999	0.09
	n-Hexane and Cyclohexane	DSLAISips	0.986	0.999	0.09

Table S11 *Dual-site Langmuir Sips fitting parameters used for the calculation of VOC IAST-selectivities.*

**Model**		**Dual Site Langmuir Sips**			
		**Parameters**			
**MOF**	**VOC**	**Affinity const. 1 [1 bar^{-1}]**	**Max. loading [mmol g^{-1}]**	**Affinity const. 2 [1 bar^{-1}]**	**Heterogenity exponent**
Basolite® A520	Benzene	565.165	5.344	8.662	2.885
	Cyclohexane	771.935	3.288	13.069	3.435
	n-Hexane	1767.028	2.815	7.009	1.739
Aluminumfumarate	Benzene	2359.052	3.156	10.041	3.231
	Cyclohexane	2398.896	2.157	9.814	2.295
	n-Hexane	6101.405	2.368	4.330	1.392
MIL-160	Benzene	1906.082	4.293	6.525	1.799
	Cyclohexane	978.990	3.814	6.450	2.648
	n-Hexane	6633.506	2.641	8.982	4.255
DUT-4	Benzene	292.982	7.013	16.782	3.358
	Cyclohexane	44.246	5.147	13.667	4.803
	n-Hexane	2203.091	2.606	11.936	1.691
DUT-5	Benzene	353.543	7.596	10.008	2.845
	Cyclohexane	8.351	5.938	489.205	1.770
	n-Hexane	1255.007	4.958	11.869	4.222
MIL-53-TDC	Benzene	5487.091	2.999	5.519	1.606
	Cyclohexane	9999.000	2.010	5.779	1.553
	n-Hexane	7597.648	2.087	1.946	0.675
MIL-53(Al)	Benzene	713.604	5.153	15.410	6.910
	Cyclohexane	2956.745	2.572	36.003	3.872
	n-Hexane	135.134	3.820	11.820	5.110
UiO-66	Benzene	606.109	4.662	5.829	2.467

	Cyclohexane	404.155	3.189	16.820	7.457
	n-Hexane	859.966	3.885	9.506	8.077
UiO-66-NH_2	Benzene	472.539	3.455	7.372	3.049
	Cyclohexane	338.817	1.738	2180.625	3.405
	n-Hexane	920.661	3.111	10.140	7.879
UiO-66(F)$_4$	Benzene	586.254	2.365	14.568	4.585
	Cyclohexane	145.389	1.688	12.989	9.852
	n-Hexane	793.770	0.783	12.819	2.181
UiO-67	Benzene	188.863	6.759	67.131	3.929
	Cyclohexane	287.584	7.801	9.688	4.009
	n-Hexane	778.205	4.033	6.937	3.084
DUT-67	Benzene	414.857	3.009	731.498	3.872
	Cyclohexane	300.064	2.822	700.796	2.082
	n-Hexane	2261.157	2.517	9.264	5.215
NH_2-MIL-125	Benzene	708.042	2.479	7.690	1.489
	Cyclohexane	7062.105	2.707	4.008	1.378
	n-Hexane	5258.730	3.109	2.698	1.736
MIL-125	Benzene	1331.255	3.797	17.309	5.896
	Cyclohexane	1773.340	4.140	13.836	4.820
	n-Hexane	8831.719	3.567	9.143	5.437
MIL-101(Cr)	Benzene	167.326	4.916	197.208	3.087
	Cyclohexane	92.542	6.096	210.578	2.661
	n-Hexane	279.271	5.383	6.399	3.434
ZIF-8	Benzene	21.671	3.836	35.711	5.987
	Cyclohexane	2.183	8.673	7.140	3.566
	n-Hexane	596.021	2.950	7.099	4.050
ZIF-11	Benzene	1293.041	3.475	2.769	2.530
	Cyclohexane	33.274	2.471	46.363	11.594
	n-Hexane	2.039	2.176	745.968	1.773
ZIF-7	Benzene	4.708	1.330	10.418	0.623
	Cyclohexane	16.907	0.615	10.842	20.426
	n-Hexane	9.769	0.678	59.865	4.525

Fitted isotherms

Al-MOFs

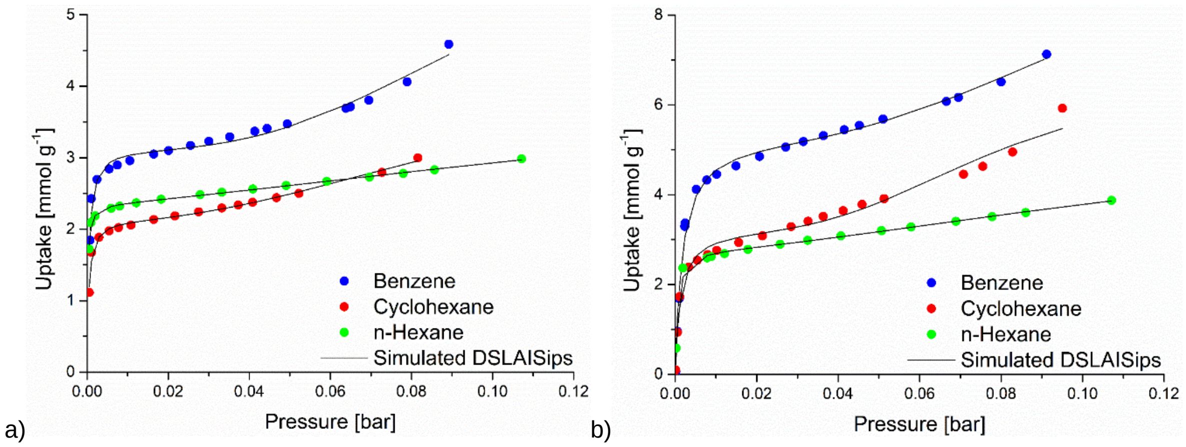

Figure S91 *Experimental sorption isotherms (circles) and fitted VOC adsorption isotherms (line) of Basolite® A520 (a) and Aluminumfumarate (b).*

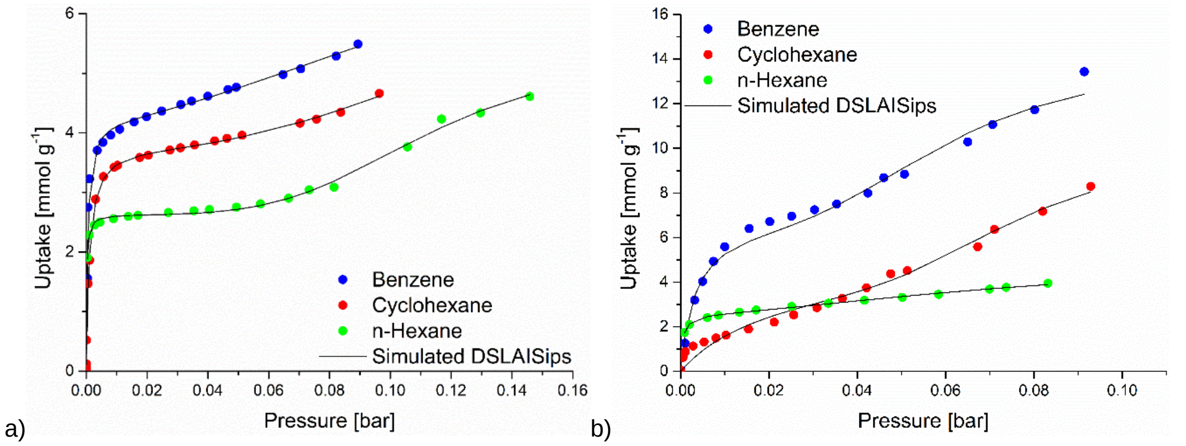

Figure S92 *Experimental sorption isotherms (circles) and fitted VOC adsorption isotherms (line) of MIL-160 (a) and DUT-4 (b).*

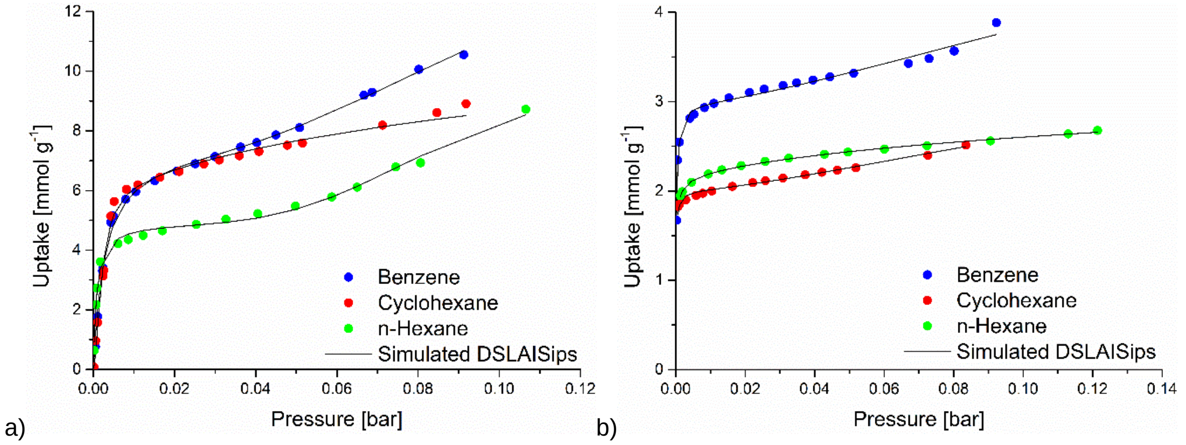

Figure S93 *Experimental sorption isotherms (circles) and fitted VOC adsorption isotherms (line) of DUT-5 (a) and MIL-53-TDC (b).*

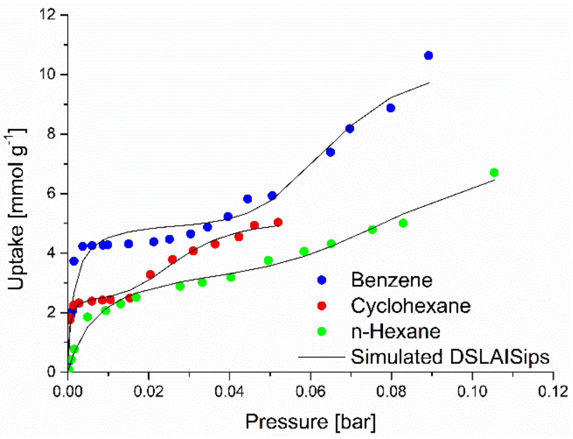

Figure S94 *Experimental sorption isotherms (circles) and fitted VOC adsorption isotherms (line) of MIL-53.*

Zr-MOFs

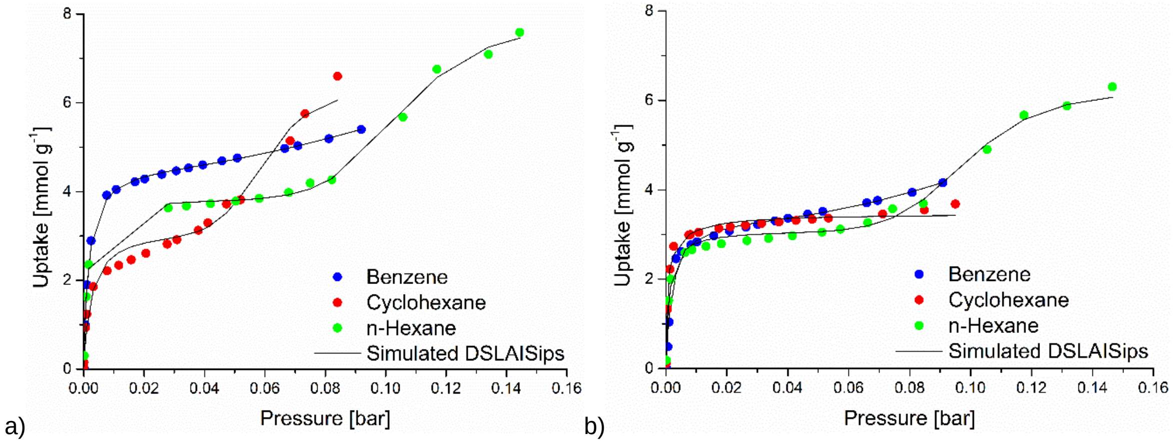

Figure S95 *Experimental sorption isotherms (circles) and fitted VOC adsorption isotherms (line) of UiO-66 (a) and UiO-66-NH_2 (b).*

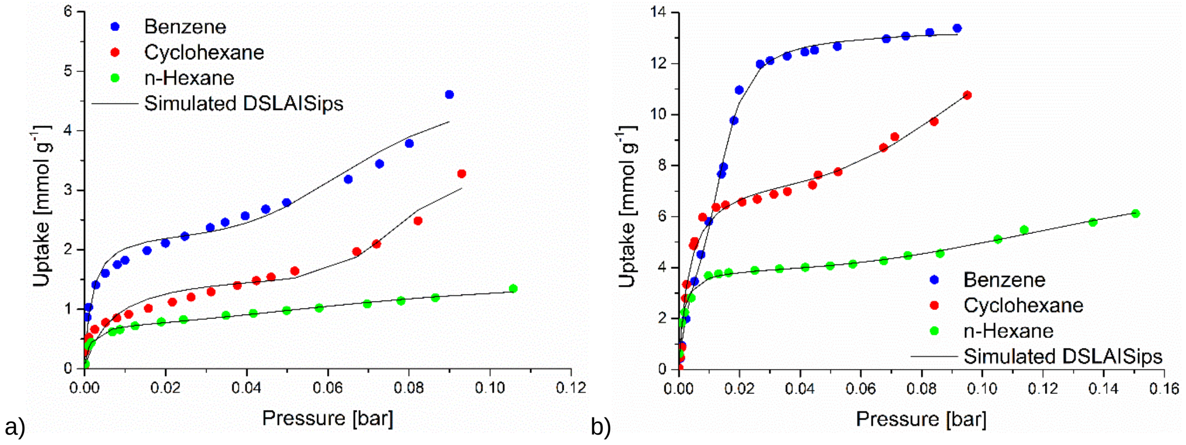

Figure S96 *Experimental sorption isotherms (circles) and fitted VOC adsorption isotherms (line) of UiO-66(F)$_4$ (a) and UiO-67 (b).*

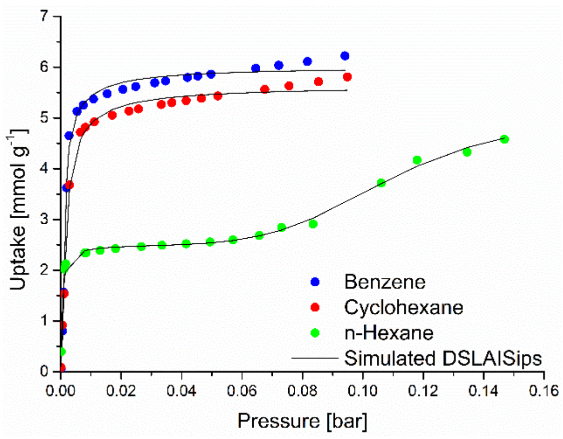

Figure S97 *Experimental sorption isotherms (circles) and fitted VOC adsorption isotherms (line) of DUT-67.*

Ti-MOFs

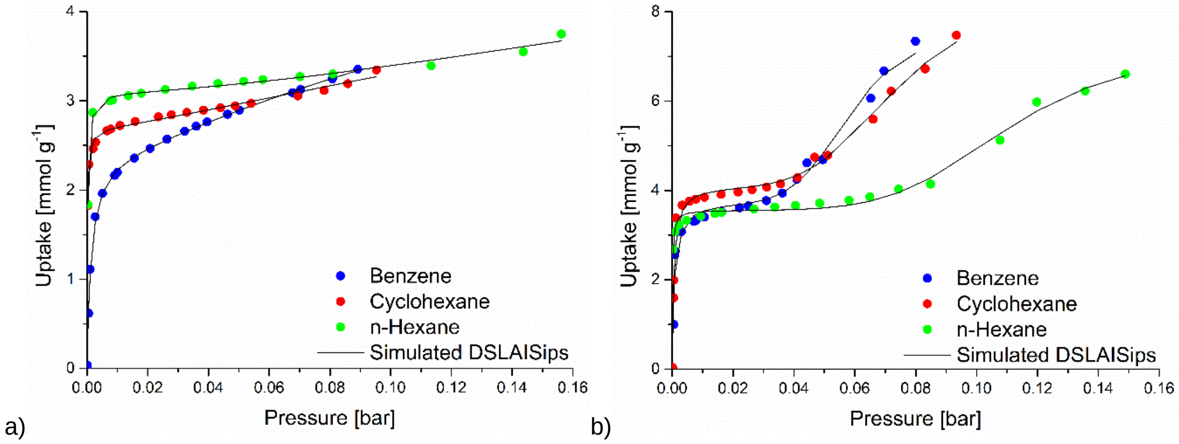

Figure S98 *Experimental sorption isotherms (circles) and fitted VOC adsorption isotherms (line) of NH_2-MIL-125 (a) and MIL-125 (b).*

Cr-MOF

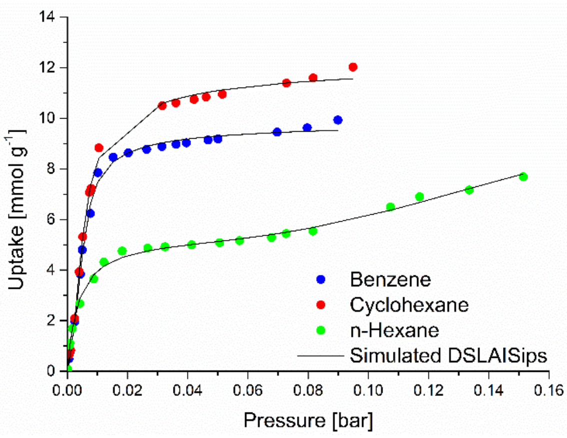

Figure S99 *Experimental sorption isotherms (circles) and fitted VOC adsorption isotherms (line) of MIL-101(Cr).*

ZIFs

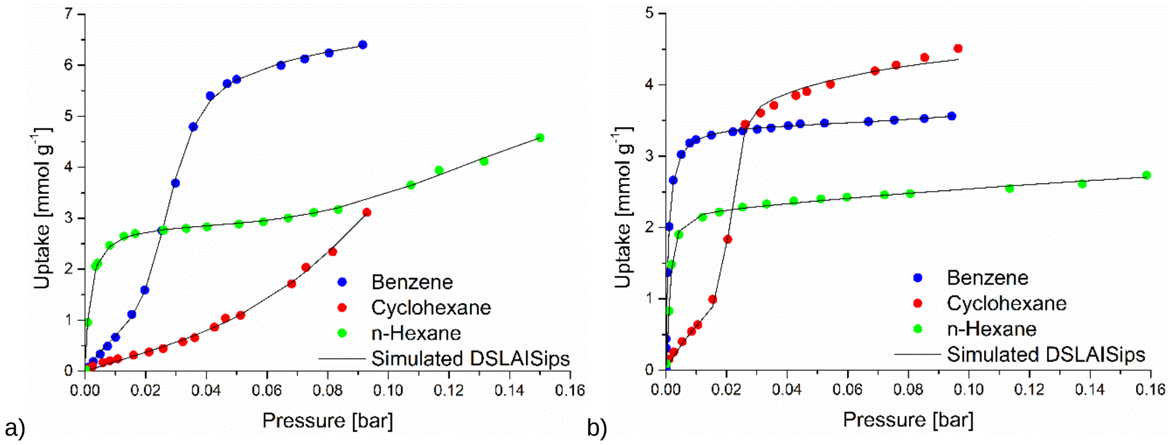

Figure S100 *Experimental sorption isotherms (circles) and fitted VOC adsorption isotherms (line) of ZIF-8 (a) and ZIF-11 (b).*

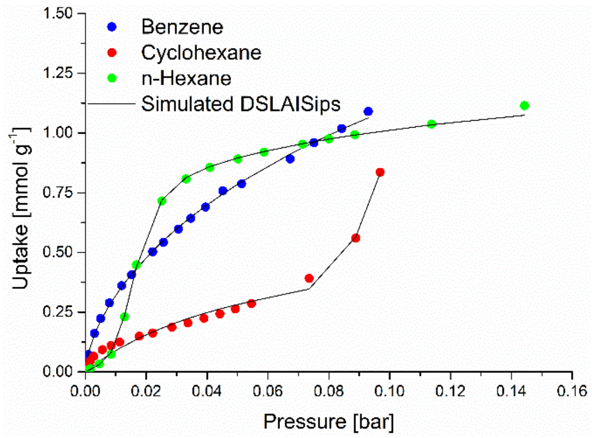

Figure S101 *Experimental sorption isotherms (circles) and fitted VOC adsorption isotherms (line) of ZIF-7.*

S11 Ideal Adsorbed Solution Theory, IAST-Selectivity

Method

Selectivities of VOCs in Figure 7 in the main article and

Table **S12** were calculated from fitted isotherm data (Section S10, Table S10 and Table S11) of the experimentally measured isotherms at 293 K. Here the VOC sorption isotherms are fitted with the dual-site Langmuir Sips model with the “3P sim” software (see above in section S10, Supplementary Information). The 3P sim software (3P Instruments, Germany, version 1.1.0.7) calculates the maximal loadings of each vapor depending on the given mole fraction. IAST selectivities S of binary vapor mixtures were calculated using the following equation, where x_i represents the absorbed gas amount and y_i the mole fraction of each adsorptive.

IAST-Selectivity

$$S = \frac{x_1/x_2}{y_1/y_2}$$

IAST selectivities were calculated over the pressure range of the fitted isotherms from 0.00 up to a maximum pressure of 0.09 bar. The pressure region below 0.01 was excluded from evaluation to avoid errors from the isotherm fitting in this region.

IAST-based estimations are most suitable when the following conditions are fulfilled: (i) the same accessible surface area is available to all adsorbates, (ii) low pressures (⩽1 bar), and (iii) similar polarity of adsorbates.[49]

Al-MOFs

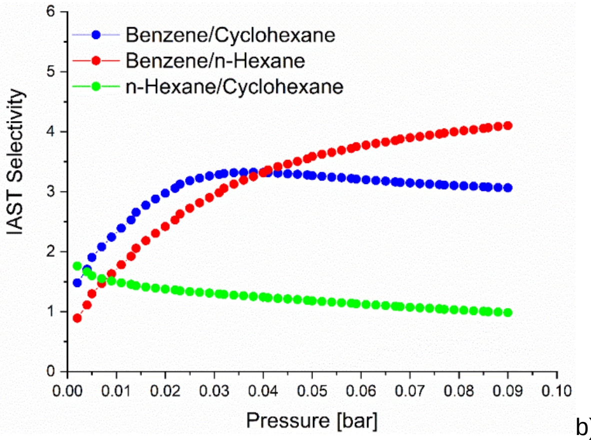

a)

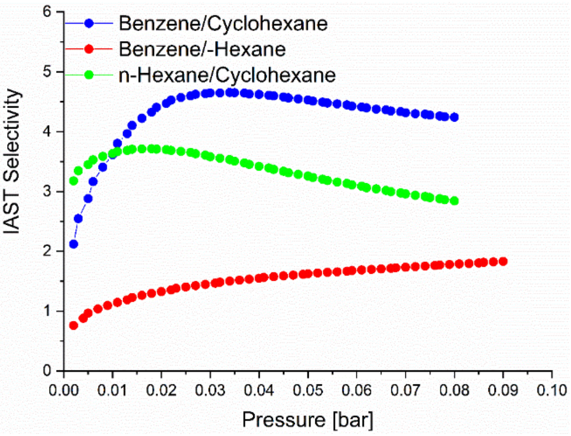

b)

Figure S102 *The calculated VOC selectivity dependence on the pressure range [bar] for Basolite® A520 (a) and Aluminumfumarate (b).*

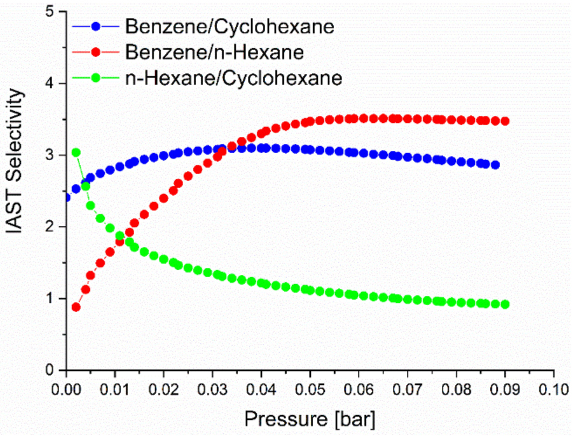

a)

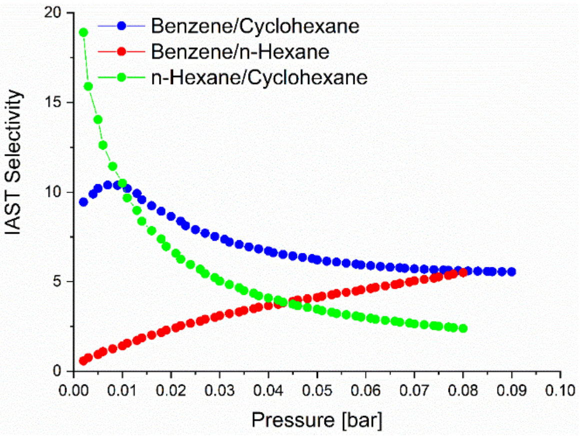

b)

Figure S103 *The calculated VOC selectivity dependence on the pressure range [bar] for MIL-160 (a) and DUT-4 (b).*

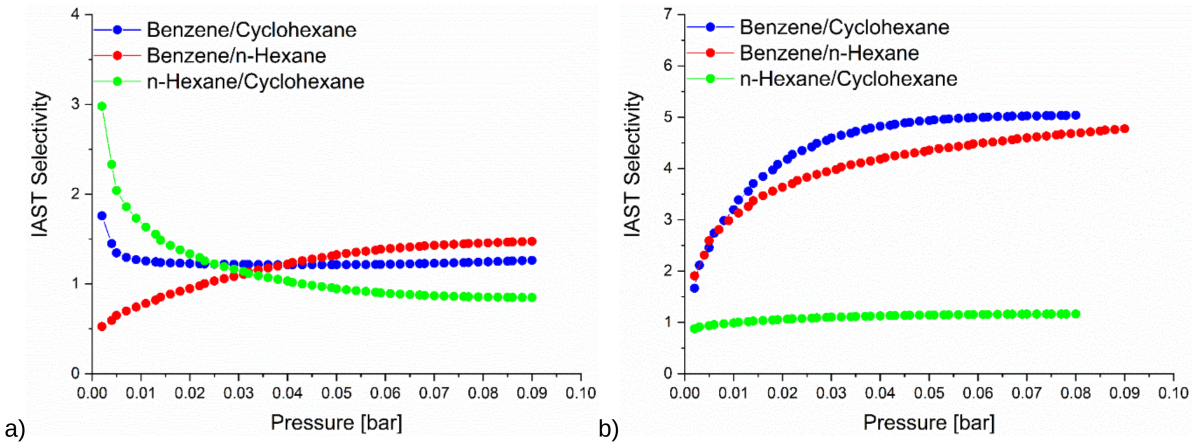

Figure S104 *The calculated VOC selectivity dependence on the pressure range [bar] for DUT-5 (a) and MIL-53-TDC (b).*

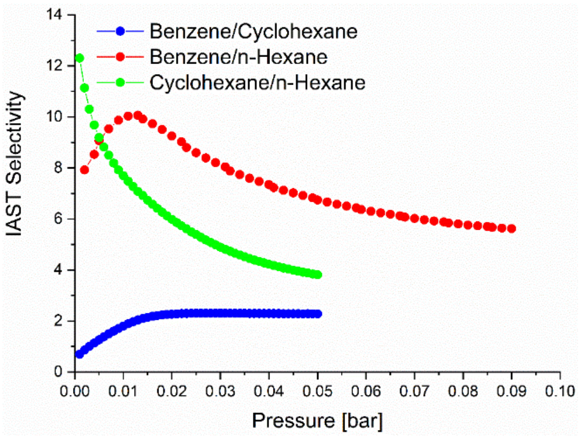

Figure S105 *The calculated VOC selectivity dependence on the pressure range [bar] for MIL-53.*

Zr-MOFs

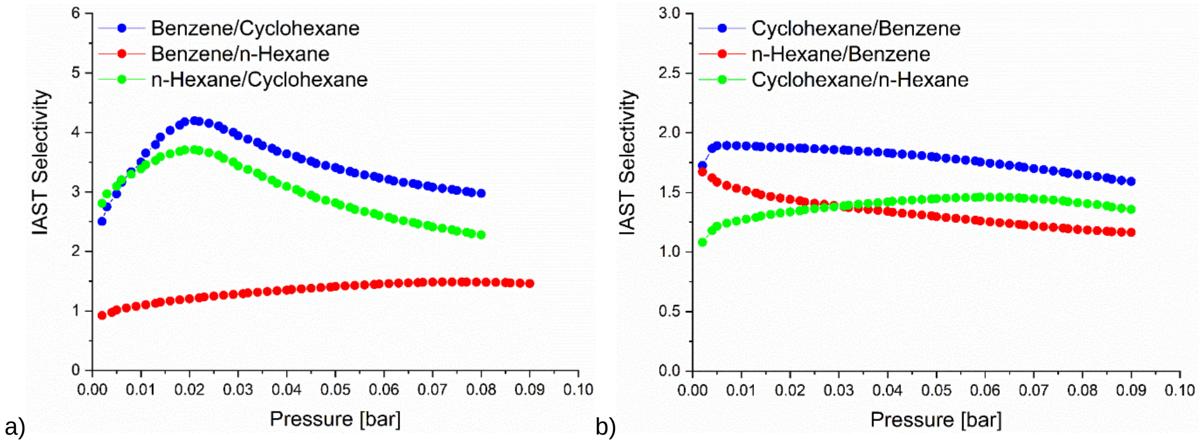

Figure S106 *The calculated VOC selectivity dependence on the pressure range [bar] for UiO-66 (a) and UiO-66-NH_2 (b).*

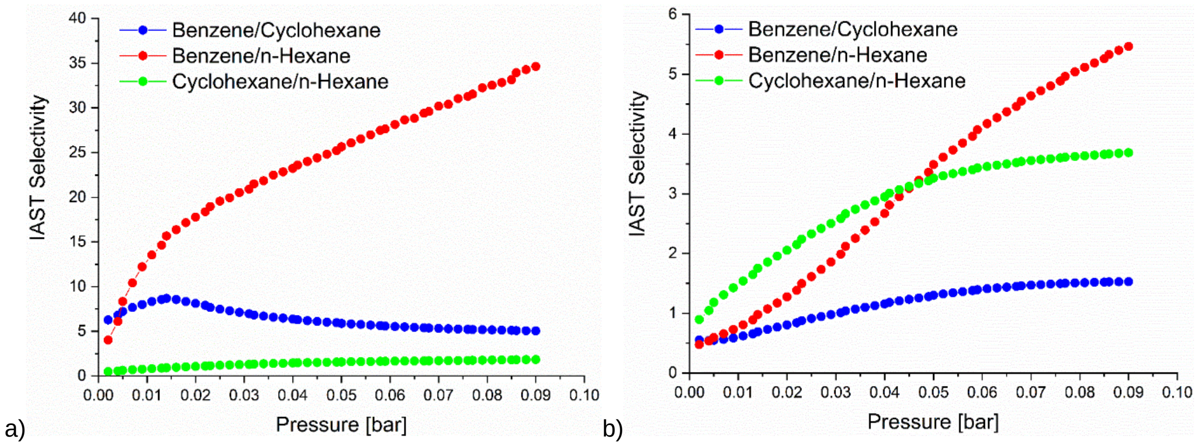

Figure S107 *The calculated VOC selectivity dependence on the pressure range [bar] for UiO-66(F)$_4$ (a) and UiO-67 (b).*

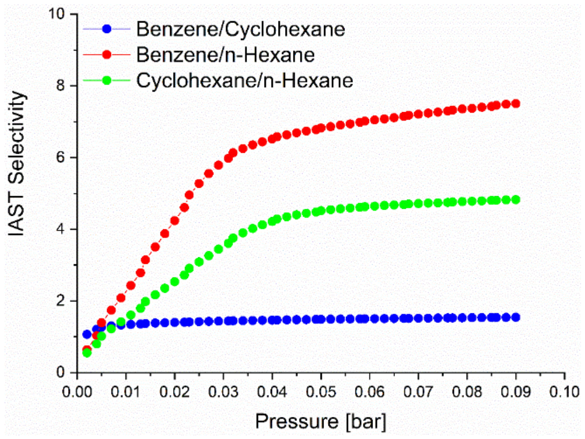

Figure S108 *The calculated VOC selectivity dependence on the pressure range [bar] for DUT-67*

Ti-MOFs

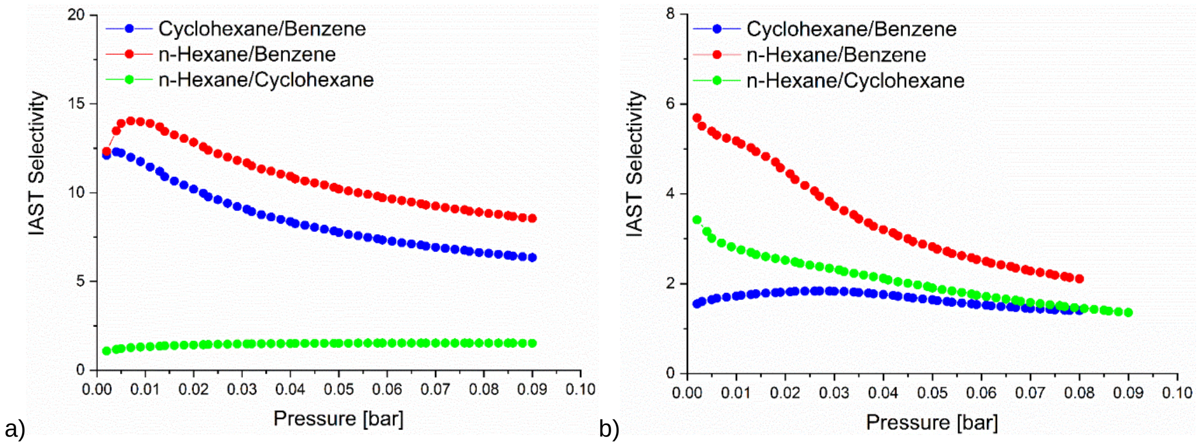

Figure S109 *The calculated VOC selectivity dependence on the pressure range [bar] for NH$_2$-MIL-125 (a) and MIL-125 (b).*

Cr-MOFs

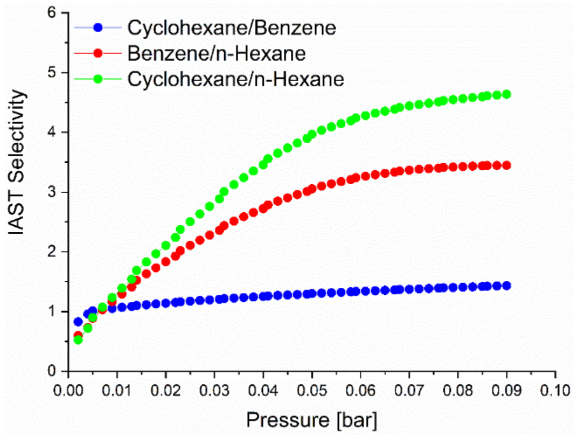

Figure S110 *The calculated VOC selectivity dependence on the pressure range [bar] for MIL-101(Cr).*

ZIFs

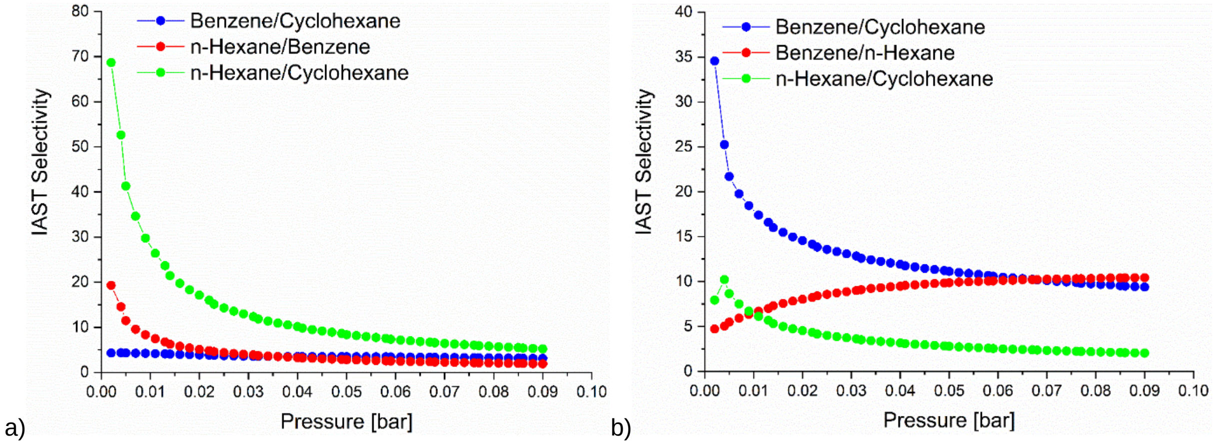

Figure S111 *The calculated VOC selectivity dependence on the pressure range [bar] for ZIF-8 (a) and ZIF-11 (b).*

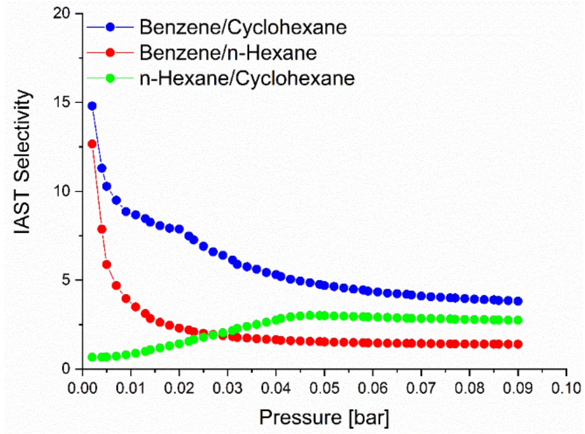

Figure S112 *The calculated VOC selectivity dependence on the pressure range [bar] for ZIF-7.*

All calculated binary IAST selectivities for all examined materials are presented in Figure 7 in the main article.

Table S12 *IAST selectivtiy of a 50/50 molar ratio of the different binary mixtures at different pressure for each MOF.*

**MOF**	**VOC (X_1 and X_2)**	**IAST Selectivity for X_1/X_2** (rounded to the next integer value)		
		**0.01 [bar]**	**0.05 [bar]**	**0.09 [bar]**
Basolite® A520	Benzene and Cyclohexane	2	3	3
	Benzene and *n*-Hexane	2	4	4
	n-Hexane and Cyclohexane	1	1	1
Aluminumfumarate	Benzene and Cyclohexane	4	5	4
	Benzene and *n*-Hexane	1	2	2
	n-Hexane and Cyclohexane	4	3	3
MIL-160	Benzene and Cyclohexane	3	3	0
	Benzene and *n*-Hexane	2	3	3
	n-Hexane and Cyclohexane	2	1	1
DUT-4	Benzene and Cyclohexane	10	6	6
	Benzene and *n*-Hexane	1	4	6
	n-Hexane and Cyclohexane	10	3	2
DUT-5	Benzene and Cyclohexane	1	1	1
	Benzene and *n*-Hexane	1	1	1
	n-Hexane and Cyclohexane	2	1	1
MIL-53-TDC	Benzene and Cyclohexane	3	5	5
	Benzene and *n*-Hexane	3	4	5
	n-Hexane and Cyclohexane	1	1	1
MIL-53(Al)	Benzene and Cyclohexane	2	2	-
	Benzene and *n*-Hexane	10	7	6
	Cyclohexane and *n*-Hexane	8	4	-
UiO-66	Benzene and Cyclohexane	4	3	3
	Benzene and *n*-Hexane	1	1	1
	n-Hexane and Cyclohexane	3	3	2
UiO-66-NH_2	Cyclohexane and Benzene	2	2	2
	n-Hexane and Benzene	2	1	1
	Cyclohexane and *n*-Hexane	1	1	1
UiO-66$(F)_4$	Benzene and Cyclohexane	8	6	5
	Benzene and *n*-Hexane	13	26	35
	Cyclohexane and *n*-Hexane	1	2	2
UiO-67	Benzene and Cyclohexane	1	1	2

	Benzene and *n*-Hexane	1	3	5
	Cyclohexane and *n*-Hexane	1	3	4
DUT-67	Benzene and Cyclohexane	1	1	2
	Benzene and *n*-Hexane	2	7	8
	Cyclohexane and *n*-Hexane	2	5	5
NH_2-MIL-125	Cyclohexane and Benzene	12	8	6
	n-Hexane and Benzene	14	10	9
	n-Hexane and Cyclohexane	1	2	2
MIL-125	Cyclohexane and Benzene	2	2	1
	n-Hexane and Benzene	5	3	2
	n-Hexane and Cyclohexane	3	2	1
MIL-101(Cr)	Cyclohexane and Benzene	1	1	1
	Benzene and *n*-Hexane	1	3	3
	Cyclohexane and *n*-Hexane	1	4	5
ZIF-8	Benzene and Cyclohexane	4	3	3
	n-Hexane and Benzene	8	3	2
	n-Hexane and Cyclohexane	28	8	5
ZIF-11	Benzene and Cyclohexane	18	11	9
	Benzene and *n*-Hexane	6	10	10
	n-Hexane and Cyclohexane	6	3	2
ZIF-7	Benzene and Cyclohexane	9	5	4
	Benzene and *n*-Hexane	4	2	1
	n-Hexane and Cyclohexane	1	3	3

S12 Stability tests

All MOFs were tested under VOC-vapor conditions and in liquid VOCs for five days to simulate a long-term exposure towards benzene, cyclohexane and *n*-hexane. The stability of all 18 MOFs was tested with the following activation protocol. All MOF samples were activated overnight at 120 °C under vacuum and tested afterwards against benzene, cyclohexane and *n*-hexane.

Vapor stability

For the vapor stabilty tests 20 mg of each activated sample was used. Therfore the MOFs were put in an vacuum chamber and one of the three VOCs were let flow in the vacuum to create a VOC atmosphere. Each sample was left for five days under these VOC vapor conditions. Afterwards the samples were analyzed with the PXRD, to see if the crystallinity decreases. The experimental setup can be seen in the following Figure S113.

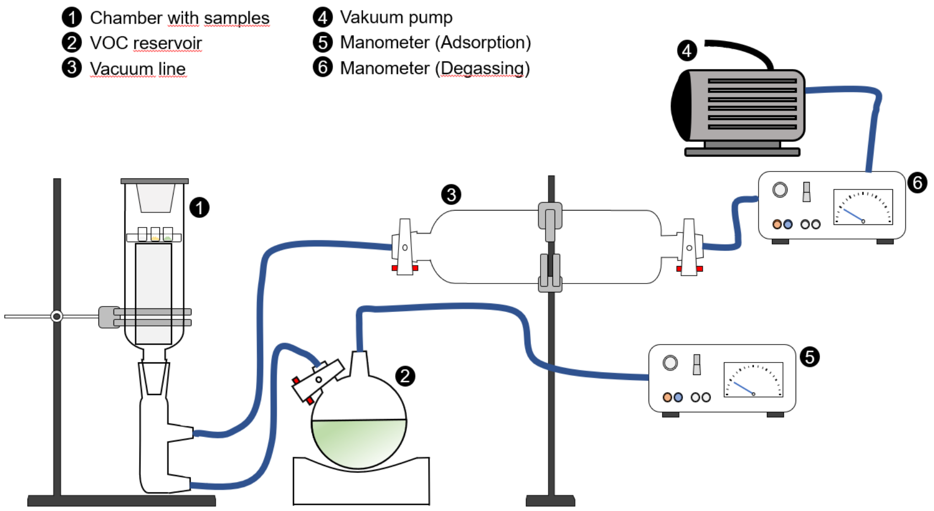

Figure S113 *Schematic graphic of the experimental set up for the vapor stability tests.*

Liquid stability

The stability of the MOFs was also examined against VOC solutions. For the implementation, 10 mg of each of the 18 MOFs were added to 10 mL of the benzene, cyclohexane and *n*-hexane solution, sealed airtight and left to stand for 5 days at room temperature. A large part of the solutions was then drawn off with the aid of a syringe and the remaining residue was dried at 60 °C overnight in a vacuum oven. The samples were then also measured with the PXRD.

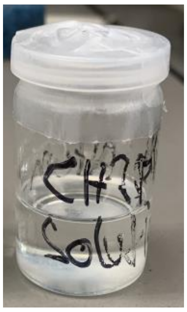

Figure S114 *Exemplary MOF sample in VOC solution.*

Vapor stability was tested with the set-up shown in Figure S114. Details about the experimental conditions and about the set-up are in the supporting information here in chapter S12. The activated MOFs were placed in a glass vial filled with VOC and stored for five days and afterwards PXRD were measured to analyze the samples for un/changed crystallinity. For this, the VOC were removed with a syringe and the MOFs were dried at 60 °C under vacuum and stored under a C_6 vapor. The PXRDs were measured directly, with the adsorbed VOC inside the pores.

Al-MOFs

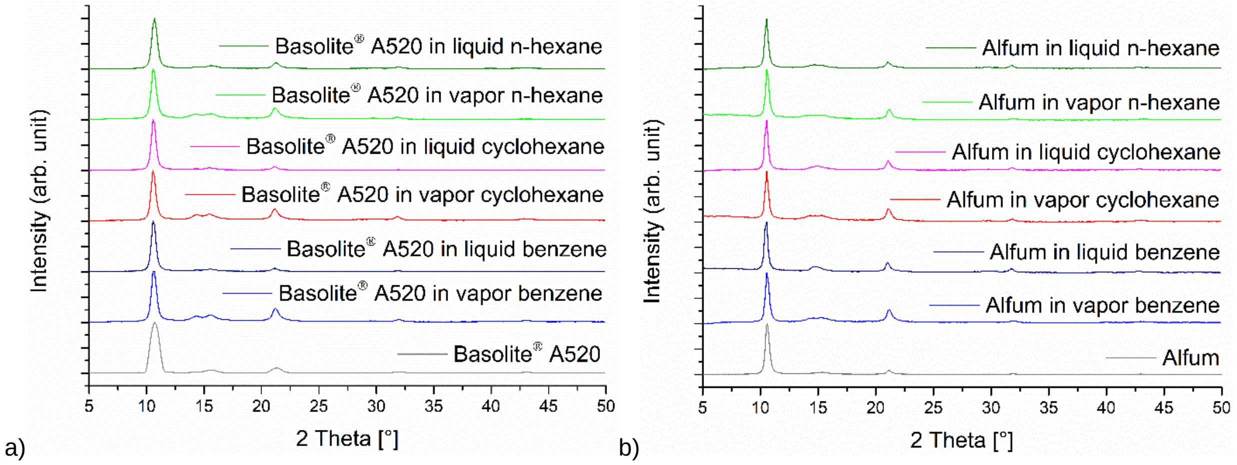

Figure S115 *(a) Stability tests of Basolite® A520 against benzene/cyclohexane/n-hexane in liquid and vapor phase. (b) Stability tests of Aluminumfumarate against benzene/cyclohexane/n-hexane in liquid and vapor phase.*

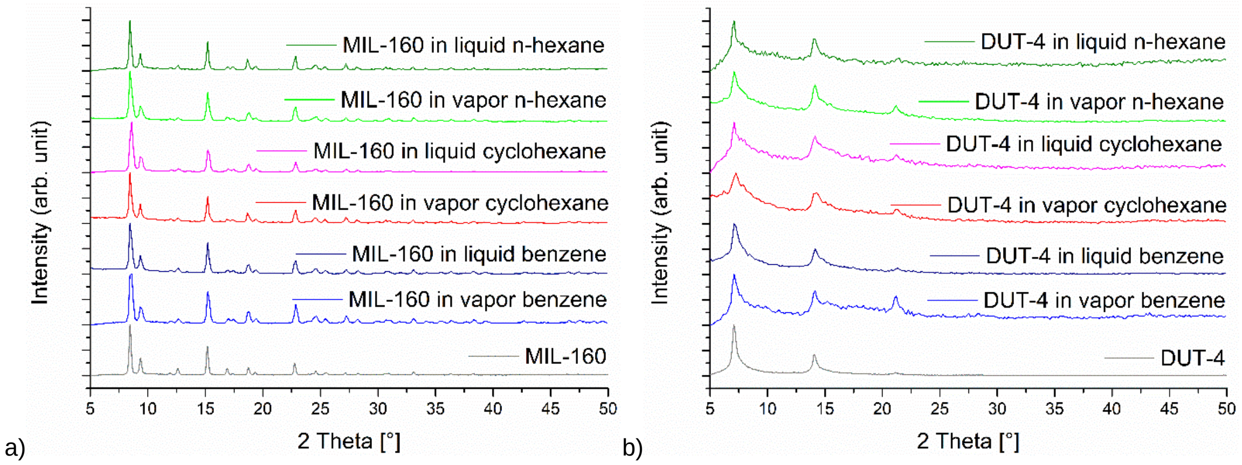

Figure S116 *(a) Stability tests of MIL-160 against benzene/cyclohexane/n-hexane in liquid and vapor phase. (b) Stability tests of DUT-4 against benzene/cyclohexane/n-hexane in liquid and vapor phase.*

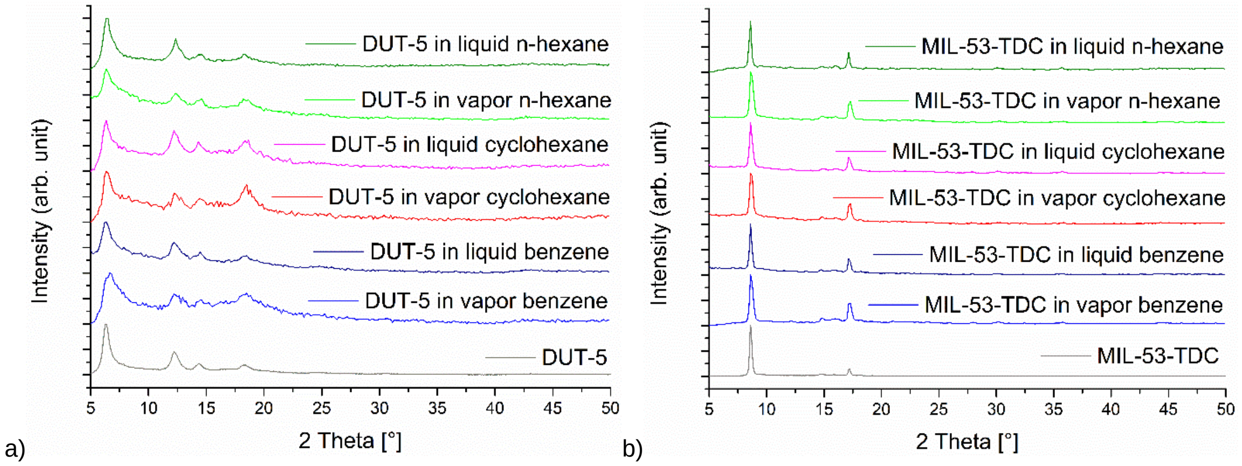

Figure S117 *(a) Stability tests of DUT-5 against benzene/cyclohexane/n-hexane in liquid and vapor phase. (b) Stability tests of MIL-53-TDC against benzene/cyclohexane/n-hexane in liquid and vapor phase.*

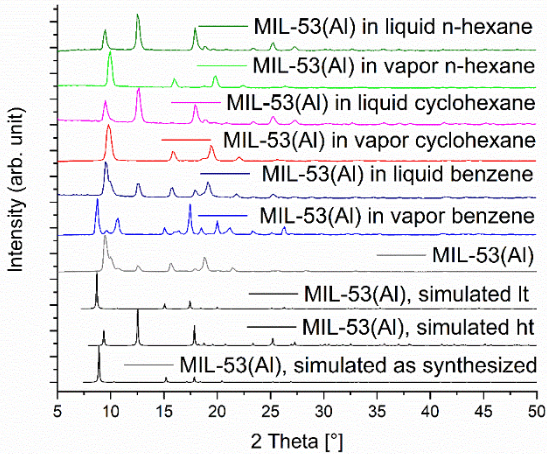

Figure S118 *Stability tests of MIL-53 against benzene/cyclohexane/n-hexane in liquid and vapor phase and with simulated pattern as synthesized, ht, lt (CSD-Number: 797261, 1007172, 220475).*[10,11]

Most of the aluminum-MOFs indicate a good stability against the VOCs, as shown in the PXRDs in Figure S115-Figure S118. There is no loss of crystallinity recognizable and the main peaks are still well resolved. DUT-4 and DUT-5 have a higher background after VOC adsorption, which is due to a lower stability against VOCs. This is supported by the N_2-sorption after the VOC-sorption for DUT-4 and DUT-5, which shows a decrease in the surface area for DUT-4 after cyclohexane-sorption to 372 m^2 g^{-1}. There is a clear difference for MIL-53, compared to the other Al-MOFs, because this MOF has three different phases (as = as synthesized; lt = low temperature/np = narrow pore; ht = high temperature/wp = wide pore), which are correlated with the breathing-effect of this MOF. It is interesting that the PXRD shows the ht-form for benzene (in liquid), cyclohexane (in liquid) and *n*-hexane (in liquid) (Figure S118), but for benzene there is also one diffraction peak of the lt-form at 17° 2θ. This means that drying at 60 ° C under vacuum is sufficient to reactivate the MOF for benzene, cyclohexane and *n*-hexane. The PXRDs which were measured for the samples in vapor show, that there are still molecules inside the pores, due to a measurement without drying.

Zr-MOFs

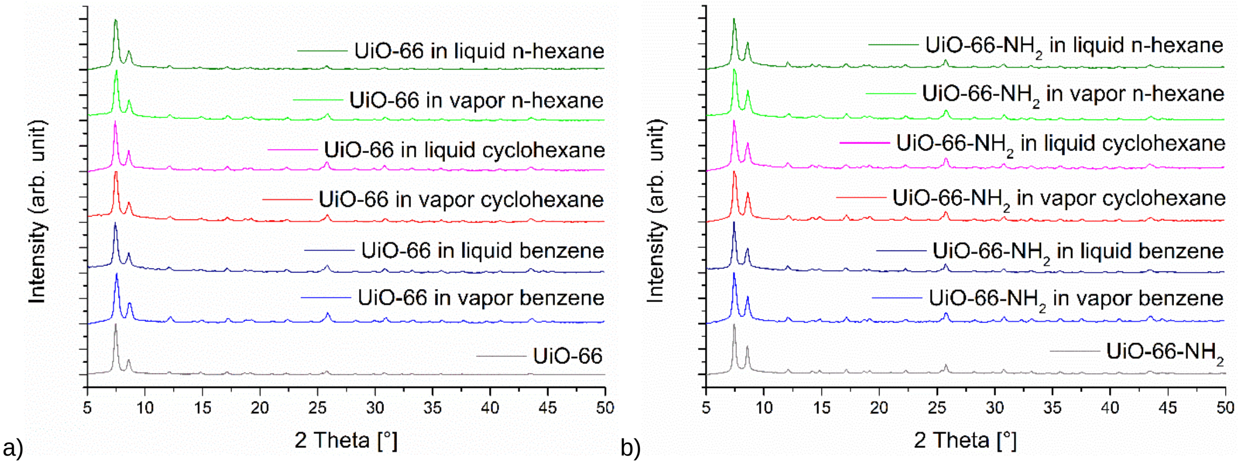

Figure S119 *(a) Stability tests of UiO-66 against benzene/cyclohexane/n-hexane in liquid and vapor phase. (b) Stability tests of UiO-66-NH_2 against benzene/cyclohexane/n-hexane in liquid and vapor phase.*

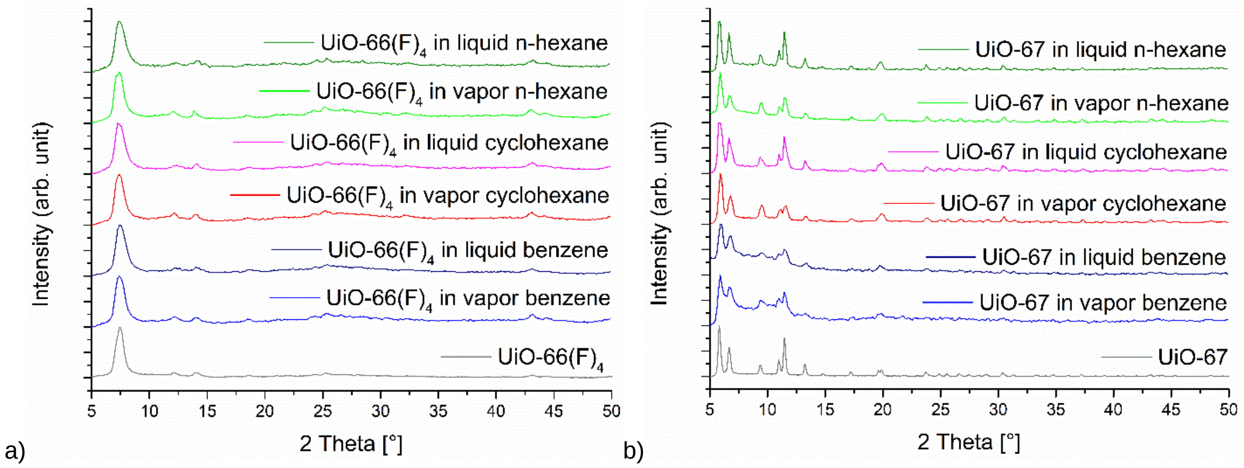

Figure S120 *(a) Stability tests of UiO-66(F)$_4$ against benzene/cyclohexane/n-hexane in liquid and vapor phase. (b) Stability tests of UiO-67 against benzene/cyclohexane/n-hexane in liquid and vapor phase.*

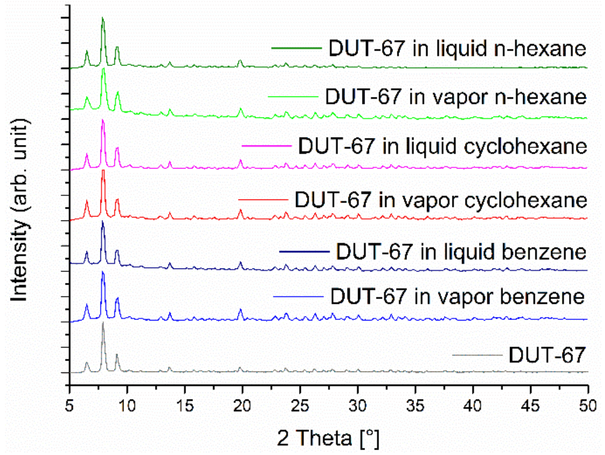

Figure S121 *Stability tests of DUT-67 against benzene/cyclohexane/n-hexane in liquid and vapor phase.*

All Zirconium-MOFs are stable against the C$_6$-VOCs, both in the liquid and the vapor phase. The diffraction peaks of all MOFs are still present (Figure S119-Figure S121). UiO-67 exhibits a broadening of the diffraction peaks and loss of intensity after the treatment with benzene (Figure S120 (b)).

Ti-MOFs

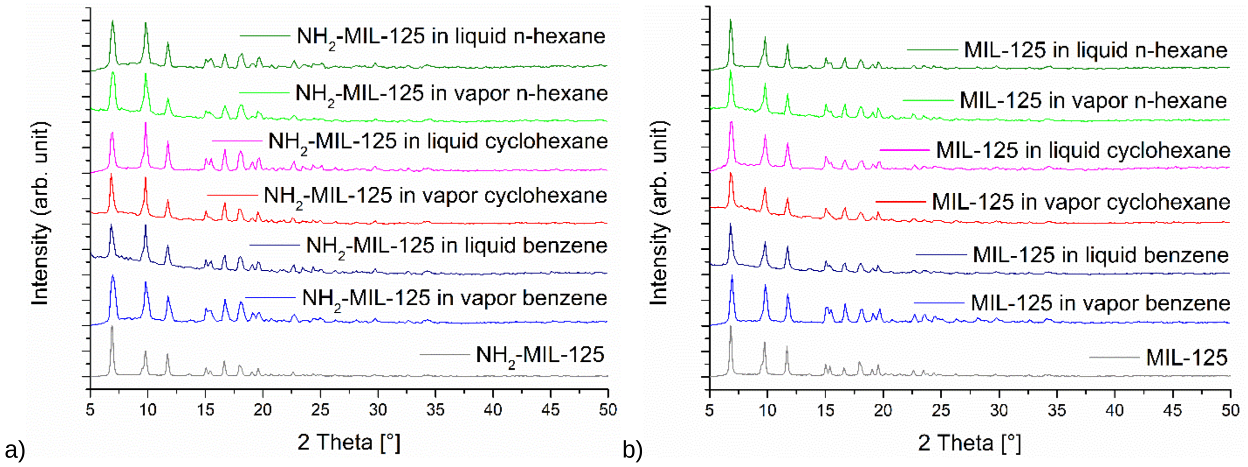

Figure S122 *(a) Stability tests of NH$_2$-MIL-125 against benzene/cyclohexane/n-hexane in liquid and vapor phase. (b) Stability tests of MIL-125 against benzene/cyclohexane/n-hexane in liquid and vapor phase.*

Cr-MOF

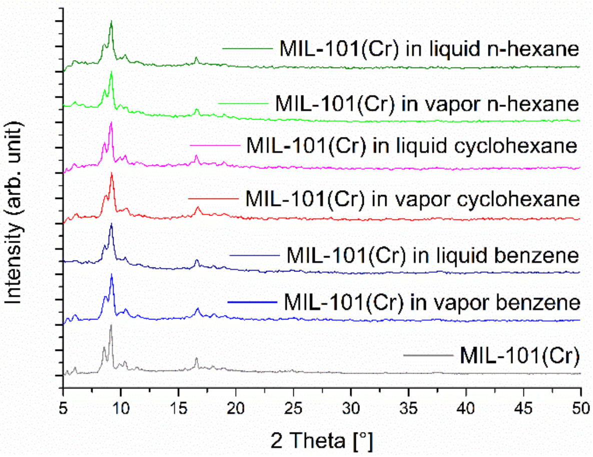

Figure S123 *Stability tests of MIL-101(Cr) against benzene/cyclohexane/n-hexane in liquid and vapor phase.*

Both Titanium-MOFs (MIL-125 and NH_2-MIL-125) and MIL-101(Cr) are stable against all three VOCs in liquid and in the vapor phase. No effect in the PXRD can be seen in Figure S122-Figure S123.

ZIFs

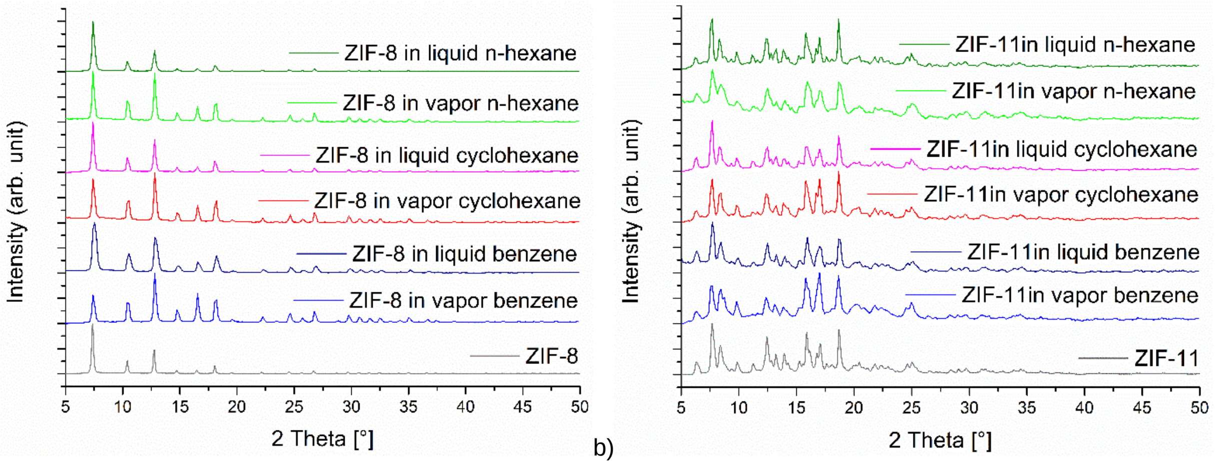

a) b)

Figure S124 *(a) Stability tests of ZIF-8 against benzene/cyclohexane/n-hexane in liquid and vapor phase. (b) Stability tests of ZIF-11 against benzene/cyclohexane/n-hexane in liquid and vapor phase.*

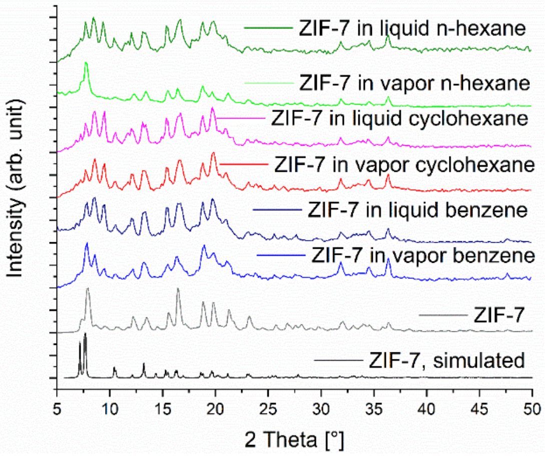

Figure S125 *Stability tests of ZIF-7 against benzene/cyclohexane/n-hexane in liquid and vapor phase and with simulated pattern (CSD-Number: 1036075).[40]*

ZIFs are known for their high stability and all three ZIFs are stable against the VOCs. There is no recognizable loss of crystallinity. For ZIF-7, however, it can be seen that a phase change has taken place, when the material was exposed to the vapor phase of *n*-hexane. The PXRD is shown in Figure S124-Figure S125 and the lower resolution is due to the shortened measurement time. The PXRD shows that a phase change has taken place between two of the three known phases of ZIF-7, namely phase I and II occur, because less diffraction peaks can be seen. The experimental PXRDs of ZIF-7 can be correlated to phase I. Phase III only occurs in connection with water and can be excluded in the absence of any polar solvents, as noted by Zhao et al.[50] The PXRDs of the MOFs with the other experimental conditions differ significantly and show a combination of phase I and II, therefore the simulated pattern of Phase I was shown again, to underline the phase change. I

S13 Gas Sorption at 293 K

The six best performing MOFs at low pressures were further investigated at 293 K with different gases (nitrogen, oxygen, carbon dioxide). The following Figure S126 and Figure S127 show the adsorption isotherms for all six MOFs.

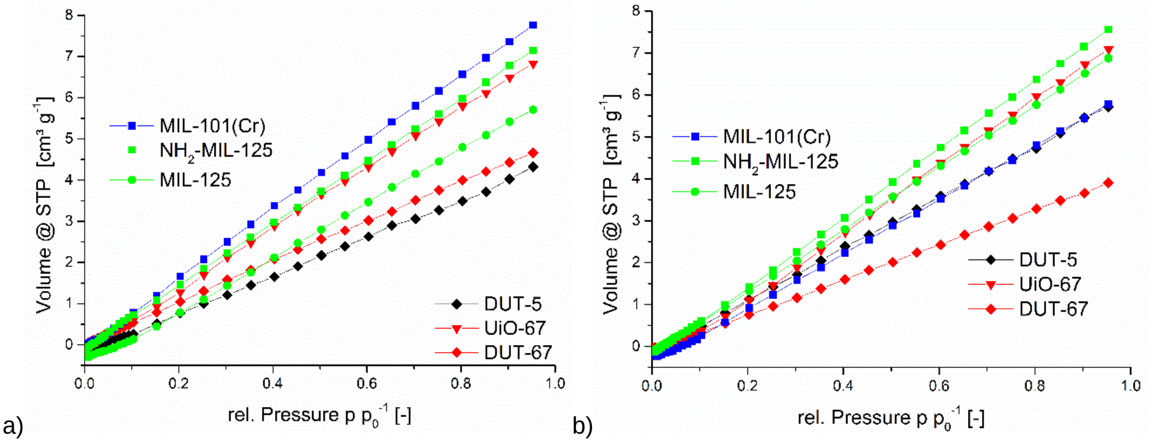

Figure S126 *Nitrogen (a) and oxygen (b) adsorption isotherms at 293 K for the six best performing MOFs at low pressures.*

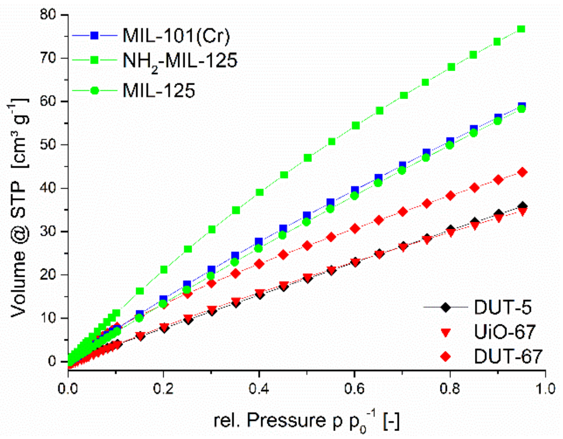

Figure S127 *Carbon dioxide adsorption isotherms at 293 K for the six best performing MOFs at low pressures.*

Due to the higher measurement temperature, in comparison to the standard experiment temperature (e. g. 77 K for nitrogen), the MOFs have no capacity for nitrogen and oxygen. Only the carbon dioxide adsorption is significant at 293 K and competes with the VOCs.

S14 Crystal structures of benzene

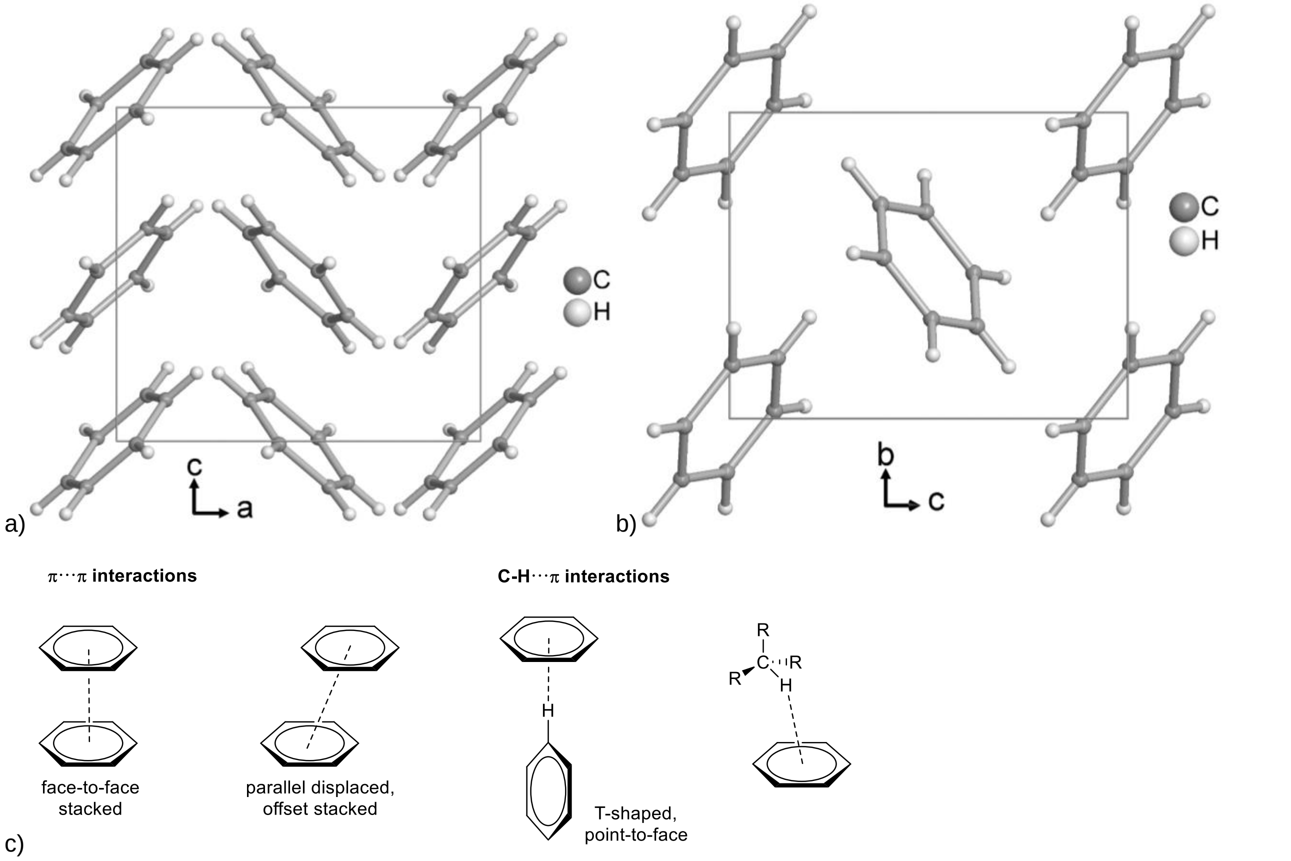

Figure S128 *Section of the packing diagrams in the crystal structure of benzene. (a) Orthorhombic polymorph I (CSD Refcode BENZEN11, CCDC 298305).[51] (b) Monoclinic polymorph II (CSD Refcode BENZEN16, CCDC 757060).[52] (c) Schematic representation of π···π and C-H···π interactions between benzene molecules.*

S15 Images from 'Mercury – Display Voids' calculation

The images picture the solvent accessible surface (outside color light yellow, inside color dark yellow) for a probe radius of 2 Å (the maximum to which the probe radius can be set in Mercury) unless noted otherwise, and an approximate grid spacing of 0.1.

In Mercury voids can be calculated using two different methods:
- Calculate voids using Solvent Accessible Surface gives the volume which can be occupied by the center of a probe of a given radius.
- Calculate voids using Contact Surface maps the volume that can be occupied by the full probe (including its radius) and thus gives a better estimate of the volume that could be filled by solvent or guest molecules.
- A full description of these two different surfaces and the ways in which they can be used is given in: L. J. Barbour, *Chem. Commun.* **2006**, 1163–1168.

The dimensions of the crystallograpic axes are given above the images to facilitate the estimate of the cross-sections of the pore apertures:

Aluminum fumarate, Alfum (CCDC 1051975) water removed, view along a, b to right, c down (a = 6.84 Å, b = 12.09 Å, c = 14.21 Å)

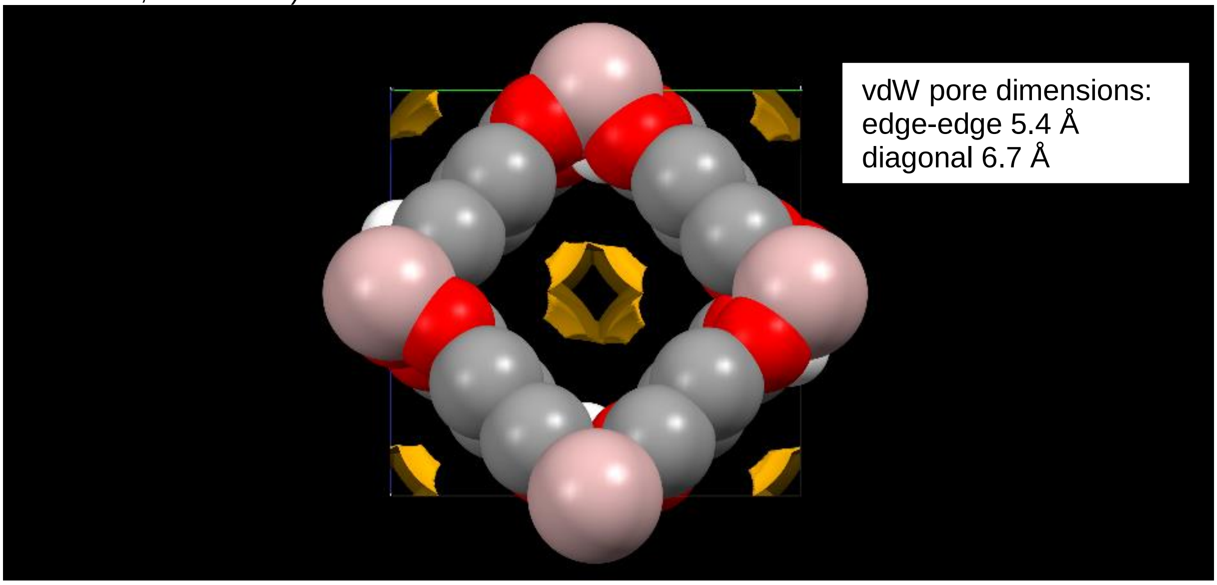

MIL-160 (CCDC 1828695), water removed, view along c, a right, b down (a = b = 21.07, c = 10.64 Å)

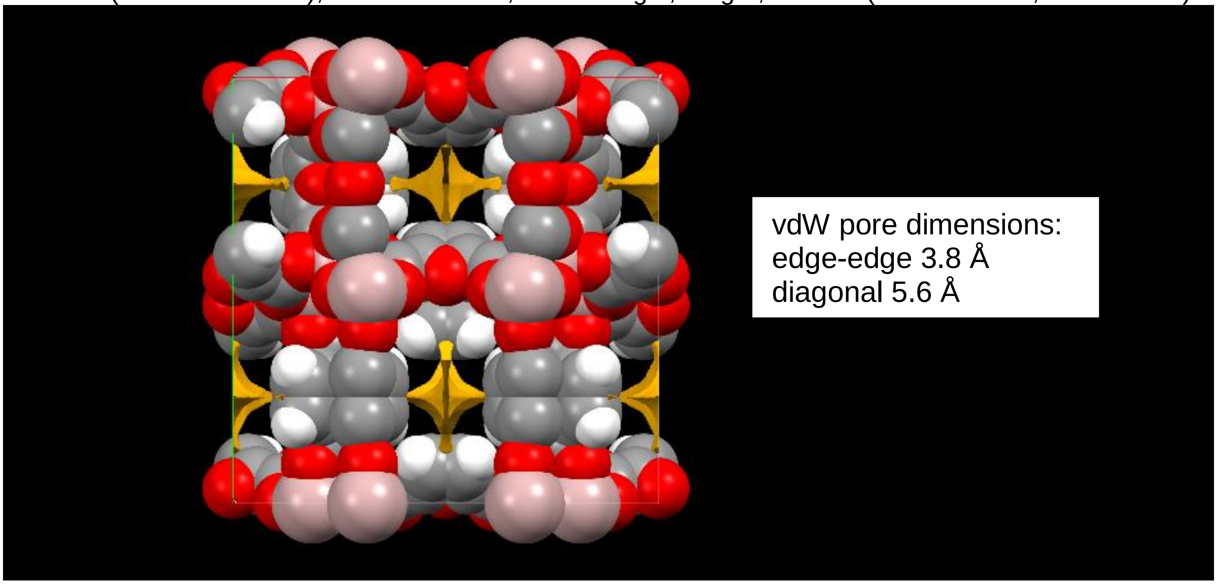

view along a, c right, b down (w. depth cuing)

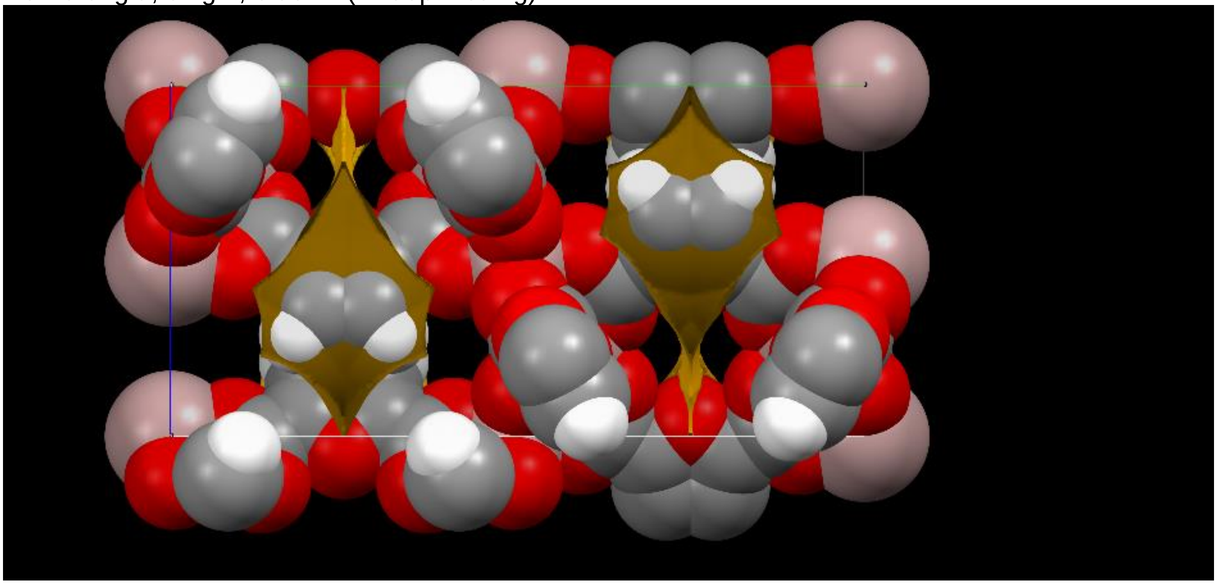

DUT-4 (CCDC 691978), view along b, c to right, a down: (a = 18.82, b = 6.7, c = 16.90 Å)

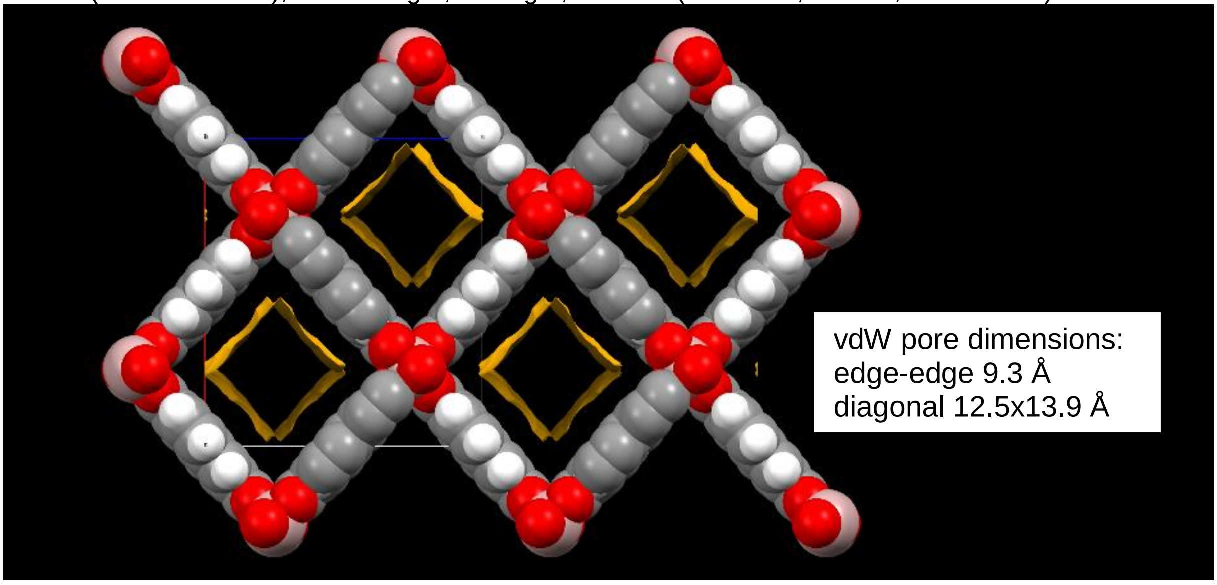

DUT-5 (CCDC 691979) along b, c to right, a down: (a = 22.70, b = 6.61, c = 19.24 Å)

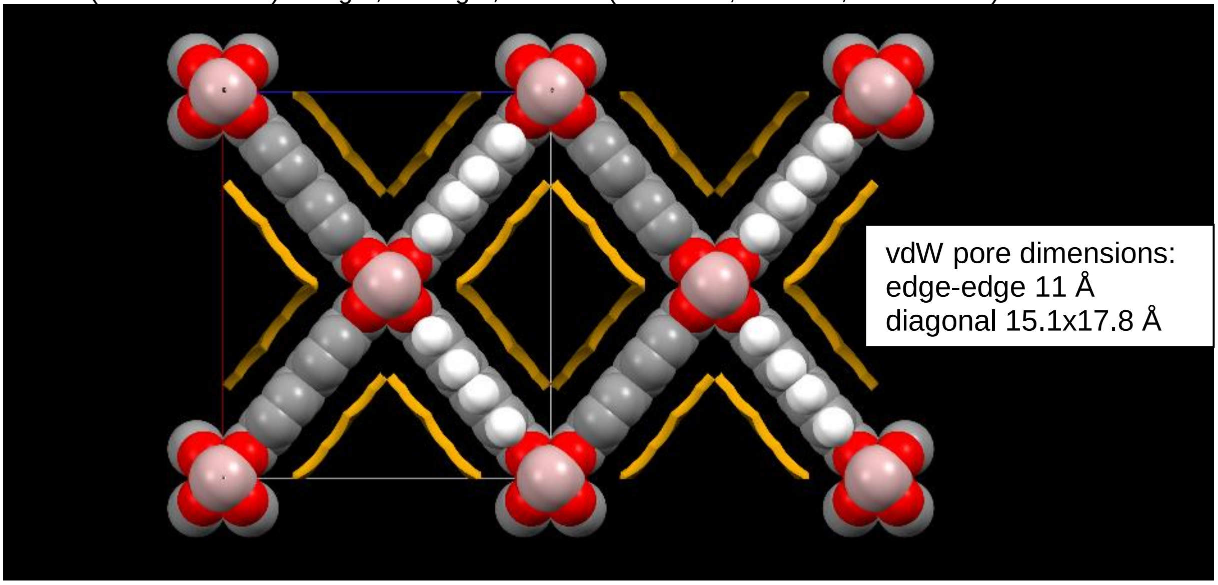

MIL-53-TDC, water removed, along b, c to right, a down: (a = 14.63, b = 6.56, c = 14.73 Å)

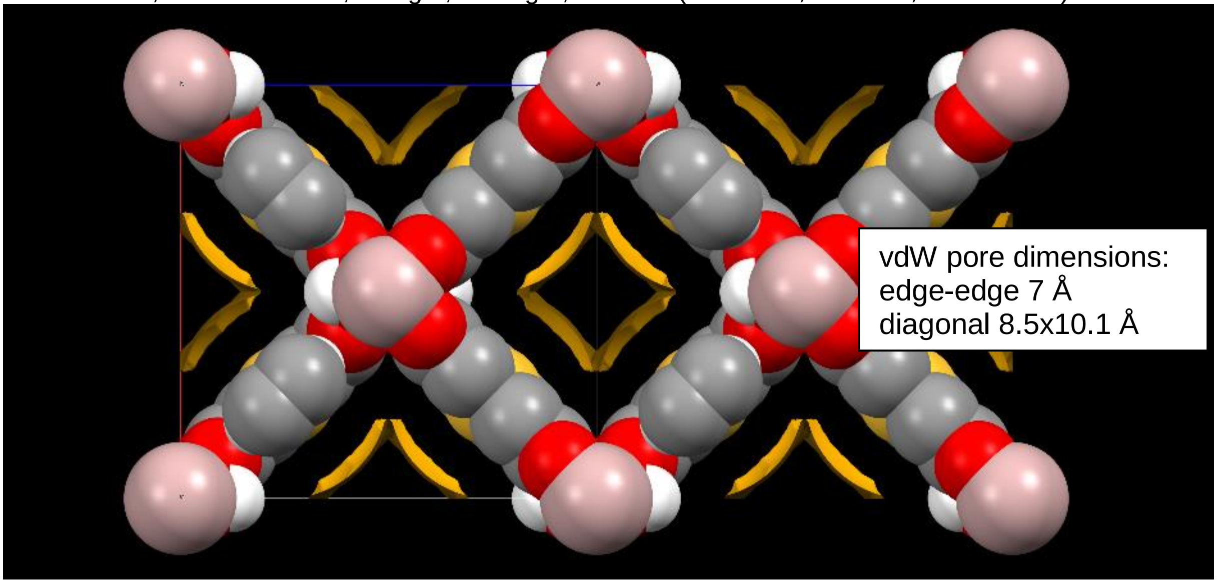

MIL-53(Al) as, (CCDC 220475), view along b, c up, a right (a = 17.13, b = 6.63, c = 12.18 Å)

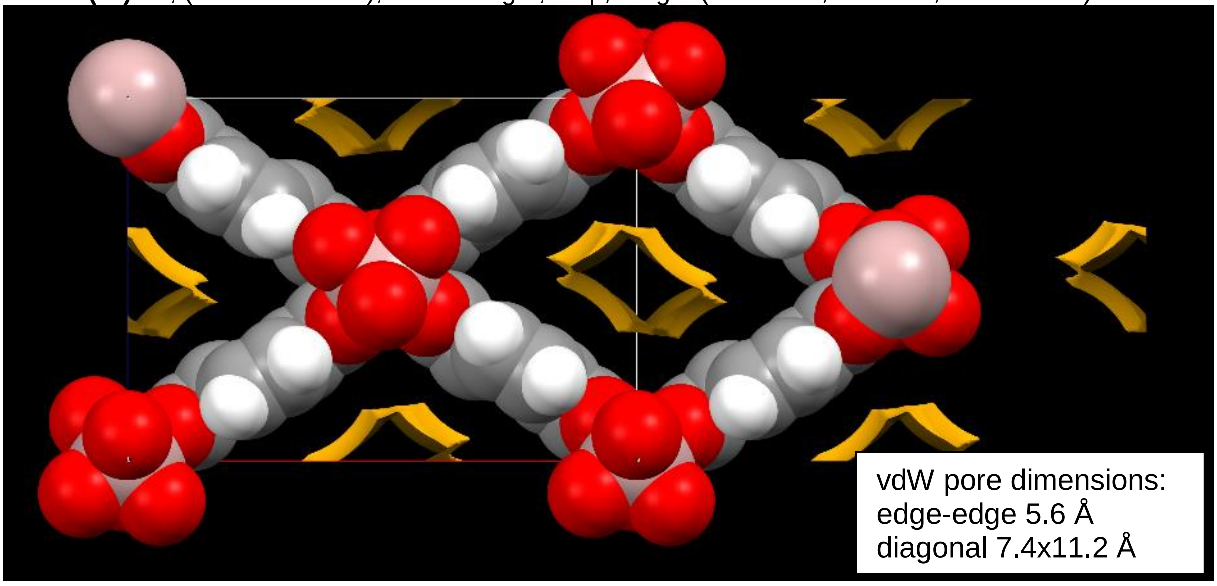

UiO-66 (CCDC 733458, view along b, a down, c right (a = b = c = 20.70 Å)
probe radius 2 Å:

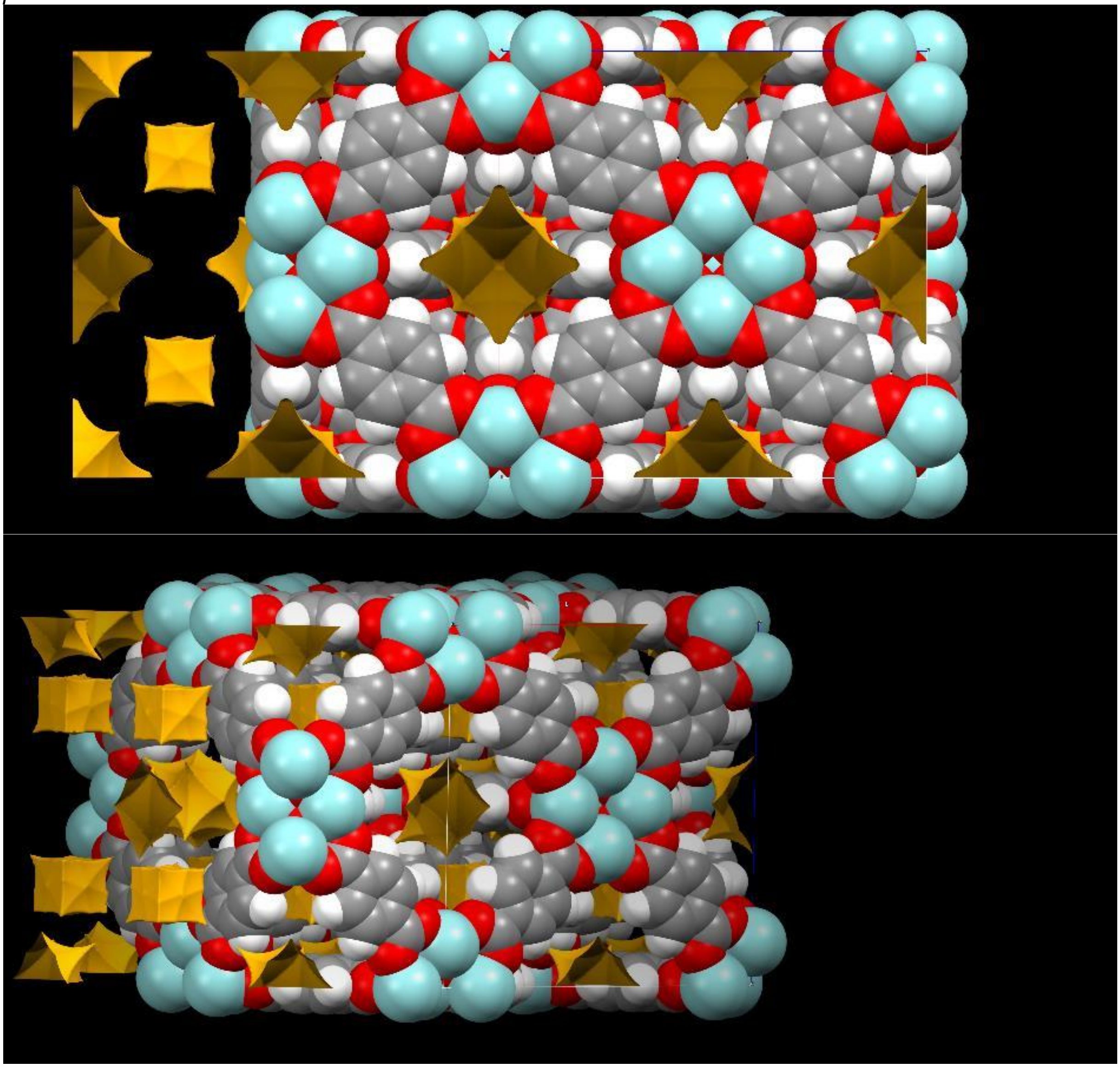

UiO-66 (CCDC 733458, view along b, a down, c right (a = b = c = 20.70 Å)
probe radius 1.5 Å:

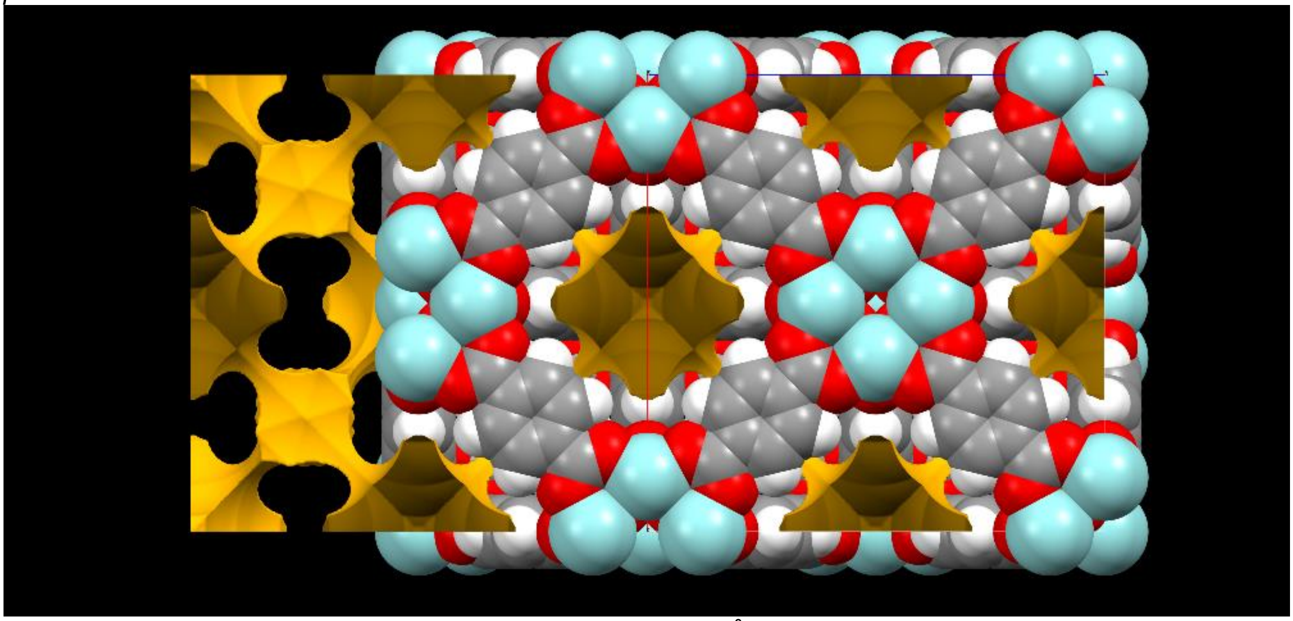

for measurement of channel cross-sections a = b = c = 20.70 Å:

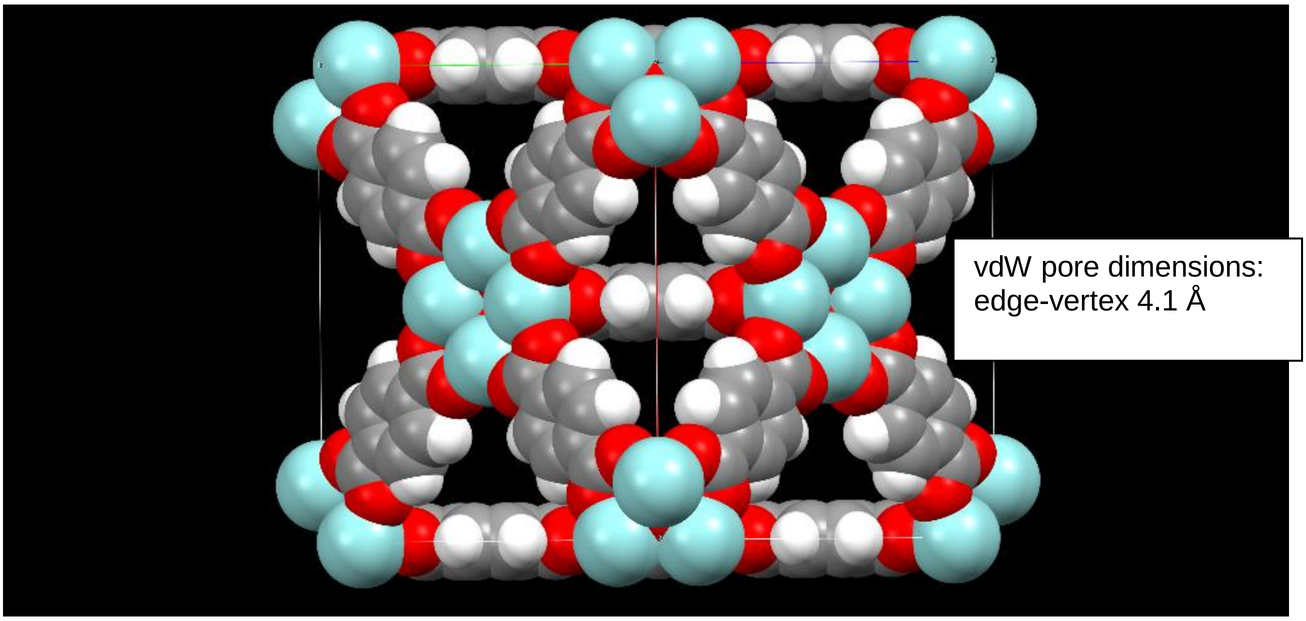

UiO-66-NH_2 (CCDC 1405751) (disordered O atom on Zr removed) view along b, a down, c right
(H atoms not added, because of N atom disorder, and thus, H atoms not shown)
probe radius 2 Å:

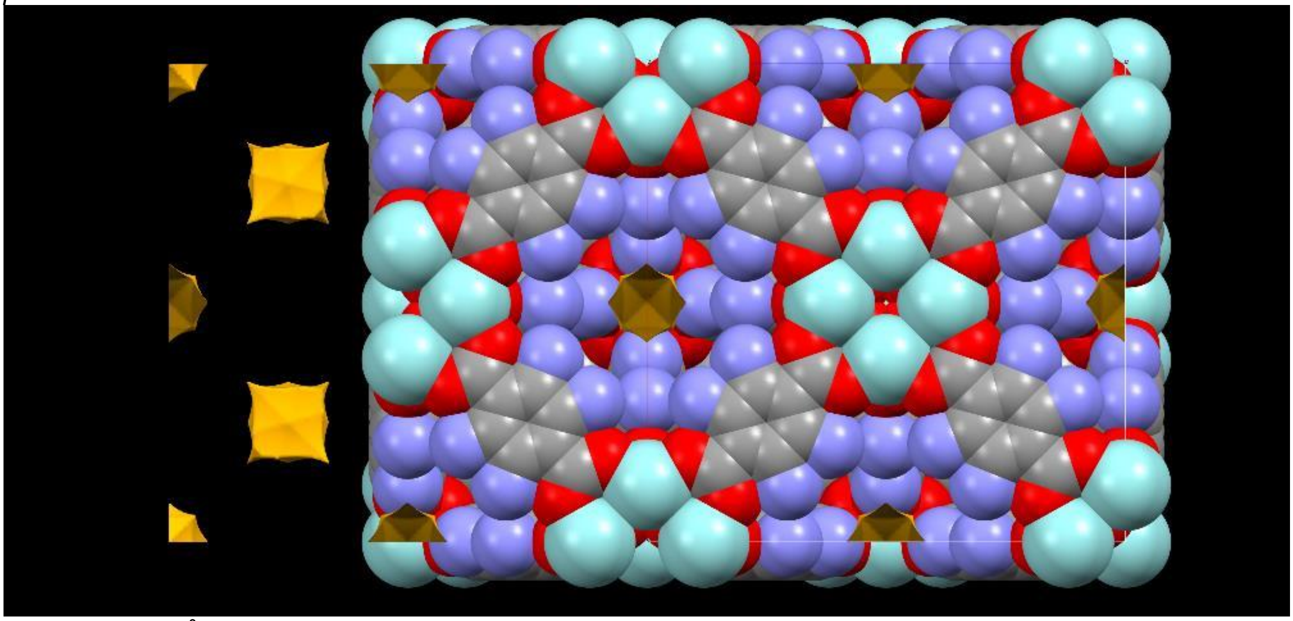

probe radius 1 Å:

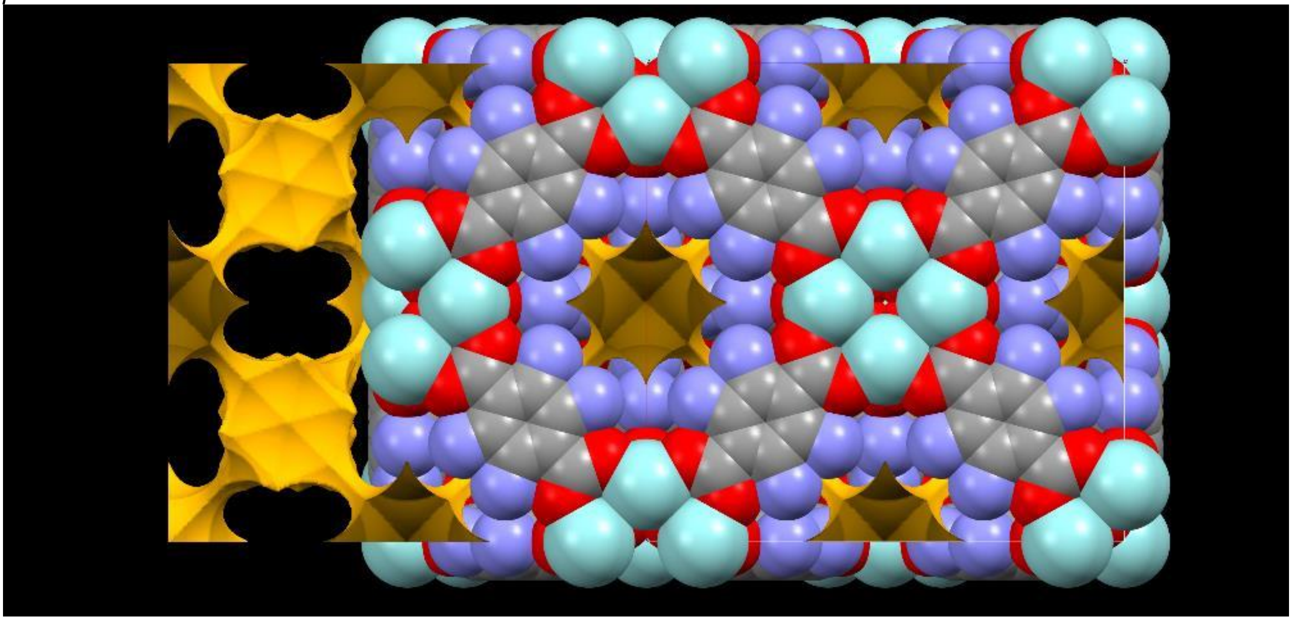

vdW pore dimensions: as in UiO-66
edge-vertex 4.1 Å
The N atoms are statistically oriented. Induced by the crystallographic symmetry, there is a ¼ N atom at each C atom. There will also be channel sections which are just lined by H atoms as in UiO-66.

UiO-66(F)$_4$ no X-ray structure, but isostructural to UiO-66-NH_2
The pore volume, pore aperture and images for the solvent accessible surface can be taken from the structure of UiO-66-"N" (above and below) with the crystallographically-induced N atom at each C atom of the benzene ring. (For measurement of channel cross-sections a = b = c = 20.80 Å).

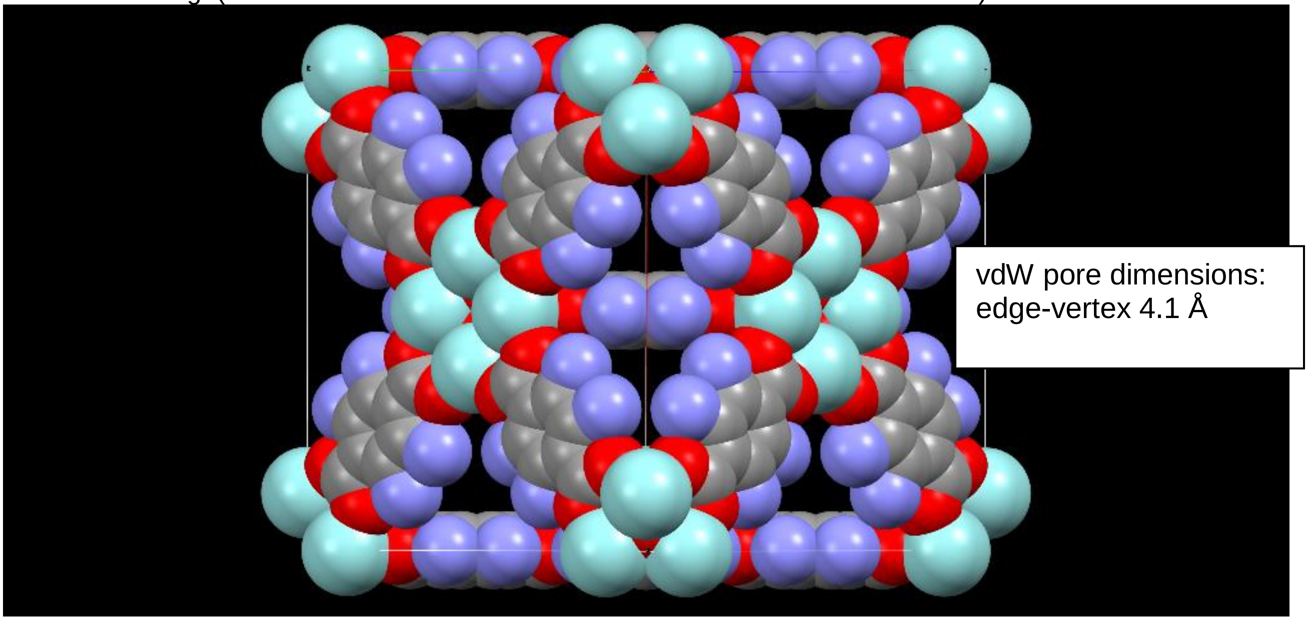

UiO-67 (CCDC 1018032) view along b, a down, c right (a = b = c = 26.9 Å)

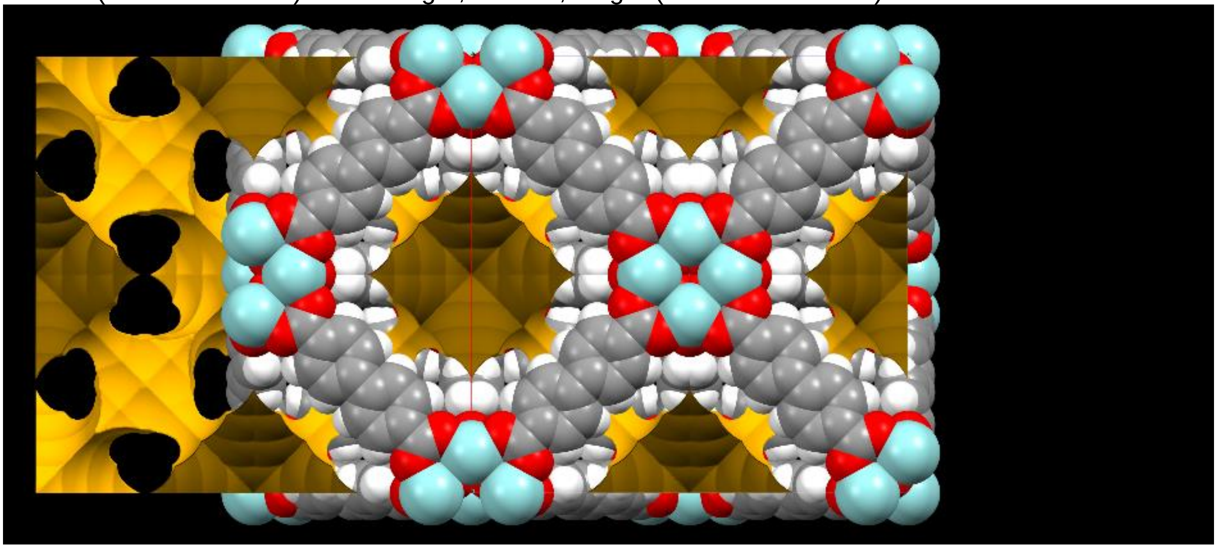

view along cube diagonal to show the connectivity between the pores:

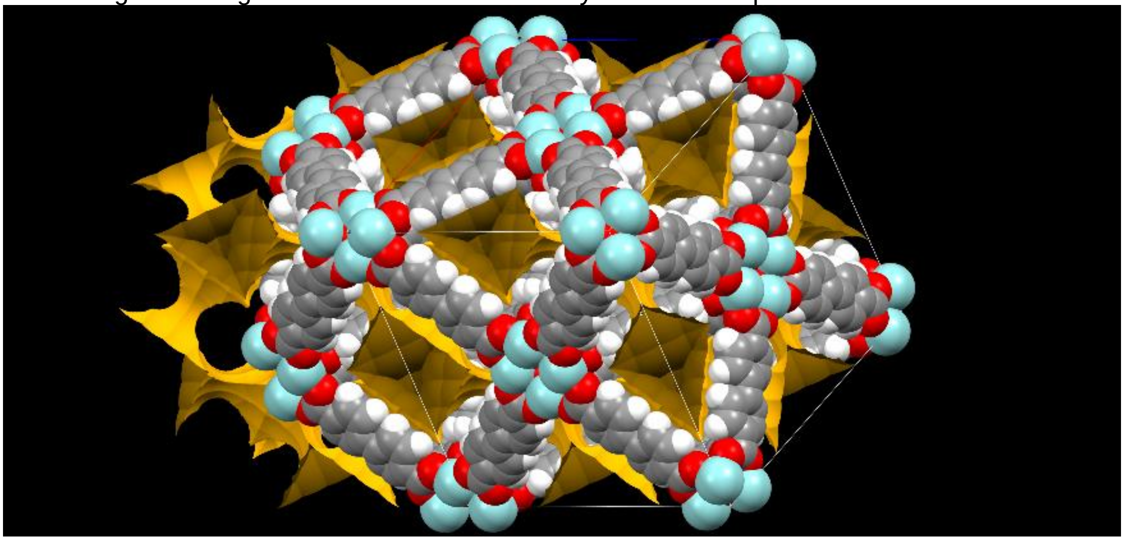

UiO-67 for measurement of channel cross-sections a = b = c = 26.9 Å

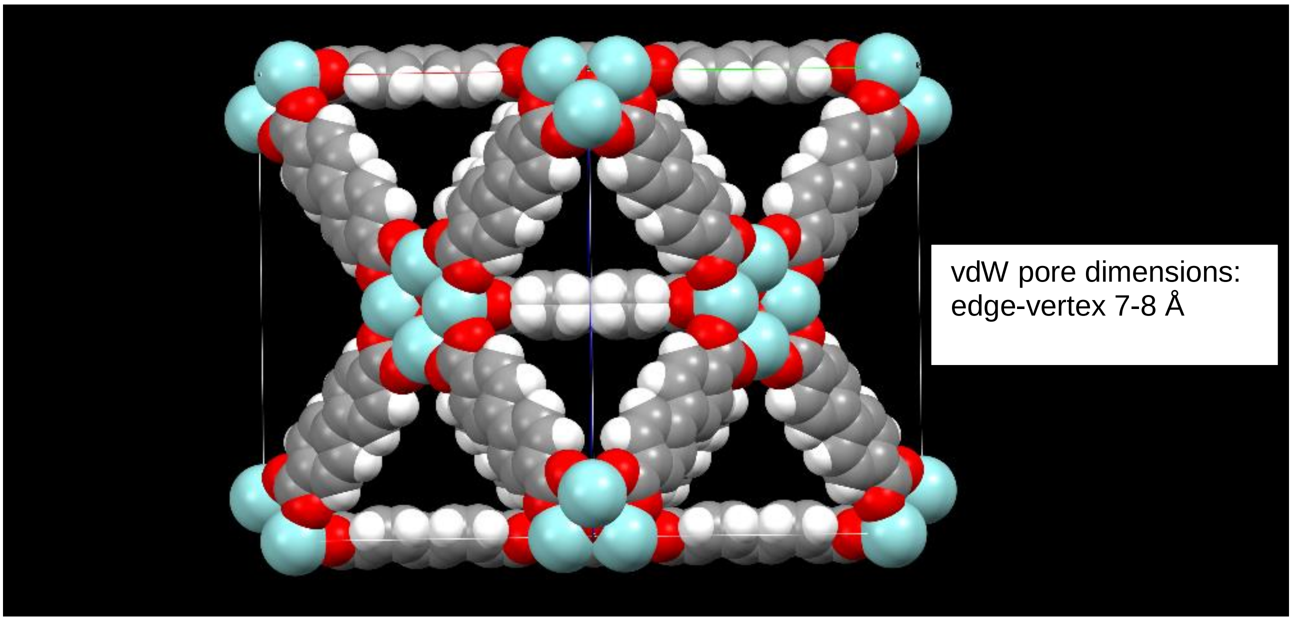

DUT-67 (CCDC 921644) along b, c to right, a down (a = b = c = 39.1 Å)

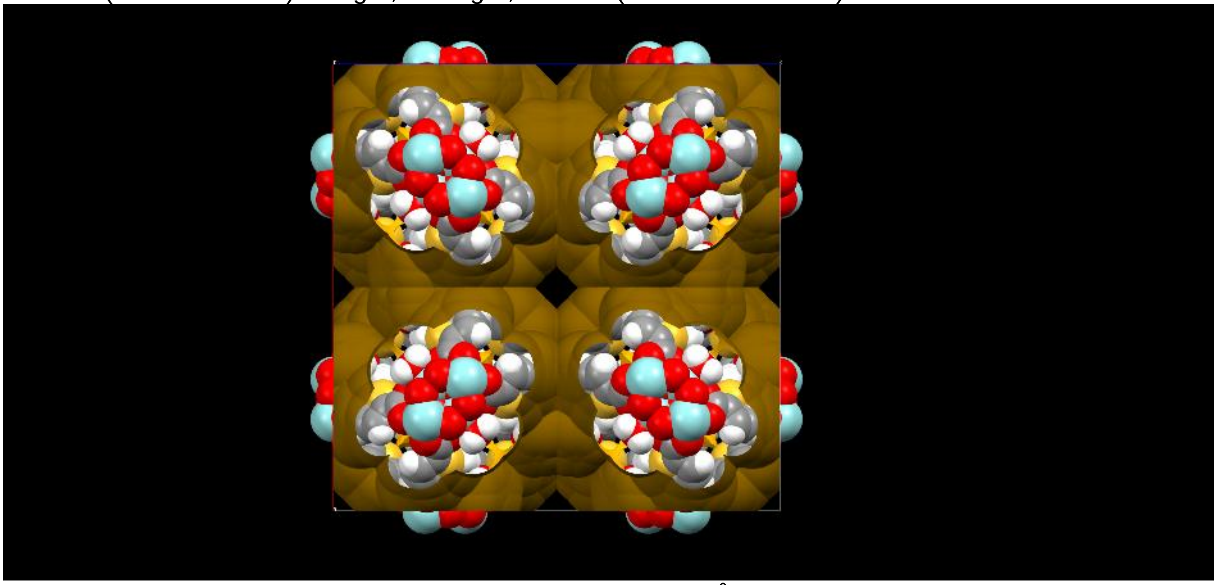

for measurement of channel cross-sections a = b = c = 39.1 Å:

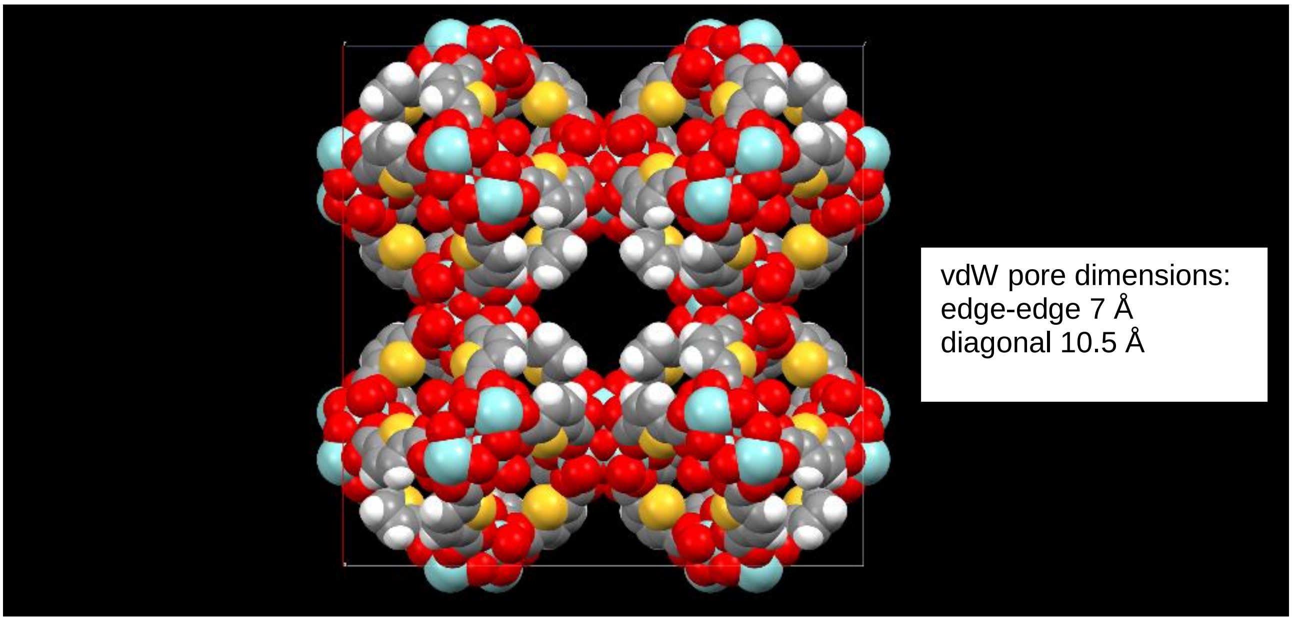

MIL-125 (CCDC 751157), along b, c to right, a down

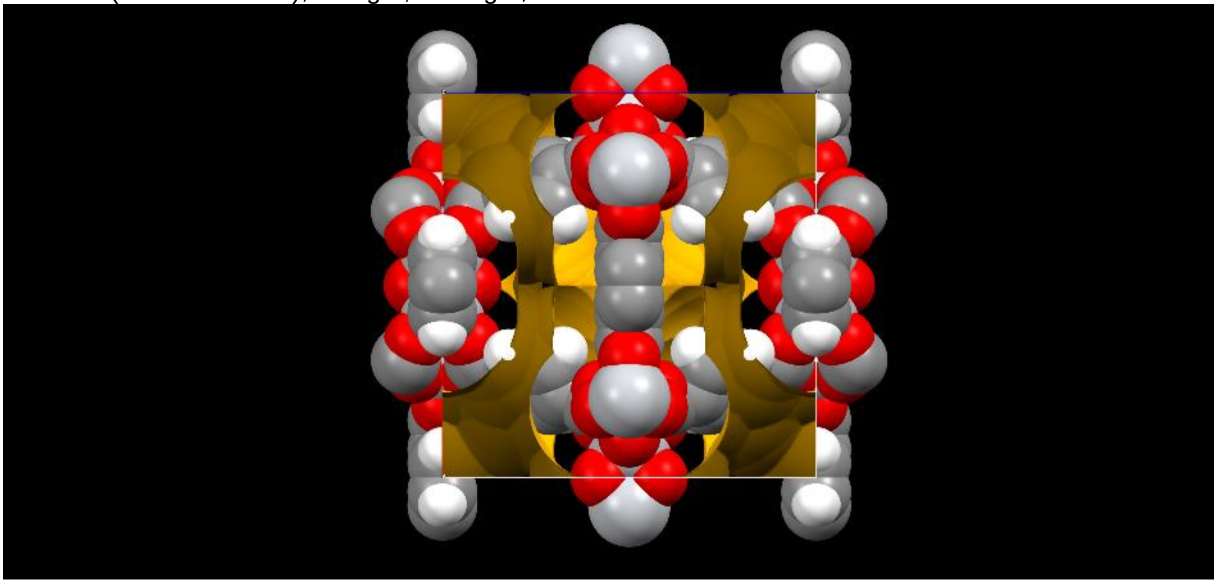

enlarged, along b, c to right, a down

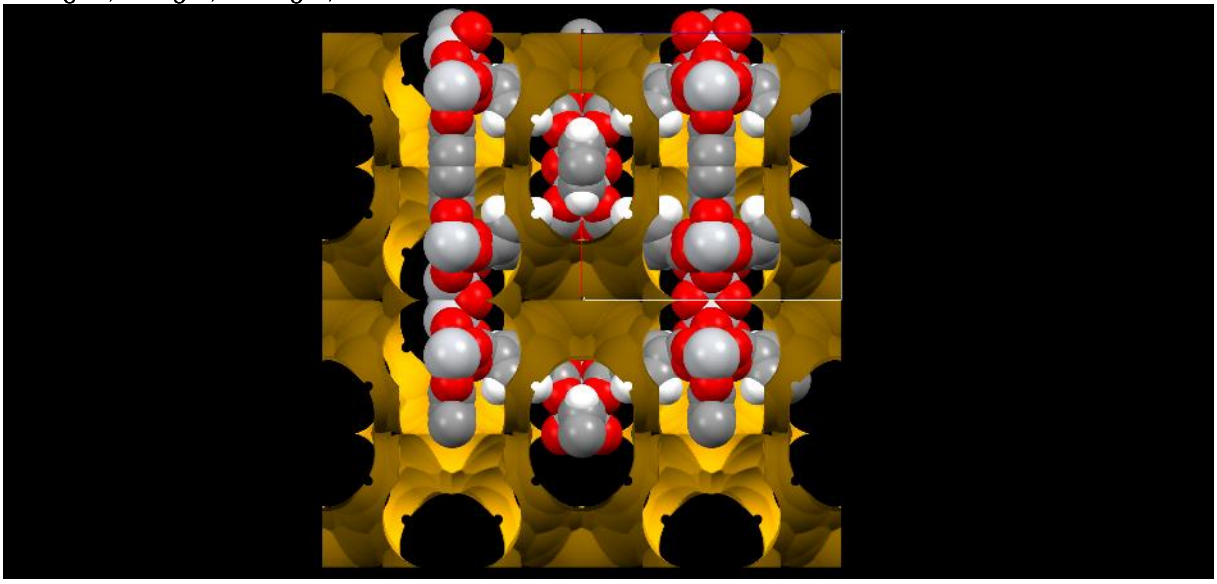

view along c, a to right, b down

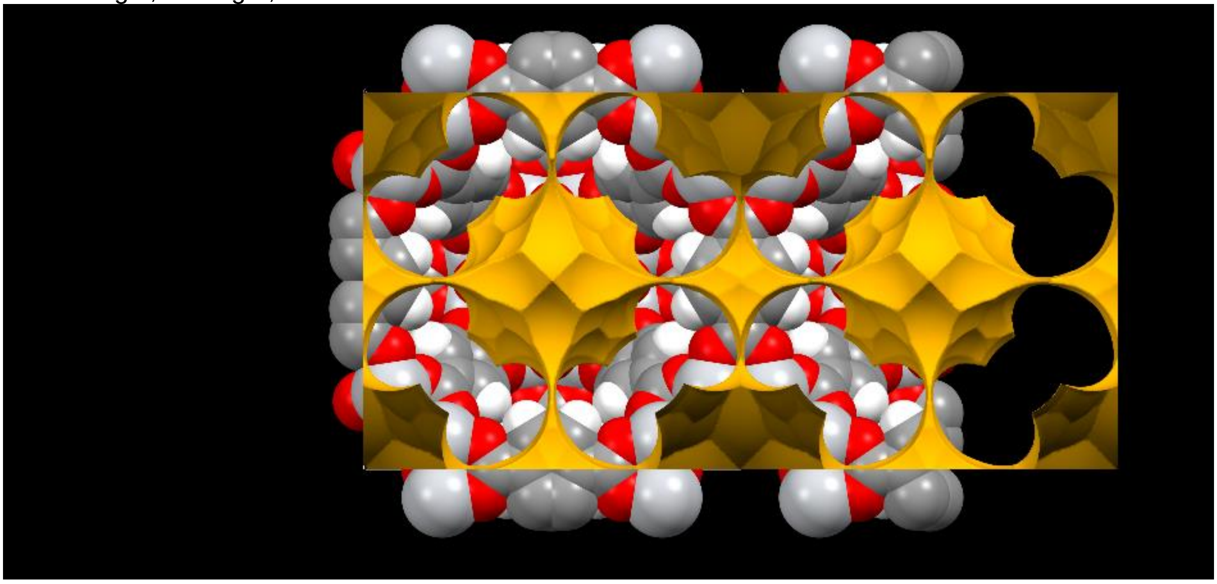

MIL-125 for measurement of channel cross-sections a = b = 18.65, c = 18.14 Å:

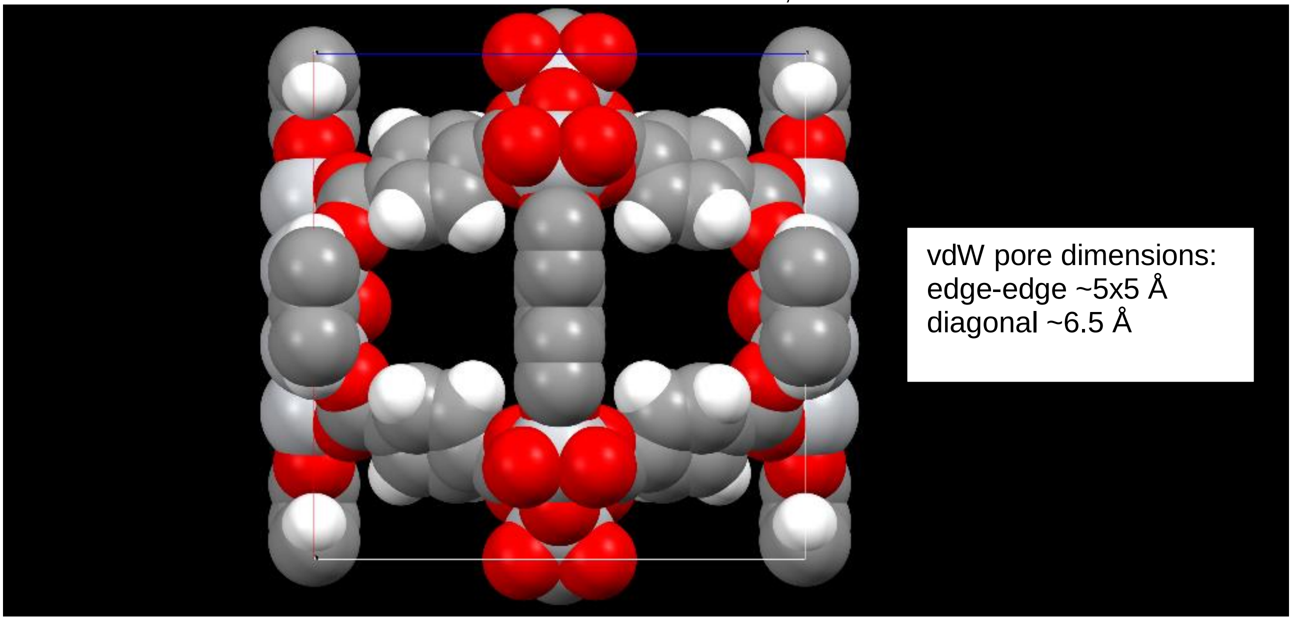

ZIF-8 (CCDC 864309), water removed, along b, c to right, a down (a = b = c = 16.85 Å)
probe radius 2 A:

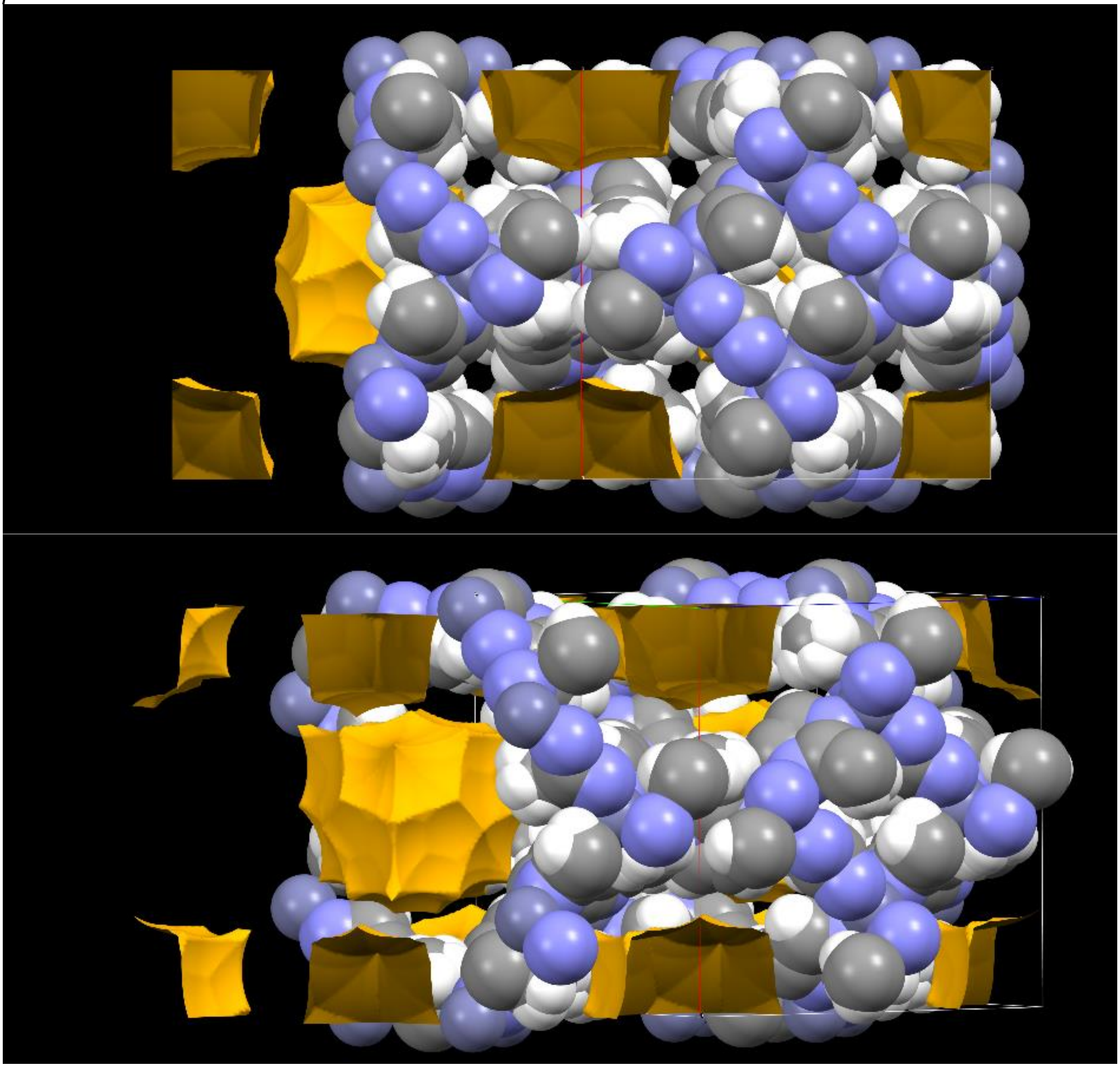

probe radius 1.5 Å – continuous channels:

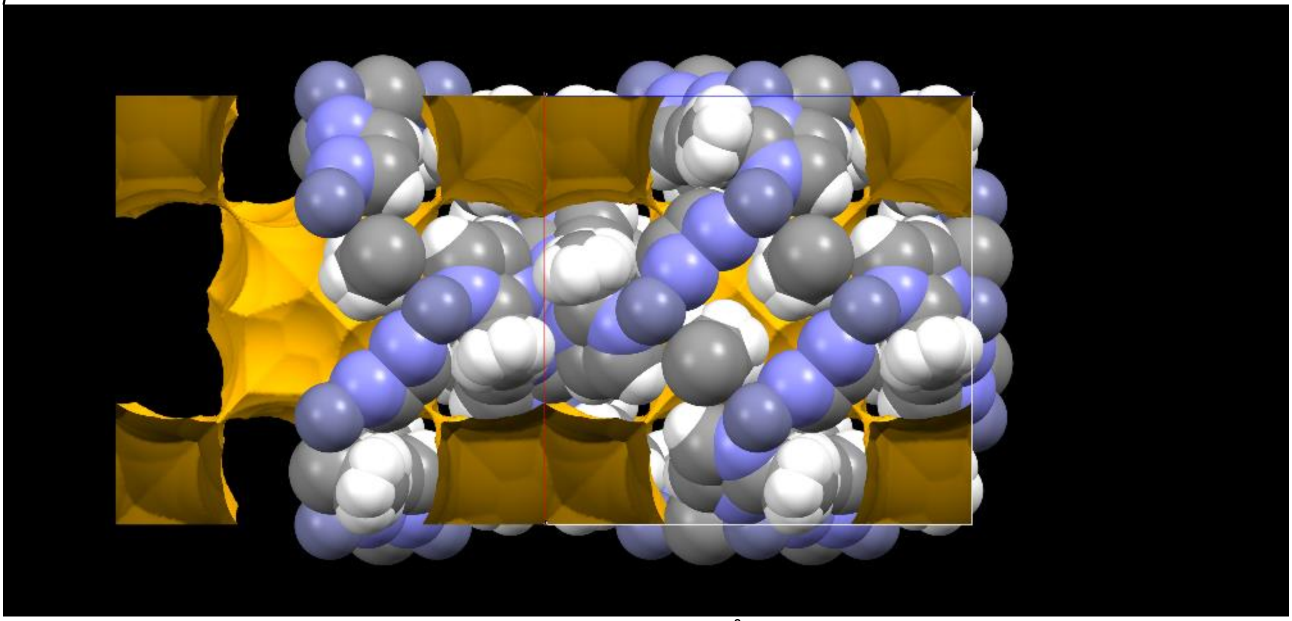

for measurement of channel cross-sections a = b = c = 16.85 Å:

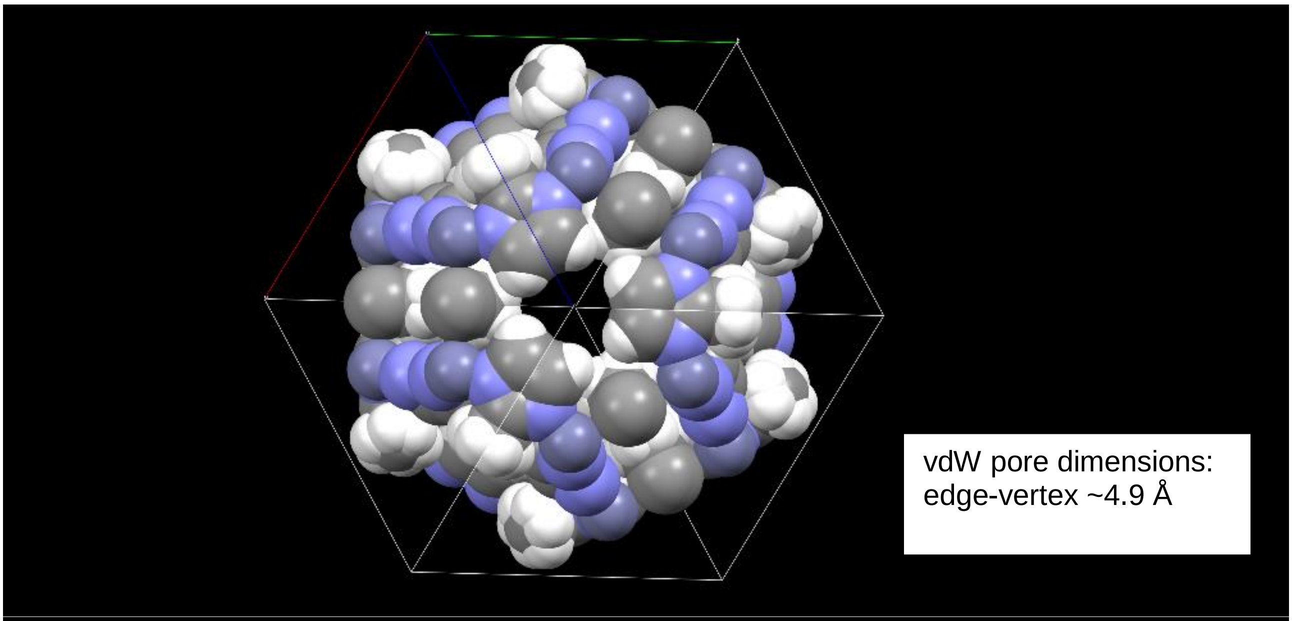

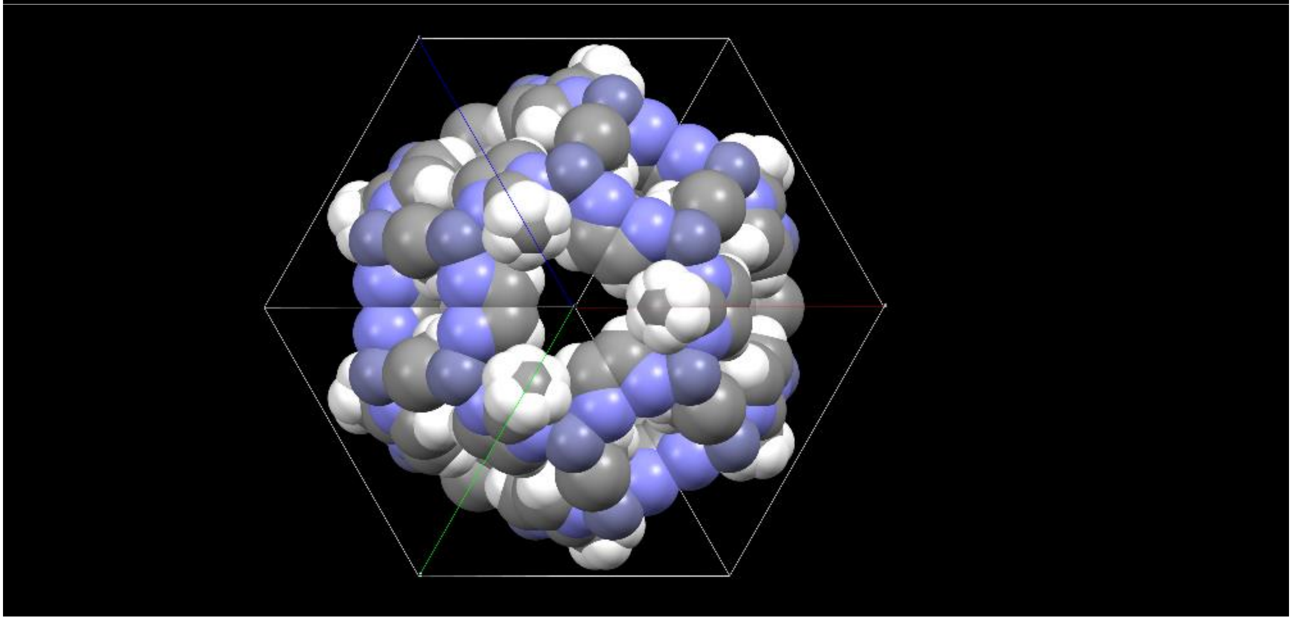

ZIF-11 (CCDC 602545), water removed, disorder removed, along b, c to right, a down (a = b = c = 28.76 Å)
probe radius 2 Å:

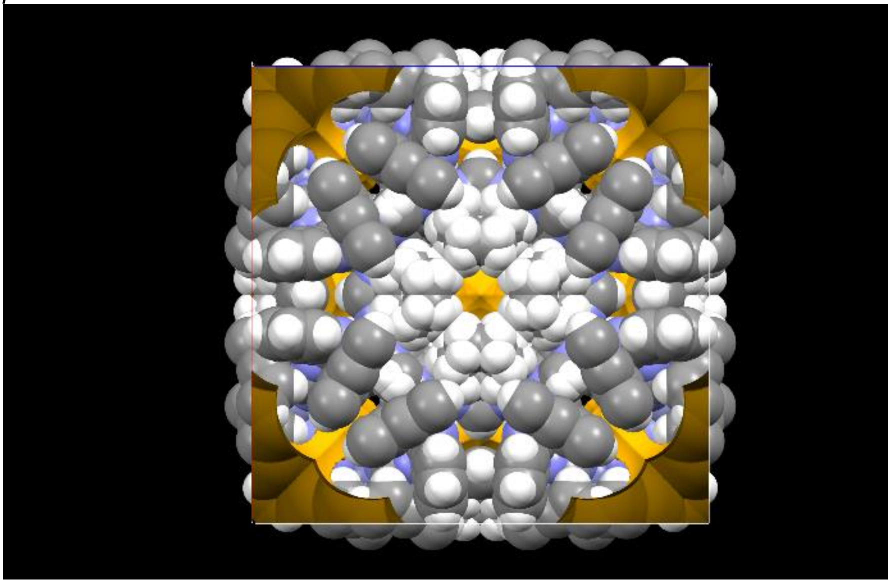

along cube diagonal:

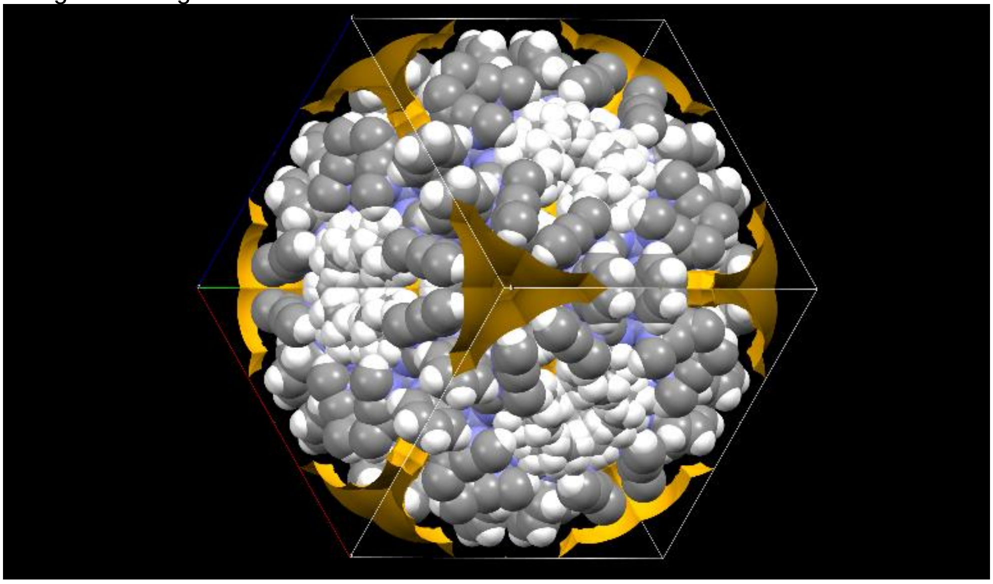

no continuous channels:

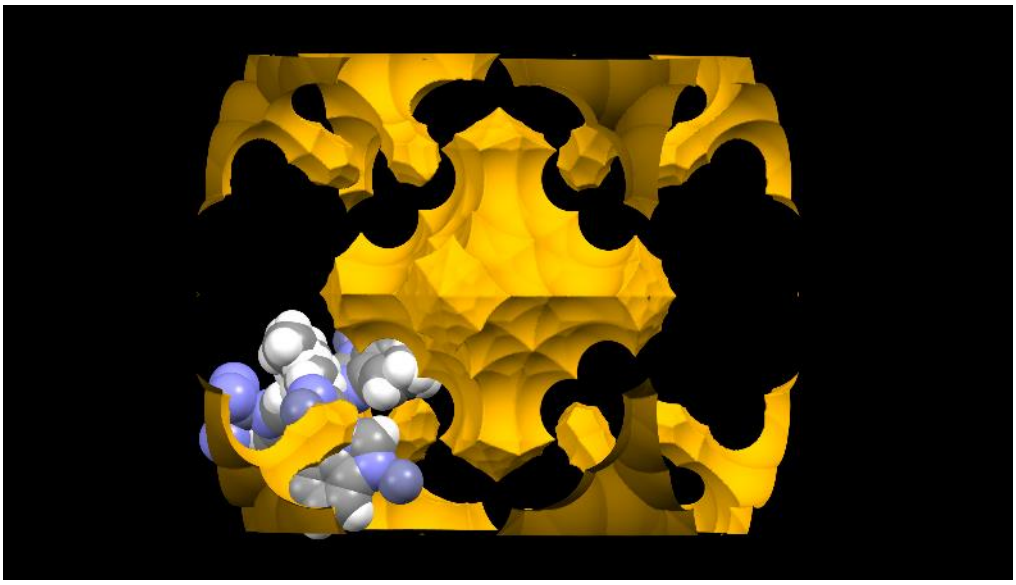

probe radius 1.5 Å, continuous channels:

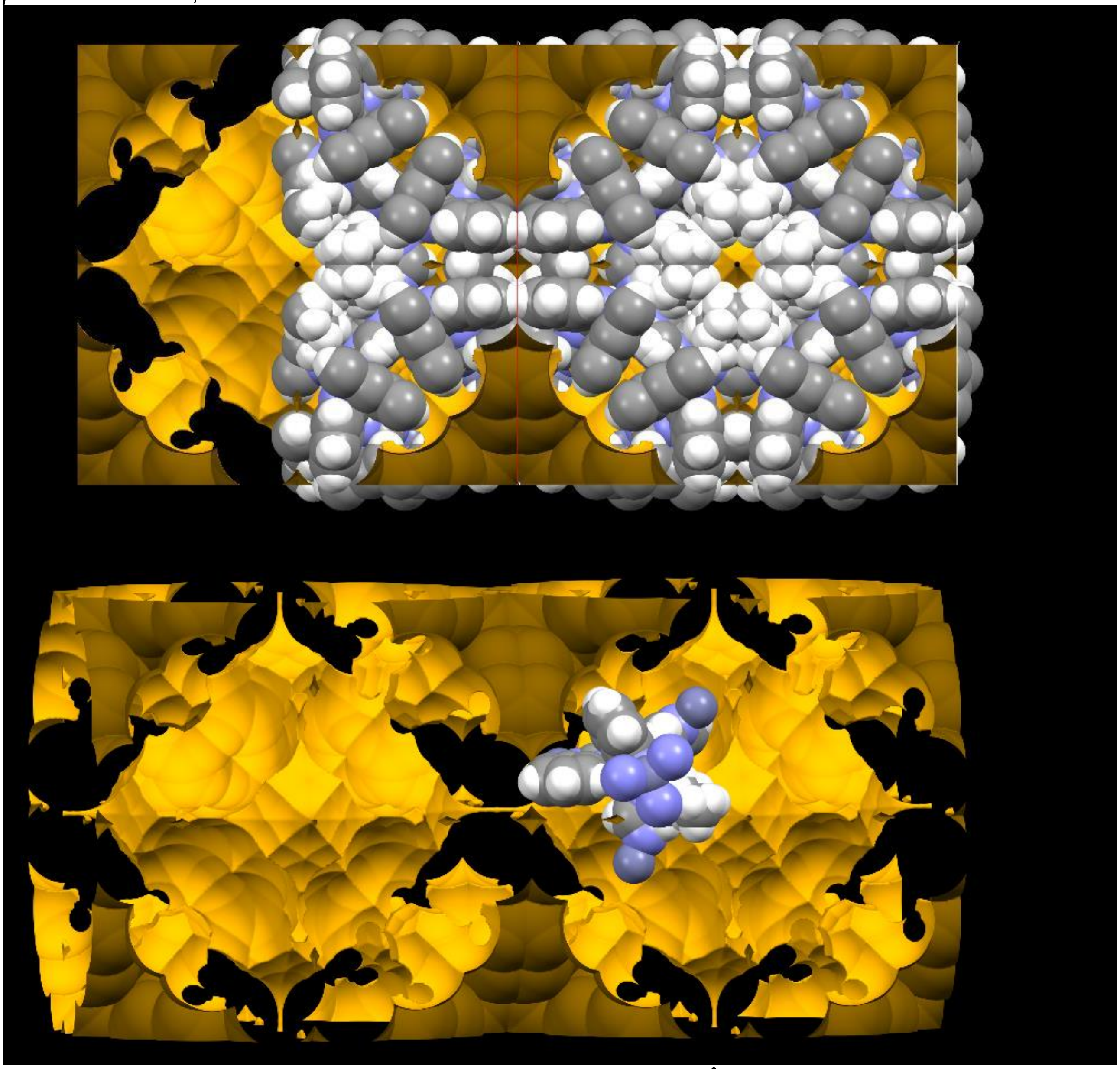

ZIF-11 for measurement of channel cross-sections a = b = c = 28.76 Å

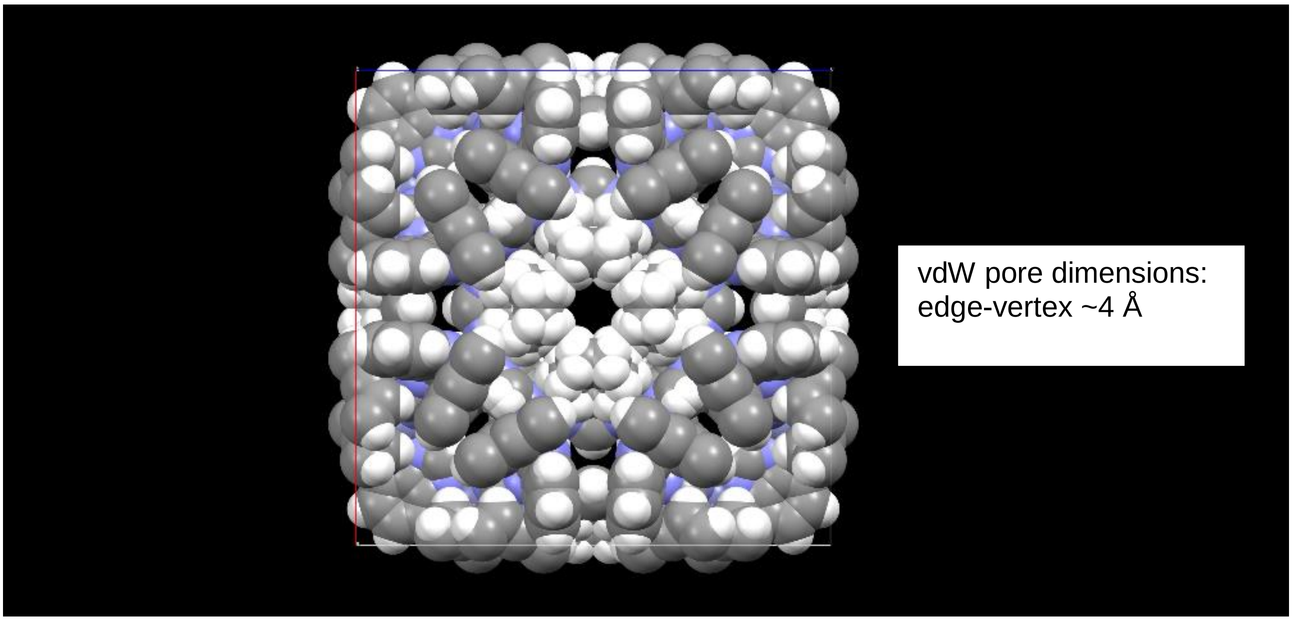

ZIF-7 (CCDC 973356), along b, c to right, a down (a = b = 22.94, c = 15.75 Å)
probe radius 2 Å:

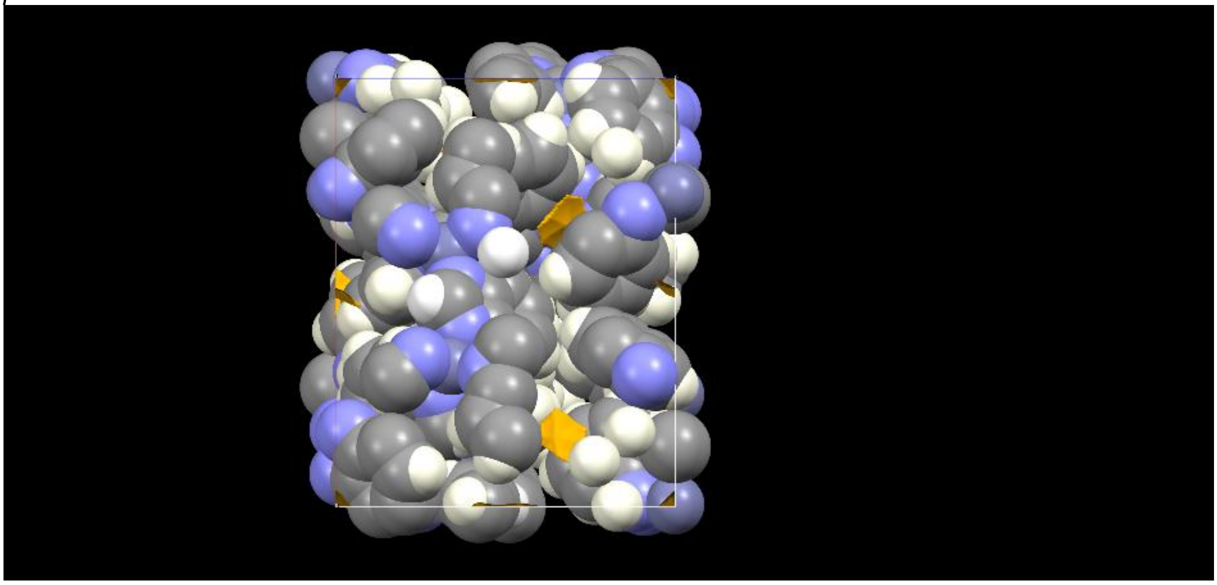

along c, a to right, b down:

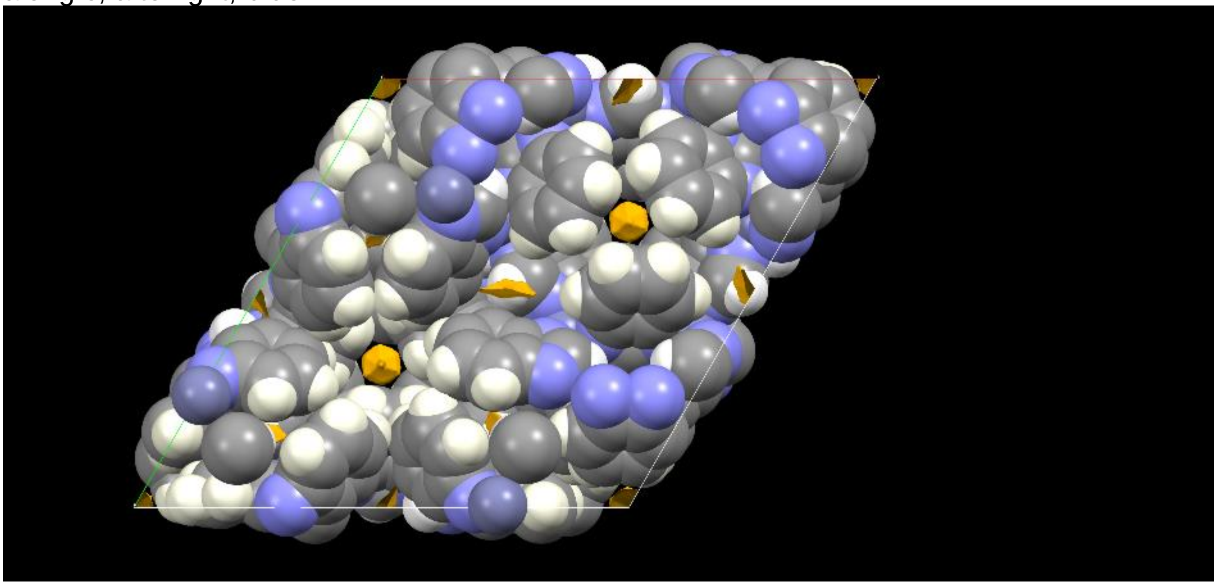

probe radius 1.5 Å, along b, c to right, a down:

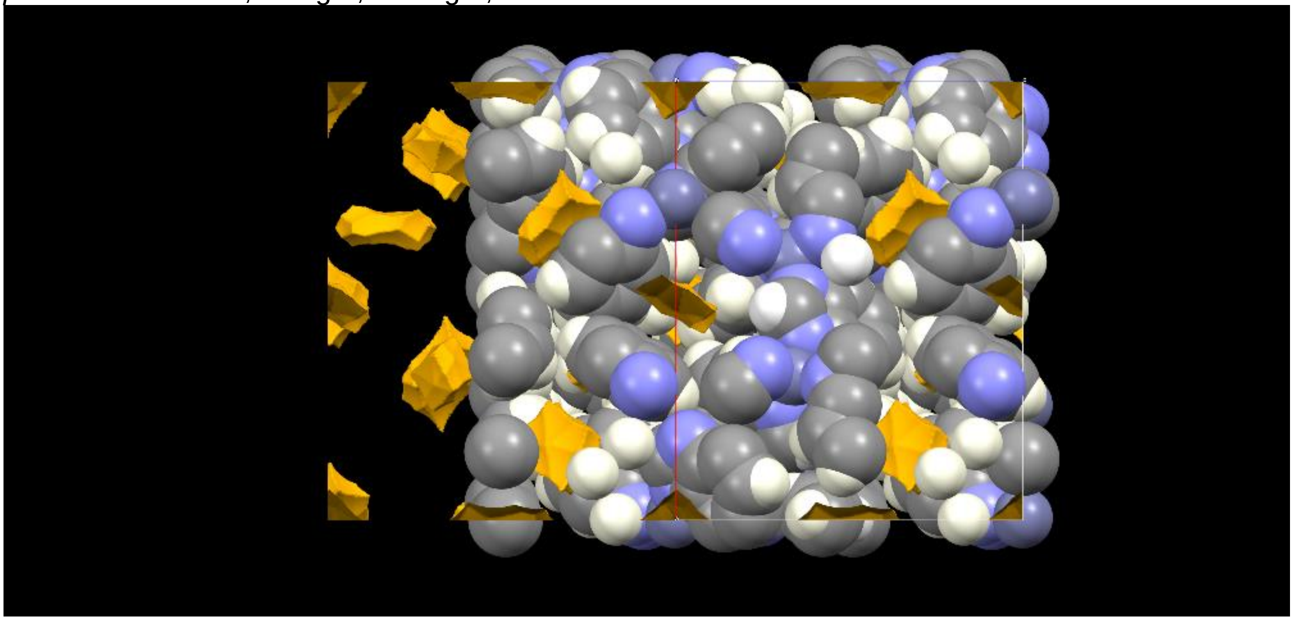

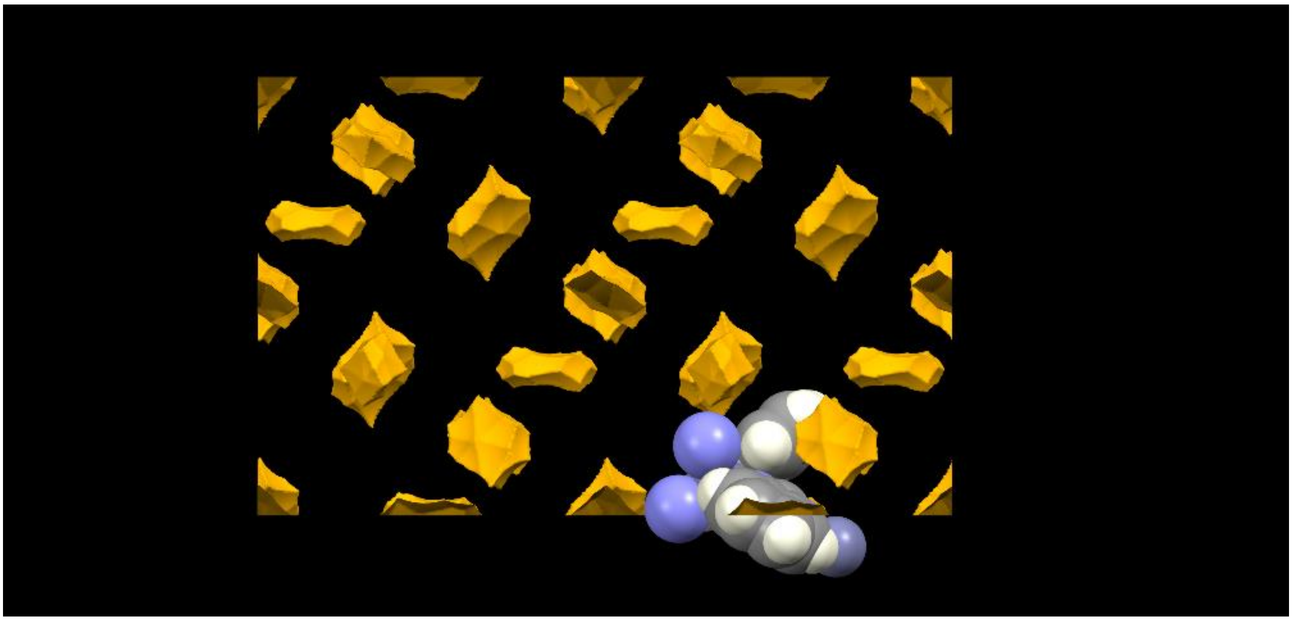

probe radius 1 Å, continuous channels:

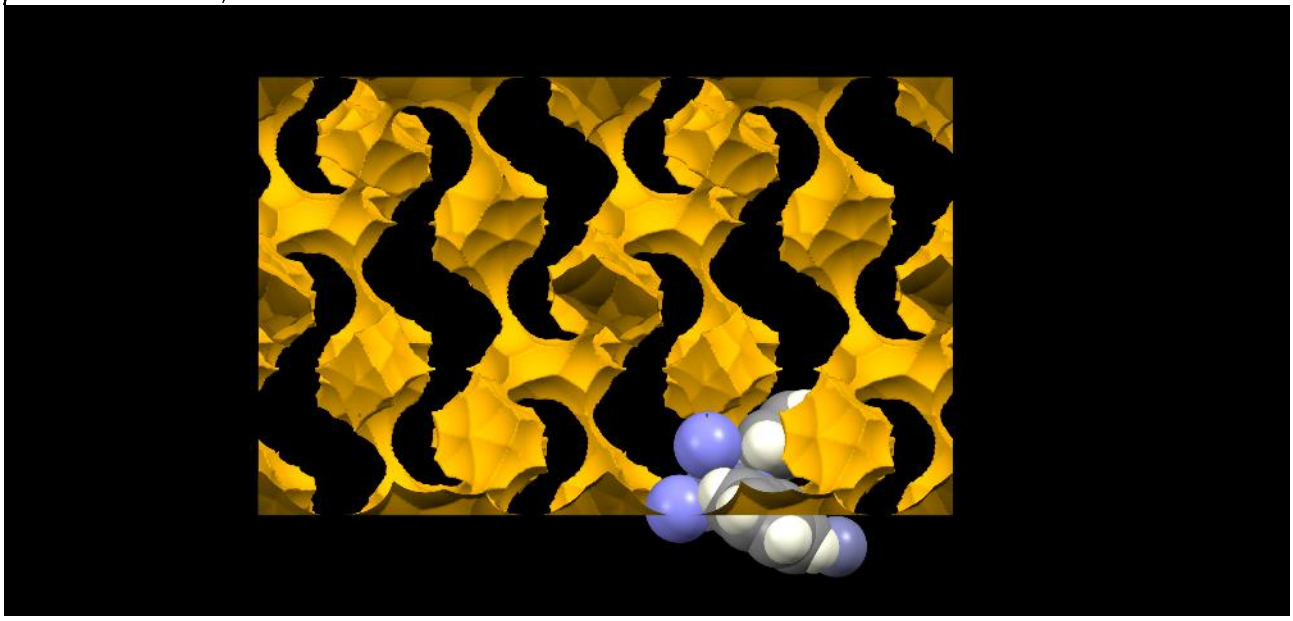

S16 References

[1] Alvarez, E.; Guillou, N.; Martineau, C.; Bueken, B.; Van de Voorde, B.; Le Guillouzer, C.; Fabry, P.; Nouar, F.; Taulelle, F.; Vos, D. de; Chang, J.-S.; Cho, K.H.; Ramsahye, N.; Devic, T.; Daturi, M.; Maurin, G.; Serre, C. The structure of the aluminum fumarate metal-organic framework A520. *Angew. Chem. Int. Ed.* **2015**, *54*, 3664–3668, doi:10.1002/anie.201410459.

[2] Brandenburg, K. Diamond 4.6, Crystal and Molecular Structure Visualization. Copyright 1997-2022 Crystal Impact GbR, Bon, Germany. Available online: https://www.crystalimpact.com/diamond/ (accessed on 10 August 2022).

[3] Cadiau, A.; Lee, J.S.; Damasceno Borges, D.; Fabry, P.; Devic, T.; Wharmby, M.T.; Martineau, C.; Foucher, D.; Taulelle, F.; Jun, C.-H.; Hwang, Y.K.; Stock, N.; Lange, M.F. de; Kapteijn, F.; Gascon, J.; Maurin, G.; Chang, J.-S.; Serre, C. Design of hydrophilic metal organic framework water adsorbents for heat reallocation. *Adv. Mater.* **2015**, *27*, 4775–4780, doi:10.1002/adma.201502418.

[4] Wahiduzzaman, M.; Lenzen, D.; Maurin, G.; Stock, N.; Wharmby, M.T. Rietveld Refinement of MIL-160 and Its Structural Flexibility Upon H_2O and N_2 Adsorption. *Eur. J. Inorg. Chem.* **2018**, 3626–3632, doi:10.1002/ejic.201800323.

[5] Senkovska, I.; Hoffmann, F.; Fröba, M.; Getzschmann, J.; Böhlmann, W.; Kaskel, S. New highly porous aluminium based metal-organic frameworks: Al(OH)(ndc) (ndc=2,6-naphthalene dicarboxylate) and Al(OH)(bpdc) (bpdc=4,4′-biphenyl dicarboxylate). *Microporous Mesoporous Mater.* **2009**, *122*, 93–98, doi:10.1016/j.micromeso.2009.02.020.

[6] Gotthardt, M.A.; Grosjean, S.; Brunner, T.S.; Kotzel, J.; Gänzler, A.M.; Wolf, S.; Bräse, S.; Kleist, W. Synthesis and post-synthetic modification of amine-, alkyne-, azide- and nitro-functionalized metal-organic frameworks based on DUT-5. *Dalton Trans.* **2015**, *44*, 16802–16809, doi:10.1039/c5dt02276b.

[7] Tannert, N.; Ernst, S.-J.; Jansen, C.; Bart, H.-J.; Henninger, S.K.; Janiak, C. Evaluation of the highly stable metal–organic framework MIL-53(Al)-TDC (TDC = 2,5-thiophenedicarboxylate) as a new and promising adsorbent for heat transformation applications. *J. Mater. Chem. A* **2018**, *6*, 17706–17712, doi:10.1039/C8TA04407D.

[8] Zi, G.; Yan, Z.; Wang, Y.; Chen, Y.; Guo, Y.; Yuan, F.; Gao, W.; Wang, Y.; Wang, J. Catalytic hydrothermal conversion of carboxymethyl cellulose to value-added chemicals over metal-organic framework MIL-53(Al). *Carbohydr. Polym.* **2015**, *115*, 146–151, doi:10.1016/j.carbpol.2014.08.065.

[9] Rallapalli, P.; Patil, D.; Prasanth, K.P.; Somani, R.S.; Jasra, R.V.; Bajaj, H.C. An alternative activation method for the enhancement of methane storage capacity of nanoporous aluminium terephthalate, MIL-53(Al). *J. Porous Mater.* **2010**, *17*, 523–528, doi:10.1007/s10934-009-9320-5.

[10] Ortiz, G.; Chaplais, G.; Paillaud, J.-L.; Nouali, H.; Patarin, J.; Raya, J.; Marichal, C. New Insights into the Hydrogen Bond Network in Al-MIL-53 and Ga-MIL-53. *J. Phys. Chem. C* **2014**, *118*, 22021–22029, doi:10.1021/jp505893s.

[11] Loiseau, T.; Serre, C.; Huguenard, C.; Fink, G.; Taulelle, F.; Henry, M.; Bataille, T.; Férey, G. A rationale for the large breathing of the porous aluminum terephthalate (MIL-53) upon hydration. *Chem. Eur. J.* **2004**, *10*, 1373–1382, doi:10.1002/chem.200305413.

[12] Aguilera-Sigalat, J.; Bradshaw, D. A colloidal water-stable MOF as a broad-range fluorescent pH sensor via post-synthetic modification. *Chem. Commun.* **2014**, *50*, 4711–4713, doi:10.1039/c4cc00659c.

[13] Hu, Z.; Peng, Y.; Kang, Z.; Qian, Y.; Zhao, D. A Modulated Hydrothermal (MHT) Approach for the Facile Synthesis of UiO-66-Type MOFs. *Inorg. Chem.* **2015**, *54*, 4862–4868, doi:10.1021/acs.inorgchem.5b00435.

[14] Katz, M.J.; Brown, Z.J.; Colón, Y.J.; Siu, P.W.; Scheidt, K.A.; Snurr, R.Q.; Hupp, J.T.; Farha, O.K. A facile synthesis of UiO-66, UiO-67 and their derivatives. *Chem. Commun.* **2013**, *49*, 9449–9451, doi:10.1039/c3cc46105j.

[15] Cavka, J.H.; Jakobsen, S.; Olsbye, U.; Guillou, N.; Lamberti, C.; Bordiga, S.; Lillerud, K.P. A new zirconium inorganic building brick forming metal organic frameworks with exceptional stability. *J. Am. Chem. Soc* **2008**, *130*, 13850–13851, doi:10.1021/ja8057953.

[16] Drache, F.; Bon, V.; Senkovska, I.; Marschelke, C.; Synytska, A.; Kaskel, S. Postsynthetic Inner-Surface Functionalization of the Highly Stable Zirconium-Based Metal-Organic Framework DUT-67. *Inorg. Chem.* **2016**, *55*, 7206–7213, doi:10.1021/acs.inorgchem.6b00829.

[17] Bon, V.; Senkovska, I.; Baburin, I.A.; Kaskel, S. Zr- and Hf-Based Metal–Organic Frameworks: Tracking Down the Polymorphism. *Cryst. Growth. Des.* **2013**, *13*, 1231–1237, doi:10.1021/cg301691d.

[18] Sohail, M.; Yun, Y.-N.; Lee, E.; Kim, S.K.; Cho, K.; Kim, J.-N.; Kim, T.W.; Moon, J.-H.; Kim, H. Synthesis of Highly Crystalline NH_2-MIL-125 (Ti) with S-Shaped Water Isotherms for Adsorption Heat Transformation. *Cryst. Growth. Des.* **2017**, *17*, 1208–1213, doi:10.1021/acs.cgd.6b01597.

[19] Santaclara, J.G.; Nasalevich, M.A.; Castellanos, S.; Evers, W.H.; Spoor, F.C.M.; Rock, K.; Siebbeles, L.D.A.; Kapteijn, F.; Grozema, F.; Houtepen, A.; Gascon, J.; Hunger, J.; van der Veen, M.A. Organic Linker Defines the Excited-State Decay of Photocatalytic MIL-125(Ti)-Type Materials. *ChemSusChem.* **2016**, *9*, 388–395, doi:10.1002/cssc.201501353.

[20] Dan-Hardi, M.; Serre, C.; Frot, T.; Rozes, L.; Maurin, G.; Sanchez, C.; Férey, G. A new photoactive crystalline highly porous titanium(IV) dicarboxylate. *J. Am. Chem. Soc* **2009**, *131*, 10857–10859, doi:10.1021/ja903726m.
[21] Zhao, T.; Jeremias, F.; Boldog, I.; Nguyen, B.; Henninger, S.K.; Janiak, C. High-yield, fluoride-free and large-scale synthesis of MIL-101(Cr). *Dalton Trans.* **2015**, *44*, 16791–16801, doi:10.1039/c5dt02625c.
[22] Férey, G.; Mellot-Draznieks, C.; Serre, C.; Millange, F.; Dutour, J.; Surblé, S.; Margiolaki, I. A chromium terephthalate-based solid with unusually large pore volumes and surface area. *Science* **2005**, *309*, 2040–2042, doi:10.1126/science.1116275.
[23] Kida, K.; Okita, M.; Fujita, K.; Tanaka, S.; Miyake, Y. Formation of high crystalline ZIF-8 in an aqueous solution. *CrystEngComm.* **2013**, *15*, 1794–1801, doi:10.1039/c2ce26847g.
[24] Morris, W.; Stevens, C.J.; Taylor, R.E.; Dybowski, C.; Yaghi, O.M.; Garcia-Garibay, M.A. NMR and X-ray Study Revealing the Rigidity of Zeolitic Imidazolate Frameworks. *J. Phys. Chem. C* **2012**, *116*, 13307–13312, doi:10.1021/jp303907p.
[25] He, M.; Yao, J.; Liu, Q.; Zhong, Z.; Wang, H. Toluene-assisted synthesis of RHO-type zeolitic imidazolate frameworks: synthesis and formation mechanism of ZIF-11 and ZIF-12. *Dalton Trans.* **2013**, *42*, 16608–16613, doi:10.1039/c3dt52103f.
[26] Park, K.S.; Ni, Z.; Côté, A.P.; Choi, J.Y.; Huang, R.; Uribe-Romo, F.J.; Chae, H.K.; O'Keeffe, M.; Yaghi, O.M. Exceptional chemical and thermal stability of zeolitic imidazolate frameworks. *Proc. Natl. Acad. Sci. USA* **2006**, *103*, 10186–10191, doi:10.1073/pnas.0602439103.
[27] Kang, C.-H.; Lin, Y.-F.; Huang, Y.-S.; Tung, K.-L.; Chang, K.-S.; Chen, J.-T.; Hung, W.-S.; Lee, K.-R.; Lai, J.-Y. Synthesis of ZIF-7/chitosan mixed-matrix membranes with improved separation performance of water/ethanol mixtures. *J. Membr. Sci.* **2013**, *438*, 105–111, doi:10.1016/j.memsci.2013.03.028.
[28] Li, Y.; Liang, F.; Bux, H.; Yang, W.; Caro, J. Zeolitic imidazolate framework ZIF-7 based molecular sieve membrane for hydrogen separation. *J. Membr. Sci.* **2010**, *354*, 48–54, doi:10.1016/j.memsci.2010.02.074.
[29] Tschense, C.B.L.; Reimer, N.; Hsu, C.-W.; Reinsch, H.; Siegel, R.; Chen, W.-J.; Lin, C.-H.; Cadiau, A.; Serre, C.; Senker, J.; Stock, N. New Group 13 MIL-53 Derivates based on 2,5-Thiophenedicarboxylic Acid. *Z. Anorg. Allg. Chem.* **2017**, *643*, 1600–1608, doi:10.1002/zaac.201700260.
[30] Lin, Z.-J.; Zheng, H.-Q.; Zeng, Y.-N.; Wang, Y.-L.; Chen, J.; Cao, G.-J.; Gu, J.-F.; Chen, B. Effective and selective adsorption of organoarsenic acids from water over a Zr-based metal-organic framework. *Chem. Eng. J.* **2019**, *378*, 122196, doi:10.1016/j.cej.2019.122196.
[31] Chen, D.-L.; Wu, S.; Yang, P.; He, S.; Dou, L.; Wang, F.-F. Ab Initio Molecular Dynamic Simulations on Pd Clusters Confined in UiO-66-NH_2. *J. Phys. Chem. C* **2017**, *121*, 8857–8863, doi:10.1021/acs.jpcc.7b00957.
[32] Reinsch, H.; Bueken, B.; Vermoortele, F.; Stassen, I.; Lieb, A.; Lillerud, K.-P.; Vos, D. de. Green synthesis of zirconium-MOFs. *CrystEngComm.* **2015**, *17*, 4070–4074, doi:10.1039/C5CE00618J.
[33] Kim, S.-N.; Kim, J.; Kim, H.-Y.; Cho, H.-Y.; Ahn, W.-S. Adsorption/catalytic properties of MIL-125 and NH_2-MIL-125. *Catal. Today* **2013**, *204*, 85–93, doi:10.1016/j.cattod.2012.08.014.
[34] Novaković, S.B.; Bogdanović, G.A.; Heering, C.; Makhloufi, G.; Francuski, D.; Janiak, C. Charge-density distribution and electrostatic flexibility of ZIF-8 based on high-resolution X-ray diffraction data and periodic calculations. *Inorg. Chem.* **2015**, *54*, 2660–2670, doi:10.1021/ic5028256.
[35] Mercury 2021.2.0, Program for Crystal Structure Visualisation, Exploration and Analysis from the Cambridge Crystallographic Data Center, Copyright CCDC 2001-2021, Available online: http://www.ccdc.cam.ac.uk/mercury/ (accessed on 10 July 2022)
[36] Macrae, C.F.; Sovago, I.; Cottrell, S.J.; Galek, P.T.A.; McCabe; P., Pidcock, E.; Platings, M.; Shields, G.P.; Stevens, J.S.; Towler, M.; Wood, P.A. Mercury 4.0: from visualization to analysis, design and prediction. *J. Appl. Cryst.* **2020**, *53*, 226–235.
[37] Shearer, G.C.; Chavan, S.; Ethiraj, J.; Vitillo, J.G.; Svelle, S.; Olsbye, U.; Lamberti, C.; Bordiga, S.; Lillerud, K.P. Tuned to Perfection: Ironing Out the Defects in Metal–Organic Framework UiO-66. *Chem. Mater.* **2014**, *26*, 4068–4071, doi:10.1021/cm501859p.
[38] Trickett, C.A.; Gagnon, K.J.; Lee, S.; Gándara, F.; Bürgi, H.-B.; Yaghi, O.M. Definitive molecular level characterization of defects in UiO-66 crystals. *Angew. Chem. Int. Ed.* **2015**, *54*, 11162–11167, doi:10.1002/anie.201505461.
[39] Øien, S.; Wragg, D.; Reinsch, H.; Svelle, S.; Bordiga, S.; Lamberti, C.; Lillerud, K.P. Detailed Structure Analysis of Atomic Positions and Defects in Zirconium Metal–Organic Frameworks. *Cryst. Growth. Des.* **2014**, *14*, 5370–5372, doi:10.1021/cg501386j.
[40] Zhao, P.; Bennett, T.D.; Casati, N.P.M.; Lampronti, G.I.; Moggach, S.A.; Redfern, S.A.T. Pressure-induced oversaturation and phase transition in zeolitic imidazolate frameworks with remarkable mechanical stability. *Dalton Trans.* **2015**, *44*, 4498–4503, doi:10.1039/c4dt02680b.
[41] Thommes, M.; Kaneko, K.; Neimark, A.V.; Olivier, J.P.; Rodriguez-Reinoso, F.; Rouquerol, J.; Sing, K.S. Physisorption of gases, with special reference to the evaluation of surface area and pore size distribution (IUPAC Technical Report). *Pure Appl. Chem.* **2015**, *87*, 160, doi:10.1515/pac-2014-1117.
[42] Yang, Q.-Y.; Lama, P.; Sen, S.; Lusi, M.; Chen, K.-J.; Gao, W.-Y.; Shivanna, M.; Pham, T.; Hosono, N.; Kusaka, S.; Perry, J.J.; Ma, S.; Space, B.; Barbour, L.J.; Kitagawa, S.; Zaworotko, M.J. Reversible Switching between Highly Porous and Nonporous Phases of an Interpenetrated Diamondoid Coordination

Network That Exhibits Gate-Opening at Methane Storage Pressures. *Angew. Chem. Int. Ed.* **2018**, *57*, 5684–5689, doi:10.1002/anie.201800820.

[43] Kökçam-Demir, Ü.; Goldman, A.; Esrafili, L.; Gharib, M.; Morsali, A.; Weingart, O.; Janiak, C. Coordinatively unsaturated metal sites (open metal sites) in metal-organic frameworks: design and applications. *Chem. Soc. Rev.* **2020**, *49*, 2751–2798, doi:10.1039/c9cs00609e.

[44] Lin, X.; Blake, A.J.; Wilson, C.; Sun, X.Z.; Champness, N.R.; George, M.W.; Hubberstey, P.; Mokaya, R.; Schröder, M. A porous framework polymer based on a zinc(II) 4,4'-bipyridine-2,6,2',6'-tetracarboxylate: synthesis, structure, and "zeolite-like" behaviors. *J. Am. Chem. Soc.* **2006**, *128*, 10745–10753, doi:10.1021/ja060946u.

[45] Saini, V.K.; Pires, J. Development of metal organic fromwork-199 immobilized zeolite foam for adsorption of common indoor VOCs. *J. Environ. Sci.* **2017**, *55*, 321–330, doi:10.1016/j.jes.2016.09.017.

[46] Brandt, P.; Xing, S.-H.; Liang, J.; Kurt, G.; Nuhnen, A.; Weingart, O.; Janiak, C. Zirconium and Aluminum MOFs for Low-Pressure SO_2 Adsorption and Potential Separation: Elucidating the Effect of Small Pores and NH2 Groups. *ACS Appl. Mater. Interfaces* **2021**, *13*, 29137–29149, doi:10.1021/acsami.1c06003.

[47] Brandt, P.; Nuhnen, A.; Öztürk, S.; Kurt, G.; Liang, J.; Janiak, C. Comparative Evaluation of Different MOF and Non-MOF Porous Materials for SO_2 Adsorption and Separation Showing the Importance of Small Pore Diameters for Low-Pressure Uptake. *Adv. Sustain. Syst.* **2021**, *122*, 2000285, doi:10.1002/adsu.202000285.

[48] 3P INSTRUMENTS, 3P sim, Version 1.1.0.7, Simulation and Evaluation Tool for mixSorb, 3P INSTRUMENTS 2018.

[49] Cessford, N.F.; Seaton, N.A.; Düren, T. Evaluation of Ideal Adsorbed Solution Theory as a Tool for the Design of Metal–Organic Framework Materials. *Ind. Eng. Chem. Res.* **2012**, *51*, 4911–4921, doi:10.1021/ie202219w.

[50] Zhao, P.; Lampronti, G.I.; Lloyd, G.O.; Wharmby, M.T.; Facq, S.; Cheetham, A.K.; Redfern, S.A.T. Phase Transitions in Zeolitic Imidazolate Framework 7: The Importance of Framework Flexibility and Guest-Induced Instability. *Chem. Mater.* **2014**, *26*, 1767–1769, doi:10.1021/cm500407f.

[51] Budzianowski, A.; Katrusiak, A. Pressure-frozen benzene I revisited. *Acta Crystallogr. Sect. B* **2006**, *B62*, 94-101.

[52] Katrusiak, A.; Podsiadzo, M.; Budzianowski, A. Association CH⋯π and No van der Waals Contacts at the Lowest Limits of Crystalline Benzene I and II Stability Regions. *Cryst. Growth Des.* **2010**, *10*, 3461-3465.
